# Ketamine-induced static and dynamic functional connectivity changes are modulated by opioid receptors and biological sex in rats

**DOI:** 10.1038/s41386-025-02108-0

**Published:** 2025-04-19

**Authors:** Valeria Grasso, Joseph Tennyson, Raag D. Airan, Tommaso Di Ianni

**Affiliations:** 1 Department of Psychiatry and Behavioral Sciences, University of California, San Francisco, CA 94158 USA; 2 Weill Institute for Neurosciences, University of California, San Francisco, CA 94158 USA; 3 Electrical Engineering and Computer Science, University of California, Berkeley, CA 94720 USA; 4 Departments of Radiology, Psychiatry and Behavioral Sciences, and Materials Science and Engineering, Stanford University School of Medicine, Stanford, CA 94305 USA; 5 Department of Radiology and Biomedical Imaging, University of California, San Francisco, CA 94158 USA

**Keywords:** Reward, Neurophysiology

## Abstract

Subanesthetic ketamine is currently used as a rapid-acting treatment for varied neuropsychiatric disorders. However, the mechanistic underpinnings of its therapeutic action remain unclear, and emerging clinical and preclinical evidence highlights a potential involvement of the opioid system. We used pharmacological functional ultrasound imaging data acquired during and after ketamine administration in male and female rats pretreated with naltrexone, an opioid receptor antagonist, or vehicle. We found that ketamine-induced functional connectivity changes are modulated by opioid receptor blockade, and that these responses are dependent on biological sex. Specifically, naltrexone sex-dependently altered the connectivity patterns within the medial prefrontal cortex (mPFC), a key node of the brain’s default-mode network, and between the mPFC and other functional nodes. Furthermore, ketamine produced an opioid-dependent shift toward states of increased dysconnectivity and brain entropy in male rats only. Our findings warrant further investigation into the neurophysiological underpinnings of ketamine action and potential sex-specific interactions with opioid receptors.

## Introduction

Subanesthetic ketamine has gained considerable attention as a rapid-acting treatment for neuropsychiatric disorders including treatment-resistant depression [1, 2], obsessive-compulsive disorder [3], and chronic pain [4]. However, the mechanistic underpinnings of its varied therapeutic effects have remained unclear. A popular hypothesis is that ketamine exerts its therapeutic action via noncompetitive antagonism of cortical glutamatergic *N*-methyl-D-aspartate receptors [5, 6] (NMDARs), but accumulating clinical and preclinical evidence suggests a potential role of the opioid system [7–11]. Pretreatment with naltrexone, a nonselective opioid receptor antagonist, has been shown to attenuate ketamine’s antidepressant and anti-suicidal effects in patients with treatment-resistant depression [10, 11], but subsequent work has challenged these findings [12, 13]. Another study also found a potential interference of prior opioid agonist exposure with ketamine’s antidepressant effect [14]. The putative role played by opioid signaling in ketamine’s therapeutic action raises concern for its abuse liability and potential for dependence [15], and it is therefore critical to better elucidate ketamine’s mechanism of action and safety profile [16].

To map opioid-mediated neural responses to subanesthetic ketamine with high sensitivity and high spatiotemporal resolution, we have pioneered using pharmacological functional ultrasound imaging (pharmaco-fUSI) in the acute-restraint rat model of stress [17]. fUSI is an innovative neuroimaging technology based on neurovascular coupling that provides imaging readouts comparable to functional MRI [18, 19], but it facilitates imaging of awake and freely behaving rodents [20]. We found robust opioid-mediated activations in regions of the rat brain relevant for the pathophysiology of depression and for processing of reward. Surprisingly, we also found that these opioid-mediated responses were strongly sex-dependent, as neural activity differences between naltrexone and vehicle pretreatment were only present in male rats, whereas females and gonadectomized males showed only minor differences between treatments [17]. These acute neural responses were reflected in long-term changes in postsynaptic density and behavioral sensitization.

In the present work, we sought to determine whether these opioid-mediated and sex-dependent effects of subanesthetic ketamine extend to functional connectivity measures, and in particular the connectivity of the medial prefrontal cortex (mPFC). To this end, we analyzed fUSI data from our previous study [17] using well-established methods, such as ROI-based and seed-based functional connectivity, and quantified local synchrony and dynamic brain state shifts. Based on our prior findings, we hypothesized that ketamine would alter brain functional connectivity signatures differently in male and female rats, and that these effects would depend on the availability of opioid receptors. Our results highlight complex and dynamic interactions between subanesthetic ketamine, opioid receptors, and sex factors in the connectivity of the mPFC and other functional nodes.

## Methods

### Data acquisition and pre-processing

We analyzed fUSI data from our previous study [17] to quantify ketamine’s effects on brain functional connectivity. Additional details about animal preparation, fUSI implementation, and pharmaco-fUSI recordings can be found in the referenced manuscript. All animal procedures were approved by the Institutional Animal Care and Use Committee at Stanford University. In this section, we report all the experimental details necessary for a correct interpretation of the current findings.

#### Functional ultrasound imaging acquisition

We recorded fUSI data in awake-restrained rats to assess ketamine’s action in the context of an acute stress model [21, 22]. fUSI data were acquired using a Verasonics Vantage 256 research scanner (Verasonics Inc.) connected to a linear array transducer (Vermon; 128 elements, lateral pitch of 100 μm) operating at 15 MHz. fUSI acquisitions consisted of 200 compound frames sampled at a rate of 1 kHz (200 ms observation window), and the frames were beamformed in a regular grid of pixels with in-plane resolution of 100 μm × 100 μm. The final power Doppler frame rate was 1 frame/s.

Before imaging, rats were briefly anesthetized with isoflurane, and a catheter was placed in the tail vein for vascular access. While under anesthesia, animals were placed in a plastic restraint cone (Stoelting Co.) and positioned in a custom 3-D printed restraining apparatus [23]. The ultrasound probe was carefully placed over the region of interest using a micropositioner. With the animal in the imaging apparatus, we waited for 30–45 min before data acquisition to allow for complete isoflurane clearance.

Naltrexone (10 mg/kg) and vehicle (1 mL/kg) were administered subcutaneously. Racemic ketamine (10 mg/kg) was administered intravenously after 10 min. We acquired fUSI data continuously for 50 min following ketamine administration. Each rat was imaged three times under the conditions of VEH + KET, NTX + KET, and NTX + VEH in a three-arm crossover design. This design informed the choice of within- and between-subjects factors in the subsequent statistical analyses, as appropriate. Treatment conditions were shuffled to control for potential order effects and interference from prior drug exposure. We allowed for a 7-day washout period between ketamine injections for full drug clearance.

#### Signal pre-processing

To prevent motion artifacts in the fUSI images, translational and rotational movements were corrected by applying a motion correction algorithm to the time series, as reported elsewhere [17]. A filter was used to remove registered data frames affected by excessive motion or other artifacts. Each power Doppler dataset was then registered to the Paxinos brain atlas [24] (at bregma +2.5 mm).

In the functional connectivity analyses, we filtered the CBV time series in the (0.01, 0.1) Hz range using a fourth-order Butterworth bandpass filter. fUSI fluctuations in this frequency range have been shown to have maximal spectral coherence with neuronal firing rates [25], and this range has been previously used in resting-state fUSI functional connectivity [26].

### Static functional connectivity

#### Functional connectivity matrices

**Fig. 1:**
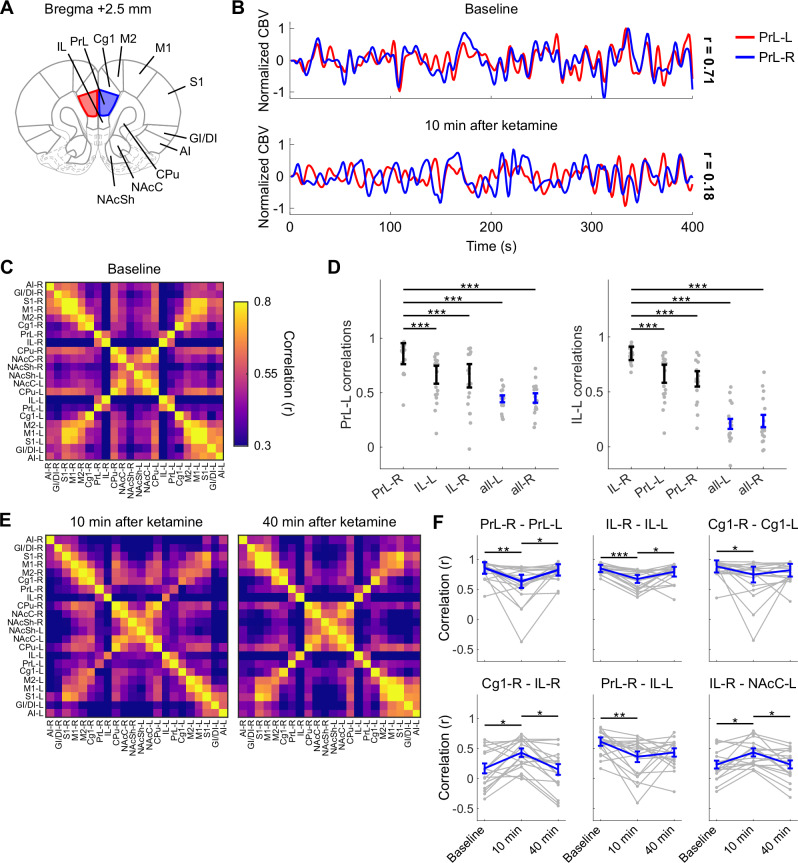
Functional connectivity changes evoked by intravenous ketamine administration. **A** Segmented brain regions of interest highlighted on the coronal rat brain atlas. **B** Representative normalized time-series of cerebral blood volume signal in the left and right PrL at baseline and 10 min after ketamine administration. **C** Functional connectivity matrix at baseline. **D** Baseline correlations of the left PrL (IL) cortex with the contralateral functional homologous, the left and right IL (PrL), and subject-level mean of all the other segmented ROIs in the left and right hemisphere. *** corrected *P* < 0.001 in a two-tailed paired *t*-test. **E** Functional connectivity matrices at 10 min and 40 min post ketamine administration. **F** Functional connectivity changes in prefrontal cortical regions. * corrected *P* < 0.05; ** corrected *P* < 0.01; *** corrected *P* < 0.001 in the Tukey’s HSD post-hoc test. Hedge’s *g* effect sizes (baseline vs 10 min): PrL-R – PrL-L: 0.92; IL-R – IL-L: 1.45; Cg1-R – Cg1-L: 0.64; Cg1-R – IL-R: −0.79; PrL-R – IL-L: 0.77; IL-R – NAcC-L: −0.78. Data presented as mean ± s.e.m. *N* = 18 rats, 9 female. IL infralimbic cortex, PrL prelimbic cortex, Cg1 cingulate area 1, M2 secondary motor cortex, M1 primary motor cortex, S1 primary somatosensory cortex, GI/DI granular/dysgranular insular cortex, AI anterior insular cortex, CPu caudate putamen, NAcC nucleus accumbens core, NAcSh nucleus accumbens shell.

**Fig. 2:**
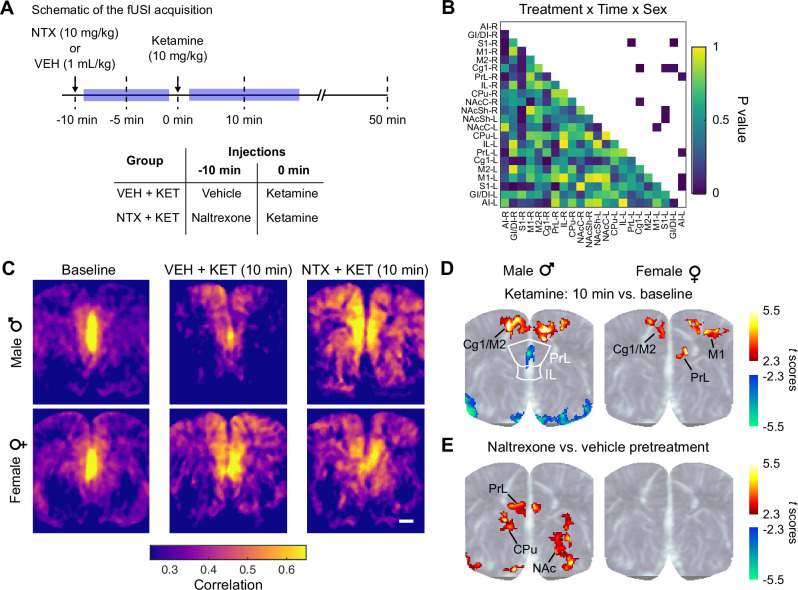
Ketamine functional connectivity changes depend on opioid receptor availability and biological sex. **A** Rats were administered either vehicle (VEH) or naltrexone (NTX; 10 mg/kg) followed by ketamine (KET; 10 mg/kg, i.v.) after 10 min. Functional connectivity was quantified at the pre-ketamine baseline (−5 min) and 10 min post-ketamine administration. **B** *P* values from three-way ANOVA with treatment (within-subjects), time (within-subjects), and sex (between-subjects) factors stratified by ROI pair. *N* = 18 rats, 9 females. **C** Seed-based connectivity maps in male and female rats at baseline and at 10 min post-ketamine in the VEH + KET and NTX + KET conditions. The correlation maps were seeded in the medial prefrontal cortex (bilateral PrL and IL). The seed is shown as the white contour. Plots are mean of *N* = 9 male and *N* = 9 female rats. Scale bar: 1 mm. **D**, **E** Statistical parametric maps of the group-level seed-based correlation analyses. The *t* scores were calculated by contrasting the pixel-wise correlations in the VEH + KET condition at the 10 min time point vs. baseline (**B**) and in the NXT + KET vs. VEH + KET conditions at the 10 min time point (**C**). Statistically significant clusters are displayed overlaid on a power Doppler template. Two-tailed paired *t*-test, cluster-corrected *P* < 0.05.

To calculate the connectivity matrices, we segmented 11 bilateral ROIs (Fig. 1A) and averaged the filtered temporal CBV signals in each ROI. We then calculated ROI-pair-wise Pearson’s correlations using a 9-min interval centered at the −5 min time point (baseline) and 15-min intervals centered at the 10 min and 40 min time points (Fig. 2A). We applied the Fisher’s Z transformation to normalize the ROI-based correlation values before performing any further operations. The processed z values were then converted back to the correlation domain for visualization purposes.

#### Seed-based connectivity

For the seed-based connectivity maps, we segmented the filtered CBV time series in the seed region defined as the bilateral PrL and IL. We performed Pearson’s correlations between the resulting temporal signal and the filtered CBV signals in all the image pixels. We calculated the seed-based maps using a 9-min interval centered at −5 min (baseline) and a 15-min interval centered at 10 min. We smoothed the connectivity maps using a median filter with a 0.3 × 0.3 mm^2^ kernel. We applied the Fisher’s Z transformation to normalize the pixel-wise correlation values before performing any further operations, and the processed z values were then reverse transformed to the correlation domain for visualization purposes.

For the group-level analyses, we used pixel-wise statistical inference to analyze differences in the correlation values between treatments and time points. Individual correlation maps were registered to the template atlas space by performing a rigid transformation, and *t* scores were calculated for the contrasted groups (10 min vs. baseline, NTX + KET vs. VEH + KET; two-tailed paired *t* test). The *t* scores were thresholded for significance and were corrected for multiple comparisons using a cluster-size threshold of 37 contiguous pixels. The threshold was determined via Monte Carlo simulations using the 3dClustSim program of the AFNI library [27] to obtain an overall cluster-*P* < 0.05, family-wise error rate corrected.

### Kendall’s coefficient of concordance

To quantify the synchronization of local oscillations in the fUSI signals, we calculated the Kendall’s coefficient of concordance between the fUSI time series for each pixel within a cluster of 9 neighboring pixels [28]. The KCC was calculated as:

$$\frac{12}{{k}^{2}\,({n}^{3}-n)} {\sum }_{i=1}^{n}{\left({\sum }_{j=1}^{k}{R}_{{ij}}-k\bar{R}\right)}^{2}$$

where $k$ is the number of pixels in the clusters, $n$ is the number of time points, ${R}_{{ij}}$ is the rank of the $i$-th time point in the $j$-th pixel, and $\bar{R}$ is the mean rank across all pixels and time points. The KCC does not make any assumptions on the probability distribution and assumes a value between 0 (minimum synchrony) and 1 (maximum synchrony). We z-scored the KCC values at the animal level by calculating the mean and standard deviation of the 4 resulting maps (baseline and 10 min; VEH + KET and NTX + KET). The z-scored KCC maps were then segmented to perform group-level analyses between time points and treatment conditions in the different ROIs.

### Dynamic functional connectivity

We implemented an unsupervised algorithm to identify connectivity patterns (brain states). We segmented the filtered CBV time series in each ROI, then we calculated functional connectivity matrices by applying the sliding window connectivity (SWC) method. We computed ROI pair-wise Pearson’s correlations from 30-s temporal windows with 1-s step size, giving a total of *N* = 3572 matrices per imaging session (−20 min to 40 min). Each temporal SWC sequence was reshaped into a linear mixture matrix, where the number of rows was the number of time points *N* and the number of columns was the total number of ROI pairs (484). The reshaped maps were concatenated across subjects (*n* = 18, 9 females) and treatment conditions (VEH + KET and NTX + KET) along the time variable into a global matrix. The concatenated matrix was then used as input for the k-means algorithm (Scikit-Learn, v. 1.4.1 in Python v. 3.12).

To determine the optimal number of clusters, we applied the elbow method based on singular value decomposition [29]. We first computed the relative variance for each singular value (calculated as the cumulative variance of the ranked singular values over the total variance) (Supplementary Fig. 5A). To reduce the singular-value dimensionality, we identified the smallest number of components for which the cumulative variance was greater than or equal to 99% of the total variance (225 singular values). Then, to identify the optimal number of clusters we computed the second derivative of the largest 225 singular values to identify the point where the rate of change of the singular values was minimum (elbow point). This analysis indicated an optimal number of 5 clusters (Supplementary Fig. 5B). We sorted the 5 clusters based on the mean within-cluster connectivity.

Following k-means clustering, one single state was assigned to each time point. To perform group-level analyses, we calculated the time fraction, defined as the fraction of time spent in each state, and the dwell time, defined as the average amount of uninterrupted time spent in each state, in the pre-ketamine (−18.5 min to −1.5 min excluding 3 min at the time of pretreatment injection) and post-ketamine (2.5 min to 17.5 min) periods.

#### Markov entropy

The Markov entropy measures the unpredictability or disorder in a system that can be described as a stochastic process described by a Markov chain. To calculate the Markov entropy, we calculated the probability of transition between states ${P}_{i,j}$ as the ratio between the transition counts from state $i$ to state $j$ dived by the total transitions from state $i$. We then calculated the entropy from the transition probabilities as $H=-{\sum }_{i}^{k}{\sum }_{j}^{k}{P}_{i,j}\log ({P}_{i,j})$, where $i,j\in 1,\ldots 5$. Higher entropy implies a more dynamically complex and variable system.

### General statistical analysis

Rats were randomly assigned to treatment conditions. When within-subject factors were present in the ANOVA, Mauchly’s test for sphericity was performed to determine whether the sphericity assumption was satisfied. In cases where the assumption was violated, we used a Greenhouse-Geisser adjustment to the degrees of freedom. Pairwise post-hoc comparisons were performed in case of significant ANOVA effects using the Tukey’s honestly significant difference (HSD) test with multiple comparisons correction. All tests were two-tailed. We calculated effect sizes using Hedge’s *g*. Statistical tests, sample sizes *n*, *P* values, and effect sizes *g* are reported for each analysis in the text and figure captions. All statistical analyses were performed using custom scripts in R Studio and MATLAB.

## Results

### Subanesthetic ketamine transiently modulates brain functional connectivity

We quantified resting-state functional connectivity changes induced by intravenous (i.v.) subanesthetic ketamine (10 mg/kg). This ketamine dose reliably produces antidepressant-like effects in rat behavioral models [30], and in both male and female rats, it increases the expression of the postsynaptic density protein PSD-95 in the mPFC and produces locomotor sensitization [17]. We segmented 11 bilateral regions of interest (ROIs) in the pharmaco-fUSI data and averaged the temporal CBV signals in each ROI (Fig. 1A) (*N* = 18 rats, 9 females). We calculated ROI pair-wise Pearson’s correlations to compute the functional connectivity matrices (Fig. 1B). We first quantified the test-retest reliability by calculating the baseline connectivity in three consecutive imaging sessions 7 days apart. There was a significant effect of ROI pair (*F*_230,3910_ = 17.63, *P* < 0.001), but we found no significant effect of session (*F*_2,34_ = 1.27, *P* = 0.295) nor a significant ROI pair × session interaction (*F*_460,7820_ = 0.76, *P* = 1) (Supplementary Fig. 1), indicating that the intra-subject variability over repeated sessions was negligible.

At baseline, the matrices showed a pattern of high connectivity in functionally homologous contralateral brain regions coherent with prior studies [31, 32], and clusters of highly correlated ROI pairs within the sensory (S1) and motor (M1, M2) cortices and within the basal ganglia (caudate putamen (CPu) and nucleus accumbens core (NAcC) and shell (NAcSh); Fig. 1C). We observed a significant effect of ROI pair (*F*_230,3680_ = 11.04, *P* < 0.001) but no significant effect of sex (*F*_1,16_ = 0.38, *P* = 0.558) and no significant sex × ROI pair interaction (*F*_230,3680_ = 1.04, *P* = 0.315). Interestingly, the prelimbic (PrL) and infralimbic (IL) cortices, two sub-territories of the rodent mPFC [33], were highly correlated with each other bilaterally but showed low baseline connectivity with all the other ROIs (Fig. 1D).

Ketamine produced widespread changes in functional connectivity, with effects most prominent in functionally homologous contralateral regions and in the insular cortices. These connectivity changes were transient and resolved nearly completely 40 min after ketamine administration (Fig. 1E, F, Supplementary Fig. 2). We found a significant effect of ROI pair (*F*_230,3680_ = 14.8, *P* < 0.001) and a significant interaction between time (pre-ketamine baseline, and 10- and 40-min post-ketamine) and ROI pair (*F*_460,7360_ = 2.06, *P* < 0.001). Importantly, we found no main effect of sex (*P* = 0.237), and no significant sex × ROI pair (*P* = 0.875), sex × time (*P* = 0.238), and sex × ROI pair × time (*P* = 0.463) interactions. Ketamine-induced connectivity changes extended to all the ROIs in the mPFC, including PrL, IL, and the cingulate area 1 (Cg1) (Fig. 1F), as well as in the insular cortex, including the granular/dysgranular insula (GIDI) and the anterior insula (AI) (Supplementary Fig. 2). In most of these ROIs, ketamine reduced the correlation at the 10 min time point. Upon administration of an inert solution (1 mL/kg saline, i.v.; *N* = 8 male rats), we found a significant effect of ROI pair (*F*_230,1610_ = 5.2, *P* < 0.001) but no significant effect of time (*F*_1.1,7.7_ = 1.88, *P* = 0.211) and no significant ROI pair × time interaction (*F*_460,3220_ = 0.91, *P* = 0.91). This indicates that, while the different ROI pairs showed characteristic patterns of connectivity, as expected, a saline injection did not alter those patterns, as there were no significant differences between the baseline and the 10- and 40-min post-injection time points. Therefore, the injection alone produced no significant effect and was not sufficient to explain the connectivity changes observed upon ketamine administration. Taken together, these results confirm that pharmaco-fUSI can reliably image functional connectivity over repeated sessions, and that ketamine produces transient and region-dependent connectivity changes.

### Opioid receptor blockade modulates ketamine-induced functional connectivity changes differently in male and female rats

We have previously reported that the region-specific neural activations evoked by ketamine are robustly modulated by pretreatment with naltrexone, an opioid receptor antagonist [9, 17], and that these effects are dependent on biological sex [17]. Here we sought to determine if these opioid- and sex-dependent differences extend to measures of brain functional connectivity. To this end, we used pharmaco-fUSI data recorded in male and female rats pretreated with either naltrexone (NTX; 10 mg/kg, s.c.) or vehicle (VEH) before ketamine (KET) administration (Fig. 2A). This naltrexone dose produced nearly total mu opioid receptor (MOR) occupancy in the mouse brain [34] and suppressed the effect of (*S*)-ketamine on acute locomotion [7].

To quantify the effects of naltrexone administration alone (NTX + VEH condition), we performed a three-way ANOVA with within-subjects factors of ROI pair and time (-15 min: pre-naltrexone; −5 min; 10 min; 40 min) and between-subjects factor of sex (Supplementary Fig. 3). We found a significant effect of ROI pair (*F*_230,3680_ = 9.31, *P* < 0.001) and no significant main effect of time (*P* = 0.259), but there was a significant ROI pair × time interaction (*F*_690,11040_ = 1.16, *P* = 0.003). However, in the Tukey’s HSD post-hoc test, only a single ROI pair (GIDI-R - S1-R ROI in males) showed significant differences between time points (*P* = 0.045). Importantly, we found no significant main effect of sex nor interactions with the sex factor (*P* > 0.192) in these analyses.

Opioid receptor blockade by naltrexone administration modulated the functional connectivity changes evoked by ketamine (Fig. 2B). In the NTX + KET condition, we found significant main effects of ROI pair (*F*_230,3680_ = 8.08, *P* < 0.001) and time (*F*_1,16_ = 4.52, *P* = 0.049) and significant ROI pair × time (*F*_230,3680_ = 1.99, *P* < 0.001) and sex × ROI pair × time (*F*_230,3680_ = 1.25, *P* < 0.001) interactions. To identify region-specific effects, we performed three-way ANOVAs stratified by ROI pair with the treatment, time, and sex factors (Fig. 2B). We found significant treatment × time × sex interactions in several ROI pairs in the mPFC and in the insula (e.g., Cg1-R - Cg1-L: *F*_1,16_ = 12.56, *P* = 0.003; PrL-L - S1-R: *F*_1,16_ = 8.18, *P* = 0.011; PrL-R - AI-L: *F*_1,16_ = 5.24, *P* = 0.036; GIDI-L - S1-R: *F*_1,16_ = 11.11, *P* = 0.004) (Fig. 2B; see Supplementary Table 1 for a complete report of all the factors and ROI pairs). To further dissect these sex-dependent effects, we quantified the functional connectivity changes evoked by ketamine with and without naltrexone pretreatment in pharmaco-fUSI data recorded in gonadectomized male rats [17]. We performed two-way ANOVAs stratified by ROI pair with the treatment and time factors. We found significant treatment × time interactions in 3 ROI pairs (GIDI R - M2 L: *F*_2,12_ = 4.96, *P* = 0.027; M1 R - GIDI L: *F*_2,12_ = 8.64, *P* = 0.005; and S1 R - GIDI L: *F*_2,12_ = 4.22, *P* = 0.041; Supplementary Fig. 4). In contrast, the same analysis in intact males returned significant effects in 12 ROI pairs, including many of the regions where significant difference were recorded in the previous analyses (Cg1 R - Cg1 L: *F*_1,8_ = 7.44, *P* = 0.026; PrL R - PrL L: *F*_1,8_ = 5.49, *P* = 0.047; Cg1 R - PrL R: *F*_1,8_ = 10.49, *P* = 0.012 and Cg1 L - PrL L: *F*_1,8_ = 10.1, *P* = 0.013; IL R - GIDI L: *F*_1,8_ = 13.24, *P* = 0.007). Altogether, these results demonstrate the presence of complex and dynamic interactions between ketamine, opioid receptors, and sex that appear to modulate the functional connectivity changes produced by ketamine in cortical and limbic regions, in particular in the mPFC and insula.

### Seed-based connectivity of the medial prefrontal cortex and regional homogeneity

The mPFC is a primary node of the default-mode network (DMN), a network of functionally connected brain regions that plays a pivotal role in the continuous monitoring of sensory and affective input at rest to guide behavioral responses to environmental contingencies [35–37], and whose connectivity is dysregulated in major depressive disorder [38–40]. Motivated by the fact that our prior analyses consistently revealed significant effects in mPFC regions, we sought to obtain a deeper insight into the spatial distribution of the functional connectivity changes induced by ketamine and potential modulatory effects of sex and opioid receptor blockade. We computed seed-based, whole-slice connectivity maps using the bilateral PrL and IL sub-regions of the mPFC as the seed region (Fig. 2C). Baseline fUSI signals showed strong correlation within the seed in both male and female rats, in agreement with the connectivity matrix of Fig. 1C. In male rats, ketamine partially attenuated these correlations within the seed and enhanced the correlation between the seed and dorsal regions of the mPFC (Cg1/M2) (corrected *P* < 0.05; Fig. 2D). In females, ketamine increased the connectivity of the seed with dorsal areas of the mPFC (Cg1/M2) and with the primary motor cortex (M1), as well as with a smaller cluster within the seed (Fig. 2D). In male rats, naltrexone pretreatment modulated ketamine-induced connectivity changes and enhanced the correlation within the seed and between the seed and areas of the dorsal and ventral striatum (CPu and NAc). No significant clusters were found in females.

**Fig. 3:**
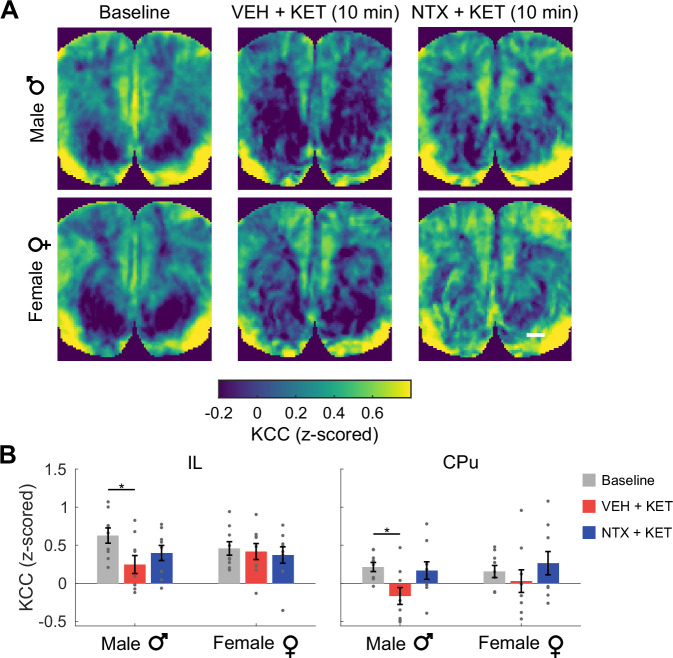
Ketamine effects on the regional homogeneity. **A** Maps of Kendall’s coefficient of concordance (KCC) in male and female rats at baseline and at 10 min post-ketamine in the VEH + KET and NTX + KET conditions. Scale bar: 1 mm. **B** Segmented KCC in cortical and limbic regions. *corrected *P* < 0.05 in the Tukey’s HSD post-hoc test. Hedge’s *g* effect sizes: IL: −0.72; CPU: −0.82. Data presented as mean ± s.e.m. *N* = 18 rats, 9 female.

To further investigate ketamine-induced changes in local connectivity, we computed regional homogeneity maps as measured by the pixel-wise Kendall’s coefficient of concordance [28] (KCC) in a 9-pixel neighborhood. The KCC quantifies local synchrony in the entire slice without relying on an a-priori seed choice. In line with our previous results, we found that ketamine produced changes in the KCC that were modulated by naltrexone differently in male and female rats (Fig. 3A). In males in the VEH + KET condition, we observed significant differences between the baseline and post-ketamine KCC in the IL (corrected *P* = 0.045 in the Tukey’s HSD test) and CPu (*P* = 0.03) (Fig. 3B). Overall, these results indicate that ketamine produces significant connectivity and local synchrony changes in the mPFC, and that these effects are again modulated by opioid receptor blockade in a sex-dependent manner.

### Dynamic functional connectivity and brain state shifts induced by ketamine

**Fig. 4:**
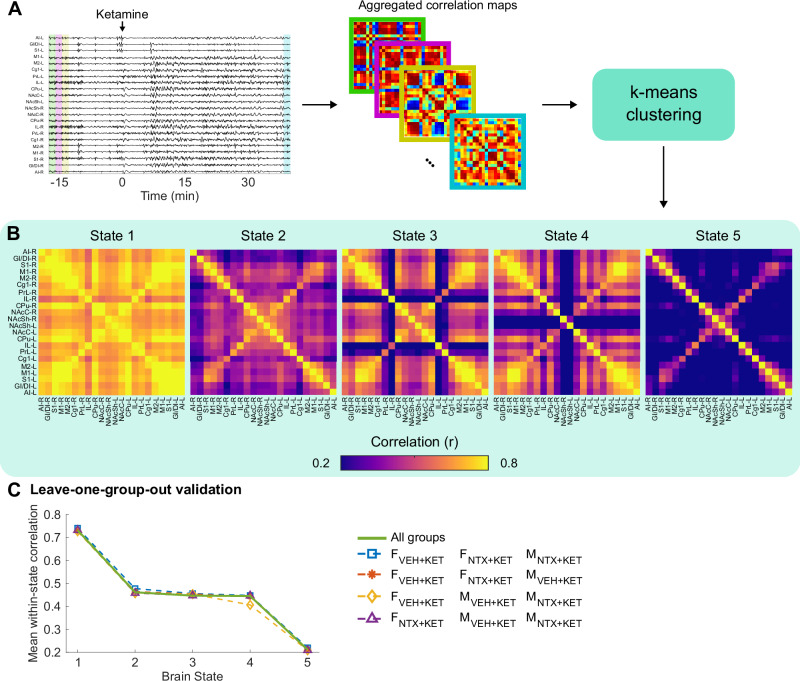
Schematic of the dynamic functional connectivity processing. **A** Sliding-window functional connectivity maps were calculated in 30-s time intervals (1-s step size), and unsupervised clustering was performed via k-means to identify prominent clusters (brain states). **B** Brain states identified by k-means clustering and sorted based on the mean correlation within each state. **C** Results of the leave-one-group-out validation showing that k-means yields equivalent clusters in all the five conditions. The connectivity maps for all the conditions are in Supplementary Fig. 6.

The previous functional connectivity and local synchrony analyses revealed the presence of a complex, dynamic interplay between ketamine, naltrexone, and sex that manifested in both global and local connectivity changes. However, these analyses compressed the temporal domain by integrating the information over several minutes. To quantify ketamine’s effects on brain dynamics with higher temporal resolution, we used a dynamic functional connectivity approach with unsupervised k-means clustering [41, 42]. We first applied a 30-s sliding-window correlation procedure to obtain a temporal sequence of connectivity matrices (Fig. 4A). Then, to identify a set of discrete states that are visited by the brain over time, we aggregated the temporal connectivity sequences across all subjects, time points, and treatment conditions and performed k-means clustering to identify the most prominent clusters or brain states. To select the optimal number of states in a data-driven way, we applied the elbow method based on singular value decomposition. This analysis indicated that five clusters were the optimal number beyond which the marginal gain of adding another cluster was minimum (Supplementary Fig. 5). The five prominent states identified by this clustering approach show distinct, recurrent connectivity patterns (Fig. 4B). We sorted these states from highly connected (state 1) to highly disconnected (state 5) based on the mean correlation within each state (Fig. 4B-C). To further assess the robustness of this clustering approach, we performed a leave-one-group-out validation, where we repeated the clustering leaving out one of the four conditions (males or females, NTX + KET or VEH + KET treatment). This validation confirmed that k-means provided equivalent clustering results in all the repetitions (Fig. 4C and Supplementary Fig. 6). Notably, we also obtained equivalent clustering results using the instantaneous phase difference between CBV signals from different ROI pairs (Supplementary Fig. 7), which does not rely on temporal windowing and Pearson’s correlations [26], but we continued the group-level analyses with the sliding-window approach for consistency with most of the prior literature.

**Fig. 5:**
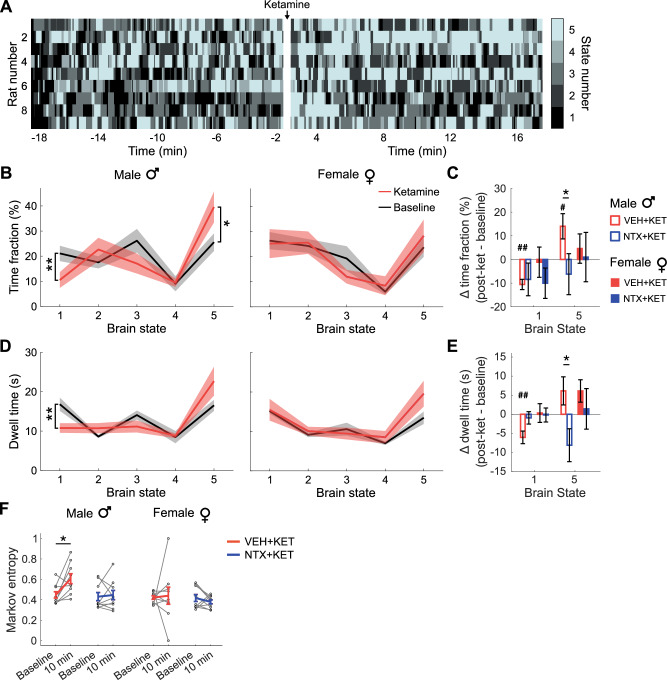
Ketamine induces a shift toward disconnected brain states that is dependent on opioid receptor availability. **A** Brain state transitions in male rats pre- and post-ketamine administration. **B**, **D** Time fraction and dwell time quantified in the dynamic functional connectivity analysis in male and female rats. Two-tailed paired *t*-test, *P < 0.05, **P < 0.01. **C**, **E** Time fraction and dwell time change (ketamine – baseline) in brain states 1 (most correlated) and 5 (least correlated) in male and female rats and with naltrexone and vehicle pretreatment. *P < 0.05 in a two-tailed paired *t*-test. ^#^*P* < 0.05, ^##^*P* < 0.01 in a two-tailed one-sample *t*-test. **F** Markov entropy. *P < 0.05 in a two-tailed paired *t*-test. Hedge’s *g* effect sizes for all the plots are reported in Supplementary Table 1. Data presented as mean ± s.e.m.

In male rats, ketamine administration produced a shift toward the most disconnected state at the expense of the most connected one (Fig. 5A). This effect was reflected in both the time fraction (i.e., the total fraction of time spent in each state) and the dwell time (i.e., the average time uninterruptedly spent in a state before a transition) (Fig. 5B, D). Specifically, we observed a significant decrease of both time fraction (two-tailed paired *t*-test; *P* = 0.002) and dwell time (*P* = 0.009) for state 1, and a significant time fraction increase for state 5 (*P* = 0.039). These effects were modulated by sex and by naltrexone pretreatment. Indeed, we observed significant differences between the NTX + KET and VEH + KET conditions in the time fraction change (*P* = 0.032; Fig. 5C) and dwell time change (*P* = 0.035; Fig. 5E) for brain state 5 in male but not female rats. These results indicate that state transitions in males had a higher chance to converge onto the most disconnected brain state, while in females the overall rate of exploration was similar for the different treatment conditions.

Finally, we quantified the Markov entropy to evaluate the degree of intrinsic variance and signal diversity and to assess how these were altered by subanesthetic ketamine. Prior evidence suggests increased entropy during altered states of consciousness induced by psilocybin, LSD, and ketamine administration [43–45]. Consistently, we found that ketamine increased the entropy in male rats at the 10-min time point compared to the baseline (two-tailed, paired *t*-test; *P* = 0.024), and this effect was suppressed by naltrexone pretreatment (*P* = 0.739) and was not evident in female rats in either treatment condition (*P* > 0.332) (Fig. 5F).

## Discussion

The principal finding of this study is that subanesthetic ketamine induces acute functional connectivity changes that are dependent on opioid receptor availability. In some brain regions, these opioid-dependent connectivity changes are also sex-specific. In particular, we found prominent interactions between ketamine, opioid receptors, and biological sex in the connectivity of the mPFC, a key node of the rat anterior DMN. In addition, our dynamic functional connectivity analyses revealed the occurrence of dynamic brain state shifts, which were also sex-dependent and modulated by opioid receptors. These results complement and extend our prior findings of opioid- and sex-dependent ketamine responses in cortical and limbic regions that are important for cognitive and affective functions and for processing of reward [17].

While the influence of biological sex on the opioid-mediated neurophysiological and behavioral responses to subanesthetic ketamine has not been thoroughly characterized, sex-divergent effects have been reported in the pharmacokinetics of ketamine and its metabolites, as well as in other neurophysiological and behavioral measures, both in humans and in rodents [30, 46]. Notably, a recent study found opioid- and sex-dependent hyperlocomotion induced by ketamine in mice [47], in agreement with our findings, and sex differences have been reported in the analgesic and reinforcing effects of opioid drugs, both in animals and in humans [48, 49]. Sex-specific effects have also been observed with other psychoactive compounds, such as psilocin and psilocybin, in rats [50, 51] and mice [52]. This growing body of literature provides evidence that sex-dependent effects may be pervasive in neuropsychiatric disorders and in pharmacological treatments, and that biological sex may help explain much of the variability observed in human trials. In this context, preclinical animal models offer an important opportunity to isolate the contribution of factors related to biological sex, such as gonadal hormones, independent of social constructs [53].

While we cannot directly identify the opioid receptor subtypes responsible for the effects that we observed in our experiments, as naltrexone is a nonselective opioid receptor antagonist, several lines of evidence point toward MOR binding and activation as the likely mechanism underlying these opioid receptor-mediated responses to ketamine. Levinstein et al. showed that (*S*)-ketamine occupies and activates MORs in some of the regions where we observed significant opioid-mediated effects with racemic ketamine, including the mPFC and striatum [9]. In addition, repeated (*S*)-ketamine administration reduced the density of MORs but did not alter the density of NMDARs and kappa opioid receptors (KORs). Both (*S*)- and (*R*)-ketamine show stronger binding potential at MORs (~65% inhibition for (*S*)-ketamine and ~30% inhibition for (*R*)-ketamine at 10 µM) than at KORs (~35% inhibition for (*S*)-ketamine and ~13% inhibition for (*R*)-ketamine at 10 µM), while delta opioid receptors show minimal binding with either stereoisomer [7]. A selective MOR antagonist (beta-funaltrexamine) but not a selective KOR antagonist (norbinaltorphimine) effectively blocked ketamine-induced hyperlocomotion in a recent study [47]. Similar studies using more specific opioid receptor antagonists may help further clarify the precise mechanisms involved in our observed sex differences.

### Functional connectivity of the medial prefrontal cortex and relevance for the default-mode network

The mPFC is a hub of the brain’s DMN, a network created by the coherent organization of anatomically distinct but functionally connected brain nodes whose activity is highly correlated during wakeful rest and is suppressed by salient stimuli and by cognitively demanding tasks [37]. Numerous studies have associated cognitive deficits in major depressive disorder, including ruminative responding, to dysregulated connectivity within the DMN [40] and between the DMN and other brain networks [38, 39]. Therefore, it has been hypothesized that antidepressant treatments would normalize aberrant DMN connectivity, and several studies have confirmed this hypothesis with different therapeutic interventions [40, 54–56], including ketamine [57, 58]. Accordingly, we found that, in male rats, ketamine partially decreased the correlation within the mPFC seed while increasing the correlation between the seed and other mPFC regions including Cg1, a rodent homolog of the human anterior cingulate cortex [33]. Furthermore, ketamine significantly decreased the local synchrony in some of these regions, as measured by the Kendall’s coefficient of concordance, in agreement with a previous report [59]. Our current results add to the existing knowledge by revealing that these connectivity changes may be dependent on opioid receptor availability and highlighting potential sex-specific mechanisms. Follow up studies should investigate the role of opioid receptors and biological sex in modulating ketamine’s neurophysiological responses in the entire DMN (including other hubs, such as the posterior cingulate cortex in humans and the retrosplenial cortex in rodents) and between the DMN and other relevant regions like the anterior insula, anterior cingulate cortex, and striatum.

### Dynamic functional connectivity

Static functional connectivity analyses assume that the coupling between brain regions is relatively stationary within the acquisition time window (typically >5 min, and between 9 and 15 min in our study). In contrast, dynamic functional connectivity can characterize time-varying, reoccurring brain connectivity patterns and can identify changes in the repertoire of brain states in response to a given perturbation [60, 61]. This approach has been previously used to characterize connectivity signatures of schizophrenia [62] and to identify latent brain state dynamics that predict performance in a working memory task [63]. Dynamic functional connectivity processing has also been applied to fUSI data to reveal aberrant networks in preterm neonates [26] and in a rat model of sustained inflammatory pain [64].

Our dynamic functional connectivity analyses showed that, in male rats without naltrexone pretreatment, ketamine produced a transient shift toward the most disconnected brain state at the expense of the most connected one. Our results are in agreement with a previous report of increased entropy in the dynamic state occupancy and state transitions during a subanesthetic ketamine challenge using source-localized electroencephalography in healthy humans [65]. Consistent with our static functional connectivity results, we found that opioid receptor blockade and biological sex significantly altered the dynamic connectivity changes induced by ketamine.

### Limitations

Our study has some limitations. First, we only focused our functional connectivity analyses in a single brain slice. Therefore, we could not determine how ketamine affects the functional connectivity between the mPFC and other key regions of the DMN, such as the retrosplenial cortex, and the effect of sex and opioid receptor blockade on the entire network. In addition, the sex dependence may be related to differential patterns of connectivity that are missed by the limited 2-D imaging setup. Future work should leverage whole-brain fUSI implementations to measure brain-wide connectivity changes.

The CBV signals recorded by fUSI might be affected by nonspecific cardiovascular responses that may confound some of the findings of the current study. To mitigate this concern, in our prior study we quantified the correlation between fUSI signals and intracranial electrocorticography recordings, and we found that fUSI signals closely track neural activity in the gamma frequency band, in agreement with previous reports [17, 25]. In addition, we found no significant correlations between the CBV changes and the intrinsic vascularization levels in the different brain regions [17]. Nevertheless, confounding cardiovascular effects cannot be completely ruled out in our current analyses.

In female rats we did not control for the estrous cycle [66]. Prior studies indicate that the neurophysiological effects of ketamine are not dependent on the estrous phase [67], and surgical removal of the ovaries did not alter the plasma levels of ketamine and its metabolites [46]. Moreover, we used a randomized study design that should mitigate any confounds related to the estrous phase in the group-level analyses, as female rats were imaged repeatedly in different phases of the cycle. However, we cannot rule out that naltrexone alters the pharmacokinetics of ketamine in a sex-divergent manner, and future studies should quantify dose-dependent effects of both naltrexone and ketamine on the functional connectivity measures.

Finally, our functional connectivity recording only extended to 40 min after ketamine administration; therefore, our results should be interpreted as acute neural activity responses. Future work should investigate the chronic responses to subanesthetic ketamine and potential interactions with opioid receptors in that experimental setting.

In conclusion, our results suggest that ketamine induces acute static and dynamic functional connectivity changes that may depend on biological sex and may be modulated by opioid receptor blockade. Taken together with our prior studies [9, 17], our current results warrant further investigation into the neurophysiological underpinnings of ketamine action and potential sex-dependent interactions with opioid receptors. This is a follow-up analysis of data from a prior study; independent replication of our findings in a new cohort is warranted.

## Supplementary information

Supplementary Information

Supplementary Table 1

Supplementary Table 1

## Data Availability

Data and codes are available from the corresponding authors upon reasonable request.
