# Supplementary material for: Ketamine-induced static and dynamic functional connectivity changes are modulated by opioid receptors and biological sex in rats
Source: Neuropsychopharmacology. 2025 Apr 19;50(11):1695–705. doi: 10.1038/s41386-025-02108-0 (PMC12436656; doi:10.1038/s41386-025-02108-0)
Supplement: Supplementary file 1 — Supplementary Information [file 41386_2025_2108_MOESM1_ESM.pdf]

Ketamine-induced static and dynamic functional connectivity changes are modulated by opioid receptors and biological sex in rats

Valeria Grasso[1,2], Joseph Tennyson[1,2,3], Raag D. Airan[4], Tommaso Di Ianni[1,2,5*]

[1] Department of Psychiatry and Behavioral Sciences, University of California, San Francisco, CA 94158 USA

[2] Weill Institute for Neurosciences, University of California, San Francisco, CA 94158 USA

[3] Electrical Engineering and Computer Science, University of California, Berkeley, CA 94720 USA

[4] Departments of Radiology, Psychiatry and Behavioral Sciences, and Materials Science and Engineering, Stanford University School of Medicine, Stanford, CA 94305 USA

[5] Department of Radiology and Biomedical Imaging, University of California, San Francisco, CA 94158 USA

*Corresponding author:

Tommaso Di Ianni, Ph.D.
Email: tommaso.diianni@ucsf.edu

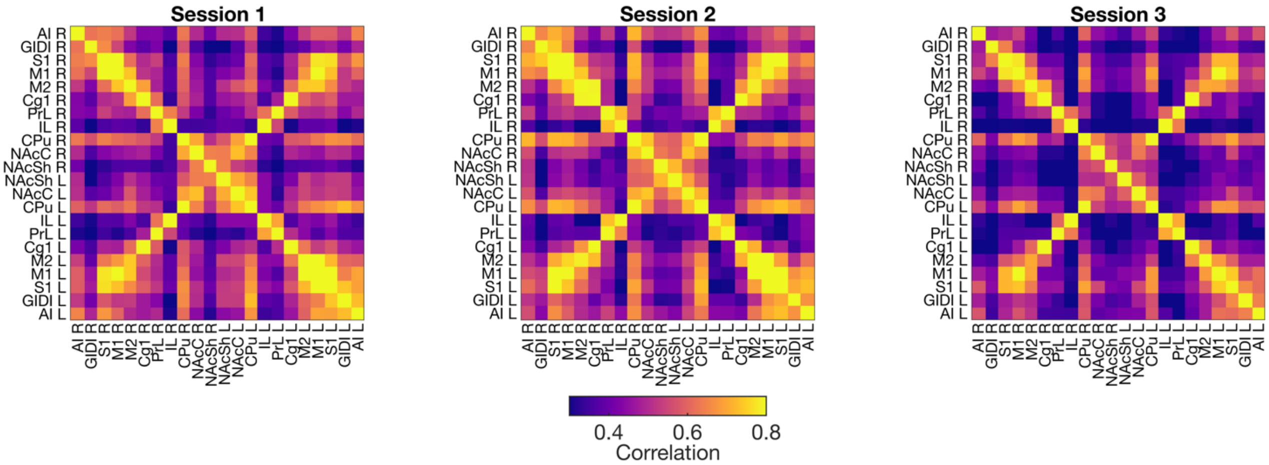

Supplementary Figure 1: Test-retest reliability of functional ultrasound imaging functional connectivity. Connectivity matrices from three consecutive imaging sessions 7 days apart. Two-way ANOVA with session and ROI pair factors: session, $F_{2,34} = 1.27$, $P = 0.295$; ROI pair, $F_{230,3910} = 17.63$, $P < 0.001$; ROI pair × session interaction. $F_{460,7820} = 0.76$, $P = 1$. Matrices are mean of $N = 18$ rats, 9 females.

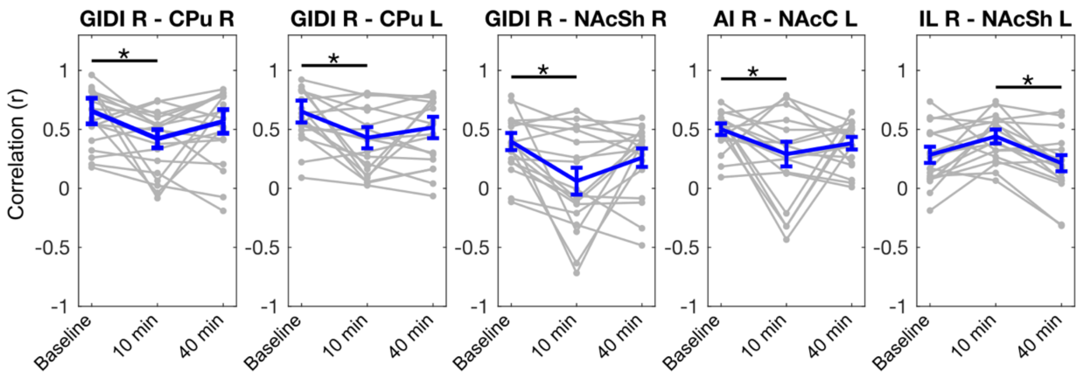

Supplementary Figure 2: Ketamine-evoked functional connectivity changes in selected brain regions. Data presented as mean ± s.e.m. * corrected $P < 0.05$ in the Tukey's HSD post-hoc test. N = 18 rats, 9 females.

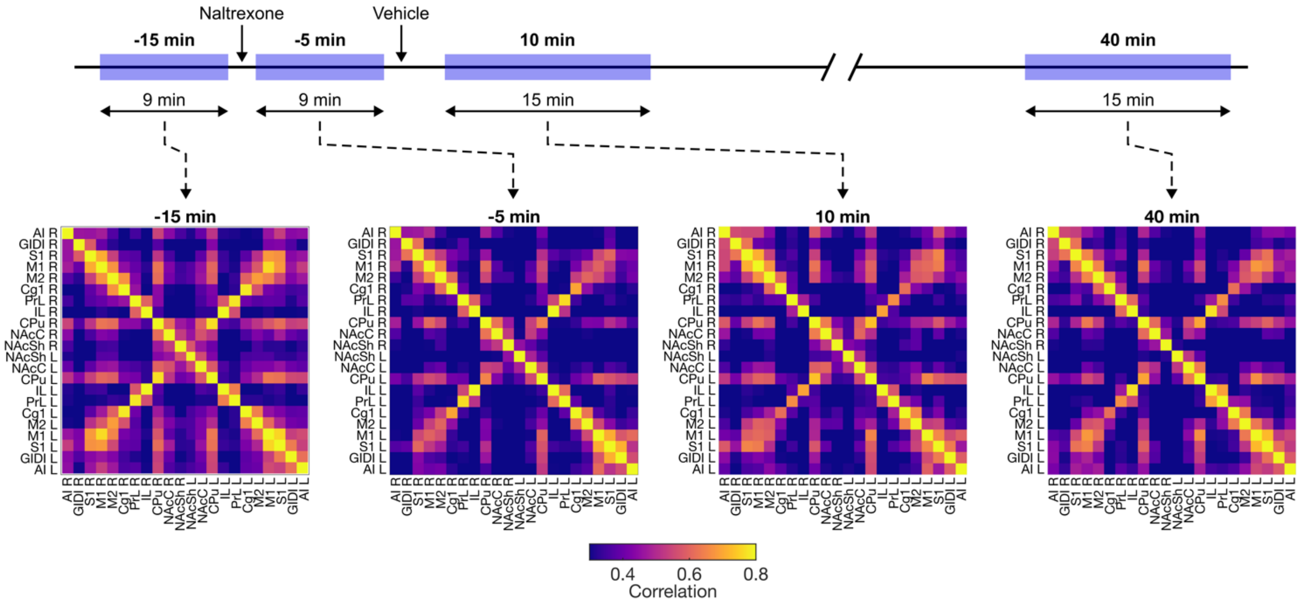

Supplementary Figure 3: Functional connectivity effects of naltrexone administration. Rats were administered naltrexone (10 mg/kg) followed by vehicle after 10 min to ensure infusion conditions consistent with the ketamine imaging experiments. Matrices are mean of *N* = 18 rats (9 females) at the. Three-way ANOVA with within-subjects factors of ROI pair and time (-15 min: pre-naltrexone; -5 min, 10 min, and 40 min) and between-subjects factor of sex: time, $F_{1.84,29.5}$ = 0.728, *P* = 0.259; ROI pair, $F_{230,3680}$ = 9.31, *P* < 0.001; sex, $F_{1,16}$ = 0.014, *P* = 0.909; ROI pair × time interaction, $F_{690,11040}$ = 1.16, *P* = 0.003. In the Tukey's post-hoc test, the only significant difference was in the GIDI-R - S1-R ROI pair between the -5-min and 40-min time points (*P* = 0.045). See **Supplementary Table 1** for a complete report of all the factors and post-hoc test.

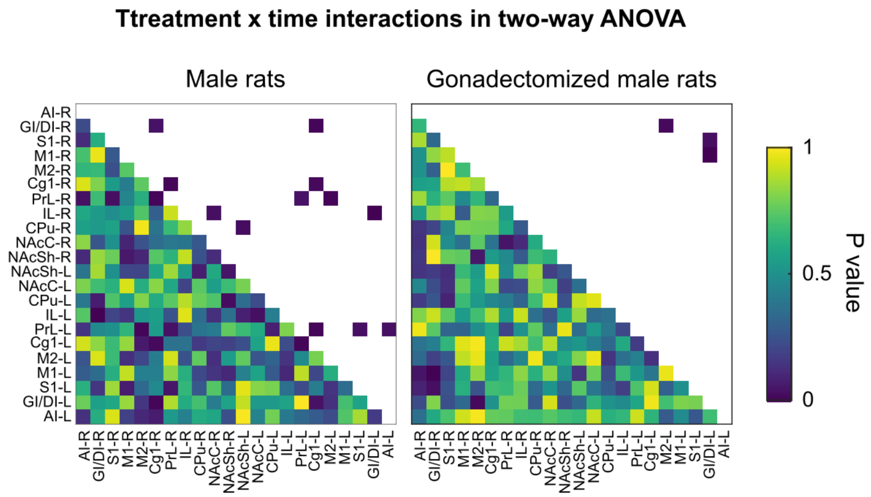

Supplementary Figure 4: Functional connectivity changes in gonadectomized male rats. *P* values from two-way ANOVA stratified by ROI pair with treatment and time within-subjects factors. Only the treatment × time interaction is reported in the plots. *N* = 7 gonadectomized male rats; *N* = 9 normal male rats.

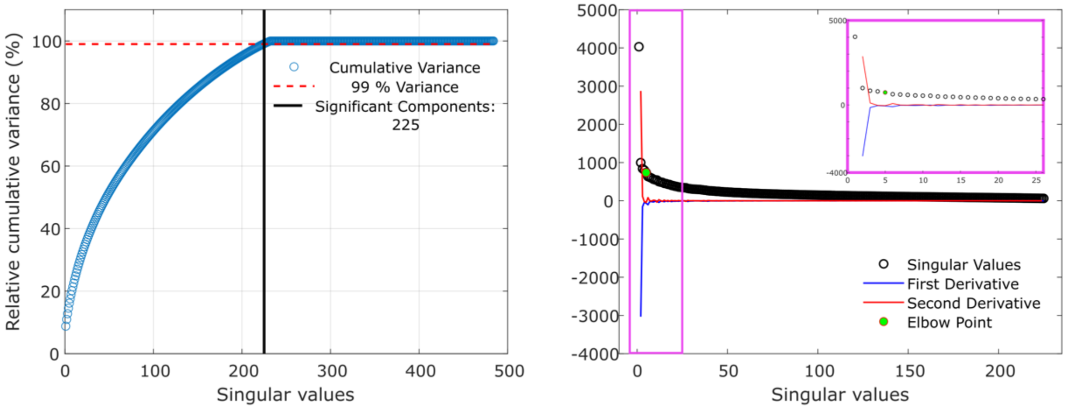

Supplementary Figure 5: Dynamic functional connectivity methods. To find the inflection point of the singular values, we applied the elbow method based on singular value decomposition. We first calculated the cumulative variance of the ranked singular values and identified the smallest number of singular values for which the cumulative variance was greater than or equal to 99% (left). Then, we computed the second derivative of the largest 225 singular values to identify the point where the rate of change of the singular values is minimum (elbow point) (right). This analysis determined that 5 clusters were the optimal number beyond which the marginal gain of adding another cluster was minimum.

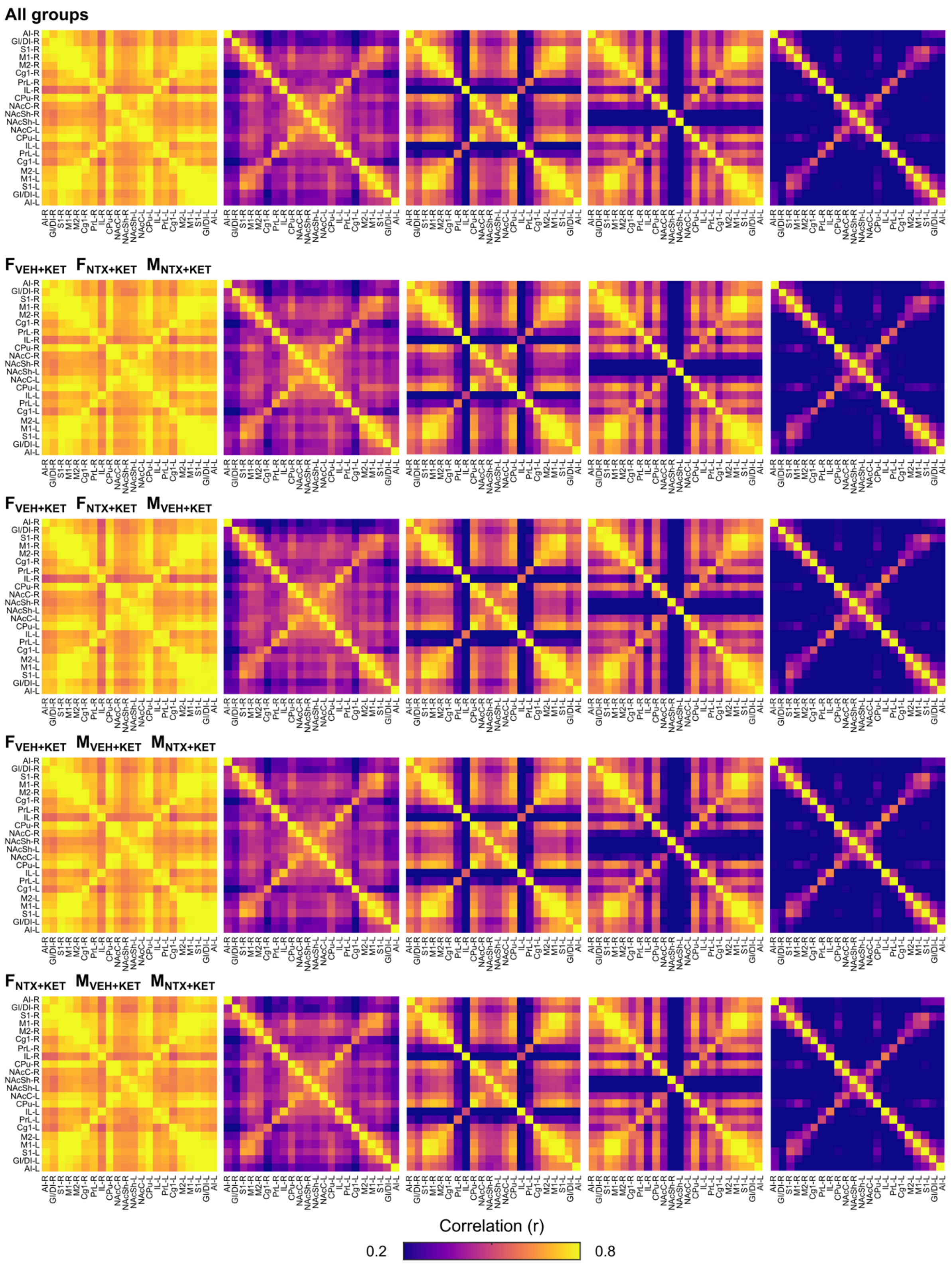

Supplementary Figure 6: Leave-one-group-out validation. To validate the k-means clustering approach, we performed a leave-one-group-out validation, where we repeated the clustering leaving out

one of the four conditions (males or females, NTX+KET or VEH+KET treatment; $N = 9$ rats per condition). This validation confirmed that k-means provides equivalent clustering results under all the different conditions.

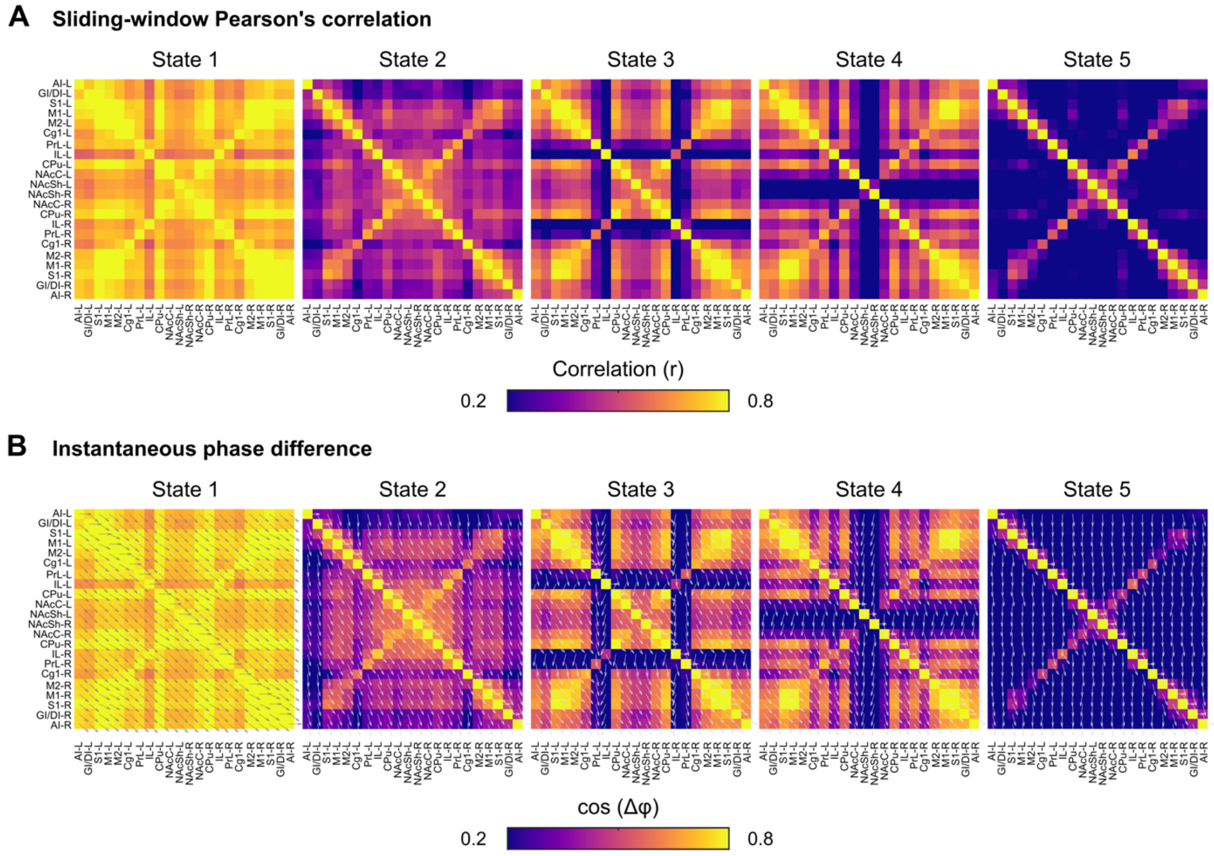

Supplementary Figure 7: Dynamic functional connectivity from instantaneous phase difference. We applied k-means clustering to a temporal sequence of connectivity matrices calculated using the cosine of the instantaneous phase difference between CBV signals in the different ROIs. This method does not require a sliding temporal window. The connectivity clusters/brain states using the instantaneous phase (**B**) show high similarity with the ones found with the 30-s sliding temporal window approach (**A**).

Supplementary Table 1: Results of the statistical analyses. This table contains the details of all the statistical comparisons reported in the manuscript text and figures.
