# Supplementary material for: Ketamine-induced static and dynamic functional connectivity changes are modulated by opioid receptors and biological sex in rats
Source: Neuropsychopharmacology. 2025 Apr 19;50(11):1695–705. doi: 10.1038/s41386-025-02108-0 (PMC12436656; doi:10.1038/s41386-025-02108-0)
Supplement: Supplementary file 3 — Supplementary Table 1 [file 41386_2025_2108_MOESM3_ESM.pdf]

Figure 1C. Functional connectivity matrix at baseline

Sample size: n = 18 rats, 9 females

Two-way mixed-effects ANOVA with within-subjects factor of ROI and between-subjects factor of sex

**Effect**	**DFn**	**DFd**	**F**	**p**	**p<.05**	**ges**
**sex**	1	16	0.357	0.558		0.003
**roi**	230	3680	11.037	5.67E-277	*	0.368
**sex:roi**	230	3680	1.044	0.315		0.052

Figure 1D. Baseline correlations of the left PrL and IL

Sample size: n = 18 rats, 9 females

**PrL**								
Two-tailed paired t-test with Benjamini–Hochberg correction								
**group1**	**group2**	**n1**	**n2**	**statistic**	**df**	**p**	**p.adj**	**p.adj.signif**
IL L	PrL R	18	18	-5.0374	17	0.000101	0.000119	***
IL R	PrL R	18	18	-4.9592	17	0.000119	0.000119	***
All left	PrL R	18	18	-7.5168	17	8.43E-07	1.69E-06	****
All right	PrL R	18	18	-9.1806	17	5.34E-08	2.14E-07	****

**IL**								
Two-tailed paired t-test with Benjamini–Hochberg correction								
**group1**	**group2**	**n1**	**n2**	**statistic**	**df**	**p**	**p.adj**	**p.adj.signif**
IL R	PrL L	18	18	5.16447	17	7.78E-05	7.78E-05	****
IL R	PrL R	18	18	8.02482	17	3.50E-07	4.67E-07	****
All left	IL R	18	18	-12.985	17	2.98E-10	1.19E-09	****
All right	IL R	18	18	-12.131	17	8.53E-10	1.71E-09	****

Figure 1E-F, Supplementary Fig. 2. Functional connectivity post ketamine administration

Sample size: n = 18 rats, 9 females

Three-way mixed-effects ANOVA with within-subjects factors of ROI and time and between-subjects factor of sex

Effect	DFn	DFd	F	p	p<.05	ges
**sex**	1	16	1.509	0.237		0.011
**time**	2	32	1.215	0.31		0.005
**roi**	230	3680	14.803	0	*	0.308
**sex:time**	2	32	1.504	0.238		0.006
**sex:roi**	230	3680	0.891	0.875		0.026
**time:roi**	460	7360	2.056	#######	*	0.042
**sex:time**	460	7360	1.005	0.463		0.021

Tukey's HSD post-hoc test

roi	term	time	time	estimate	conf.low	conf.higl	p.adj	p.adj.signif
IL R - IL L	time	1	2	-0.4389	-0.678	-0.1998	0.00015	***
PrL R - P	time	1	2	-0.5352	-0.8782	-0.1921	0.00123	**
PrL R - IL	time	1	2	-0.3385	-0.5993	-0.0777	0.00793	**
PrL R - P	time	2	3	0.42414	0.08111	0.76718	0.0119	*
GIDI R - I	time	1	2	-0.3519	-0.6438	-0.0599	0.0146	*
IL R - NA	time	2	3	-0.2561	-0.48	-0.0321	0.0215	*
IL R - IL L	time	2	3	0.26802	0.02893	0.50712	0.0246	*
Cg1 R - II	time	2	3	-0.3091	-0.587	-0.0312	0.026	*
Cg1 R - C	time	1	2	-0.4257	-0.8156	-0.0358	0.0294	*
AI R - NA	time	1	2	-0.2661	-0.5132	-0.019	0.0321	*
CPu L - A	time	1	2	-0.2928	-0.5665	-0.0192	0.0334	*
AI R - NA	time	1	2	-0.3033	-0.592	-0.0147	0.0374	*
Cg1 R - II	time	1	2	0.28968	0.0118	0.56755	0.0393	*
GIDI R - (	time	1	2	-0.308	-0.6086	-0.0073	0.0435	*
NAcC L -	time	1	2	-0.3042	-0.6026	-0.0057	0.0449	*
AI R - CP	time	1	2	-0.2752	-0.5455	-0.0049	0.0451	*
IL R - NA	time	1	2	0.23056	0.00402	0.4571	0.0452	*
IL R - NA	time	2	3	-0.2278	-0.4543	-0.0012	0.0485	*
GIDI R - I	time	1	2	-0.29	-0.5789	-0.0012	0.0489	*
IL R - Cg	time	2	3	-0.2772	-0.56	0.00549	0.0557	ns
M1 L - S1	time	2	3	0.27618	-0.0061	0.55844	0.0563	ns
CPu R - A	time	1	2	-0.2902	-0.5872	0.00677	0.0567	ns
M2 R - AI	time	1	2	-0.3048	-0.6174	0.00785	0.0575	ns
PrL R - IL	time	1	3	-0.2516	-0.5124	0.0092	0.0608	ns
GIDI R - (	time	1	2	-0.3113	-0.6456	0.02293	0.0727	ns
AI R - CP	time	1	2	-0.2447	-0.5126	0.0232	0.0799	ns
M1 R - M	time	1	2	-0.2685	-0.5642	0.02719	0.0822	ns
S1 R - Cg	time	1	3	-0.2195	-0.4642	0.02528	0.0872	ns
IL L - Cg1	time	2	3	-0.2292	-0.4875	0.02908	0.0914	ns
IL R - M1	time	1	2	0.21114	-0.0288	0.45106	0.0949	ns
M1 R - GI	time	1	2	-0.2806	-0.5994	0.0383	0.095	ns
PrL L - Cg	time	1	2	0.26298	-0.036	0.56194	0.0951	ns
M1 R - AI	time	1	2	-0.3101	-0.665	0.04489	0.0981	ns
M1 R - NA	time	2	3	-0.1922	-0.4142	0.02985	0.102	ns
Cg1 L - M	time	2	3	-0.2184	-0.4712	0.03446	0.103	ns
M2 R - M	time	1	2	-0.2322	-0.5022	0.03791	0.105	ns
GIDI R - I	time	1	2	-0.2559	-0.5538	0.04207	0.106	ns
Cg1 R - F	time	1	2	0.2914	-0.0492	0.632	0.107	ns
IL R - NA	time	2	3	-0.2356	-0.5132	0.04193	0.111	ns
S1 R - NA	time	1	3	-0.1988	-0.4359	0.03844	0.117	ns
M1 R - NA	time	1	3	-0.1847	-0.4067	0.0373	0.12	ns
NAcC L -	time	2	3	-0.1856	-0.4084	0.03723	0.12	ns
AI R - M2	time	1	2	-0.3014	-0.6684	0.06561	0.127	ns
IL R - PrL	time	1	2	-0.2417	-0.5365	0.05317	0.128	ns
IL R - NA	time	1	2	0.19845	-0.0449	0.44184	0.131	ns
S1 R - NA	time	2	3	-0.1932	-0.4304	0.04398	0.131	ns
S1 R - NA	time	2	3	-0.2124	-0.4746	0.04975	0.134	ns
IL R - M2	time	2	3	-0.2349	-0.5272	0.05739	0.138	ns
IL R - NA	time	1	2	0.17992	-0.044	0.40386	0.138	ns
IL R - NA	time	2	3	-0.1949	-0.4382	0.04854	0.14	ns
NAcC L -	time	1	3	-0.1784	-0.4013	0.0444	0.14	ns
IL L - PrL	time	1	2	-0.2021	-0.4553	0.05103	0.141	ns
CPu L - C	time	2	3	-0.2033	-0.4596	0.05295	0.145	ns
AI R - M2	time	2	3	0.28976	-0.0772	0.65674	0.147	ns
AI R - M1	time	1	2	-0.3019	-0.6852	0.0813	0.149	ns
AI R - IL I	time	2	3	0.19075	-0.0526	0.43415	0.151	ns
S1 L - AI	time	1	3	-0.2771	-0.6312	0.07699	0.152	ns
NAcC R -	time	1	2	0.2109	-0.0602	0.48204	0.156	ns
IL R - M1	time	2	3	-0.1858	-0.4257	0.05411	0.158	ns
M1 R - S1	time	1	2	-0.2621	-0.6003	0.07613	0.158	ns
M2 R - GI	time	1	2	-0.2196	-0.5053	0.06603	0.162	ns
NAcSh R	time	1	3	0.24682	-0.0756	0.56925	0.165	ns
GIDI R - I	time	1	2	-0.2262	-0.5224	0.06994	0.166	ns
NAcC L -	time	2	3	-0.1951	-0.4511	0.06098	0.167	ns
GIDI R - (	time	1	2	-0.1945	-0.4504	0.06134	0.168	ns
IL R - S1	time	1	2	0.21529	-0.0679	0.49851	0.169	ns

Hedge's *g* effect size

effsize	n1	n2	magnitude
1.44813	18	18	large
0.91916	18	18	large
0.77192	18	18	moderate
-0.7515	18	18	moderate
0.71934	18	18	moderate
1.00386	18	18	large
-0.635	18	18	moderate
0.94692	18	18	large
0.6394	18	18	moderate
0.52991	18	18	moderate
0.66219	18	18	moderate
0.69353	18	18	moderate
-0.7889	18	18	moderate
0.69571	18	18	moderate
0.55928	18	18	moderate
0.48912	18	18	small
-0.7755	18	18	moderate
0.78797	18	18	moderate
0.59704	18	18	moderate
0.76264	18	18	moderate
-0.5645	18	18	moderate
0.82712	18	18	large
0.6324	18	18	moderate
0.78919	18	18	moderate
0.59967	18	18	moderate
0.41297	18	18	small
0.55372	18	18	moderate
0.76057	18	18	moderate
0.61927	18	18	moderate
-0.6086	18	18	moderate
0.46052	18	18	small
-0.474	18	18	small
0.59231	18	18	moderate
0.52223	18	18	moderate
0.72487	18	18	moderate
0.47627	18	18	small
0.48223	18	18	small
-0.7062	18	18	moderate
0.64302	18	18	moderate
0.62387	18	18	moderate
0.59488	18	18	moderate
0.53187	18	18	moderate
0.47718	18	18	small
0.41287	18	18	small
-0.8551	18	18	large
0.60565	18	18	moderate
0.69084	18	18	moderate
0.52404	18	18	moderate
-0.5276	18	18	moderate
0.77261	18	18	moderate
0.6744	18	18	moderate
0.39941	18	18	small
0.61316	18	18	moderate
-0.6235	18	18	moderate
0.50366	18	18	moderate
-0.4928	18	18	small
0.92822	18	18	large
-0.5588	18	18	moderate
0.51673	18	18	moderate
0.43289	18	18	small
0.45348	18	18	small
-0.4829	18	18	small
0.4677	18	18	small
0.46282	18	18	small
0.55393	18	18	moderate
-0.5111	18	18	moderate

GIDI R - I	time	1	3	-0.2186	-0.5075	0.07023	0.171	ns	0.81767	18	18	large
AI R - M1	time	2	3	0.28941	-0.0938	0.67264	0.172	ns	-0.5744	18	18	moderate
IL R - M2	time	1	2	0.22085	-0.0715	0.51316	0.172	ns	-0.5701	18	18	moderate
M2 L - M	time	2	3	-0.2079	-0.4831	0.06744	0.172	ns	0.5866	18	18	moderate
S1 R - Cg	time	2	3	-0.1835	-0.4283	0.06124	0.177	ns	0.69106	18	18	moderate
GIDI R - (	time	1	3	-0.2244	-0.5251	0.07622	0.179	ns	0.73242	18	18	moderate
IL L - M2	time	2	3	-0.1945	-0.4572	0.06832	0.184	ns	0.43249	18	18	small
S1 R - NA	time	2	3	-0.1751	-0.4126	0.06242	0.187	ns	0.47866	18	18	small
CPu L - N	time	2	3	-0.1758	-0.4171	0.0656	0.194	ns	0.44448	18	18	small
M2 R - NA	time	1	3	-0.1626	-0.3863	0.06112	0.195	ns	0.61242	18	18	moderate
AI R - NA	time	1	2	-0.2181	-0.5198	0.08349	0.198	ns	0.39597	18	18	small
IL R - IL L	time	1	3	-0.1709	-0.41	0.06821	0.206	ns	0.44173	18	18	small
CPu L - A	time	1	3	-0.1944	-0.4681	0.07922	0.209	ns	0.5587	18	18	moderate
S1 R - NA	time	1	3	-0.1731	-0.4167	0.07058	0.21	ns	0.48837	18	18	small
AI R - NA	time	2	3	0.20454	-0.0841	0.49322	0.211	ns	-0.3796	18	18	small
IL R - Cg	time	1	2	0.20019	-0.0825	0.48292	0.212	ns	-0.5565	18	18	moderate
IL L - Cg1	time	1	2	0.18041	-0.0779	0.4387	0.22	ns	-0.4372	18	18	small
Cg1 R - N	time	1	2	0.17283	-0.0759	0.42155	0.224	ns	-0.4752	18	18	small
S1 R - IL	time	1	2	0.19085	-0.0838	0.46547	0.224	ns	-0.6604	18	18	moderate
NAcC R -	time	1	2	-0.2111	-0.5166	0.09436	0.227	ns	0.46109	18	18	small
NAcSh L	time	1	2	0.1907	-0.0879	0.46934	0.234	ns	-0.4681	18	18	small
M2 R - S	time	1	2	-0.2109	-0.5198	0.09804	0.235	ns	0.34971	18	18	small
IL L - PrL	time	1	3	-0.1725	-0.4257	0.08065	0.236	ns	0.43024	18	18	small
M1 L - S1	time	1	2	-0.1924	-0.4747	0.08985	0.236	ns	0.43954	18	18	small
PrL R - IL	time	1	3	-0.2109	-0.5218	0.09997	0.239	ns	0.49441	18	18	small
NAcSh R	time	1	2	-0.2595	-0.6429	0.12392	0.241	ns	0.44984	18	18	small
M1 R - Cl	time	1	3	-0.1745	-0.4327	0.08361	0.242	ns	0.6409	18	18	moderate
S1 R - M	time	1	2	-0.2128	-0.5277	0.10206	0.242	ns	0.44113	18	18	small
IL R - NA	time	1	2	0.18471	-0.0929	0.46228	0.252	ns	-0.5787	18	18	moderate
M2 R - M	time	1	2	-0.2007	-0.5037	0.10227	0.255	ns	0.38215	18	18	small
IL R - GIE	time	1	2	0.14432	-0.0744	0.36309	0.258	ns	-0.4261	18	18	small
NAcC R -	time	2	3	-0.1621	-0.4087	0.0845	0.261	ns	0.53417	18	18	moderate
GIDI R - I	time	1	2	-0.1789	-0.4531	0.09528	0.266	ns	0.46627	18	18	small
M2 R - NA	time	2	3	-0.1456	-0.3694	0.07807	0.267	ns	0.48435	18	18	small
GIDI R - I	time	2	3	0.15207	-0.0822	0.38636	0.269	ns	-0.4759	18	18	small
M2 R - AI	time	2	3	0.20283	-0.1098	0.51545	0.269	ns	-0.5081	18	18	moderate
AI R - NA	time	1	3	-0.1584	-0.4055	0.08877	0.278	ns	0.75908	18	18	moderate
NAcSh R	time	1	2	0.20282	-0.1196	0.52524	0.291	ns	-0.5019	18	18	moderate
GIDI R - I	time	2	3	0.18562	-0.1105	0.48178	0.293	ns	-0.406	18	18	small
NAcSh L	time	2	3	-0.1474	-0.3824	0.08766	0.293	ns	0.4909	18	18	small
S1 R - M	time	1	2	-0.2055	-0.5338	0.12275	0.294	ns	0.38576	18	18	small
S1 R - M	time	2	3	0.19704	-0.1178	0.51193	0.295	ns	-0.3926	18	18	small
GIDI R - I	time	1	2	-0.1459	-0.3802	0.08837	0.298	ns	0.36642	18	18	small
NAcSh L	time	2	3	-0.1389	-0.3621	0.08431	0.298	ns	0.36274	18	18	small
IL R - S1	time	2	3	-0.176	-0.4592	0.10725	0.3	ns	0.44827	18	18	small
NAcSh L	time	1	2	-0.2189	-0.5716	0.13375	0.3	ns	0.42854	18	18	small
GIDI R - I	time	1	2	-0.2063	-0.5416	0.12896	0.306	ns	0.31216	18	18	small
M1 R - NA	time	1	2	-0.1813	-0.4761	0.11351	0.307	ns	0.37973	18	18	small
NAcC R -	time	2	3	-0.1819	-0.4782	0.11429	0.308	ns	0.38275	18	18	small
CPu L - C	time	1	3	-0.1775	-0.4688	0.1138	0.313	ns	0.7055	18	18	moderate
NAcC L -	time	1	3	-0.1556	-0.4117	0.1004	0.315	ns	0.52867	18	18	moderate
GIDI R - I	time	1	3	-0.1771	-0.4691	0.11481	0.316	ns	0.5952	18	18	moderate
Cg1 R - C	time	1	3	-0.2355	-0.6254	0.15438	0.319	ns	0.38804	18	18	small
GIDI R - I	time	2	3	0.17471	-0.1172	0.46666	0.326	ns	-0.3375	18	18	small
M1 R - NA	time	1	3	-0.1762	-0.471	0.1186	0.327	ns	0.45786	18	18	small
S1 R - CF	time	2	3	-0.1682	-0.4497	0.11324	0.327	ns	0.61749	18	18	moderate
NAcSh L	time	1	3	-0.1329	-0.3561	0.09026	0.329	ns	0.42542	18	18	small
M2 R - Pr	time	1	2	-0.153	-0.4105	0.10442	0.331	ns	0.34818	18	18	small
M2 R - Cg	time	1	3	-0.1592	-0.428	0.10954	0.333	ns	0.47026	18	18	small
M1 R - NA	time	2	3	-0.1365	-0.368	0.09505	0.337	ns	0.41263	18	18	small
AI R - Cg	time	1	2	-0.1603	-0.434	0.11339	0.341	ns	0.32726	18	18	small
M1 R - NA	time	1	3	-0.1262	-0.3416	0.08917	0.341	ns	0.36981	18	18	small
GIDI R - (	time	2	3	0.19502	-0.1393	0.52928	0.344	ns	-0.4475	18	18	small
NAcC R -	time	2	3	-0.1567	-0.4278	0.11445	0.351	ns	0.41862	18	18	small
PrL L - Cg	time	2	3	-0.172	-0.4709	0.12701	0.354	ns	0.34898	18	18	small
Cg1 R - F	time	2	3	-0.1957	-0.5363	0.14493	0.355	ns	0.45833	18	18	small
NAcSh L	time	1	3	-0.1433	-0.395	0.10841	0.362	ns	0.49144	18	18	small
CPu R - (	time	1	2	-0.1705	-0.4704	0.12935	0.363	ns	0.38121	18	18	small
M2 R - Cl	time	1	3	-0.1506	-0.4156	0.11433	0.363	ns	0.57527	18	18	moderate
Cg1 R - N	time	2	3	-0.1423	-0.3938	0.10911	0.366	ns	0.34347	18	18	small
M1 R - Gl	time	1	3	-0.1801	-0.499	0.13876	0.367	ns	0.51233	18	18	moderate
NAcC L -	time	1	3	-0.1686	-0.467	0.12983	0.367	ns	0.56333	18	18	moderate
AI R - Cg	time	1	2	-0.1233	-0.3431	0.0965	0.372	ns	0.34389	18	18	small
IL R - CP	time	1	2	0.12625	-0.0992	0.35167	0.374	ns	-0.3642	18	18	small
NAcSh R	time	1	3	-0.1566	-0.4372	0.12394	0.376	ns	0.47889	18	18	small
S1 L - GII	time	1	2	-0.1857	-0.5181	0.14675	0.376	ns	0.34425	18	18	small
S1 L - AI	time	1	2	-0.1974	-0.5515	0.1567	0.377	ns	0.37862	18	18	small
GIDI R - I	time	1	2	-0.16	-0.4473	0.12734	0.378	ns	0.35317	18	18	small
GIDI R - (	time	1	2	-0.1356	-0.3796	0.10832	0.379	ns	0.51373	18	18	moderate
AI R - M1	time	1	2	-0.1729	-0.4844	0.13853	0.38	ns	0.42073	18	18	small
M1 R - AI	time	2	3	0.19613	-0.1588	0.5511	0.383	ns	-0.4537	18	18	small
NAcC L -	time	2	3	-0.138	-0.3896	0.11356	0.388	ns	0.36186	18	18	small
S1 R - M	time	2	3	0.17965	-0.1486	0.50794	0.39	ns	-0.4014	18	18	small
S1 R - NA	time	1	3	-0.13	-0.3675	0.10745	0.39	ns	0.47073	18	18	small
IL R - PrL	time	1	3	-0.1613	-0.4561	0.13357	0.391	ns	0.35363	18	18	small
NAcSh L	time	2	3	-0.137	-0.3887	0.11468	0.394	ns	0.31111	18	18	small

S1 R - CP	time	1	3	-0.153	-0.4345	0.12843	0.395	ns	0.54522	18	18	moderate
AI R - CP	time	1	3	-0.145	-0.4129	0.12294	0.398	ns	0.4822	18	18	small
GIDI R - I	time	1	2	-0.1295	-0.3687	0.10974	0.398	ns	0.29993	18	18	small
S1 R - Cg	time	1	3	-0.1291	-0.3679	0.10966	0.399	ns	0.3524	18	18	small
IL R - GID	time	2	3	-0.1179	-0.3366	0.10089	0.401	ns	0.37545	18	18	small
IL L - AI L	time	2	3	0.11836	-0.1025	0.33918	0.405	ns	-0.3411	18	18	small
AI R - CP	time	1	3	-0.1446	-0.4149	0.12567	0.406	ns	0.46251	18	18	small
IL L - M2	time	1	2	0.14014	-0.1226	0.40292	0.409	ns	-0.327	18	18	small
NAcC L -	time	2	3	-0.1474	-0.4242	0.12945	0.41	ns	0.31687	18	18	small
M2 R - S1	time	2	3	0.16185	-0.1471	0.47078	0.421	ns	-0.3395	18	18	small
NAcC R -	time	1	2	0.13796	-0.1254	0.40136	0.422	ns	-0.292	18	18	small
CPu R - S	time	1	3	-0.1483	-0.4323	0.13556	0.423	ns	0.3694	18	18	small
GIDI R - (	time	1	3	-0.1327	-0.3886	0.12315	0.429	ns	0.53627	18	18	moderate
M1 R - NA	time	2	3	-0.1117	-0.3271	0.10375	0.429	ns	0.25765	18	18	small
S1 R - Cg	time	2	3	-0.1224	-0.3612	0.11635	0.437	ns	0.438	18	18	small
CPu R - A	time	2	3	0.1519	-0.1451	0.44886	0.438	ns	-0.3394	18	18	small
PrL L - AI	time	1	2	-0.1437	-0.4251	0.13767	0.44	ns	0.30222	18	18	small
S1 R - IL	time	2	3	-0.1396	-0.4143	0.13498	0.443	ns	0.47733	18	18	small
GIDI R - I	time	1	3	-0.1455	-0.4328	0.14181	0.446	ns	0.42167	18	18	small
GIDI L - A	time	1	3	-0.199	-0.5929	0.19479	0.447	ns	0.654	18	18	moderate
NAcSh L	time	1	2	0.12407	-0.1215	0.36963	0.447	ns	-0.3333	18	18	small
AI R - NA	time	2	3	0.15145	-0.1502	0.45307	0.452	ns	-0.279	18	18	small
Cg1 L - M	time	1	2	0.12654	-0.1263	0.37937	0.454	ns	-0.4193	18	18	small
GIDI R - I	time	1	2	-0.123	-0.3691	0.1231	0.455	ns	0.39665	18	18	small
GIDI R - I	time	2	3	0.16659	-0.1687	0.50184	0.459	ns	-0.3734	18	18	small
Cg1 L - S	time	2	3	-0.1267	-0.3825	0.12904	0.461	ns	0.30818	18	18	small
Cg1 R - N	time	2	3	-0.1151	-0.3478	0.11763	0.462	ns	0.29265	18	18	small
GIDI R - I	time	1	3	-0.147	-0.4449	0.15092	0.464	ns	0.54354	18	18	moderate
AI R - GID	time	1	2	-0.1379	-0.4184	0.14256	0.466	ns	0.26436	18	18	small
NAcSh L	time	1	2	0.15426	-0.1594	0.46796	0.466	ns	-0.3461	18	18	small
CPu R - (	time	1	3	-0.1473	-0.4472	0.15253	0.467	ns	0.51188	18	18	moderate
GIDI R - I	time	1	3	-0.1342	-0.4084	0.13996	0.469	ns	0.58163	18	18	moderate
Cg1 R - C	time	2	3	0.19017	-0.1997	0.58009	0.472	ns	-0.34	18	18	small
PrL R - N	time	2	3	-0.1242	-0.3809	0.13245	0.477	ns	0.29408	18	18	small
AI R - CP	time	2	3	0.13059	-0.1397	0.40088	0.479	ns	-0.2828	18	18	small
PrL R - M	time	1	2	0.13574	-0.1456	0.4171	0.48	ns	-0.3871	18	18	small
CPu L - M	time	1	3	-0.1161	-0.3575	0.12524	0.481	ns	0.41064	18	18	small
CPu L - II	time	2	3	0.09265	-0.1003	0.28559	0.483	ns	-0.3625	18	18	small
CPu R - N	time	1	3	-0.1295	-0.3993	0.14026	0.483	ns	0.40715	18	18	small
CPu L - C	time	1	2	-0.1392	-0.4305	0.15205	0.486	ns	0.28863	18	18	small
GIDI R - (	time	1	2	-0.1292	-0.3994	0.14098	0.486	ns	0.32645	18	18	small
M1 L - AI	time	1	2	-0.151	-0.4668	0.16482	0.486	ns	0.36	18	18	small
PrL R - S	time	1	2	0.11133	-0.122	0.34468	0.487	ns	-0.317	18	18	small
M2 R - M	time	2	3	0.12845	-0.1416	0.39851	0.489	ns	-0.3211	18	18	small
M2 L - M1	time	1	2	0.12877	-0.1465	0.40406	0.501	ns	-0.2713	18	18	small
IL L - M1	time	1	2	0.09599	-0.1099	0.3019	0.503	ns	-0.3013	18	18	small
AI R - M2	time	1	2	-0.1557	-0.4904	0.17895	0.504	ns	0.30525	18	18	small
CPu R - A	time	1	3	-0.1383	-0.4353	0.15866	0.504	ns	0.39384	18	18	small
NAcSh R	time	2	3	0.17863	-0.2048	0.56206	0.504	ns	-0.3138	18	18	small
NAcC R -	time	1	2	0.12597	-0.1449	0.39686	0.505	ns	-0.3032	18	18	small
S1 R - NA	time	1	3	-0.1214	-0.3836	0.14076	0.508	ns	0.36013	18	18	small
PrL R - N	time	1	2	0.11153	-0.1299	0.35296	0.509	ns	-0.2986	18	18	small
M2 R - GI	time	1	3	-0.1318	-0.4174	0.15388	0.51	ns	0.44812	18	18	small
S1 L - GII	time	1	3	-0.1532	-0.4856	0.17925	0.511	ns	0.52719	18	18	moderate
M2 R - M	time	1	3	-0.1387	-0.4417	0.16432	0.515	ns	0.40505	18	18	small
M2 R - NA	time	1	3	-0.104	-0.331	0.12307	0.515	ns	0.35932	18	18	small
M1 R - M	time	2	3	0.13506	-0.1606	0.43077	0.517	ns	-0.3269	18	18	small
NAcC R -	time	2	3	-0.1232	-0.3928	0.14641	0.517	ns	0.31779	18	18	small
PrL R - G	time	1	2	0.10077	-0.1199	0.3214	0.517	ns	-0.2632	18	18	small
Cg1 R - II	time	2	3	-0.109	-0.3481	0.12999	0.518	ns	0.38815	18	18	small
NAcC L -	time	2	3	0.13556	-0.1629	0.43399	0.521	ns	-0.2801	18	18	small
NAcSh R	time	1	2	0.14147	-0.1708	0.45378	0.522	ns	-0.3547	18	18	small
S1 R - NA	time	1	2	-0.1105	-0.3541	0.13315	0.522	ns	0.27566	18	18	small
M1 R - M	time	1	3	-0.1335	-0.4292	0.16225	0.525	ns	0.43127	18	18	small
M1 R - M	time	1	3	-0.1191	-0.3835	0.14528	0.526	ns	0.32135	18	18	small
NAcSh L	time	2	3	-0.125	-0.4036	0.15367	0.529	ns	0.30231	18	18	small
M2 R - NA	time	1	2	-0.14	-0.4544	0.17446	0.534	ns	0.34396	18	18	small
CPu L - S	time	1	3	-0.1404	-0.4577	0.17694	0.538	ns	0.36511	18	18	small
NAcSh L	time	1	3	-0.1039	-0.339	0.13109	0.538	ns	0.32857	18	18	small
CPu L - II	time	1	2	-0.0851	-0.2781	0.1078	0.54	ns	0.25297	18	18	small
IL L - M1	time	2	3	-0.0907	-0.2966	0.11523	0.541	ns	0.29909	18	18	small
AI R - NA	time	2	3	0.10773	-0.1394	0.35487	0.548	ns	-0.2073	18	18	small
AI R - AI	time	1	3	-0.1738	-0.5735	0.22596	0.55	ns	0.60183	18	18	moderate
Cg1 R - C	time	1	2	0.12299	-0.1606	0.40662	0.551	ns	-0.3136	18	18	small
M1 R - M	time	1	2	-0.1797	-0.5979	0.23851	0.557	ns	0.32355	18	18	small
M2 R - Pr	time	2	3	0.11028	-0.1472	0.36772	0.559	ns	-0.2848	18	18	small
NAcC R -	time	1	2	0.12622	-0.17	0.42244	0.562	ns	-0.2392	18	18	small
PrL R - IL	time	2	3	-0.1326	-0.4435	0.1783	0.562	ns	0.3281	18	18	small
NAcSh R	time	1	3	-0.1162	-0.3902	0.15785	0.566	ns	0.31977	18	18	small
S1 R - AI	time	1	2	-0.1324	-0.4459	0.18109	0.568	ns	0.31383	18	18	small
CPu L - C	time	1	2	0.10815	-0.1481	0.36445	0.569	ns	-0.2869	18	18	small
GIDI R - I	time	1	3	-0.102	-0.3481	0.14409	0.58	ns	0.42394	18	18	small
GIDI R - (	time	1	3	-0.1117	-0.3818	0.15853	0.582	ns	0.39722	18	18	small
AI R - IL I	time	1	2	-0.1004	-0.3438	0.143	0.583	ns	0.25163	18	18	small
AI R - GID	time	1	3	-0.1154	-0.3959	0.16505	0.584	ns	0.27382	18	18	small
M1 R - IL	time	1	2	0.12258	-0.1758	0.42096	0.585	ns	-0.3675	18	18	small

NAcC R -	time	1	2	0.10051	-0.1461	0.34712	0.59	ns	-0.2332	18	18	small
CPu R - (	time	2	3	-0.1066	-0.3684	0.15529	0.591	ns	0.24628	18	18	small
M1 R - C(	time	2	3	-0.1004	-0.3467	0.146	0.591	ns	0.29371	18	18	small
M2 L - AI	time	1	2	-0.1262	-0.4371	0.18474	0.593	ns	0.26697	18	18	small
AI R - NA	time	1	2	-0.1176	-0.4085	0.17334	0.595	ns	0.25579	18	18	small
Cg1 R - N	time	1	3	0.1006	-0.1481	0.34932	0.595	ns	-0.1913	18	18	negligible
S1 R - Pr	time	1	2	0.08096	-0.1205	0.28243	0.599	ns	-0.2979	18	18	small
Cg1 L - M	time	1	2	0.13243	-0.1977	0.46257	0.6	ns	-0.2274	18	18	small
CPu R - N	time	1	2	-0.1359	-0.4754	0.20356	0.601	ns	0.33341	18	18	small
Cg1 R - C	time	2	3	-0.1021	-0.3576	0.15339	0.602	ns	0.24444	18	18	small
M1 R - Cl	time	1	2	-0.1025	-0.3607	0.15559	0.606	ns	0.27877	18	18	small
IL R - CP	time	1	3	0.08938	-0.136	0.3148	0.607	ns	-0.2161	18	18	small
Cg1 R - C	time	1	3	-0.1011	-0.3566	0.15438	0.608	ns	0.25367	18	18	small
S1 R - M:	time	1	2	-0.108	-0.3822	0.1662	0.611	ns	0.2121	18	18	small
IL L - AI L	time	1	2	-0.0868	-0.3076	0.13405	0.612	ns	0.24107	18	18	small
NAcC R -	time	2	3	0.1197	-0.1858	0.42519	0.614	ns	-0.2694	18	18	small
AI R - S1	time	1	2	-0.1158	-0.4122	0.1806	0.616	ns	0.24329	18	18	small
NAcC L -	time	1	2	0.10807	-0.1688	0.3849	0.616	ns	-0.2145	18	18	small
M1 R - S:	time	2	3	0.13141	-0.2068	0.46962	0.619	ns	-0.2609	18	18	small
M1 R - S:	time	1	3	-0.1307	-0.4689	0.20753	0.622	ns	0.23889	18	18	small
NAcSh L	time	2	3	-0.0949	-0.3405	0.15061	0.622	ns	0.20851	18	18	small
NAcC L -	time	1	2	-0.0966	-0.3469	0.15363	0.623	ns	0.27063	18	18	small
M1 R - Pr	time	1	3	0.10221	-0.1641	0.36856	0.626	ns	-0.342	18	18	small
M2 R - M	time	1	3	-0.1037	-0.3738	0.16636	0.626	ns	0.28967	18	18	small
Cg1 R - N	time	1	2	0.09641	-0.155	0.34786	0.627	ns	-0.2248	18	18	small
M1 R - Cl	time	1	3	-0.106	-0.3836	0.17159	0.629	ns	0.32586	18	18	small
PrL R - G	time	2	3	-0.0838	-0.3044	0.13684	0.632	ns	0.23994	18	18	small
Cg1 L - M	time	2	3	-0.1251	-0.4553	0.20499	0.633	ns	0.19442	18	18	negligible
GIDI L - A	time	1	2	-0.1491	-0.5429	0.2447	0.634	ns	0.31403	18	18	small
GIDI R - A	time	1	2	-0.1139	-0.4151	0.18722	0.634	ns	0.2894	18	18	small
M2 R - Cl	time	1	2	-0.099	-0.3608	0.16269	0.634	ns	0.29827	18	18	small
NAcSh R	time	1	2	-0.1055	-0.3861	0.17508	0.638	ns	0.26119	18	18	small
Cg1 R - N	time	1	3	0.09547	-0.1587	0.34962	0.639	ns	-0.2202	18	18	small
NAcSh L	time	2	3	-0.1176	-0.4313	0.19613	0.64	ns	0.26041	18	18	small
M1 R - NA	time	1	3	-0.0865	-0.318	0.14508	0.642	ns	0.30082	18	18	small
AI R - CP	time	2	3	0.09974	-0.1682	0.36765	0.644	ns	-0.2003	18	18	small
AI R - IL L	time	1	3	0.09036	-0.153	0.33375	0.645	ns	-0.3261	18	18	small
Cg1 R - S	time	2	3	-0.0898	-0.3317	0.15207	0.645	ns	0.25264	18	18	small
CPu L - C	time	1	3	-0.0952	-0.3515	0.1611	0.645	ns	0.27996	18	18	small
M1 L - GI	time	1	2	-0.1201	-0.4437	0.20345	0.645	ns	0.27958	18	18	small
NAcC L -	time	1	3	-0.0925	-0.3428	0.15775	0.648	ns	0.35397	18	18	small
NAcC L -	time	1	2	0.09255	-0.159	0.34414	0.65	ns	-0.2661	18	18	small
S1 R - M:	time	1	3	-0.1087	-0.4049	0.18748	0.651	ns	0.35398	18	18	small
PrL R - M	time	1	2	0.08399	-0.1456	0.31357	0.653	ns	-0.2073	18	18	small
GIDI R - I	time	2	3	0.10885	-0.1891	0.40678	0.654	ns	-0.2454	18	18	small
CPu R - N	time	2	3	0.12358	-0.2159	0.46308	0.656	ns	-0.2607	18	18	small
Cg1 L - M	time	1	3	-0.0918	-0.3447	0.16099	0.657	ns	0.30485	18	18	small
AI R - M2	time	1	3	-0.1212	-0.4559	0.21343	0.659	ns	0.43874	18	18	small
M2 R - NA	time	1	3	-0.1139	-0.4283	0.20052	0.659	ns	0.29186	18	18	small
M1 R - C(	time	1	3	-0.0889	-0.3352	0.1575	0.661	ns	0.26936	18	18	small
CPu L - A	time	2	3	0.09839	-0.1753	0.37205	0.663	ns	-0.2384	18	18	small
NAcSh L	time	2	3	0.12616	-0.2265	0.47881	0.665	ns	-0.226	18	18	small
S1 R - M:	time	2	3	0.09806	-0.1761	0.37227	0.666	ns	-0.199	18	18	negligible
CPu L - S	time	2	3	-0.1128	-0.4301	0.20454	0.669	ns	0.36924	18	18	small
Cg1 R - C	time	2	3	-0.1002	-0.3839	0.1834	0.672	ns	0.25739	18	18	small
AI R - Cg	time	1	3	-0.0964	-0.3701	0.17729	0.674	ns	0.39709	18	18	small
CPu L - N	time	2	3	-0.1119	-0.4311	0.20735	0.676	ns	0.23364	18	18	small
NAcSh R	time	1	2	-0.0927	-0.358	0.17267	0.678	ns	0.24929	18	18	small
AI R - PrL	time	1	2	-0.0817	-0.3163	0.15296	0.68	ns	0.1693	18	18	negligible
CPu R - S	time	1	2	-0.0988	-0.3827	0.1851	0.68	ns	0.17388	18	18	negligible
GIDI R - (	time	1	3	-0.1163	-0.4506	0.21795	0.68	ns	0.39401	18	18	small
M2 R - NA	time	2	3	-0.0788	-0.3059	0.14824	0.681	ns	0.20935	18	18	small
S1 R - NA	time	1	2	0.09101	-0.1712	0.3532	0.681	ns	-0.1773	18	18	negligible
PrL L - M	time	1	2	0.09403	-0.1775	0.36554	0.683	ns	-0.1833	18	18	negligible
NAcC L -	time	1	2	0.09921	-0.1904	0.38882	0.688	ns	-0.2031	18	18	small
AI R - NA	time	1	3	-0.0988	-0.3875	0.18988	0.689	ns	0.32483	18	18	small
IL R - NA	time	1	3	-0.0761	-0.3001	0.1478	0.692	ns	0.1974	18	18	negligible
M2 R - Cl	time	2	3	-0.0897	-0.3546	0.17527	0.694	ns	0.2659	18	18	small
GIDI R - I	time	2	3	0.09336	-0.1837	0.37045	0.697	ns	-0.2578	18	18	small
NAcSh R	time	2	3	0.08932	-0.176	0.35466	0.697	ns	-0.1969	18	18	negligible
PrL L - AI	time	1	3	-0.0946	-0.3759	0.18681	0.698	ns	0.32397	18	18	small
AI R - Cg	time	1	3	-0.0737	-0.2935	0.14609	0.699	ns	0.25556	18	18	small
S1 R - Pr	time	2	3	-0.0676	-0.269	0.13391	0.699	ns	0.28167	18	18	small
GIDI R - A	time	1	3	-0.1007	-0.4019	0.20042	0.7	ns	0.34175	18	18	small
CPu R - I	time	1	2	-0.0786	-0.3141	0.15685	0.701	ns	0.23378	18	18	small
PrL R - IL	time	2	3	0.08691	-0.1739	0.34768	0.702	ns	-0.1884	18	18	negligible
M2 R - C(	time	1	2	-0.0889	-0.3576	0.17986	0.706	ns	0.20251	18	18	small
AI R - M1	time	2	3	0.10271	-0.2087	0.41415	0.707	ns	-0.2105	18	18	small
Cg1 R - F	time	2	3	0.10571	-0.2145	0.4259	0.707	ns	-0.2169	18	18	small
M1 R - Cl	time	1	2	-0.0916	-0.3692	0.18598	0.707	ns	0.2611	18	18	small
M2 R - AI	time	1	3	-0.1019	-0.4146	0.21069	0.713	ns	0.30461	18	18	small
PrL R - P	time	1	3	-0.111	-0.454	0.23202	0.716	ns	0.23058	18	18	small
S1 R - CF	time	1	2	-0.0791	-0.3239	0.16571	0.717	ns	0.17708	18	18	negligible
AI R - PrL	time	2	3	0.07566	-0.159	0.3103	0.718	ns	-0.2094	18	18	small
S1 R - M:	time	2	3	-0.0953	-0.3915	0.20088	0.719	ns	0.21656	18	18	small
M1 R - AI	time	1	3	-0.114	-0.4689	0.24102	0.72	ns	0.38916	18	18	small

Cg1 R - C	time	2	3	0.08613	-0.1855	0.35778	0.726	ns	-0.1589	18	18	negligible
S1 R - AI	time	1	3	-0.0989	-0.4124	0.2146	0.728	ns	0.32016	18	18	small
M1 R - GI	time	2	3	0.10046	-0.2184	0.41933	0.729	ns	-0.2239	18	18	small
M1 R - IL	time	2	3	-0.0939	-0.3923	0.20447	0.729	ns	0.28405	18	18	small
IL R - AI I	time	1	2	0.07029	-0.1534	0.29396	0.73	ns	-0.1804	18	18	negligible
M2 R - CI	time	2	3	0.08186	-0.1799	0.3436	0.732	ns	-0.2225	18	18	small
PrL R - N	time	1	3	0.07515	-0.1663	0.31658	0.734	ns	-0.2164	18	18	small
S1 R - GI	time	1	2	-0.0917	-0.386	0.20269	0.734	ns	0.1851	18	18	negligible
M2 L - AI	time	2	3	0.09566	-0.2153	0.40659	0.739	ns	-0.2058	18	18	small
M2 R - GI	time	2	3	0.08785	-0.1978	0.37351	0.74	ns	-0.2448	18	18	small
PrL L - C	time	1	3	0.09101	-0.208	0.38998	0.744	ns	-0.2437	18	18	small
NAcSh L	time	1	3	-0.0683	-0.2934	0.15672	0.745	ns	0.2032	18	18	small
NAcC R -	time	1	3	-0.0914	-0.3969	0.21406	0.751	ns	0.34118	18	18	small
GIDI R -	time	1	2	-0.0836	-0.3639	0.19664	0.753	ns	0.21714	18	18	small
M1 L - S1	time	1	3	0.08378	-0.1985	0.36603	0.755	ns	-0.2907	18	18	small
CPu R - I	time	2	3	0.06915	-0.1663	0.30465	0.759	ns	-0.3703	18	18	small
M2 R - Pr	time	2	3	0.08694	-0.2109	0.38481	0.762	ns	-0.2001	18	18	small
M1 R - Pr	time	1	2	0.07745	-0.1889	0.3438	0.763	ns	-0.1726	18	18	negligible
NAcSh L	time	1	3	-0.0898	-0.3986	0.21901	0.763	ns	0.29545	18	18	small
Cg1 R - N	time	2	3	-0.0722	-0.3209	0.17649	0.764	ns	0.15984	18	18	negligible
NAcC L -	time	1	2	-0.089	-0.3971	0.21917	0.766	ns	0.16476	18	18	negligible
NAcC R -	time	2	3	-0.076	-0.3394	0.18738	0.766	ns	0.1704	18	18	negligible
S1 R - CF	time	1	3	-0.0707	-0.3155	0.17406	0.766	ns	0.19407	18	18	negligible
M1 L - AI	time	1	3	-0.091	-0.4068	0.22474	0.767	ns	0.29702	18	18	small
M2 L - M	time	1	3	-0.0791	-0.3544	0.19621	0.768	ns	0.21588	18	18	small
GIDI R - (	time	2	3	0.06964	-0.1743	0.31361	0.771	ns	-0.2123	18	18	small
GIDI R - I	time	2	3	0.068	-0.1712	0.30724	0.773	ns	-0.2002	18	18	small
PrL R - C	time	2	3	0.07696	-0.1945	0.34846	0.774	ns	-0.2048	18	18	small
PrL L - GI	time	1	3	-0.0705	-0.3196	0.17864	0.775	ns	0.21838	18	18	small
AI R - NA	time	2	3	0.08199	-0.2089	0.37293	0.776	ns	-0.142	18	18	negligible
Cg1 R - F	time	1	3	0.09572	-0.2449	0.43632	0.777	ns	-0.2324	18	18	small
NAcSh L	time	2	3	-0.0631	-0.2882	0.1619	0.778	ns	0.17067	18	18	negligible
M1 R - CI	time	2	3	-0.072	-0.3301	0.18616	0.78	ns	0.26396	18	18	small
GIDI R - (	time	2	3	0.08357	-0.2171	0.38422	0.781	ns	-0.2313	18	18	small
NAcSh R	time	2	3	-0.0869	-0.3992	0.22543	0.781	ns	0.16848	18	18	negligible
Cg1 R - C	time	1	3	0.07536	-0.1963	0.34701	0.782	ns	-0.1537	18	18	negligible
CPu R - (	time	1	2	0.07264	-0.1892	0.33448	0.782	ns	-0.172	18	18	negligible
PrL R - C	time	1	2	-0.0754	-0.3469	0.19612	0.782	ns	0.15488	18	18	negligible
PrL R - M	time	2	3	-0.0779	-0.3593	0.20347	0.783	ns	0.15129	18	18	negligible
NAcC R -	time	1	3	-0.074	-0.3436	0.19558	0.786	ns	0.28096	18	18	small
NAcSh L	time	1	3	0.07368	-0.1947	0.34202	0.786	ns	-0.2101	18	18	small
AI R - GI	time	1	2	-0.0829	-0.3868	0.22104	0.789	ns	0.17824	18	18	negligible
CPu R - N	time	2	3	-0.0735	-0.3433	0.19625	0.789	ns	0.17354	18	18	negligible
GIDI R - I	time	2	3	0.06266	-0.1677	0.29298	0.789	ns	-0.2483	18	18	small
IL R - Cg	time	1	3	-0.077	-0.3598	0.20568	0.789	ns	0.18564	18	18	negligible
IL R - PrL	time	2	3	0.0804	-0.2144	0.37525	0.789	ns	-0.261	18	18	small
PrL R - M	time	1	3	0.06244	-0.1671	0.29202	0.789	ns	-0.1715	18	18	negligible
CPu R - F	time	1	2	-0.0735	-0.344	0.19702	0.79	ns	0.17284	18	18	negligible
GIDI R - (	time	1	3	-0.066	-0.31	0.17796	0.791	ns	0.20255	18	18	small
M2 R - Pr	time	1	3	0.07967	-0.2182	0.37753	0.796	ns	-0.2286	18	18	small
IL R - AI I	time	2	3	-0.0596	-0.2832	0.1641	0.797	ns	0.17152	18	18	negligible
Cg1 L - C	time	2	3	-0.0781	-0.373	0.21685	0.799	ns	0.1875	18	18	negligible
NAcC R -	time	2	3	-0.0712	-0.3421	0.19969	0.802	ns	0.16824	18	18	negligible
NAcSh L	time	1	3	-0.0927	-0.4454	0.2599	0.802	ns	0.18879	18	18	negligible
M2 R - C	time	2	3	-0.0703	-0.3391	0.19843	0.803	ns	0.22178	18	18	small
NAcC L -	time	1	3	-0.0806	-0.3888	0.22751	0.803	ns	0.28973	18	18	small
S1 R - Pr	time	1	2	-0.0608	-0.2937	0.17214	0.804	ns	0.17347	18	18	negligible
PrL L - G	time	1	2	-0.0643	-0.3134	0.18478	0.808	ns	0.1452	18	18	negligible
GIDI R - I	time	1	3	-0.0615	-0.3007	0.17774	0.81	ns	0.24381	18	18	small
S1 R - N	time	2	3	-0.0626	-0.3062	0.18106	0.81	ns	0.15427	18	18	negligible
Cg1 L - S	time	1	3	-0.0654	-0.3212	0.1903	0.811	ns	0.18606	18	18	negligible
Cg1 L - C	time	1	2	0.07524	-0.2197	0.37018	0.812	ns	-0.1509	18	18	negligible
CPu L - F	time	1	2	-0.0747	-0.3677	0.21825	0.812	ns	0.22503	18	18	small
GIDI R - I	time	1	2	-0.0705	-0.3476	0.2066	0.813	ns	0.16734	18	18	negligible
Cg1 R - F	time	1	2	-0.0813	-0.4014	0.23893	0.814	ns	0.18487	18	18	negligible
AI R - AI I	time	1	2	-0.1008	-0.5005	0.2989	0.816	ns	0.32915	18	18	small
PrL R - IL	time	1	2	-0.0783	-0.3892	0.23256	0.816	ns	0.16286	18	18	negligible
NAcC R -	time	1	3	-0.0616	-0.3082	0.18501	0.819	ns	0.20217	18	18	small
Cg1 R - N	time	1	2	0.05787	-0.1749	0.29059	0.821	ns	-0.1559	18	18	negligible
M2 R - IL	time	1	2	0.07388	-0.2238	0.37159	0.821	ns	-0.1918	18	18	negligible
GIDI R - I	time	2	3	0.07142	-0.2174	0.36026	0.822	ns	-0.1707	18	18	negligible
CPu L - N	time	1	2	0.05964	-0.1817	0.30099	0.823	ns	-0.1296	18	18	negligible
PrL R - N	time	1	2	0.06326	-0.1934	0.31995	0.823	ns	-0.1553	18	18	negligible
Cg1 R - N	time	1	3	-0.0572	-0.29	0.1755	0.824	ns	0.13796	18	18	negligible
PrL R - S	time	1	3	0.05749	-0.1759	0.29084	0.824	ns	-0.1506	18	18	negligible
S1 R - GI	time	1	3	-0.0725	-0.3668	0.22188	0.824	ns	0.21792	18	18	small
AI R - S1	time	1	3	-0.0725	-0.3689	0.22388	0.826	ns	0.23013	18	18	small
Cg1 R - II	time	1	3	-0.0581	-0.2971	0.18096	0.828	ns	0.19822	18	18	negligible
GIDI R - (	time	2	3	0.06181	-0.194	0.31766	0.83	ns	-0.1836	18	18	negligible
M1 R - IL	time	1	3	0.05907	-0.1859	0.30405	0.83	ns	-0.1897	18	18	negligible
Cg1 L - S	time	1	2	0.06126	-0.1945	0.31701	0.832	ns	-0.1607	18	18	negligible
AI R - S1	time	1	2	-0.0744	-0.3863	0.23742	0.833	ns	0.22611	18	18	small
PrL R - N	time	1	3	-0.061	-0.3177	0.19571	0.835	ns	0.18611	18	18	negligible
NAcSh L	time	1	3	0.06573	-0.2129	0.34436	0.837	ns	-0.125	18	18	negligible
NAcC R -	time	1	3	0.06194	-0.2015	0.32534	0.838	ns	-0.202	18	18	small
AI R - Cg	time	2	3	0.0639	-0.2098	0.33758	0.84	ns	-0.1401	18	18	negligible

M1 R - C	time	1	2	0.06061	-0.1996	0.32078	0.841	ns		-0.1439	18	18	negligible
M1 R - M	time	1	3	-0.0974	-0.5156	0.32082	0.841	ns		0.22538	18	18	small
NAcC R -	time	1	3	0.0702	-0.2316	0.37203	0.841	ns		-0.1941	18	18	negligible
PrL L - M	time	2	3	-0.0627	-0.3342	0.2088	0.843	ns		0.11796	18	18	negligible
PrL R - S	time	2	3	-0.0538	-0.2872	0.17951	0.843	ns		0.15244	18	18	negligible
M2 R - Cl	time	1	2	-0.0609	-0.3259	0.20401	0.844	ns		0.14869	18	18	negligible
M1 R - M	time	1	2	-0.0602	-0.3246	0.20421	0.847	ns		0.11148	18	18	negligible
PrL R - C	time	1	2	0.07144	-0.242	0.3849	0.847	ns		-0.1748	18	18	negligible
NAcSh R	time	1	2	-0.0622	-0.3362	0.21183	0.848	ns		0.12171	18	18	negligible
AI R - Cg	time	2	3	0.0496	-0.1702	0.26941	0.85	ns		-0.1486	18	18	negligible
AI R - M1	time	1	3	-0.0702	-0.3816	0.24123	0.85	ns		0.23642	18	18	small
S1 L - AI	time	2	3	-0.0797	-0.4338	0.27437	0.85	ns		0.1781	18	18	negligible
IL R - CP	time	1	2	0.05783	-0.2008	0.31644	0.852	ns		-0.1473	18	18	negligible
M1 R - M	time	2	3	-0.0589	-0.3233	0.20549	0.853	ns		0.14718	18	18	negligible
AI R - NA	time	1	3	-0.0667	-0.3683	0.23495	0.855	ns		0.30558	18	18	small
GIDI R -	time	1	2	-0.0509	-0.2813	0.17938	0.855	ns		0.12024	18	18	negligible
S1 R - IL	time	2	3	0.05317	-0.1902	0.29654	0.858	ns		-0.2073	18	18	small
M1 R - N	time	1	2	0.05003	-0.1815	0.28158	0.861	ns		-0.1098	18	18	negligible
NAcSh L	time	1	2	0.0576	-0.2107	0.32594	0.863	ns		-0.1533	18	18	negligible
Cg1 R - I	time	1	2	0.05097	-0.1881	0.29	0.865	ns		-0.1696	18	18	negligible
NAcSh R	time	1	3	-0.0809	-0.4643	0.30255	0.867	ns		0.2292	18	18	small
PrL L - S	time	1	2	-0.0506	-0.2931	0.1918	0.87	ns		0.11624	18	18	negligible
CPu R -	time	1	2	-0.056	-0.3258	0.21378	0.871	ns		0.13779	18	18	negligible
NAcSh R	time	2	3	-0.0629	-0.365	0.23925	0.871	ns		0.10733	18	18	negligible
IL L - M2	time	1	3	-0.0543	-0.3171	0.20846	0.872	ns		0.16242	18	18	negligible
NAcSh R	time	1	2	-0.0655	-0.3837	0.25264	0.873	ns		0.12056	18	18	negligible
PrL R - M	time	1	3	0.05784	-0.2235	0.33921	0.873	ns		-0.1176	18	18	negligible
GIDI R -	time	1	3	-0.0575	-0.3377	0.22279	0.874	ns		0.18493	18	18	negligible
M2 R - M	time	2	3	0.06204	-0.241	0.36505	0.874	ns		-0.148	18	18	negligible
NAcC R -	time	1	3	-0.0547	-0.3218	0.21238	0.874	ns		0.19644	18	18	negligible
Cg1 R -	time	1	2	0.0516	-0.2025	0.30575	0.876	ns		-0.1356	18	18	negligible
AI R - IL	time	1	3	0.05424	-0.2135	0.32199	0.877	ns		-0.1699	18	18	negligible
M2 R - IL	time	2	3	-0.0603	-0.358	0.23741	0.877	ns		0.17988	18	18	negligible
NAcC R -	time	1	3	0.05476	-0.2161	0.32566	0.877	ns		-0.1822	18	18	negligible
NAcC R -	time	1	3	0.05421	-0.2169	0.32536	0.88	ns		-0.1372	18	18	negligible
S1 R - Pr	time	1	3	-0.0464	-0.2793	0.18653	0.881	ns		0.19848	18	18	negligible
M1 R - M	time	2	3	0.08231	-0.3359	0.50051	0.883	ns		-0.2318	18	18	small
NAcSh R	time	2	3	-0.054	-0.328	0.22005	0.883	ns		0.12062	18	18	negligible
Cg1 L - A	time	1	2	-0.0601	-0.3672	0.24705	0.885	ns		0.12806	18	18	negligible
M1 R - IL	time	2	3	0.04776	-0.1972	0.29274	0.885	ns		-0.1394	18	18	negligible
IL R - CP	time	2	3	-0.05	-0.3086	0.20859	0.887	ns		0.26374	18	18	small
M1 L - GI	time	2	3	0.06257	-0.261	0.38616	0.887	ns		-0.1449	18	18	negligible
PrL R - N	time	1	2	0.05708	-0.2374	0.35151	0.887	ns		-0.1401	18	18	negligible
CPu L - F	time	2	3	0.05636	-0.2366	0.34933	0.888	ns		-0.1611	18	18	negligible
NAcC R -	time	1	2	0.0578	-0.244	0.35963	0.889	ns		-0.1467	18	18	negligible
CPu L -	time	1	3	-0.0608	-0.38	0.2584	0.89	ns		0.17311	18	18	negligible
CPu R -	time	1	3	-0.0516	-0.3221	0.21887	0.89	ns		0.19658	18	18	negligible
PrL R - A	time	2	3	0.05425	-0.2303	0.33878	0.89	ns		-0.1264	18	18	negligible
Cg1 R -	time	1	2	0.06186	-0.2645	0.38823	0.891	ns		-0.1327	18	18	negligible
M1 L - AI	time	2	3	0.05992	-0.2559	0.37571	0.891	ns		-0.1385	18	18	negligible
S1 R - N	time	1	2	0.04503	-0.1925	0.28252	0.891	ns		-0.1217	18	18	negligible
IL L - Cg	time	1	3	-0.0488	-0.3071	0.20949	0.892	ns		0.14551	18	18	negligible
NAcSh R	time	2	3	-0.0551	-0.3469	0.23671	0.892	ns		0.16676	18	18	negligible
NAcC R -	time	1	3	-0.0557	-0.3519	0.2405	0.893	ns		0.12209	18	18	negligible
Cg1 R - S	time	1	2	0.04495	-0.1969	0.28685	0.895	ns		-0.1308	18	18	negligible
S1 R - IL	time	1	3	0.05121	-0.2234	0.32583	0.895	ns		-0.1805	18	18	negligible
Cg1 R - S	time	1	3	-0.0449	-0.2868	0.19702	0.896	ns		0.10949	18	18	negligible
NAcSh L	time	1	2	0.04343	-0.1916	0.27846	0.896	ns		-0.133	18	18	negligible
IL R - NA	time	1	3	-0.0509	-0.3285	0.22664	0.898	ns		0.1426	18	18	negligible
NAcSh L	time	2	3	-0.0567	-0.3655	0.25209	0.898	ns		0.12081	18	18	negligible
AI R - AI	time	2	3	-0.0729	-0.4727	0.32677	0.899	ns		0.18623	18	18	negligible
Cg1 R -	time	1	3	-0.0459	-0.2974	0.20552	0.899	ns		0.12932	18	18	negligible
NAcC R -	time	1	2	0.04916	-0.2204	0.31876	0.899	ns		-0.0956	18	18	negligible
NAcSh R	time	2	3	-0.0511	-0.3317	0.22944	0.899	ns		0.11368	18	18	negligible
AI R - S1	time	2	3	0.05641	-0.2554	0.36826	0.9	ns		-0.1366	18	18	negligible
NAcC L -	time	1	3	-0.0455	-0.2971	0.2061	0.901	ns		0.10728	18	18	negligible
AI R - PrL	time	1	3	0.03866	-0.1779	0.25517	0.903	ns		-0.1353	18	18	negligible
M1 L - GI	time	1	3	-0.0576	-0.3812	0.26603	0.904	ns		0.26398	18	18	small
M1 R - C	time	1	3	0.04614	-0.214	0.30631	0.904	ns		-0.1214	18	18	negligible
M1 R - Pr	time	1	2	-0.045	-0.2998	0.2098	0.905	ns		0.10451	18	18	negligible
NAcC L -	time	2	3	-0.0512	-0.3408	0.23841	0.905	ns		0.10792	18	18	negligible
Cg1 R -	time	2	3	-0.0579	-0.3873	0.27146	0.906	ns		0.09279	18	18	negligible
PrL L - S	time	1	3	-0.0426	-0.2851	0.19981	0.906	ns		0.13489	18	18	negligible
CPu R - S	time	2	3	-0.0495	-0.3334	0.23436	0.907	ns		0.11101	18	18	negligible
NAcSh R	time	1	3	0.0546	-0.2577	0.3669	0.907	ns		-0.1053	18	18	negligible
PrL L - AI	time	2	3	0.04914	-0.2322	0.33051	0.907	ns		-0.136	18	18	negligible
PrL R - N	time	2	3	-0.0512	-0.3456	0.24323	0.908	ns		0.11568	18	18	negligible
Cg1 R -	time	2	3	0.04387	-0.2103	0.29802	0.909	ns		-0.1014	18	18	negligible
IL L - S1	time	1	3	0.03956	-0.1954	0.2745	0.913	ns		-0.125	18	18	negligible
M2 R - Pr	time	1	3	-0.0427	-0.3002	0.21471	0.915	ns		0.13541	18	18	negligible
NAcC R -	time	1	2	-0.0511	-0.3588	0.2566	0.915	ns		0.10541	18	18	negligible
NAcSh R	time	1	2	0.04854	-0.2433	0.34033	0.915	ns		-0.1335	18	18	negligible
NAcC L -	time	1	3	0.04802	-0.2416	0.33762	0.916	ns		-0.1223	18	18	negligible
GIDI R -	time	2	3	0.04468	-0.2295	0.31888	0.918	ns		-0.1384	18	18	negligible
IL R - CP	time	2	3	-0.0369	-0.2623	0.18855	0.918	ns		0.12615	18	18	negligible
CPu R -	time	1	2	-0.0445	-0.3194	0.23039	0.919	ns		0.09789	18	18	negligible

PrL R - C	time	2	3	-0.0508	-0.3643	0.26264	0.919	ns		0.11854	18	18	negligible
CPu L - M	time	1	2	0.05105	-0.2682	0.37028	0.921	ns		-0.0865	18	18	negligible
NAcC R -	time	2	3	-0.0407	-0.295	0.21366	0.921	ns		0.0876	18	18	negligible
M2 R - S	time	1	3	-0.049	-0.358	0.2599	0.922	ns		0.09215	18	18	negligible
AI R - GI	time	1	3	-0.0481	-0.352	0.25583	0.923	ns		0.14929	18	18	negligible
M2 L - GI	time	1	2	-0.047	-0.3463	0.25231	0.924	ns		0.08554	18	18	negligible
NAcC L -	time	1	2	0.03942	-0.2166	0.29546	0.927	ns		-0.0811	18	18	negligible
Cg1 L - A	time	1	3	-0.0463	-0.3534	0.26083	0.93	ns		0.10564	18	18	negligible
PrL R - N	time	2	3	-0.0364	-0.2778	0.20505	0.93	ns		0.08257	18	18	negligible
S1 R - IL	time	1	3	0.03673	-0.2066	0.2801	0.93	ns		-0.1464	18	18	negligible
CPu R - N	time	1	3	-0.0407	-0.3155	0.23422	0.932	ns		0.13281	18	18	negligible
NAcC L -	time	1	3	-0.0326	-0.2519	0.18674	0.932	ns		0.12422	18	18	negligible
S1 R - Cg	time	1	2	-0.036	-0.2807	0.2088	0.933	ns		0.09157	18	18	negligible
AI R - S1	time	2	3	0.04328	-0.2531	0.33966	0.934	ns		-0.1234	18	18	negligible
M2 R - IL	time	1	3	0.04053	-0.2396	0.3207	0.935	ns		-0.1362	18	18	negligible
NAcSh R	time	1	2	-0.0402	-0.3173	0.23699	0.935	ns		0.0923	18	18	negligible
IL L - AI L	time	1	3	0.03158	-0.1892	0.25241	0.936	ns		-0.1109	18	18	negligible
NAcC R -	time	2	3	-0.0383	-0.3054	0.22884	0.936	ns		0.0932	18	18	negligible
NAcSh R	time	1	3	-0.0399	-0.3171	0.23727	0.936	ns		0.11516	18	18	negligible
CPu R - C	time	2	3	-0.049	-0.3914	0.29354	0.937	ns		0.12225	18	18	negligible
M2 R - Cg	time	1	2	-0.0442	-0.3537	0.26529	0.937	ns		0.08743	18	18	negligible
NAcC L -	time	1	3	-0.0393	-0.3161	0.23752	0.937	ns		0.08796	18	18	negligible
IL R - S1	time	1	3	0.03932	-0.2439	0.32254	0.94	ns		-0.0975	18	18	negligible
GIDI R - N	time	1	3	-0.0406	-0.3368	0.25555	0.941	ns		0.12981	18	18	negligible
CPu R - N	time	2	3	-0.0405	-0.336	0.25498	0.942	ns		0.07659	18	18	negligible
NAcSh R	time	2	3	0.044	-0.2784	0.36643	0.942	ns		-0.0735	18	18	negligible
NAcSh R	time	1	3	-0.0429	-0.3611	0.27523	0.943	ns		0.12762	18	18	negligible
NAcSh R	time	1	3	-0.0409	-0.343	0.26125	0.943	ns		0.17188	18	18	negligible
CPu L - G	time	2	3	-0.0382	-0.3295	0.25305	0.946	ns		0.09591	18	18	negligible
PrL R - A	time	1	3	0.03716	-0.2474	0.32169	0.947	ns		-0.101	18	18	negligible
CPu R - C	time	1	3	-0.0339	-0.2958	0.22793	0.948	ns		0.08117	18	18	negligible
M2 R - NA	time	2	3	-0.0343	-0.2997	0.23104	0.948	ns		0.1008	18	18	negligible
NAcSh R	time	2	3	-0.0421	-0.3682	0.28401	0.948	ns		0.08232	18	18	negligible
NAcC L -	time	2	3	-0.0279	-0.2472	0.19144	0.949	ns		0.09586	18	18	negligible
CPu R - C	time	1	3	-0.0433	-0.3858	0.29916	0.95	ns		0.10036	18	18	negligible
GIDI L - A	time	2	3	-0.0499	-0.4437	0.34391	0.95	ns		0.12921	18	18	negligible
M1 R - Pr	time	1	3	-0.0315	-0.2863	0.22327	0.952	ns		0.09688	18	18	negligible
AI R - NA	time	1	3	-0.0356	-0.3265	0.25533	0.953	ns		0.09837	18	18	negligible
M2 R - Cg	time	1	3	-0.0379	-0.3474	0.27156	0.953	ns		0.09843	18	18	negligible
NAcSh R	time	1	3	-0.0397	-0.3658	0.28635	0.953	ns		0.07696	18	18	negligible
Cg1 R - A	time	2	3	0.03454	-0.2513	0.32037	0.954	ns		-0.0913	18	18	negligible
IL R - GID	time	1	3	0.02644	-0.1923	0.24521	0.954	ns		-0.0974	18	18	negligible
M2 R - NA	time	1	3	-0.0316	-0.297	0.23373	0.955	ns		0.12199	18	18	negligible
GIDI R - N	time	1	3	-0.0397	-0.375	0.29555	0.956	ns		0.11661	18	18	negligible
M2 L - GI	time	1	3	-0.0355	-0.3348	0.26386	0.956	ns		0.12202	18	18	negligible
NAcSh L	time	1	3	0.02912	-0.2164	0.27469	0.956	ns		-0.0616	18	18	negligible
IL L - PrL	time	2	3	0.02962	-0.2236	0.2828	0.957	ns		-0.0756	18	18	negligible
NAcSh L	time	1	3	0.03669	-0.277	0.35039	0.957	ns		-0.1733	18	18	negligible
Cg1 R - N	time	2	3	-0.0378	-0.3641	0.28862	0.958	ns		0.06314	18	18	negligible
M2 L - S1	time	2	3	0.03424	-0.261	0.32949	0.958	ns		-0.0705	18	18	negligible
PrL L - M	time	1	3	0.03131	-0.2402	0.30282	0.958	ns		-0.0671	18	18	negligible
AI R - GID	time	2	3	0.0348	-0.2691	0.33871	0.959	ns		-0.0744	18	18	negligible
AI R - IL P	time	1	2	0.03019	-0.2376	0.29794	0.96	ns		-0.0792	18	18	negligible
M2 R - IL	time	2	3	0.03178	-0.2484	0.31196	0.96	ns		-0.0784	18	18	negligible
M2 R - NA	time	1	2	-0.0252	-0.2522	0.20189	0.961	ns		0.08094	18	18	negligible
Cg1 R - M	time	1	2	0.03501	-0.2944	0.36439	0.964	ns		-0.058	18	18	negligible
NAcC R -	time	1	3	-0.0273	-0.2816	0.22702	0.964	ns		0.09407	18	18	negligible
NAcSh L	time	1	2	-0.0331	-0.3419	0.27573	0.964	ns		0.05589	18	18	negligible
S1 R - AI	time	2	3	0.03351	-0.28	0.34702	0.964	ns		-0.0788	18	18	negligible
IL L - S1	time	2	3	0.02468	-0.2103	0.25962	0.965	ns		-0.078	18	18	negligible
IL R - M1	time	1	3	0.02533	-0.2146	0.26525	0.965	ns		-0.0691	18	18	negligible
AI R - M2	time	2	3	0.03449	-0.3002	0.36916	0.966	ns		-0.0703	18	18	negligible
NAcC R -	time	1	3	-0.0319	-0.3396	0.27573	0.966	ns		0.12362	18	18	negligible
AI R - PrL	time	2	3	0.02156	-0.1949	0.23807	0.969	ns		-0.0907	18	18	negligible
M2 L - AI	time	1	3	-0.0305	-0.3415	0.28039	0.97	ns		0.07297	18	18	negligible
S1 L - GII	time	2	3	0.03249	-0.2999	0.3649	0.97	ns		-0.0799	18	18	negligible
M1 R - IL	time	1	3	0.02867	-0.2697	0.32705	0.971	ns		-0.0867	18	18	negligible
GIDI R - S	time	2	3	0.02615	-0.2541	0.30641	0.972	ns		-0.061	18	18	negligible
PrL R - M	time	2	3	-0.0215	-0.2511	0.20803	0.972	ns		0.05752	18	18	negligible
M1 R - Pr	time	2	3	0.02476	-0.2416	0.29111	0.973	ns		-0.0695	18	18	negligible
AI R - IL P	time	2	3	0.02405	-0.2437	0.29181	0.974	ns		-0.057	18	18	negligible
IL L - GID	time	1	2	-0.0184	-0.2237	0.1868	0.974	ns		0.04795	18	18	negligible
IL L - GID	time	2	3	0.01853	-0.1867	0.22376	0.974	ns		-0.0534	18	18	negligible
CPu L - S	time	1	2	-0.0276	-0.3449	0.28974	0.976	ns		0.06147	18	18	negligible
PrL R - C	time	2	3	0.02199	-0.2291	0.27311	0.976	ns		-0.0747	18	18	negligible
GIDI R - N	time	2	3	0.02099	-0.2251	0.26708	0.977	ns		-0.0618	18	18	negligible
GIDI R - I	time	1	3	0.02287	-0.2542	0.29996	0.978	ns		-0.0647	18	18	negligible
M2 R - NA	time	2	3	0.02607	-0.2884	0.3405	0.978	ns		-0.0666	18	18	negligible
PrL R - C	time	1	3	0.02074	-0.2304	0.27186	0.978	ns		-0.053	18	18	negligible
CPu R - N	time	1	3	-0.024	-0.3194	0.27153	0.979	ns		0.05436	18	18	negligible
CPu R - N	time	1	2	0.02353	-0.2628	0.30984	0.979	ns		-0.0479	18	18	negligible
CPu R - P	time	2	3	0.02184	-0.2487	0.29234	0.979	ns		-0.0522	18	18	negligible
NAcC R -	time	2	3	-0.0202	-0.2708	0.23042	0.979	ns		0.0606	18	18	negligible
AI R - GID	time	2	3	0.02249	-0.258	0.30298	0.98	ns		-0.059	18	18	negligible
AI R - PrL	time	1	2	0.01709	-0.1994	0.2336	0.98	ns		-0.0471	18	18	negligible
Cg1 R - C	time	1	3	0.02275	-0.2609	0.30639	0.98	ns		-0.0911	18	18	negligible

S1 R - M2	time	1	3	-0.0259	-0.3542	0.3024	0.98	ns	0.06327	18	18	negligible
Cg1 R - P	time	1	3	0.02446	-0.2957	0.34465	0.981	ns	-0.0585	18	18	negligible
CPu R - (	time	2	3	0.02318	-0.2767	0.32304	0.981	ns	-0.0633	18	18	negligible
PrL R - G	time	1	3	0.01698	-0.2037	0.23761	0.981	ns	-0.046	18	18	negligible
M2 R - NA	time	1	2	-0.017	-0.2407	0.20676	0.982	ns	0.04122	18	18	negligible
Cg1 R - N	time	1	3	0.02411	-0.3023	0.35048	0.983	ns	-0.0457	18	18	negligible
NAcC R -	time	1	2	0.01838	-0.2322	0.26899	0.983	ns	-0.0498	18	18	negligible
NAcSh R	time	1	2	0.022	-0.2801	0.32411	0.983	ns	-0.0447	18	18	negligible
Cg1 R - Il	time	1	3	-0.0194	-0.2973	0.25844	0.984	ns	0.05863	18	18	negligible
NAcSh R	time	2	3	0.02258	-0.2956	0.34075	0.984	ns	-0.0442	18	18	negligible
S1 R - S1	time	2	3	-0.0212	-0.3173	0.27491	0.984	ns	0.05004	18	18	negligible
Cg1 R - A	time	1	3	0.01963	-0.2662	0.30546	0.985	ns	-0.0408	18	18	negligible
Cg1 R - M	time	1	3	-0.0229	-0.3523	0.30647	0.985	ns	0.04208	18	18	negligible
M1 R - NA	time	1	2	-0.0146	-0.23	0.20082	0.985	ns	0.04082	18	18	negligible
M2 L - S1	time	1	3	0.02049	-0.2748	0.31574	0.985	ns	-0.0475	18	18	negligible
S1 R - IL	time	1	2	-0.0164	-0.2598	0.22692	0.985	ns	0.06265	18	18	negligible
M2 R - Cl	time	1	3	-0.0172	-0.2789	0.24456	0.986	ns	0.06064	18	18	negligible
PrL R - C	time	1	3	0.02063	-0.2928	0.33408	0.986	ns	-0.0513	18	18	negligible
S1 R - GI	time	2	3	0.0192	-0.2752	0.31355	0.986	ns	-0.0411	18	18	negligible
S1 R - Pr	time	1	3	0.01339	-0.1881	0.21486	0.986	ns	-0.0457	18	18	negligible
CPu L - P	time	1	3	-0.0184	-0.3113	0.2746	0.987	ns	0.05387	18	18	negligible
GIDI R - (	time	2	3	0.01755	-0.2526	0.28773	0.987	ns	-0.0427	18	18	negligible
GIDI R - S	time	1	2	0.01838	-0.2653	0.3021	0.987	ns	-0.0402	18	18	negligible
IL L - S1	time	1	2	0.01488	-0.2201	0.24982	0.987	ns	-0.0461	18	18	negligible
PrL L - M	time	1	3	-0.0158	-0.2598	0.22818	0.987	ns	0.05592	18	18	negligible
NAcC R -	time	2	3	0.01913	-0.2885	0.32681	0.988	ns	-0.0481	18	18	negligible
NAcC R -	time	1	2	-0.0165	-0.2836	0.25066	0.988	ns	0.03842	18	18	negligible
PrL R - A	time	1	2	-0.0171	-0.3016	0.26744	0.988	ns	0.03361	18	18	negligible
S1 R - Pr	time	2	3	0.0144	-0.2185	0.24734	0.988	ns	-0.0497	18	18	negligible
AI R - S1	time	1	3	-0.018	-0.3299	0.29383	0.989	ns	0.04241	18	18	negligible
NAcSh L	time	2	3	0.01608	-0.2523	0.28443	0.989	ns	-0.0412	18	18	negligible
CPu R - N	time	1	2	0.01655	-0.2789	0.31203	0.99	ns	-0.029	18	18	negligible
M1 R - Cg	time	2	3	-0.0145	-0.2746	0.24569	0.99	ns	0.04328	18	18	negligible
Cg1 R - A	time	1	2	-0.0149	-0.3007	0.27092	0.991	ns	0.03429	18	18	negligible
GIDI R - S	time	1	3	0.01518	-0.2685	0.29891	0.991	ns	-0.0596	18	18	negligible
M1 R - Cl	time	2	3	-0.0144	-0.292	0.26318	0.991	ns	0.04691	18	18	negligible
M1 R - Pr	time	2	3	0.01347	-0.2413	0.26825	0.991	ns	-0.0394	18	18	negligible
NAcC R -	time	1	2	0.01336	-0.241	0.26769	0.991	ns	-0.0259	18	18	negligible
S1 R - CP	time	1	2	0.01519	-0.2663	0.29666	0.991	ns	-0.0374	18	18	negligible
S1 R - S1	time	1	3	-0.0153	-0.3114	0.2808	0.991	ns	0.03481	18	18	negligible
GIDI R - I	time	2	3	0.01447	-0.2728	0.30177	0.992	ns	-0.0295	18	18	negligible
GIDI R - I	time	1	3	0.01172	-0.2186	0.24204	0.992	ns	-0.0369	18	18	negligible
S1 R - M1	time	1	3	-0.0158	-0.3307	0.29911	0.992	ns	0.04756	18	18	negligible
IL R - AI I	time	1	3	0.01072	-0.213	0.23439	0.993	ns	-0.036	18	18	negligible
IL R - M2	time	1	3	-0.0141	-0.3064	0.27824	0.993	ns	0.03255	18	18	negligible
M1 R - Cg	time	1	2	0.0115	-0.2349	0.25786	0.993	ns	-0.0259	18	18	negligible
M1 R - IL	time	1	2	0.01131	-0.2337	0.25629	0.993	ns	-0.0412	18	18	negligible
M2 L - S1	time	1	2	-0.0137	-0.309	0.2815	0.993	ns	0.02866	18	18	negligible
M2 R - IL	time	1	3	0.01358	-0.2841	0.31129	0.993	ns	-0.0327	18	18	negligible
S1 R - M2	time	1	2	-0.0134	-0.3096	0.2828	0.993	ns	0.02598	18	18	negligible
Cg1 L - A	time	2	3	0.01378	-0.2934	0.32092	0.994	ns	-0.0339	18	18	negligible
CPu R - N	time	2	3	-0.0128	-0.2991	0.27355	0.994	ns	0.02839	18	18	negligible
GIDI R - A	time	2	3	0.01319	-0.288	0.31435	0.994	ns	-0.0364	18	18	negligible
Cg1 R - C	time	1	2	-0.0108	-0.2824	0.26089	0.995	ns	0.02511	18	18	negligible
CPu L - Il	time	1	3	0.00751	-0.1854	0.20045	0.995	ns	-0.0253	18	18	negligible
CPu R - I	time	1	3	-0.0095	-0.245	0.22601	0.995	ns	0.03104	18	18	negligible
CPu R - N	time	1	3	0.01077	-0.2755	0.29708	0.995	ns	-0.0303	18	18	negligible
M2 L - GI	time	1	3	0.01155	-0.2878	0.31086	0.995	ns	-0.024	18	18	negligible
NAcC R -	time	2	3	0.0124	-0.2894	0.31423	0.995	ns	-0.0283	18	18	negligible
CPu R - N	time	1	3	-0.0124	-0.3519	0.32714	0.996	ns	0.03574	18	18	negligible
M1 R - NA	time	1	2	0.00745	-0.2146	0.22945	0.996	ns	-0.0175	18	18	negligible
PrL L - M	time	2	3	-0.0082	-0.2522	0.23576	0.996	ns	0.0232	18	18	negligible
PrL R - N	time	1	3	0.01085	-0.2982	0.31994	0.996	ns	-0.0323	18	18	negligible
S1 R - CP	time	2	3	0.00835	-0.2364	0.25314	0.996	ns	-0.0224	18	18	negligible
S1 R - M1	time	1	3	-0.0099	-0.2841	0.26427	0.996	ns	0.02584	18	18	negligible
AI R - M1	time	1	3	-0.0125	-0.3958	0.37071	0.997	ns	0.02796	18	18	negligible
AI R - M2	time	1	3	-0.0116	-0.3786	0.35537	0.997	ns	0.03217	18	18	negligible
IL R - CP	time	1	3	0.0078	-0.2508	0.26641	0.997	ns	-0.0205	18	18	negligible
M2 R - IL	time	1	2	0.00874	-0.2714	0.28892	0.997	ns	-0.0229	18	18	negligible
NAcC L -	time	1	2	0.00717	-0.2157	0.22999	0.997	ns	-0.0191	18	18	negligible
PrL L - M	time	1	2	-0.0076	-0.2516	0.23641	0.997	ns	0.01699	18	18	negligible
PrL L - S1	time	2	3	0.00801	-0.2344	0.25044	0.997	ns	-0.0245	18	18	negligible
PrL R - N	time	2	3	0.00988	-0.2992	0.31897	0.997	ns	-0.0185	18	18	negligible
S1 R - Cg	time	1	2	-0.0067	-0.2455	0.23206	0.997	ns	0.01934	18	18	negligible
AI R - PrL	time	1	3	-0.006	-0.2407	0.22862	0.998	ns	0.02284	18	18	negligible
Cg1 L - M	time	1	3	0.00729	-0.3228	0.33742	0.998	ns	-0.0138	18	18	negligible
GIDI R - I	time	1	3	0.00615	-0.2281	0.24044	0.998	ns	-0.0221	18	18	negligible
IL L - M1	time	1	3	0.00531	-0.2006	0.21121	0.998	ns	-0.0201	18	18	negligible
M2 R - Pr	time	1	2	-0.0073	-0.3051	0.29059	0.998	ns	0.01877	18	18	negligible
NAcC L -	time	2	3	0.00834	-0.2998	0.31647	0.998	ns	-0.0206	18	18	negligible
NAcSh L	time	1	2	0.00595	-0.2172	0.22914	0.998	ns	-0.0166	18	18	negligible
NAcSh L	time	1	2	-0.0052	-0.2302	0.21987	0.998	ns	0.01762	18	18	negligible
NAcSh L	time	1	2	-0.0063	-0.258	0.24544	0.998	ns	0.01333	18	18	negligible
NAcSh R	time	1	3	-0.0065	-0.2983	0.28525	0.998	ns	0.01755	18	18	negligible
PrL L - G	time	2	3	-0.0061	-0.2552	0.24298	0.998	ns	0.01798	18	18	negligible
S1 R - NA	time	1	2	-0.0055	-0.2427	0.23165	0.998	ns	0.01214	18	18	negligible

CPu R - (	time	1	2	0.00562	-0.3369	0.34812	0.999	ns		-0.0097	18	18	negligible
CPu R - N	time	2	3	0.00382	-0.271	0.2787	0.999	ns		-0.0086	18	18	negligible
IL R - NA	time	1	3	0.0036	-0.2398	0.24699	0.999	ns		-0.0113	18	18	negligible
M1 R - N/	time	2	3	0.00509	-0.2897	0.29989	0.999	ns		-0.0111	18	18	negligible
M2 R - C(	time	2	3	0.00627	-0.3032	0.31576	0.999	ns		-0.0206	18	18	negligible
NAcC L -	time	1	2	-0.0047	-0.224	0.21461	0.999	ns		0.01366	18	18	negligible
NAcC L -	time	2	3	0.00412	-0.2461	0.25439	0.999	ns		-0.0106	18	18	negligible
NAcSh R	time	1	3	-0.0034	-0.2687	0.26199	0.999	ns		0.00992	18	18	negligible
PrL R - N	time	1	3	0.00587	-0.2886	0.30031	0.999	ns		-0.0159	18	18	negligible
S1 R - S1	time	1	2	0.00589	-0.2902	0.30199	0.999	ns		-0.0131	18	18	negligible
Cg1 L - G	time	1	3	-0.0028	-0.2978	0.2921	1	ns		0.01189	18	18	negligible
Cg1 R - (	time	1	2	0.00099	-0.2545	0.25646	1	ns		-0.0023	18	18	negligible
CPu R - N	time	1	2	0.0002	-0.2663	0.26672	1	ns		-0.0009	18	18	negligible
CPu R - N	time	1	3	-0.0009	-0.2674	0.2656	1	ns		0.0028	18	18	negligible
CPu R - N	time	2	3	-0.0011	-0.2676	0.2654	1	ns		0.0031	18	18	negligible
GIDI R - S	time	2	3	-0.0032	-0.2869	0.28053	1	ns		0.00725	18	18	negligible
IL L - GID	time	1	3	#######	-0.2051	0.20533	1	ns		-0.0004	18	18	negligible
IL R - NA	time	1	3	0.00278	-0.2238	0.22932	1	ns		-0.0092	18	18	negligible
M2 R - N/	time	1	2	0.00269	-0.2627	0.26807	1	ns		-0.0063	18	18	negligible
NAcC R -	time	1	3	-0.0018	-0.2524	0.2488	1	ns		0.00828	18	18	negligible
NAcSh R	time	1	2	0.00233	-0.3238	0.32842	1	ns		-0.0054	18	18	negligible
NAcSh R	time	2	3	0.00028	-0.2769	0.27745	1	ns		-0.0007	18	18	negligible
PrL R - C	time	1	2	-0.0012	-0.2524	0.24987	1	ns		0.0035	18	18	negligible
PrL R - C	time	1	3	0.00158	-0.2699	0.27308	1	ns		-0.0045	18	18	negligible
PrL R - N	time	1	2	0.00097	-0.3081	0.31006	1	ns		-0.0021	18	18	negligible

Figure 2B. ROI-dependent effects of treatment, time, and sex factors

Sample size: n = 18 rats, 9 females

Three-way mixed-effects ANOVA with within-subjects factors of treatment and time and between-subjects factor of sex, stratified by ROI

roi	Effect	DFn	DFd	F	p	p<.05	ges
AI R - AI L	sex	1	16	0.321	0.579		0.009
AI R - AI L	treatmt	1	16	0.002	0.964		3.41E-05
AI R - AI L	time	1	16	6.108	0.025	*	0.054
AI R - AI L	sex:treatmt	1	16	2.011	0.175		0.032
AI R - AI L	sex:time	1	16	1.314	0.269		0.012
AI R - AI L	treatmt:time	1	16	2.798	0.114		0.021
AI R - AI L	sex:treatmt:time	1	16	0.086	0.773		0.000658
AI R - Cg1 L	sex	1	16	0.053	0.821		0.001
AI R - Cg1 L	treatmt	1	16	0.702	0.414		0.011
AI R - Cg1 L	time	1	16	2.679	0.121		0.034
AI R - Cg1 L	sex:treatmt	1	16	2.253	0.153		0.035
AI R - Cg1 L	sex:time	1	16	0.058	0.813		0.000763
AI R - Cg1 L	treatmt:time	1	16	0.032	0.859		0.000385
AI R - Cg1 L	sex:treatmt:time	1	16	4.007	0.063		0.045
AI R - Cg1 R	sex	1	16	0.219	0.646		0.003
AI R - Cg1 R	treatmt	1	16	0.965	0.341		0.015
AI R - Cg1 R	time	1	16	1.647	0.218		0.023
AI R - Cg1 R	sex:treatmt	1	16	0.061	0.808		0.000965
AI R - Cg1 R	sex:time	1	16	0.034	0.855		0.000494
AI R - Cg1 R	treatmt:time	1	16	0.046	0.833		0.000767
AI R - Cg1 R	sex:treatmt:time	1	16	1.707	0.21		0.028
AI R - CPu L	sex	1	16	2.31	0.148		0.035
AI R - CPu L	treatmt	1	16	0.00089	0.977		1.54E-05
AI R - CPu L	time	1	16	8.708	0.009	*	0.145
AI R - CPu L	sex:treatmt	1	16	0.148	0.706		0.003
AI R - CPu L	sex:time	1	16	1.3	0.271		0.025
AI R - CPu L	treatmt:time	1	16	0.232	0.636		0.002
AI R - CPu L	sex:treatmt:time	1	16	0.062	0.806		0.000633
AI R - CPu R	sex	1	16	1.75	0.205		0.032
AI R - CPu R	treatmt	1	16	0.00088	0.977		1.59E-05
AI R - CPu R	time	1	16	8.927	0.009	*	0.098
AI R - CPu R	sex:treatmt	1	16	0.362	0.556		0.007
AI R - CPu R	sex:time	1	16	0.132	0.721		0.002
AI R - CPu R	treatmt:time	1	16	0.074	0.789		0.001
AI R - CPu R	sex:treatmt:time	1	16	0.486	0.496		0.007
AI R - GIDI L	sex	1	16	0.253	0.622		0.006
AI R - GIDI L	treatmt	1	16	0.268	0.612		0.005
AI R - GIDI L	time	1	16	2.975	0.104		0.031
AI R - GIDI L	sex:treatmt	1	16	0.2	0.66		0.004
AI R - GIDI L	sex:time	1	16	2.354	0.144		0.025
AI R - GIDI L	treatmt:time	1	16	0.913	0.354		0.006
AI R - GIDI L	sex:treatmt:time	1	16	2.85	0.111		0.019
AI R - GIDI R	sex	1	16	0.03	0.865		0.000479
AI R - GIDI R	treatmt	1	16	0.153	0.701		0.004
AI R - GIDI R	time	1	16	1.507	0.237		0.018
AI R - GIDI R	sex:treatmt	1	16	0.386	0.543		0.009
AI R - GIDI R	sex:time	1	16	2.383	0.142		0.028
AI R - GIDI R	treatmt:time	1	16	0.195	0.664		0.002
AI R - GIDI R	sex:treatmt:time	1	16	4.17	0.058		0.045
AI R - IL L	sex	1	16	4.108	0.06		0.05
AI R - IL L	treatmt	1	16	1.468	0.243		0.023
AI R - IL L	time	1	16	2.193	0.158		0.042
AI R - IL L	sex:treatmt	1	16	3.14	0.095		0.047
AI R - IL L	sex:time	1	16	2.856	0.11		0.055
AI R - IL L	treatmt:time	1	16	0.389	0.541		0.005

AI R - IL L	sex:treatmt:time	1	16	0.466	0.505		0.006
AI R - IL R	sex	1	16	3.923	0.065		0.066
AI R - IL R	treatmt	1	16	0.527	0.478		0.009
AI R - IL R	time	1	16	1.527	0.234		0.015
AI R - IL R	sex:treatmt	1	16	1.455	0.245		0.024
AI R - IL R	sex:time	1	16	3.086	0.098		0.031
AI R - IL R	treatmt:time	1	16	1.533	0.234		0.026
AI R - IL R	sex:treatmt:time	1	16	6.98E-05	0.993		1.20E-06
AI R - M1 L	sex	1	16	1.762	0.203		0.039
AI R - M1 L	treatmt	1	16	0.188	0.67		0.004
AI R - M1 L	time	1	16	5.376	0.034	*	0.06
AI R - M1 L	sex:treatmt	1	16	0.395	0.538		0.008
AI R - M1 L	sex:time	1	16	3.006	0.102		0.035
AI R - M1 L	treatmt:time	1	16	0.168	0.688		0.001
AI R - M1 L	sex:treatmt:time	1	16	1.73	0.207		0.014
AI R - M1 R	sex	1	16	1.182	0.293		0.029
AI R - M1 R	treatmt	1	16	0.147	0.706		0.002
AI R - M1 R	time	1	16	9.317	0.008	*	0.087
AI R - M1 R	sex:treatmt	1	16	1.225	0.285		0.02
AI R - M1 R	sex:time	1	16	0.221	0.644		0.002
AI R - M1 R	treatmt:time	1	16	0.009	0.927		8.89E-05
AI R - M1 R	sex:treatmt:time	1	16	0.072	0.791		0.000739
AI R - M2 L	sex	1	16	0.624	0.441		0.017
AI R - M2 L	treatmt	1	16	0.103	0.753		0.001
AI R - M2 L	time	1	16	3.141	0.095		0.037
AI R - M2 L	sex:treatmt	1	16	4.633	0.047	*	0.061
AI R - M2 L	sex:time	1	16	0.762	0.396		0.009
AI R - M2 L	treatmt:time	1	16	0.008	0.929		6.69E-05
AI R - M2 L	sex:treatmt:time	1	16	2.967	0.104		0.024
AI R - M2 R	sex	1	16	1.633	0.219		0.034
AI R - M2 R	treatmt	1	16	0.346	0.565		0.005
AI R - M2 R	time	1	16	9.806	0.006	*	0.082
AI R - M2 R	sex:treatmt	1	16	0.572	0.46		0.009
AI R - M2 R	sex:time	1	16	0.338	0.569		0.003
AI R - M2 R	treatmt:time	1	16	0.112	0.742		0.002
AI R - M2 R	sex:treatmt:time	1	16	1.416	0.251		0.022
AI R - NAcC L	sex	1	16	2.011	0.175		0.039
AI R - NAcC L	treatmt	1	16	0.00091	0.976		1.31E-05
AI R - NAcC L	time	1	16	3.419	0.083		0.055
AI R - NAcC L	sex:treatmt	1	16	0.953	0.344		0.014
AI R - NAcC L	sex:time	1	16	4.482	0.05		0.071
AI R - NAcC L	treatmt:time	1	16	2.03	0.173		0.022
AI R - NAcC L	sex:treatmt:time	1	16	0.538	0.474		0.006
AI R - NAcC R	sex	1	16	3.214	0.092		0.067
AI R - NAcC R	treatmt	1	16	0.801	0.384		0.012
AI R - NAcC R	time	1	16	2.875	0.109		0.042
AI R - NAcC R	sex:treatmt	1	16	0.038	0.847		0.000572
AI R - NAcC R	sex:time	1	16	1.954	0.181		0.029
AI R - NAcC R	treatmt:time	1	16	0.462	0.507		0.005
AI R - NAcC R	sex:treatmt:time	1	16	0.328	0.575		0.003
AI R - NAcSh L	sex	1	16	2.429	0.139		0.032
AI R - NAcSh L	treatmt	1	16	0.587	0.455		0.012
AI R - NAcSh L	time	1	16	7.007	0.018	*	0.093
AI R - NAcSh L	sex:treatmt	1	16	6.174	0.024	*	0.11
AI R - NAcSh L	sex:time	1	16	1.92	0.185		0.027
AI R - NAcSh L	treatmt:time	1	16	1.929	0.184		0.027
AI R - NAcSh L	sex:treatmt:time	1	16	0.024	0.878		0.000347
AI R - NAcSh R	sex	1	16	2.124	0.164		0.028
AI R - NAcSh R	treatmt	1	16	0.061	0.809		0.001
AI R - NAcSh R	time	1	16	12.635	0.003	*	0.138
AI R - NAcSh R	sex:treatmt	1	16	2.355	0.144		0.049
AI R - NAcSh R	sex:time	1	16	0.175	0.681		0.002
AI R - NAcSh R	treatmt:time	1	16	0.011	0.918		0.000154

AI R - NAcSh R	sex:treatmt:time	1	16	0.014	0.907		2.00E-04
AI R - PrL L	sex	1	16	0.769	0.394		0.015
AI R - PrL L	treatmt	1	16	1.175	0.294		0.019
AI R - PrL L	time	1	16	4.572	0.048	*	0.058
AI R - PrL L	sex:treatmt	1	16	1.208	0.288		0.02
AI R - PrL L	sex:time	1	16	0.289	0.598		0.004
AI R - PrL L	treatmt:time	1	16	1.468	0.243		0.018
AI R - PrL L	sex:treatmt:time	1	16	0.013	0.909		0.000165
AI R - PrL R	sex	1	16	0.872	0.364		0.019
AI R - PrL R	treatmt	1	16	0.014	0.906		0.00028
AI R - PrL R	time	1	16	0.242	0.629		0.002
AI R - PrL R	sex:treatmt	1	16	1.66	0.216		0.031
AI R - PrL R	sex:time	1	16	2.981	0.103		0.027
AI R - PrL R	treatmt:time	1	16	0.448	0.513		0.006
AI R - PrL R	sex:treatmt:time	1	16	1.234	0.283		0.015
AI R - S1 L	sex	1	16	0.551	0.469		0.013
AI R - S1 L	treatmt	1	16	0.027	0.871		0.000684
AI R - S1 L	time	1	16	5.434	0.033	*	0.034
AI R - S1 L	sex:treatmt	1	16	0.026	0.875		0.000644
AI R - S1 L	sex:time	1	16	0.638	0.436		0.004
AI R - S1 L	treatmt:time	1	16	1.785	0.2		0.011
AI R - S1 L	sex:treatmt:time	1	16	1.503	0.238		0.009
AI R - S1 R	sex	1	16	0.971	0.339		0.017
AI R - S1 R	treatmt	1	16	0.018	0.895		0.000464
AI R - S1 R	time	1	16	2.69	0.121		0.03
AI R - S1 R	sex:treatmt	1	16	0.372	0.55		0.009
AI R - S1 R	sex:time	1	16	0.021	0.885		0.000247
AI R - S1 R	treatmt:time	1	16	0.288	0.599		0.002
AI R - S1 R	sex:treatmt:time	1	16	0.54	0.473		0.004
Cg1 L - AI L	sex	1	16	0.00023	0.988		5.51E-06
Cg1 L - AI L	treatmt	1	16	0.316	0.582		0.005
Cg1 L - AI L	time	1	16	2.551	0.13		0.022
Cg1 L - AI L	sex:treatmt	1	16	0.463	0.506		0.008
Cg1 L - AI L	sex:time	1	16	1.319	0.268		0.012
Cg1 L - AI L	treatmt:time	1	16	0.435	0.519		0.005
Cg1 L - AI L	sex:treatmt:time	1	16	0.337	0.57		0.004
Cg1 L - GIDI L	sex	1	16	0.003	0.956		6.08E-05
Cg1 L - GIDI L	treatmt	1	16	0.559	0.465		0.012
Cg1 L - GIDI L	time	1	16	0.131	0.722		0.001
Cg1 L - GIDI L	sex:treatmt	1	16	0.196	0.664		0.004
Cg1 L - GIDI L	sex:time	1	16	0.047	0.832		0.000418
Cg1 L - GIDI L	treatmt:time	1	16	1.469	0.243		0.017
Cg1 L - GIDI L	sex:treatmt:time	1	16	2.125	0.164		0.024
Cg1 L - M1 L	sex	1	16	0.046	0.833		0.000844
Cg1 L - M1 L	treatmt	1	16	0.949	0.345		0.03
Cg1 L - M1 L	time	1	16	0.741	0.402		0.004
Cg1 L - M1 L	sex:treatmt	1	16	0.009	0.926		0.000283
Cg1 L - M1 L	sex:time	1	16	1.229	0.284		0.007
Cg1 L - M1 L	treatmt:time	1	16	2.17	0.16		0.013
Cg1 L - M1 L	sex:treatmt:time	1	16	0.266	0.613		0.002
Cg1 L - M2 L	sex	1	16	1.058	0.319		0.022
Cg1 L - M2 L	treatmt	1	16	2.74	0.117		0.045
Cg1 L - M2 L	time	1	16	1.061	0.318		0.014
Cg1 L - M2 L	sex:treatmt	1	16	1.638	0.219		0.027
Cg1 L - M2 L	sex:time	1	16	0.381	0.546		0.005
Cg1 L - M2 L	treatmt:time	1	16	0.358	0.558		0.004
Cg1 L - M2 L	sex:treatmt:time	1	16	0.046	0.832		0.000477
Cg1 L - S1 L	sex	1	16	0.061	0.808		0.001
Cg1 L - S1 L	treatmt	1	16	0.578	0.458		0.014
Cg1 L - S1 L	time	1	16	0.186	0.672		0.002
Cg1 L - S1 L	sex:treatmt	1	16	0.753	0.398		0.018
Cg1 L - S1 L	sex:time	1	16	0.954	0.343		0.008
Cg1 L - S1 L	treatmt:time	1	16	1.505	0.238		0.015

Cg1 L - S1 L	sex:treatmt:time	1	16	4.331	0.054		0.041
Cg1 R - AI L	sex	1	16	2.008	0.176		0.044
Cg1 R - AI L	treatmt	1	16	2.232	0.155		0.033
Cg1 R - AI L	time	1	16	2.775	0.115		0.033
Cg1 R - AI L	sex:treatmt	1	16	0.114	0.74		0.002
Cg1 R - AI L	sex:time	1	16	1.263	0.278		0.015
Cg1 R - AI L	treatmt:time	1	16	2.226	0.155		0.026
Cg1 R - AI L	sex:treatmt:time	1	16	1.901	0.187		0.022
Cg1 R - Cg1 L	sex	1	16	0.06	0.81		0.002
Cg1 R - Cg1 L	treatmt	1	16	8.811	0.009	*	0.102
Cg1 R - Cg1 L	time	1	16	8.75	0.009	*	0.13
Cg1 R - Cg1 L	sex:treatmt	1	16	1.67	0.215		0.021
Cg1 R - Cg1 L	sex:time	1	16	0.809	0.382		0.014
Cg1 R - Cg1 L	treatmt:time	1	16	1.628	0.22		0.012
Cg1 R - Cg1 L	sex:treatmt:time	1	16	12.56	0.003	*	0.083
Cg1 R - CPu L	sex	1	16	2.004	0.176		0.037
Cg1 R - CPu L	treatmt	1	16	2.457	0.137		0.047
Cg1 R - CPu L	time	1	16	0.26	0.617		0.004
Cg1 R - CPu L	sex:treatmt	1	16	0.003	0.955		6.47E-05
Cg1 R - CPu L	sex:time	1	16	0.115	0.739		0.002
Cg1 R - CPu L	treatmt:time	1	16	0.352	0.561		0.003
Cg1 R - CPu L	sex:treatmt:time	1	16	2.077	0.169		0.02
Cg1 R - CPu R	sex	1	16	6.856	0.019	*	0.104
Cg1 R - CPu R	treatmt	1	16	0.353	0.561		0.007
Cg1 R - CPu R	time	1	16	0.11	0.744		0.002
Cg1 R - CPu R	sex:treatmt	1	16	1.235	0.283		0.025
Cg1 R - CPu R	sex:time	1	16	0.023	0.882		0.000372
Cg1 R - CPu R	treatmt:time	1	16	0.084	0.776		0.00073
Cg1 R - CPu R	sex:treatmt:time	1	16	0.12	0.733		0.001
Cg1 R - GIDI L	sex	1	16	0.571	0.461		0.013
Cg1 R - GIDI L	treatmt	1	16	1.2	0.289		0.028
Cg1 R - GIDI L	time	1	16	2.23E-05	0.996		2.49E-07
Cg1 R - GIDI L	sex:treatmt	1	16	0.134	0.719		0.003
Cg1 R - GIDI L	sex:time	1	16	0.029	0.867		0.000323
Cg1 R - GIDI L	treatmt:time	1	16	6.616	0.02	*	0.025
Cg1 R - GIDI L	sex:treatmt:time	1	16	5.173	0.037	*	0.019
Cg1 R - IL L	sex	1	16	0.588	0.454		0.016
Cg1 R - IL L	treatmt	1	16	1.623	0.221		0.035
Cg1 R - IL L	time	1	16	1.549	0.231		0.009
Cg1 R - IL L	sex:treatmt	1	16	0.218	0.647		0.005
Cg1 R - IL L	sex:time	1	16	0.16	0.694		0.000895
Cg1 R - IL L	treatmt:time	1	16	4.521	0.049	*	0.03
Cg1 R - IL L	sex:treatmt:time	1	16	0.399	0.537		0.003
Cg1 R - IL R	sex	1	16	2.396	0.141		0.068
Cg1 R - IL R	treatmt	1	16	2.821	0.112		0.039
Cg1 R - IL R	time	1	16	6.328	0.023	*	0.05
Cg1 R - IL R	sex:treatmt	1	16	0.322	0.578		0.005
Cg1 R - IL R	sex:time	1	16	0.206	0.656		0.002
Cg1 R - IL R	treatmt:time	1	16	6.029	0.026	*	0.054
Cg1 R - IL R	sex:treatmt:time	1	16	0.541	0.473		0.005
Cg1 R - M1 L	sex	1	16	2.27	0.151		0.049
Cg1 R - M1 L	treatmt	1	16	1.166	0.296		0.021
Cg1 R - M1 L	time	1	16	0.772	0.393		0.011
Cg1 R - M1 L	sex:treatmt	1	16	1.512	0.237		0.028
Cg1 R - M1 L	sex:time	1	16	0.205	0.657		0.003
Cg1 R - M1 L	treatmt:time	1	16	0.127	0.726		0.000798
Cg1 R - M1 L	sex:treatmt:time	1	16	0.425	0.524		0.003
Cg1 R - M2 L	sex	1	16	0.554	0.468		0.015
Cg1 R - M2 L	treatmt	1	16	3.148	0.095		0.028
Cg1 R - M2 L	time	1	16	0.933	0.349		0.017
Cg1 R - M2 L	sex:treatmt	1	16	13.773	0.002	*	0.112
Cg1 R - M2 L	sex:time	1	16	0.609	0.447		0.011
Cg1 R - M2 L	treatmt:time	1	16	1.022	0.327		0.008

Cg1 R - M2 L	sex:treatmt:time	1	16	1.432	0.249		0.011
Cg1 R - NAcC L	sex	1	16	0.079	0.782		0.002
Cg1 R - NAcC L	treatmt	1	16	6.141	0.025	*	0.078
Cg1 R - NAcC L	time	1	16	3.104	0.097		0.027
Cg1 R - NAcC L	sex:treatmt	1	16	2.674	0.122		0.035
Cg1 R - NAcC L	sex:time	1	16	1.425	0.25		0.013
Cg1 R - NAcC L	treatmt:time	1	16	0.666	0.427		0.006
Cg1 R - NAcC L	sex:treatmt:time	1	16	0.943	0.346		0.009
Cg1 R - NAcC R	sex	1	16	1.796	0.199		0.043
Cg1 R - NAcC R	treatmt	1	16	0.133	0.72		0.002
Cg1 R - NAcC R	time	1	16	0.048	0.83		0.00066
Cg1 R - NAcC R	sex:treatmt	1	16	0.031	0.861		0.000413
Cg1 R - NAcC R	sex:time	1	16	0.267	0.612		0.004
Cg1 R - NAcC R	treatmt:time	1	16	0.235	0.634		0.002
Cg1 R - NAcC R	sex:treatmt:time	1	16	1.553	0.231		0.016
Cg1 R - NAcSh L	sex	1	16	2.186	0.159		0.042
Cg1 R - NAcSh L	treatmt	1	16	0.185	0.673		0.003
Cg1 R - NAcSh L	time	1	16	5.704	0.03	*	0.087
Cg1 R - NAcSh L	sex:treatmt	1	16	1.696	0.211		0.028
Cg1 R - NAcSh L	sex:time	1	16	2.366	0.144		0.038
Cg1 R - NAcSh L	treatmt:time	1	16	0.046	0.832		0.00041
Cg1 R - NAcSh L	sex:treatmt:time	1	16	3.06	0.099		0.026
Cg1 R - NAcSh R	sex	1	16	1.261	0.278		0.027
Cg1 R - NAcSh R	treatmt	1	16	1.038	0.323		0.017
Cg1 R - NAcSh R	time	1	16	0.325	0.577		0.004
Cg1 R - NAcSh R	sex:treatmt	1	16	0.142	0.711		0.002
Cg1 R - NAcSh R	sex:time	1	16	1.916	0.185		0.024
Cg1 R - NAcSh R	treatmt:time	1	16	0.046	0.834		0.000471
Cg1 R - NAcSh R	sex:treatmt:time	1	16	3.419	0.083		0.034
Cg1 R - PrL L	sex	1	16	2.088	0.168		0.039
Cg1 R - PrL L	treatmt	1	16	0.00039	0.984		7.86E-06
Cg1 R - PrL L	time	1	16	6.367	0.023	*	0.089
Cg1 R - PrL L	sex:treatmt	1	16	0.02	0.89		0.000393
Cg1 R - PrL L	sex:time	1	16	2.194	0.158		0.033
Cg1 R - PrL L	treatmt:time	1	16	4.878	0.042	*	0.037
Cg1 R - PrL L	sex:treatmt:time	1	16	0.545	0.471		0.004
Cg1 R - PrL R	sex	1	16	0.947	0.345		0.024
Cg1 R - PrL R	treatmt	1	16	0.001	0.97		2.50E-05
Cg1 R - PrL R	time	1	16	1.975	0.179		0.023
Cg1 R - PrL R	sex:treatmt	1	16	0.062	0.807		0.001
Cg1 R - PrL R	sex:time	1	16	0.554	0.467		0.006
Cg1 R - PrL R	treatmt:time	1	16	6.397	0.022	*	0.051
Cg1 R - PrL R	sex:treatmt:time	1	16	0.91	0.354		0.008
Cg1 R - S1 L	sex	1	16	1.756	0.204		0.049
Cg1 R - S1 L	treatmt	1	16	0.829	0.376		0.015
Cg1 R - S1 L	time	1	16	0.183	0.675		0.002
Cg1 R - S1 L	sex:treatmt	1	16	0.069	0.796		0.001
Cg1 R - S1 L	sex:time	1	16	1.239	0.282		0.011
Cg1 R - S1 L	treatmt:time	1	16	2.308	0.148		0.011
Cg1 R - S1 L	sex:treatmt:time	1	16	5.106	0.038	*	0.024
CPu L - AI L	sex	1	16	0.055	0.817		0.001
CPu L - AI L	treatmt	1	16	1.697	0.211		0.03
CPu L - AI L	time	1	16	10.848	0.005	*	0.121
CPu L - AI L	sex:treatmt	1	16	0.673	0.424		0.012
CPu L - AI L	sex:time	1	16	0.123	0.731		0.002
CPu L - AI L	treatmt:time	1	16	0.00011	0.992		8.45E-07
CPu L - AI L	sex:treatmt:time	1	16	0.506	0.487		0.004
CPu L - Cg1 L	sex	1	16	0.044	0.836		0.000896
CPu L - Cg1 L	treatmt	1	16	2.681	0.121		0.056
CPu L - Cg1 L	time	1	16	0.515	0.483		0.006
CPu L - Cg1 L	sex:treatmt	1	16	0.424	0.524		0.009
CPu L - Cg1 L	sex:time	1	16	0.632	0.438		0.008
CPu L - Cg1 L	treatmt:time	1	16	0.518	0.482		0.004

CPu L - Cg1 L	sex:treatmt:time	1	16	2.715	0.119		0.02
CPu L - GIDI L	sex	1	16	0.009	0.927		0.000157
CPu L - GIDI L	treatmt	1	16	2.162	0.161		0.032
CPu L - GIDI L	time	1	16	1.186	0.292		0.022
CPu L - GIDI L	sex:treatmt	1	16	0.006	0.94		8.98E-05
CPu L - GIDI L	sex:time	1	16	3.418	0.083		0.06
CPu L - GIDI L	treatmt:time	1	16	0.063	0.805		0.000637
CPu L - GIDI L	sex:treatmt:time	1	16	0.371	0.551		0.004
CPu L - IL L	sex	1	16	0.041	0.842		0.000464
CPu L - IL L	treatmt	1	16	6.388	0.022	*	0.12
CPu L - IL L	time	1	16	0.505	0.487		0.009
CPu L - IL L	sex:treatmt	1	16	0.842	0.372		0.018
CPu L - IL L	sex:time	1	16	0.224	0.642		0.004
CPu L - IL L	treatmt:time	1	16	0.19	0.669		0.002
CPu L - IL L	sex:treatmt:time	1	16	0.031	0.863		0.000358
CPu L - M1 L	sex	1	16	0.193	0.666		0.005
CPu L - M1 L	treatmt	1	16	6.341	0.023	*	0.076
CPu L - M1 L	time	1	16	0.005	0.944		6.77E-05
CPu L - M1 L	sex:treatmt	1	16	0.096	0.761		0.001
CPu L - M1 L	sex:time	1	16	2.751	0.117		0.035
CPu L - M1 L	treatmt:time	1	16	0.485	0.496		0.006
CPu L - M1 L	sex:treatmt:time	1	16	0.614	0.445		0.008
CPu L - M2 L	sex	1	16	0.231	0.637		0.004
CPu L - M2 L	treatmt	1	16	0.003	0.961		4.67E-05
CPu L - M2 L	time	1	16	0.004	0.953		4.68E-05
CPu L - M2 L	sex:treatmt	1	16	0.045	0.836		0.000828
CPu L - M2 L	sex:time	1	16	0.307	0.587		0.004
CPu L - M2 L	treatmt:time	1	16	0.419	0.527		0.005
CPu L - M2 L	sex:treatmt:time	1	16	0.372	0.55		0.005
CPu L - PrL L	sex	1	16	0.01	0.92		0.000235
CPu L - PrL L	treatmt	1	16	1.011	0.33		0.023
CPu L - PrL L	time	1	16	0.645	0.434		0.007
CPu L - PrL L	sex:treatmt	1	16	0.522	0.481		0.012
CPu L - PrL L	sex:time	1	16	1.528	0.234		0.016
CPu L - PrL L	treatmt:time	1	16	0.024	0.878		0.00014
CPu L - PrL L	sex:treatmt:time	1	16	0.028	0.87		0.000161
CPu L - S1 L	sex	1	16	0.06	0.81		0.002
CPu L - S1 L	treatmt	1	16	5.52	0.032	*	0.04
CPu L - S1 L	time	1	16	0.241	0.63		0.003
CPu L - S1 L	sex:treatmt	1	16	1.131	0.303		0.008
CPu L - S1 L	sex:time	1	16	1.356	0.261		0.018
CPu L - S1 L	treatmt:time	1	16	0.065	0.802		0.000674
CPu L - S1 L	sex:treatmt:time	1	16	1.122	0.305		0.012
CPu R - AI L	sex	1	16	0.201	0.66		0.005
CPu R - AI L	treatmt	1	16	0.003	0.957		4.77E-05
CPu R - AI L	time	1	16	10.794	0.005	*	0.107
CPu R - AI L	sex:treatmt	1	16	0.003	0.958		4.55E-05
CPu R - AI L	sex:time	1	16	0.034	0.856		0.000379
CPu R - AI L	treatmt:time	1	16	0.009	0.926		9.30E-05
CPu R - AI L	sex:treatmt:time	1	16	1.774	0.202		0.018
CPu R - Cg1 L	sex	1	16	0.42	0.526		0.008
CPu R - Cg1 L	treatmt	1	16	2.482	0.135		0.053
CPu R - Cg1 L	time	1	16	6.74E-08	1		8.65E-10
CPu R - Cg1 L	sex:treatmt	1	16	0.061	0.808		0.001
CPu R - Cg1 L	sex:time	1	16	1.078	0.315		0.014
CPu R - Cg1 L	treatmt:time	1	16	1.4	0.254		0.01
CPu R - Cg1 L	sex:treatmt:time	1	16	0.114	0.74		0.000827
CPu R - CPu L	sex	1	16	0.146	0.708		0.005
CPu R - CPu L	treatmt	1	16	2.775	0.115		0.028
CPu R - CPu L	time	1	16	0.004	0.947		5.36E-05
CPu R - CPu L	sex:treatmt	1	16	0.353	0.561		0.004
CPu R - CPu L	sex:time	1	16	0.869	0.365		0.01
CPu R - CPu L	treatmt:time	1	16	0.00019	0.989		1.73E-06

CPu R - CPu L	sex:treatmt:time	1	16	0.198	0.662		0.002
CPu R - GIDI L	sex	1	16	0.619	0.443		0.011
CPu R - GIDI L	treatmt	1	16	0.005	0.943		0.000123
CPu R - GIDI L	time	1	16	7.135	0.017	*	0.106
CPu R - GIDI L	sex:treatmt	1	16	1.095	0.311		0.025
CPu R - GIDI L	sex:time	1	16	4.462	0.051		0.069
CPu R - GIDI L	treatmt:time	1	16	2.135	0.163		0.009
CPu R - GIDI L	sex:treatmt:time	1	16	0.517	0.482		0.002
CPu R - IL L	sex	1	16	0.915	0.353		0.014
CPu R - IL L	treatmt	1	16	1.359	0.261		0.029
CPu R - IL L	time	1	16	1.755	0.204		0.021
CPu R - IL L	sex:treatmt	1	16	2.596	0.127		0.054
CPu R - IL L	sex:time	1	16	0.859	0.368		0.01
CPu R - IL L	treatmt:time	1	16	0.01	0.92		0.000133
CPu R - IL L	sex:treatmt:time	1	16	0.007	0.936		8.63E-05
CPu R - M1 L	sex	1	16	0.081	0.78		0.002
CPu R - M1 L	treatmt	1	16	4.458	0.051		0.054
CPu R - M1 L	time	1	16	0.17	0.685		0.003
CPu R - M1 L	sex:treatmt	1	16	1.679	0.213		0.021
CPu R - M1 L	sex:time	1	16	1.47	0.243		0.028
CPu R - M1 L	treatmt:time	1	16	0.12	0.733		0.000711
CPu R - M1 L	sex:treatmt:time	1	16	0.066	0.8		0.00039
CPu R - M2 L	sex	1	16	0.016	0.902		0.000342
CPu R - M2 L	treatmt	1	16	0.01	0.923		0.000146
CPu R - M2 L	time	1	16	0.072	0.791		0.000831
CPu R - M2 L	sex:treatmt	1	16	0.034	0.857		0.000508
CPu R - M2 L	sex:time	1	16	0.275	0.607		0.003
CPu R - M2 L	treatmt:time	1	16	0.004	0.95		5.69E-05
CPu R - M2 L	sex:treatmt:time	1	16	0.087	0.771		0.001
CPu R - NAcC L	sex	1	16	0.807	0.382		0.025
CPu R - NAcC L	treatmt	1	16	9.813	0.006	*	0.064
CPu R - NAcC L	time	1	16	0.592	0.453		0.005
CPu R - NAcC L	sex:treatmt	1	16	2.379	0.142		0.016
CPu R - NAcC L	sex:time	1	16	6.555	0.021	*	0.054
CPu R - NAcC L	treatmt:time	1	16	0.009	0.925		0.000131
CPu R - NAcC L	sex:treatmt:time	1	16	0.163	0.692		0.002
CPu R - NAcC R	sex	1	16	6.093	0.025	*	0.101
CPu R - NAcC R	treatmt	1	16	0.376	0.549		0.007
CPu R - NAcC R	time	1	16	2.068	0.17		0.031
CPu R - NAcC R	sex:treatmt	1	16	3.659	0.074		0.067
CPu R - NAcC R	sex:time	1	16	0.015	0.904		0.000229
CPu R - NAcC R	treatmt:time	1	16	4.806	0.043	*	0.043
CPu R - NAcC R	sex:treatmt:time	1	16	0.001	0.973		1.11E-05
CPu R - NAcSh L	sex	1	16	1.72	0.208		0.052
CPu R - NAcSh L	treatmt	1	16	0.67	0.425		0.013
CPu R - NAcSh L	time	1	16	5.879	0.028	*	0.031
CPu R - NAcSh L	sex:treatmt	1	16	0.149	0.705		0.003
CPu R - NAcSh L	sex:time	1	16	0.025	0.877		0.000136
CPu R - NAcSh L	treatmt:time	1	16	5.434	0.033	*	0.031
CPu R - NAcSh L	sex:treatmt:time	1	16	0.009	0.925		5.49E-05
CPu R - NAcSh R	sex	1	16	0.517	0.482		0.013
CPu R - NAcSh R	treatmt	1	16	2.094	0.167		0.046
CPu R - NAcSh R	time	1	16	17.299	0.00074	*	0.121
CPu R - NAcSh R	sex:treatmt	1	16	0.014	0.907		0.000329
CPu R - NAcSh R	sex:time	1	16	0.777	0.391		0.006
CPu R - NAcSh R	treatmt:time	1	16	6.235	0.024	*	0.037
CPu R - NAcSh R	sex:treatmt:time	1	16	1.604	0.223		0.01
CPu R - PrL L	sex	1	16	0.201	0.66		0.005
CPu R - PrL L	treatmt	1	16	0.053	0.821		0.001
CPu R - PrL L	time	1	16	1.058	0.319		0.012
CPu R - PrL L	sex:treatmt	1	16	0.098	0.758		0.002
CPu R - PrL L	sex:time	1	16	0.261	0.616		0.003
CPu R - PrL L	treatmt:time	1	16	0.007	0.937		3.62E-05

CPu R - PrL L	sex:treatmt:time	1	16	0.305	0.589		0.002
CPu R - S1 L	sex	1	16	0.172	0.684		0.004
CPu R - S1 L	treatmt	1	16	0.014	0.907		0.000174
CPu R - S1 L	time	1	16	3.901	0.066		0.07
CPu R - S1 L	sex:treatmt	1	16	0.565	0.463		0.007
CPu R - S1 L	sex:time	1	16	3.479	0.081		0.063
CPu R - S1 L	treatmt:time	1	16	1.761	0.203		0.015
CPu R - S1 L	sex:treatmt:time	1	16	3.088	0.098		0.026
GIDI L - AI L	sex	1	16	0.009	0.927		0.000224
GIDI L - AI L	treatmt	1	16	0.115	0.739		0.002
GIDI L - AI L	time	1	16	5.401	0.034	*	0.039
GIDI L - AI L	sex:treatmt	1	16	1.193	0.291		0.025
GIDI L - AI L	sex:time	1	16	0.478	0.499		0.004
GIDI L - AI L	treatmt:time	1	16	0.434	0.519		0.003
GIDI L - AI L	sex:treatmt:time	1	16	4.238	0.056		0.032
GIDI R - AI L	sex	1	16	1.386	0.256		0.035
GIDI R - AI L	treatmt	1	16	4.429	0.051		0.065
GIDI R - AI L	time	1	16	3.926	0.065		0.05
GIDI R - AI L	sex:treatmt	1	16	3.229	0.091		0.048
GIDI R - AI L	sex:time	1	16	0.216	0.648		0.003
GIDI R - AI L	treatmt:time	1	16	0.656	0.43		0.005
GIDI R - AI L	sex:treatmt:time	1	16	0.229	0.639		0.002
GIDI R - Cg1 L	sex	1	16	5.568	0.031	*	0.088
GIDI R - Cg1 L	treatmt	1	16	0.184	0.673		0.005
GIDI R - Cg1 L	time	1	16	5.131	0.038	*	0.025
GIDI R - Cg1 L	sex:treatmt	1	16	0.69	0.418		0.02
GIDI R - Cg1 L	sex:time	1	16	5.084	0.039	*	0.025
GIDI R - Cg1 L	treatmt:time	1	16	1.218	0.286		0.012
GIDI R - Cg1 L	sex:treatmt:time	1	16	1.55	0.231		0.016
GIDI R - Cg1 R	sex	1	16	0.829	0.376		0.013
GIDI R - Cg1 R	treatmt	1	16	0.362	0.556		0.01
GIDI R - Cg1 R	time	1	16	0.243	0.629		0.002
GIDI R - Cg1 R	sex:treatmt	1	16	0.299	0.592		0.008
GIDI R - Cg1 R	sex:time	1	16	3.459	0.081		0.027
GIDI R - Cg1 R	treatmt:time	1	16	2.158	0.161		0.022
GIDI R - Cg1 R	sex:treatmt:time	1	16	0.774	0.392		0.008
GIDI R - CPu L	sex	1	16	2.41	0.14		0.06
GIDI R - CPu L	treatmt	1	16	0.978	0.338		0.016
GIDI R - CPu L	time	1	16	9.824	0.006	*	0.087
GIDI R - CPu L	sex:treatmt	1	16	0.569	0.461		0.009
GIDI R - CPu L	sex:time	1	16	0.607	0.447		0.006
GIDI R - CPu L	treatmt:time	1	16	1.796	0.199		0.018
GIDI R - CPu L	sex:treatmt:time	1	16	0.539	0.473		0.005
GIDI R - CPu R	sex	1	16	0.184	0.674		0.005
GIDI R - CPu R	treatmt	1	16	2.755	0.116		0.041
GIDI R - CPu R	time	1	16	6.954	0.018	*	0.062
GIDI R - CPu R	sex:treatmt	1	16	0.179	0.678		0.003
GIDI R - CPu R	sex:time	1	16	0.394	0.539		0.004
GIDI R - CPu R	treatmt:time	1	16	0.532	0.476		0.004
GIDI R - CPu R	sex:treatmt:time	1	16	0.073	0.79		0.000615
GIDI R - GIDI L	sex	1	16	1.929	0.184		0.061
GIDI R - GIDI L	treatmt	1	16	0.291	0.597		0.004
GIDI R - GIDI L	time	1	16	1.979	0.179		0.019
GIDI R - GIDI L	sex:treatmt	1	16	1.761	0.203		0.021
GIDI R - GIDI L	sex:time	1	16	1.987	0.178		0.019
GIDI R - GIDI L	treatmt:time	1	16	0.057	0.814		0.00038
GIDI R - GIDI L	sex:treatmt:time	1	16	0.229	0.639		0.002
GIDI R - IL L	sex	1	16	0.209	0.654		0.004
GIDI R - IL L	treatmt	1	16	2.557	0.129		0.052
GIDI R - IL L	time	1	16	1.849	0.193		0.022
GIDI R - IL L	sex:treatmt	1	16	3.703	0.072		0.074
GIDI R - IL L	sex:time	1	16	0.233	0.636		0.003
GIDI R - IL L	treatmt:time	1	16	0.268	0.612		0.003

GIDI R - IL L	sex:treatmt:time	1	16	1.001	0.332		0.01
GIDI R - IL R	sex	1	16	0.012	0.915		0.000185
GIDI R - IL R	treatmt	1	16	2.227	0.155		0.047
GIDI R - IL R	time	1	16	1.153	0.299		0.009
GIDI R - IL R	sex:treatmt	1	16	4.591	0.048	*	0.093
GIDI R - IL R	sex:time	1	16	0.039	0.845		0.000318
GIDI R - IL R	treatmt:time	1	16	0.00016	0.99		2.62E-06
GIDI R - IL R	sex:treatmt:time	1	16	0.485	0.496		0.008
GIDI R - M1 L	sex	1	16	0.023	0.881		0.000744
GIDI R - M1 L	treatmt	1	16	12.192	0.003	*	0.106
GIDI R - M1 L	time	1	16	4.913	0.042	*	0.049
GIDI R - M1 L	sex:treatmt	1	16	0.698	0.416		0.007
GIDI R - M1 L	sex:time	1	16	0.524	0.48		0.006
GIDI R - M1 L	treatmt:time	1	16	0.095	0.762		0.000931
GIDI R - M1 L	sex:treatmt:time	1	16	0.389	0.542		0.004
GIDI R - M1 R	sex	1	16	0.09	0.768		0.002
GIDI R - M1 R	treatmt	1	16	0.14	0.713		0.002
GIDI R - M1 R	time	1	16	1.285	0.274		0.016
GIDI R - M1 R	sex:treatmt	1	16	3.012	0.102		0.045
GIDI R - M1 R	sex:time	1	16	1.143	0.301		0.014
GIDI R - M1 R	treatmt:time	1	16	0.953	0.343		0.015
GIDI R - M1 R	sex:treatmt:time	1	16	0.355	0.56		0.006
GIDI R - M2 L	sex	1	16	0.735	0.404		0.018
GIDI R - M2 L	treatmt	1	16	0.84	0.373		0.017
GIDI R - M2 L	time	1	16	4.917	0.041	*	0.023
GIDI R - M2 L	sex:treatmt	1	16	2.331	0.146		0.045
GIDI R - M2 L	sex:time	1	16	1.144	0.301		0.005
GIDI R - M2 L	treatmt:time	1	16	0.911	0.354		0.011
GIDI R - M2 L	sex:treatmt:time	1	16	0.267	0.612		0.003
GIDI R - M2 R	sex	1	16	0.156	0.698		0.003
GIDI R - M2 R	treatmt	1	16	1.517	0.236		0.031
GIDI R - M2 R	time	1	16	2.051	0.171		0.017
GIDI R - M2 R	sex:treatmt	1	16	2.166	0.16		0.043
GIDI R - M2 R	sex:time	1	16	5.518	0.032	*	0.044
GIDI R - M2 R	treatmt:time	1	16	2.094	0.167		0.03
GIDI R - M2 R	sex:treatmt:time	1	16	0.228	0.639		0.003
GIDI R - NAcC L	sex	1	16	0.597	0.451		0.016
GIDI R - NAcC L	treatmt	1	16	4.232	0.056		0.054
GIDI R - NAcC L	time	1	16	2.655	0.123		0.031
GIDI R - NAcC L	sex:treatmt	1	16	0.044	0.836		0.000601
GIDI R - NAcC L	sex:time	1	16	3.225	0.091		0.038
GIDI R - NAcC L	treatmt:time	1	16	4.811	0.043	*	0.047
GIDI R - NAcC L	sex:treatmt:time	1	16	2.174	0.16		0.022
GIDI R - NAcC R	sex	1	16	0.332	0.573		0.006
GIDI R - NAcC R	treatmt	1	16	3.861	0.067		0.084
GIDI R - NAcC R	time	1	16	3.349	0.086		0.036
GIDI R - NAcC R	sex:treatmt	1	16	1.039	0.323		0.024
GIDI R - NAcC R	sex:time	1	16	0.207	0.655		0.002
GIDI R - NAcC R	treatmt:time	1	16	1.291	0.273		0.013
GIDI R - NAcC R	sex:treatmt:time	1	16	0.12	0.734		0.001
GIDI R - NAcSh L	sex	1	16	0.08	0.78		0.001
GIDI R - NAcSh L	treatmt	1	16	4.261	0.056		0.085
GIDI R - NAcSh L	time	1	16	5.308	0.035	*	0.044
GIDI R - NAcSh L	sex:treatmt	1	16	1.665	0.215		0.035
GIDI R - NAcSh L	sex:time	1	16	0.34	0.568		0.003
GIDI R - NAcSh L	treatmt:time	1	16	0.002	0.967		3.15E-05
GIDI R - NAcSh L	sex:treatmt:time	1	16	0.439	0.517		0.008
GIDI R - NAcSh R	sex	1	16	0.191	0.668		0.002
GIDI R - NAcSh R	treatmt	1	16	2.284	0.15		0.062
GIDI R - NAcSh R	time	1	16	26.987	8.85E-05	*	0.127
GIDI R - NAcSh R	sex:treatmt	1	16	1.288	0.273		0.036
GIDI R - NAcSh R	sex:time	1	16	0.386	0.543		0.002
GIDI R - NAcSh R	treatmt:time	1	16	0.069	0.796		0.001

GIDI R - NAcSh R	sex:treatmt:time	1	16	0.151	0.703		0.003
GIDI R - PrL L	sex	1	16	0.494	0.492		0.011
GIDI R - PrL L	treatmt	1	16	0.904	0.356		0.022
GIDI R - PrL L	time	1	16	2.56	0.129		0.016
GIDI R - PrL L	sex:treatmt	1	16	1.581	0.227		0.038
GIDI R - PrL L	sex:time	1	16	2.338	0.146		0.015
GIDI R - PrL L	treatmt:time	1	16	0.215	0.649		0.002
GIDI R - PrL L	sex:treatmt:time	1	16	0.062	0.807		0.000525
GIDI R - PrL R	sex	1	16	0.061	0.809		0.001
GIDI R - PrL R	treatmt	1	16	1.611	0.222		0.037
GIDI R - PrL R	time	1	16	0.307	0.587		0.003
GIDI R - PrL R	sex:treatmt	1	16	0.867	0.366		0.02
GIDI R - PrL R	sex:time	1	16	0.256	0.62		0.002
GIDI R - PrL R	treatmt:time	1	16	1.627	0.22		0.017
GIDI R - PrL R	sex:treatmt:time	1	16	1.654	0.217		0.017
GIDI R - S1 L	sex	1	16	0.963	0.341		0.025
GIDI R - S1 L	treatmt	1	16	1.339	0.264		0.022
GIDI R - S1 L	time	1	16	0.443	0.515		0.004
GIDI R - S1 L	sex:treatmt	1	16	0.348	0.563		0.006
GIDI R - S1 L	sex:time	1	16	0.058	0.813		0.000568
GIDI R - S1 L	treatmt:time	1	16	0.259	0.618		0.002
GIDI R - S1 L	sex:treatmt:time	1	16	0.069	0.796		0.00064
GIDI R - S1 R	sex	1	16	1.09	0.312		0.015
GIDI R - S1 R	treatmt	1	16	2.193	0.158		0.058
GIDI R - S1 R	time	1	16	0.265	0.614		0.003
GIDI R - S1 R	sex:treatmt	1	16	0.342	0.567		0.01
GIDI R - S1 R	sex:time	1	16	0.748	0.4		0.008
GIDI R - S1 R	treatmt:time	1	16	0.096	0.761		0.000897
GIDI R - S1 R	sex:treatmt:time	1	16	0.24	0.631		0.002
IL L - AI L	sex	1	16	2.45E-06	0.999		5.89E-08
IL L - AI L	treatmt	1	16	1.224	0.285		0.022
IL L - AI L	time	1	16	3.318	0.087		0.034
IL L - AI L	sex:treatmt	1	16	2.115	0.165		0.038
IL L - AI L	sex:time	1	16	0.371	0.551		0.004
IL L - AI L	treatmt:time	1	16	0.397	0.538		0.004
IL L - AI L	sex:treatmt:time	1	16	1.703	0.21		0.016
IL L - Cg1 L	sex	1	16	0.77	0.393		0.02
IL L - Cg1 L	treatmt	1	16	2.539	0.131		0.029
IL L - Cg1 L	time	1	16	1.266	0.277		0.012
IL L - Cg1 L	sex:treatmt	1	16	0.361	0.557		0.004
IL L - Cg1 L	sex:time	1	16	0.101	0.755		0.000982
IL L - Cg1 L	treatmt:time	1	16	2.796	0.114		0.038
IL L - Cg1 L	sex:treatmt:time	1	16	0.139	0.715		0.002
IL L - GIDI L	sex	1	16	0.1	0.756		0.002
IL L - GIDI L	treatmt	1	16	5.247	0.036	*	0.102
IL L - GIDI L	time	1	16	0.928	0.35		0.013
IL L - GIDI L	sex:treatmt	1	16	0.183	0.675		0.004
IL L - GIDI L	sex:time	1	16	0.07	0.794		0.001
IL L - GIDI L	treatmt:time	1	16	0.682	0.421		0.007
IL L - GIDI L	sex:treatmt:time	1	16	0.055	0.818		0.000573
IL L - M1 L	sex	1	16	0.023	0.88		0.00028
IL L - M1 L	treatmt	1	16	2.98	0.104		0.07
IL L - M1 L	time	1	16	0.774	0.392		0.011
IL L - M1 L	sex:treatmt	1	16	0.064	0.804		0.002
IL L - M1 L	sex:time	1	16	0.007	0.933		0.000107
IL L - M1 L	treatmt:time	1	16	0.239	0.632		0.003
IL L - M1 L	sex:treatmt:time	1	16	0.142	0.711		0.002
IL L - M2 L	sex	1	16	1.355	0.261		0.028
IL L - M2 L	treatmt	1	16	0.467	0.504		0.007
IL L - M2 L	time	1	16	1.516	0.236		0.02
IL L - M2 L	sex:treatmt	1	16	0.168	0.687		0.003
IL L - M2 L	sex:time	1	16	0.0006	0.981		8.15E-06
IL L - M2 L	treatmt:time	1	16	0.459	0.508		0.005

IL L - M2 L	sex:treatmt:time	1	16	0.026	0.873		0.000308
IL L - PrL L	sex	1	16	0.364	0.555		0.007
IL L - PrL L	treatmt	1	16	0.912	0.354		0.007
IL L - PrL L	time	1	16	6.041	0.026	*	0.136
IL L - PrL L	sex:treatmt	1	16	1.275	0.276		0.01
IL L - PrL L	sex:time	1	16	0.071	0.793		0.002
IL L - PrL L	treatmt:time	1	16	0.882	0.362		0.007
IL L - PrL L	sex:treatmt:time	1	16	0.592	0.453		0.005
IL L - S1 L	sex	1	16	0.143	0.71		0.002
IL L - S1 L	treatmt	1	16	2.893	0.108		0.08
IL L - S1 L	time	1	16	0.436	0.519		0.005
IL L - S1 L	sex:treatmt	1	16	2.021	0.174		0.057
IL L - S1 L	sex:time	1	16	2.291	0.15		0.024
IL L - S1 L	treatmt:time	1	16	0.954	0.343		0.009
IL L - S1 L	sex:treatmt:time	1	16	3.387	0.084		0.03
IL R - AI L	sex	1	16	0.285	0.601		0.005
IL R - AI L	treatmt	1	16	0.632	0.438		0.01
IL R - AI L	time	1	16	1.269	0.277		0.017
IL R - AI L	sex:treatmt	1	16	3.509	0.079		0.051
IL R - AI L	sex:time	1	16	0.008	0.929		0.000113
IL R - AI L	treatmt:time	1	16	3.651	0.074		0.051
IL R - AI L	sex:treatmt:time	1	16	2.084	0.168		0.03
IL R - Cg1 L	sex	1	16	2.751	0.117		0.064
IL R - Cg1 L	treatmt	1	16	3.264	0.09		0.023
IL R - Cg1 L	time	1	16	1.152	0.299		0.022
IL R - Cg1 L	sex:treatmt	1	16	0.06	0.81		0.000428
IL R - Cg1 L	sex:time	1	16	0.014	0.908		0.00026
IL R - Cg1 L	treatmt:time	1	16	2.906	0.108		0.032
IL R - Cg1 L	sex:treatmt:time	1	16	1.159	0.298		0.013
IL R - CPu L	sex	1	16	0.391	0.541		0.008
IL R - CPu L	treatmt	1	16	4.883	0.042	*	0.062
IL R - CPu L	time	1	16	0.86	0.368		0.015
IL R - CPu L	sex:treatmt	1	16	0.757	0.397		0.01
IL R - CPu L	sex:time	1	16	0.515	0.483		0.009
IL R - CPu L	treatmt:time	1	16	0.413	0.53		0.004
IL R - CPu L	sex:treatmt:time	1	16	0.375	0.549		0.004
IL R - CPu R	sex	1	16	1.702	0.21		0.042
IL R - CPu R	treatmt	1	16	4.186	0.058		0.054
IL R - CPu R	time	1	16	0.147	0.707		0.002
IL R - CPu R	sex:treatmt	1	16	3.837	0.068		0.05
IL R - CPu R	sex:time	1	16	0.493	0.493		0.006
IL R - CPu R	treatmt:time	1	16	0.219	0.646		0.002
IL R - CPu R	sex:treatmt:time	1	16	0.245	0.627		0.003
IL R - GIDI L	sex	1	16	2.683	0.121		0.048
IL R - GIDI L	treatmt	1	16	1.985	0.178		0.037
IL R - GIDI L	time	1	16	0.044	0.837		0.000731
IL R - GIDI L	sex:treatmt	1	16	0.428	0.522		0.008
IL R - GIDI L	sex:time	1	16	2.438	0.138		0.039
IL R - GIDI L	treatmt:time	1	16	9.617	0.007	*	0.068
IL R - GIDI L	sex:treatmt:time	1	16	0.004	0.949		3.19E-05
IL R - IL L	sex	1	16	0.067	0.8		0.000878
IL R - IL L	treatmt	1	16	0.207	0.656		0.004
IL R - IL L	time	1	16	46.581	4.08E-06	*	0.336
IL R - IL L	sex:treatmt	1	16	0.931	0.349		0.018
IL R - IL L	sex:time	1	16	0.203	0.659		0.002
IL R - IL L	treatmt:time	1	16	0.005	0.942		0.000101
IL R - IL L	sex:treatmt:time	1	16	1.69E-05	0.997		3.11E-07
IL R - M1 L	sex	1	16	0.795	0.386		0.015
IL R - M1 L	treatmt	1	16	3.164	0.094		0.055
IL R - M1 L	time	1	16	3.165	0.094		0.039
IL R - M1 L	sex:treatmt	1	16	0.115	0.739		0.002
IL R - M1 L	sex:time	1	16	1.266	0.277		0.016
IL R - M1 L	treatmt:time	1	16	1.622	0.221		0.019

IL R - M1 L	sex:treatmt:time	1	16	0.594	0.452		0.007
IL R - M2 L	sex	1	16	2.087	0.168		0.046
IL R - M2 L	treatmt	1	16	1.542	0.232		0.021
IL R - M2 L	time	1	16	4.372	0.053		0.059
IL R - M2 L	sex:treatmt	1	16	0.026	0.873		0.000373
IL R - M2 L	sex:time	1	16	0.002	0.969		2.29E-05
IL R - M2 L	treatmt:time	1	16	0.582	0.457		0.006
IL R - M2 L	sex:treatmt:time	1	16	0.053	0.82		0.000572
IL R - NAcC L	sex	1	16	0.079	0.782		0.002
IL R - NAcC L	treatmt	1	16	3.613	0.075		0.057
IL R - NAcC L	time	1	16	8.617	0.01	*	0.087
IL R - NAcC L	sex:treatmt	1	16	0.267	0.612		0.004
IL R - NAcC L	sex:time	1	16	0.409	0.531		0.005
IL R - NAcC L	treatmt:time	1	16	0.259	0.618		0.001
IL R - NAcC L	sex:treatmt:time	1	16	1.935	0.183		0.011
IL R - NAcC R	sex	1	16	1.844	0.193		0.063
IL R - NAcC R	treatmt	1	16	18.874	0.0005	*	0.141
IL R - NAcC R	time	1	16	2.904	0.108		0.023
IL R - NAcC R	sex:treatmt	1	16	5.37	0.034	*	0.045
IL R - NAcC R	sex:time	1	16	2.575	0.128		0.02
IL R - NAcC R	treatmt:time	1	16	5.526	0.032	*	0.051
IL R - NAcC R	sex:treatmt:time	1	16	1.876	0.19		0.018
IL R - NAcSh L	sex	1	16	0.832	0.375		0.021
IL R - NAcSh L	treatmt	1	16	0.791	0.387		0.013
IL R - NAcSh L	time	1	16	1.255	0.279		0.011
IL R - NAcSh L	sex:treatmt	1	16	0.511	0.485		0.008
IL R - NAcSh L	sex:time	1	16	0.019	0.891		0.000178
IL R - NAcSh L	treatmt:time	1	16	3.377	0.085		0.038
IL R - NAcSh L	sex:treatmt:time	1	16	0.398	0.537		0.005
IL R - NAcSh R	sex	1	16	1.337	0.265		0.044
IL R - NAcSh R	treatmt	1	16	0.23	0.638		0.003
IL R - NAcSh R	time	1	16	1.407	0.253		0.007
IL R - NAcSh R	sex:treatmt	1	16	2.426	0.139		0.028
IL R - NAcSh R	sex:time	1	16	0.441	0.516		0.002
IL R - NAcSh R	treatmt:time	1	16	3.351	0.086		0.034
IL R - NAcSh R	sex:treatmt:time	1	16	0.003	0.955		3.33E-05
IL R - PrL L	sex	1	16	1.332	0.265		0.023
IL R - PrL L	treatmt	1	16	1.367	0.259		0.015
IL R - PrL L	time	1	16	6.533	0.021	*	0.137
IL R - PrL L	sex:treatmt	1	16	0.426	0.523		0.005
IL R - PrL L	sex:time	1	16	0.034	0.855		0.000831
IL R - PrL L	treatmt:time	1	16	0.295	0.594		0.003
IL R - PrL L	sex:treatmt:time	1	16	4.317	0.054		0.04
IL R - S1 L	sex	1	16	1.243	0.281		0.028
IL R - S1 L	treatmt	1	16	1.501	0.238		0.03
IL R - S1 L	time	1	16	0.139	0.714		0.002
IL R - S1 L	sex:treatmt	1	16	1.856	0.192		0.037
IL R - S1 L	sex:time	1	16	4.449	0.051		0.048
IL R - S1 L	treatmt:time	1	16	11.781	0.003	*	0.08
IL R - S1 L	sex:treatmt:time	1	16	1.448	0.246		0.011
M1 L - AI L	sex	1	16	0.538	0.474		0.013
M1 L - AI L	treatmt	1	16	0.824	0.378		0.017
M1 L - AI L	time	1	16	8.793	0.009	*	0.096
M1 L - AI L	sex:treatmt	1	16	0.497	0.491		0.01
M1 L - AI L	sex:time	1	16	0.424	0.524		0.005
M1 L - AI L	treatmt:time	1	16	4.357	0.053		0.024
M1 L - AI L	sex:treatmt:time	1	16	6.716	0.02	*	0.037
M1 L - GIDI L	sex	1	16	0.036	0.852		0.000733
M1 L - GIDI L	treatmt	1	16	0.372	0.551		0.007
M1 L - GIDI L	time	1	16	1.493	0.239		0.024
M1 L - GIDI L	sex:treatmt	1	16	1.356	0.261		0.024
M1 L - GIDI L	sex:time	1	16	1.881	0.189		0.03
M1 L - GIDI L	treatmt:time	1	16	0.011	0.917		8.28E-05

M1 L - GIDI L	sex:treatmt:time	1	16	0.022	0.884		0.000163
M1 L - S1 L	sex	1	16	1.001	0.332		0.03
M1 L - S1 L	treatmt	1	16	2.273	0.151		0.027
M1 L - S1 L	time	1	16	5.997	0.026	*	0.068
M1 L - S1 L	sex:treatmt	1	16	1.762	0.203		0.021
M1 L - S1 L	sex:time	1	16	0.112	0.742		0.001
M1 L - S1 L	treatmt:time	1	16	0.408	0.532		0.003
M1 L - S1 L	sex:treatmt:time	1	16	3.227	0.091		0.023
M1 R - AI L	sex	1	16	1.583	0.226		0.028
M1 R - AI L	treatmt	1	16	0.913	0.354		0.011
M1 R - AI L	time	1	16	8.319	0.011	*	0.102
M1 R - AI L	sex:treatmt	1	16	0.726	0.407		0.009
M1 R - AI L	sex:time	1	16	1.155	0.298		0.016
M1 R - AI L	treatmt:time	1	16	0.028	0.869		0.000511
M1 R - AI L	sex:treatmt:time	1	16	1.275	0.276		0.023
M1 R - Cg1 L	sex	1	16	0.181	0.676		0.003
M1 R - Cg1 L	treatmt	1	16	0.669	0.425		0.017
M1 R - Cg1 L	time	1	16	0.195	0.664		0.001
M1 R - Cg1 L	sex:treatmt	1	16	0.232	0.637		0.006
M1 R - Cg1 L	sex:time	1	16	0.253	0.622		0.002
M1 R - Cg1 L	treatmt:time	1	16	0.316	0.582		0.003
M1 R - Cg1 L	sex:treatmt:time	1	16	0.182	0.675		0.002
M1 R - Cg1 R	sex	1	16	0.653	0.431		0.014
M1 R - Cg1 R	treatmt	1	16	0.186	0.672		0.004
M1 R - Cg1 R	time	1	16	0.276	0.606		0.002
M1 R - Cg1 R	sex:treatmt	1	16	1.538	0.233		0.036
M1 R - Cg1 R	sex:time	1	16	0.416	0.528		0.003
M1 R - Cg1 R	treatmt:time	1	16	0.134	0.719		0.001
M1 R - Cg1 R	sex:treatmt:time	1	16	1.202	0.289		0.01
M1 R - CPu L	sex	1	16	1.296	0.272		0.041
M1 R - CPu L	treatmt	1	16	0.002	0.966		9.12E-06
M1 R - CPu L	time	1	16	4.245	0.056		0.063
M1 R - CPu L	sex:treatmt	1	16	2.368	0.143		0.011
M1 R - CPu L	sex:time	1	16	0.006	0.938		9.90E-05
M1 R - CPu L	treatmt:time	1	16	1.18	0.294		0.01
M1 R - CPu L	sex:treatmt:time	1	16	0.298	0.593		0.003
M1 R - CPu R	sex	1	16	0.02	0.89		0.000601
M1 R - CPu R	treatmt	1	16	0.184	0.674		0.003
M1 R - CPu R	time	1	16	2.006	0.176		0.017
M1 R - CPu R	sex:treatmt	1	16	1.813	0.197		0.028
M1 R - CPu R	sex:time	1	16	0.574	0.46		0.005
M1 R - CPu R	treatmt:time	1	16	0.006	0.939		4.44E-05
M1 R - CPu R	sex:treatmt:time	1	16	2.024	0.174		0.015
M1 R - GIDI L	sex	1	16	3.411	0.083		0.071
M1 R - GIDI L	treatmt	1	16	0.059	0.811		0.000422
M1 R - GIDI L	time	1	16	5.005	0.04	*	0.101
M1 R - GIDI L	sex:treatmt	1	16	0.352	0.561		0.002
M1 R - GIDI L	sex:time	1	16	0.221	0.645		0.005
M1 R - GIDI L	treatmt:time	1	16	0.239	0.632		0.003
M1 R - GIDI L	sex:treatmt:time	1	16	0.239	0.631		0.003
M1 R - IL L	sex	1	16	0.729	0.406		0.013
M1 R - IL L	treatmt	1	16	1.407	0.253		0.03
M1 R - IL L	time	1	16	0.315	0.582		0.004
M1 R - IL L	sex:treatmt	1	16	1.469	0.243		0.031
M1 R - IL L	sex:time	1	16	2.253	0.153		0.03
M1 R - IL L	treatmt:time	1	16	0.778	0.391		0.007
M1 R - IL L	sex:treatmt:time	1	16	0.002	0.968		1.51E-05
M1 R - IL R	sex	1	16	0.51	0.485		0.014
M1 R - IL R	treatmt	1	16	0.425	0.524		0.007
M1 R - IL R	time	1	16	0.025	0.877		0.000245
M1 R - IL R	sex:treatmt	1	16	0.81	0.381		0.013
M1 R - IL R	sex:time	1	16	0.867	0.366		0.009
M1 R - IL R	treatmt:time	1	16	4.281	0.055		0.031

M1 R - IL R	sex:treatmt:time	1	16	0.767	0.394		0.006
M1 R - M1 L	sex	1	16	2.482	0.135		0.061
M1 R - M1 L	treatmt	1	16	1.465	0.244		0.019
M1 R - M1 L	time	1	16	9.719	0.007	*	0.104
M1 R - M1 L	sex:treatmt	1	16	0.024	0.879		0.00032
M1 R - M1 L	sex:time	1	16	0.081	0.779		0.000968
M1 R - M1 L	treatmt:time	1	16	0.213	0.65		0.002
M1 R - M1 L	sex:treatmt:time	1	16	0.094	0.763		0.001
M1 R - M2 L	sex	1	16	0.192	0.667		0.004
M1 R - M2 L	treatmt	1	16	0.043	0.838		0.000871
M1 R - M2 L	time	1	16	1.117	0.306		0.013
M1 R - M2 L	sex:treatmt	1	16	0.194	0.666		0.004
M1 R - M2 L	sex:time	1	16	0.005	0.943		6.11E-05
M1 R - M2 L	treatmt:time	1	16	0.135	0.718		0.001
M1 R - M2 L	sex:treatmt:time	1	16	0.342	0.567		0.004
M1 R - M2 R	sex	1	16	1.54	0.232		0.046
M1 R - M2 R	treatmt	1	16	0.068	0.798		0.000959
M1 R - M2 R	time	1	16	2.772	0.115		0.021
M1 R - M2 R	sex:treatmt	1	16	0.17	0.685		0.002
M1 R - M2 R	sex:time	1	16	0.108	0.747		0.000848
M1 R - M2 R	treatmt:time	1	16	0.093	0.764		0.000884
M1 R - M2 R	sex:treatmt:time	1	16	0.153	0.701		0.001
M1 R - NAcC L	sex	1	16	1.367	0.259		0.025
M1 R - NAcC L	treatmt	1	16	2.744	0.117		0.057
M1 R - NAcC L	time	1	16	0.717	0.41		0.007
M1 R - NAcC L	sex:treatmt	1	16	0.016	0.901		0.00035
M1 R - NAcC L	sex:time	1	16	2.013	0.175		0.019
M1 R - NAcC L	treatmt:time	1	16	0.785	0.389		0.009
M1 R - NAcC L	sex:treatmt:time	1	16	0.977	0.338		0.011
M1 R - NAcC R	sex	1	16	0.077	0.785		0.001
M1 R - NAcC R	treatmt	1	16	0.092	0.766		0.002
M1 R - NAcC R	time	1	16	0.1	0.756		0.001
M1 R - NAcC R	sex:treatmt	1	16	0.365	0.554		0.008
M1 R - NAcC R	sex:time	1	16	0.397	0.538		0.004
M1 R - NAcC R	treatmt:time	1	16	0.787	0.388		0.011
M1 R - NAcC R	sex:treatmt:time	1	16	0.011	0.917		0.000155
M1 R - NAcSh L	sex	1	16	1.844	0.193		0.031
M1 R - NAcSh L	treatmt	1	16	0.292	0.596		0.007
M1 R - NAcSh L	time	1	16	5.428	0.033	*	0.056
M1 R - NAcSh L	sex:treatmt	1	16	0.003	0.957		7.22E-05
M1 R - NAcSh L	sex:time	1	16	3.048	0.1		0.032
M1 R - NAcSh L	treatmt:time	1	16	4.501	0.05	*	0.045
M1 R - NAcSh L	sex:treatmt:time	1	16	0.007	0.932		7.74E-05
M1 R - NAcSh R	sex	1	16	1.25	0.28		0.03
M1 R - NAcSh R	treatmt	1	16	1.879	0.189		0.037
M1 R - NAcSh R	time	1	16	15.173	0.001	*	0.107
M1 R - NAcSh R	sex:treatmt	1	16	0.269	0.611		0.006
M1 R - NAcSh R	sex:time	1	16	1.591	0.225		0.012
M1 R - NAcSh R	treatmt:time	1	16	0.705	0.413		0.007
M1 R - NAcSh R	sex:treatmt:time	1	16	0.00045	0.983		4.24E-06
M1 R - PrL L	sex	1	16	1.331	0.266		0.033
M1 R - PrL L	treatmt	1	16	0.149	0.705		0.003
M1 R - PrL L	time	1	16	0.593	0.453		0.006
M1 R - PrL L	sex:treatmt	1	16	0.813	0.381		0.014
M1 R - PrL L	sex:time	1	16	0.031	0.862		0.000301
M1 R - PrL L	treatmt:time	1	16	0.004	0.952		3.81E-05
M1 R - PrL L	sex:treatmt:time	1	16	0.045	0.834		0.000459
M1 R - PrL R	sex	1	16	0.032	0.861		0.000754
M1 R - PrL R	treatmt	1	16	0.033	0.858		0.000776
M1 R - PrL R	time	1	16	3.215	0.092		0.028
M1 R - PrL R	sex:treatmt	1	16	1.183	0.293		0.027
M1 R - PrL R	sex:time	1	16	0.004	0.948		3.92E-05
M1 R - PrL R	treatmt:time	1	16	0.536	0.475		0.003

M1 R - PrL R	sex:treatmt:time	1	16	0.311	0.585		0.002
M1 R - S1 L	sex	1	16	2.647	0.123		0.041
M1 R - S1 L	treatmt	1	16	0.081	0.78		0.001
M1 R - S1 L	time	1	16	10.159	0.006	*	0.13
M1 R - S1 L	sex:treatmt	1	16	0.522	0.48		0.007
M1 R - S1 L	sex:time	1	16	0.002	0.969		2.23E-05
M1 R - S1 L	treatmt:time	1	16	0.106	0.749		0.002
M1 R - S1 L	sex:treatmt:time	1	16	0.99	0.335		0.017
M2 L - AI L	sex	1	16	0.693	0.417		0.018
M2 L - AI L	treatmt	1	16	0.526	0.479		0.01
M2 L - AI L	time	1	16	10.825	0.005	*	0.074
M2 L - AI L	sex:treatmt	1	16	3.034	0.101		0.054
M2 L - AI L	sex:time	1	16	0.365	0.554		0.003
M2 L - AI L	treatmt:time	1	16	1.55	0.231		0.015
M2 L - AI L	sex:treatmt:time	1	16	2.743	0.117		0.025
M2 L - GIDI L	sex	1	16	0.841	0.373		0.011
M2 L - GIDI L	treatmt	1	16	0.267	0.612		0.005
M2 L - GIDI L	time	1	16	0.771	0.393		0.01
M2 L - GIDI L	sex:treatmt	1	16	0.145	0.709		0.003
M2 L - GIDI L	sex:time	1	16	1.326	0.267		0.017
M2 L - GIDI L	treatmt:time	1	16	0.086	0.773		0.001
M2 L - GIDI L	sex:treatmt:time	1	16	0.598	0.451		0.01
M2 L - M1 L	sex	1	16	0.191	0.668		0.003
M2 L - M1 L	treatmt	1	16	0.13	0.723		0.004
M2 L - M1 L	time	1	16	0.663	0.427		0.005
M2 L - M1 L	sex:treatmt	1	16	0.265	0.614		0.008
M2 L - M1 L	sex:time	1	16	0.406	0.533		0.003
M2 L - M1 L	treatmt:time	1	16	0.933	0.348		0.007
M2 L - M1 L	sex:treatmt:time	1	16	0.388	0.542		0.003
M2 L - S1 L	sex	1	16	0.761	0.396		0.012
M2 L - S1 L	treatmt	1	16	0.01	0.92		0.000297
M2 L - S1 L	time	1	16	0.888	0.36		0.007
M2 L - S1 L	sex:treatmt	1	16	0.436	0.519		0.012
M2 L - S1 L	sex:time	1	16	0.762	0.396		0.006
M2 L - S1 L	treatmt:time	1	16	0.423	0.525		0.004
M2 L - S1 L	sex:treatmt:time	1	16	4.307	0.054		0.043
M2 R - AI L	sex	1	16	0.294	0.595		0.007
M2 R - AI L	treatmt	1	16	1.296	0.272		0.012
M2 R - AI L	time	1	16	11.601	0.004	*	0.121
M2 R - AI L	sex:treatmt	1	16	1.21	0.288		0.011
M2 R - AI L	sex:time	1	16	1.925	0.184		0.022
M2 R - AI L	treatmt:time	1	16	0.119	0.735		0.002
M2 R - AI L	sex:treatmt:time	1	16	1.721	0.208		0.03
M2 R - Cg1 L	sex	1	16	0.151	0.703		0.004
M2 R - Cg1 L	treatmt	1	16	3.52	0.079		0.063
M2 R - Cg1 L	time	1	16	1.919	0.185		0.018
M2 R - Cg1 L	sex:treatmt	1	16	0.288	0.599		0.006
M2 R - Cg1 L	sex:time	1	16	1.846	0.193		0.017
M2 R - Cg1 L	treatmt:time	1	16	0.059	0.811		0.000522
M2 R - Cg1 L	sex:treatmt:time	1	16	1.978	0.179		0.017
M2 R - Cg1 R	sex	1	16	0.019	0.892		0.000515
M2 R - Cg1 R	treatmt	1	16	0.188	0.67		0.004
M2 R - Cg1 R	time	1	16	0.345	0.565		0.004
M2 R - Cg1 R	sex:treatmt	1	16	0.66	0.429		0.012
M2 R - Cg1 R	sex:time	1	16	0.182	0.675		0.002
M2 R - Cg1 R	treatmt:time	1	16	0.033	0.858		0.000178
M2 R - Cg1 R	sex:treatmt:time	1	16	0.21	0.653		0.001
M2 R - CPu L	sex	1	16	2.174	0.16		0.039
M2 R - CPu L	treatmt	1	16	0.341	0.567		0.004
M2 R - CPu L	time	1	16	1.508	0.237		0.027
M2 R - CPu L	sex:treatmt	1	16	1.017	0.328		0.012
M2 R - CPu L	sex:time	1	16	0.535	0.475		0.01
M2 R - CPu L	treatmt:time	1	16	0.461	0.507		0.006

M2 R - CPu L	sex:treatmt:time	1	16	0.068	0.798		0.000867
M2 R - CPu R	sex	1	16	0.196	0.664		0.005
M2 R - CPu R	treatmt	1	16	1.559	0.23		0.025
M2 R - CPu R	time	1	16	0.981	0.337		0.009
M2 R - CPu R	sex:treatmt	1	16	5.654	0.03	*	0.086
M2 R - CPu R	sex:time	1	16	0.688	0.419		0.006
M2 R - CPu R	treatmt:time	1	16	0.255	0.621		0.003
M2 R - CPu R	sex:treatmt:time	1	16	1.165	0.296		0.014
M2 R - GIDI L	sex	1	16	0.948	0.345		0.02
M2 R - GIDI L	treatmt	1	16	0.516	0.483		0.007
M2 R - GIDI L	time	1	16	5.854	0.028	*	0.094
M2 R - GIDI L	sex:treatmt	1	16	1.289	0.273		0.017
M2 R - GIDI L	sex:time	1	16	0.001	0.971		2.48E-05
M2 R - GIDI L	treatmt:time	1	16	0.019	0.893		0.000178
M2 R - GIDI L	sex:treatmt:time	1	16	0.002	0.965		1.93E-05
M2 R - IL L	sex	1	16	0.168	0.687		0.003
M2 R - IL L	treatmt	1	16	1.087	0.313		0.024
M2 R - IL L	time	1	16	1.308	0.27		0.012
M2 R - IL L	sex:treatmt	1	16	2.66	0.122		0.057
M2 R - IL L	sex:time	1	16	0.231	0.637		0.002
M2 R - IL L	treatmt:time	1	16	1.434	0.249		0.016
M2 R - IL L	sex:treatmt:time	1	16	0.374	0.549		0.004
M2 R - IL R	sex	1	16	0.613	0.445		0.019
M2 R - IL R	treatmt	1	16	1.988	0.178		0.029
M2 R - IL R	time	1	16	0.422	0.525		0.004
M2 R - IL R	sex:treatmt	1	16	2.042	0.172		0.03
M2 R - IL R	sex:time	1	16	0.121	0.733		0.001
M2 R - IL R	treatmt:time	1	16	4.146	0.059		0.027
M2 R - IL R	sex:treatmt:time	1	16	0.327	0.575		0.002
M2 R - M1 L	sex	1	16	0.409	0.532		0.005
M2 R - M1 L	treatmt	1	16	0.62	0.443		0.013
M2 R - M1 L	time	1	16	2.271	0.151		0.033
M2 R - M1 L	sex:treatmt	1	16	0.253	0.622		0.006
M2 R - M1 L	sex:time	1	16	1.59	0.225		0.023
M2 R - M1 L	treatmt:time	1	16	2.188	0.159		0.027
M2 R - M1 L	sex:treatmt:time	1	16	0.164	0.691		0.002
M2 R - M2 L	sex	1	16	0.022	0.883		0.000559
M2 R - M2 L	treatmt	1	16	0.405	0.534		0.007
M2 R - M2 L	time	1	16	1.709	0.21		0.018
M2 R - M2 L	sex:treatmt	1	16	0.001	0.974		1.80E-05
M2 R - M2 L	sex:time	1	16	0.379	0.547		0.004
M2 R - M2 L	treatmt:time	1	16	0.843	0.372		0.008
M2 R - M2 L	sex:treatmt:time	1	16	0.494	0.492		0.005
M2 R - NAcC L	sex	1	16	0.084	0.776		0.002
M2 R - NAcC L	treatmt	1	16	6.065	0.026	*	0.091
M2 R - NAcC L	time	1	16	0.4	0.536		0.006
M2 R - NAcC L	sex:treatmt	1	16	0.129	0.725		0.002
M2 R - NAcC L	sex:time	1	16	0.179	0.677		0.003
M2 R - NAcC L	treatmt:time	1	16	0.245	0.627		0.003
M2 R - NAcC L	sex:treatmt:time	1	16	2.039	0.172		0.021
M2 R - NAcC R	sex	1	16	1.107	0.308		0.025
M2 R - NAcC R	treatmt	1	16	0.05	0.825		0.000935
M2 R - NAcC R	time	1	16	4.83	0.043	*	0.025
M2 R - NAcC R	sex:treatmt	1	16	0.125	0.729		0.002
M2 R - NAcC R	sex:time	1	16	0.463	0.506		0.002
M2 R - NAcC R	treatmt:time	1	16	1.771	0.202		0.026
M2 R - NAcC R	sex:treatmt:time	1	16	0.141	0.712		0.002
M2 R - NAcSh L	sex	1	16	2.649	0.123		0.057
M2 R - NAcSh L	treatmt	1	16	0.389	0.542		0.007
M2 R - NAcSh L	time	1	16	2.318	0.147		0.033
M2 R - NAcSh L	sex:treatmt	1	16	0.024	0.878		0.000462
M2 R - NAcSh L	sex:time	1	16	0.056	0.816		0.000815
M2 R - NAcSh L	treatmt:time	1	16	3.378	0.085		0.02

M2 R - NAcSh L	sex:treatmt:time	1	16	0.524	0.48		0.003
M2 R - NAcSh R	sex	1	16	1.372	0.259		0.034
M2 R - NAcSh R	treatmt	1	16	2.222	0.156		0.04
M2 R - NAcSh R	time	1	16	8.547	0.01	*	0.07
M2 R - NAcSh R	sex:treatmt	1	16	0.676	0.423		0.013
M2 R - NAcSh R	sex:time	1	16	0.239	0.631		0.002
M2 R - NAcSh R	treatmt:time	1	16	0.694	0.417		0.006
M2 R - NAcSh R	sex:treatmt:time	1	16	0.171	0.685		0.002
M2 R - PrL L	sex	1	16	9.121	0.008	*	0.159
M2 R - PrL L	treatmt	1	16	0.093	0.765		0.001
M2 R - PrL L	time	1	16	3.933	0.065		0.047
M2 R - PrL L	sex:treatmt	1	16	0.154	0.7		0.002
M2 R - PrL L	sex:time	1	16	1.541	0.232		0.019
M2 R - PrL L	treatmt:time	1	16	0.03	0.864		0.000481
M2 R - PrL L	sex:treatmt:time	1	16	0.087	0.772		0.001
M2 R - PrL R	sex	1	16	0.995	0.333		0.03
M2 R - PrL R	treatmt	1	16	0.002	0.969		2.66E-05
M2 R - PrL R	time	1	16	1.65E-05	0.997		8.50E-08
M2 R - PrL R	sex:treatmt	1	16	0.44	0.517		0.008
M2 R - PrL R	sex:time	1	16	0.044	0.836		0.000229
M2 R - PrL R	treatmt:time	1	16	0.01	0.921		8.84E-05
M2 R - PrL R	sex:treatmt:time	1	16	0.665	0.427		0.006
M2 R - S1 L	sex	1	16	0.051	0.823		0.00047
M2 R - S1 L	treatmt	1	16	0.008	0.932		0.000168
M2 R - S1 L	time	1	16	7.812	0.013	*	0.104
M2 R - S1 L	sex:treatmt	1	16	1.951	0.182		0.042
M2 R - S1 L	sex:time	1	16	0.058	0.813		0.000854
M2 R - S1 L	treatmt:time	1	16	0.05	0.826		0.000812
M2 R - S1 L	sex:treatmt:time	1	16	0.437	0.518		0.007
NAcC L - AI L	sex	1	16	0.633	0.438		0.013
NAcC L - AI L	treatmt	1	16	0.021	0.887		0.000317
NAcC L - AI L	time	1	16	7.93	0.012	*	0.095
NAcC L - AI L	sex:treatmt	1	16	0.768	0.394		0.012
NAcC L - AI L	sex:time	1	16	1.866	0.191		0.024
NAcC L - AI L	treatmt:time	1	16	0.378	0.547		0.005
NAcC L - AI L	sex:treatmt:time	1	16	0.72	0.409		0.01
NAcC L - Cg1 L	sex	1	16	0.038	0.848		0.001
NAcC L - Cg1 L	treatmt	1	16	6.631	0.02	*	0.085
NAcC L - Cg1 L	time	1	16	1.189	0.292		0.012
NAcC L - Cg1 L	sex:treatmt	1	16	6.365	0.023	*	0.082
NAcC L - Cg1 L	sex:time	1	16	0.57	0.461		0.006
NAcC L - Cg1 L	treatmt:time	1	16	0.183	0.674		0.002
NAcC L - Cg1 L	sex:treatmt:time	1	16	2.264	0.152		0.018
NAcC L - CPu L	sex	1	16	0.216	0.648		0.005
NAcC L - CPu L	treatmt	1	16	0.365	0.554		0.005
NAcC L - CPu L	time	1	16	0.469	0.503		0.006
NAcC L - CPu L	sex:treatmt	1	16	0.381	0.546		0.005
NAcC L - CPu L	sex:time	1	16	3.83	0.068		0.048
NAcC L - CPu L	treatmt:time	1	16	0.356	0.559		0.004
NAcC L - CPu L	sex:treatmt:time	1	16	0.451	0.512		0.005
NAcC L - GIDI L	sex	1	16	2.58E-05	0.996		5.43E-07
NAcC L - GIDI L	treatmt	1	16	0.075	0.787		0.001
NAcC L - GIDI L	time	1	16	0.026	0.873		0.000392
NAcC L - GIDI L	sex:treatmt	1	16	0.002	0.963		3.67E-05
NAcC L - GIDI L	sex:time	1	16	1.785	0.2		0.026
NAcC L - GIDI L	treatmt:time	1	16	0.77	0.393		0.007
NAcC L - GIDI L	sex:treatmt:time	1	16	0.36	0.557		0.003
NAcC L - IL L	sex	1	16	0.008	0.928		0.000204
NAcC L - IL L	treatmt	1	16	4.013	0.062		0.067
NAcC L - IL L	time	1	16	0.195	0.665		0.002
NAcC L - IL L	sex:treatmt	1	16	1.471	0.243		0.026
NAcC L - IL L	sex:time	1	16	0.013	0.91		0.000143
NAcC L - IL L	treatmt:time	1	16	0.297	0.593		0.003

NAcC L - IL L	sex:treatmt:time	1	16	0.065	0.802		0.000614
NAcC L - M1 L	sex	1	16	0.951	0.344		0.015
NAcC L - M1 L	treatmt	1	16	0.05	0.827		0.001
NAcC L - M1 L	time	1	16	0.048	0.829		0.000661
NAcC L - M1 L	sex:treatmt	1	16	0.002	0.966		4.67E-05
NAcC L - M1 L	sex:time	1	16	5.833	0.028	*	0.074
NAcC L - M1 L	treatmt:time	1	16	0.022	0.885		0.000175
NAcC L - M1 L	sex:treatmt:time	1	16	5.03	0.039	*	0.039
NAcC L - M2 L	sex	1	16	0.916	0.353		0.022
NAcC L - M2 L	treatmt	1	16	0.05	0.826		0.000782
NAcC L - M2 L	time	1	16	0.575	0.459		0.004
NAcC L - M2 L	sex:treatmt	1	16	0.844	0.372		0.013
NAcC L - M2 L	sex:time	1	16	3.669	0.073		0.026
NAcC L - M2 L	treatmt:time	1	16	0.626	0.441		0.009
NAcC L - M2 L	sex:treatmt:time	1	16	0.644	0.434		0.009
NAcC L - PrL L	sex	1	16	0.346	0.565		0.008
NAcC L - PrL L	treatmt	1	16	0.071	0.793		0.001
NAcC L - PrL L	time	1	16	0.025	0.877		0.000416
NAcC L - PrL L	sex:treatmt	1	16	2.431	0.139		0.038
NAcC L - PrL L	sex:time	1	16	0.125	0.728		0.002
NAcC L - PrL L	treatmt:time	1	16	2.439	0.138		0.014
NAcC L - PrL L	sex:treatmt:time	1	16	0.23	0.638		0.001
NAcC L - S1 L	sex	1	16	0.093	0.764		0.002
NAcC L - S1 L	treatmt	1	16	0.103	0.752		0.002
NAcC L - S1 L	time	1	16	0.441	0.516		0.008
NAcC L - S1 L	sex:treatmt	1	16	0.334	0.571		0.005
NAcC L - S1 L	sex:time	1	16	3.228	0.091		0.055
NAcC L - S1 L	treatmt:time	1	16	0.126	0.727		0.001
NAcC L - S1 L	sex:treatmt:time	1	16	0.461	0.507		0.004
NAcC R - AI L	sex	1	16	0.00016	0.99		3.55E-06
NAcC R - AI L	treatmt	1	16	0.003	0.959		5.10E-05
NAcC R - AI L	time	1	16	5.628	0.031	*	0.051
NAcC R - AI L	sex:treatmt	1	16	0.2	0.661		0.004
NAcC R - AI L	sex:time	1	16	0.002	0.967		1.72E-05
NAcC R - AI L	treatmt:time	1	16	0.086	0.773		0.001
NAcC R - AI L	sex:treatmt:time	1	16	0.013	0.909		0.00016
NAcC R - Cg1 L	sex	1	16	0.127	0.726		0.003
NAcC R - Cg1 L	treatmt	1	16	6.258	0.024	*	0.08
NAcC R - Cg1 L	time	1	16	1.108	0.308		0.018
NAcC R - Cg1 L	sex:treatmt	1	16	1.228	0.284		0.017
NAcC R - Cg1 L	sex:time	1	16	1.752	0.204		0.028
NAcC R - Cg1 L	treatmt:time	1	16	6.074	0.025	*	0.041
NAcC R - Cg1 L	sex:treatmt:time	1	16	4.827	0.043	*	0.033
NAcC R - CPu L	sex	1	16	0.622	0.442		0.007
NAcC R - CPu L	treatmt	1	16	0.177	0.679		0.004
NAcC R - CPu L	time	1	16	0.572	0.46		0.008
NAcC R - CPu L	sex:treatmt	1	16	0.331	0.573		0.007
NAcC R - CPu L	sex:time	1	16	0.518	0.482		0.007
NAcC R - CPu L	treatmt:time	1	16	0.681	0.421		0.01
NAcC R - CPu L	sex:treatmt:time	1	16	0.108	0.746		0.002
NAcC R - GIDI L	sex	1	16	0.022	0.884		0.000394
NAcC R - GIDI L	treatmt	1	16	1.633	0.22		0.035
NAcC R - GIDI L	time	1	16	0.879	0.362		0.013
NAcC R - GIDI L	sex:treatmt	1	16	0.329	0.574		0.007
NAcC R - GIDI L	sex:time	1	16	0.474	0.501		0.007
NAcC R - GIDI L	treatmt:time	1	16	0.234	0.635		0.002
NAcC R - GIDI L	sex:treatmt:time	1	16	0.872	0.364		0.007
NAcC R - IL L	sex	1	16	0.075	0.788		0.002
NAcC R - IL L	treatmt	1	16	5.718	0.029	*	0.064
NAcC R - IL L	time	1	16	0.733	0.405		0.008
NAcC R - IL L	sex:treatmt	1	16	6.044	0.026	*	0.067
NAcC R - IL L	sex:time	1	16	0.325	0.577		0.004
NAcC R - IL L	treatmt:time	1	16	1.146	0.3		0.014

NAcC R - IL L	sex:treatmt:time	1	16	0.237	0.633		0.003
NAcC R - M1 L	sex	1	16	0.025	0.877		0.000626
NAcC R - M1 L	treatmt	1	16	1.012	0.329		0.013
NAcC R - M1 L	time	1	16	0.591	0.453		0.011
NAcC R - M1 L	sex:treatmt	1	16	3.82	0.068		0.046
NAcC R - M1 L	sex:time	1	16	0.035	0.854		0.000663
NAcC R - M1 L	treatmt:time	1	16	0.496	0.491		0.003
NAcC R - M1 L	sex:treatmt:time	1	16	0.692	0.418		0.004
NAcC R - M2 L	sex	1	16	0.013	0.909		0.000326
NAcC R - M2 L	treatmt	1	16	0.055	0.818		0.000598
NAcC R - M2 L	time	1	16	1.311	0.269		0.022
NAcC R - M2 L	sex:treatmt	1	16	0.003	0.956		3.52E-05
NAcC R - M2 L	sex:time	1	16	0.00042	0.984		7.35E-06
NAcC R - M2 L	treatmt:time	1	16	0.036	0.853		0.000352
NAcC R - M2 L	sex:treatmt:time	1	16	0.599	0.45		0.006
NAcC R - NAcC L	sex	1	16	0.046	0.832		0.000801
NAcC R - NAcC L	treatmt	1	16	0.187	0.671		0.003
NAcC R - NAcC L	time	1	16	0.582	0.457		0.007
NAcC R - NAcC L	sex:treatmt	1	16	0.153	0.701		0.002
NAcC R - NAcC L	sex:time	1	16	3.126	0.096		0.036
NAcC R - NAcC L	treatmt:time	1	16	0.238	0.632		0.004
NAcC R - NAcC L	sex:treatmt:time	1	16	0.229	0.639		0.004
NAcC R - NAcSh l	sex	1	16	0.96	0.342		0.018
NAcC R - NAcSh l	treatmt	1	16	0.929	0.35		0.014
NAcC R - NAcSh l	time	1	16	0.003	0.955		6.24E-05
NAcC R - NAcSh l	sex:treatmt	1	16	0.961	0.342		0.015
NAcC R - NAcSh l	sex:time	1	16	0.564	0.463		0.011
NAcC R - NAcSh l	treatmt:time	1	16	3.871	0.067		0.032
NAcC R - NAcSh l	sex:treatmt:time	1	16	1.942	0.183		0.016
NAcC R - NAcSh F	sex	1	16	0.218	0.647		0.008
NAcC R - NAcSh F	treatmt	1	16	0.673	0.424		0.006
NAcC R - NAcSh F	time	1	16	1.43	0.249		0.015
NAcC R - NAcSh F	sex:treatmt	1	16	3.735	0.071		0.034
NAcC R - NAcSh F	sex:time	1	16	0.828	0.376		0.009
NAcC R - NAcSh F	treatmt:time	1	16	5.078	0.039	*	0.035
NAcC R - NAcSh F	sex:treatmt:time	1	16	3.377	0.085		0.024
NAcC R - PrL L	sex	1	16	0.028	0.869		0.000729
NAcC R - PrL L	treatmt	1	16	0.283	0.602		0.004
NAcC R - PrL L	time	1	16	0.16	0.695		0.002
NAcC R - PrL L	sex:treatmt	1	16	0.157	0.697		0.002
NAcC R - PrL L	sex:time	1	16	1.236	0.283		0.019
NAcC R - PrL L	treatmt:time	1	16	0.09	0.768		0.000656
NAcC R - PrL L	sex:treatmt:time	1	16	1.489	0.24		0.011
NAcC R - S1 L	sex	1	16	0.017	0.898		0.000273
NAcC R - S1 L	treatmt	1	16	2.02	0.174		0.04
NAcC R - S1 L	time	1	16	1.80E-05	0.997		3.40E-07
NAcC R - S1 L	sex:treatmt	1	16	0.065	0.802		0.001
NAcC R - S1 L	sex:time	1	16	2.89	0.108		0.052
NAcC R - S1 L	treatmt:time	1	16	0.822	0.378		0.006
NAcC R - S1 L	sex:treatmt:time	1	16	3.103	0.097		0.021
NAcSh L - AI L	sex	1	16	0.069	0.796		0.001
NAcSh L - AI L	treatmt	1	16	0.42	0.526		0.007
NAcSh L - AI L	time	1	16	8.519	0.01	*	0.113
NAcSh L - AI L	sex:treatmt	1	16	0.437	0.518		0.007
NAcSh L - AI L	sex:time	1	16	0.665	0.427		0.01
NAcSh L - AI L	treatmt:time	1	16	1.114	0.307		0.013
NAcSh L - AI L	sex:treatmt:time	1	16	1.318	0.268		0.015
NAcSh L - Cg1 L	sex	1	16	3.637	0.075		0.046
NAcSh L - Cg1 L	treatmt	1	16	0.265	0.613		0.008
NAcSh L - Cg1 L	time	1	16	4.54	0.049	*	0.057
NAcSh L - Cg1 L	sex:treatmt	1	16	1.568	0.228		0.046
NAcSh L - Cg1 L	sex:time	1	16	0.211	0.652		0.003
NAcSh L - Cg1 L	treatmt:time	1	16	0.972	0.339		0.005

NAcSh L - Cg1 L	sex:treatmt:time	1	16	0.25	0.624		0.001
NAcSh L - CPu L	sex	1	16	0.195	0.665		0.004
NAcSh L - CPu L	treatmt	1	16	0.768	0.394		0.017
NAcSh L - CPu L	time	1	16	3.293	0.088		0.036
NAcSh L - CPu L	sex:treatmt	1	16	2.541	0.13		0.055
NAcSh L - CPu L	sex:time	1	16	0.372	0.551		0.004
NAcSh L - CPu L	treatmt:time	1	16	7.881	0.013	*	0.071
NAcSh L - CPu L	sex:treatmt:time	1	16	1.793	0.199		0.017
NAcSh L - GIDI L	sex	1	16	1.127	0.304		0.01
NAcSh L - GIDI L	treatmt	1	16	0.433	0.52		0.008
NAcSh L - GIDI L	time	1	16	1.863	0.191		0.031
NAcSh L - GIDI L	sex:treatmt	1	16	0.194	0.666		0.004
NAcSh L - GIDI L	sex:time	1	16	0.073	0.791		0.001
NAcSh L - GIDI L	treatmt:time	1	16	1.09	0.312		0.019
NAcSh L - GIDI L	sex:treatmt:time	1	16	0.657	0.429		0.011
NAcSh L - IL L	sex	1	16	0.285	0.601		0.008
NAcSh L - IL L	treatmt	1	16	0.001	0.972		1.60E-05
NAcSh L - IL L	time	1	16	0.135	0.718		0.001
NAcSh L - IL L	sex:treatmt	1	16	1.669	0.215		0.021
NAcSh L - IL L	sex:time	1	16	0.598	0.451		0.005
NAcSh L - IL L	treatmt:time	1	16	0.775	0.392		0.01
NAcSh L - IL L	sex:treatmt:time	1	16	1.068	0.317		0.014
NAcSh L - M1 L	sex	1	16	0.002	0.965		3.95E-05
NAcSh L - M1 L	treatmt	1	16	4.48E-05	0.995		1.06E-06
NAcSh L - M1 L	time	1	16	6.849	0.019	*	0.063
NAcSh L - M1 L	sex:treatmt	1	16	0.926	0.35		0.021
NAcSh L - M1 L	sex:time	1	16	2.806	0.113		0.027
NAcSh L - M1 L	treatmt:time	1	16	7.836	0.013	*	0.068
NAcSh L - M1 L	sex:treatmt:time	1	16	0.006	0.939		5.55E-05
NAcSh L - M2 L	sex	1	16	0.057	0.815		0.001
NAcSh L - M2 L	treatmt	1	16	0.12	0.734		0.003
NAcSh L - M2 L	time	1	16	0.024	0.88		0.000261
NAcSh L - M2 L	sex:treatmt	1	16	0.834	0.375		0.019
NAcSh L - M2 L	sex:time	1	16	0.522	0.48		0.006
NAcSh L - M2 L	treatmt:time	1	16	6.064	0.026	*	0.059
NAcSh L - M2 L	sex:treatmt:time	1	16	0.112	0.742		0.001
NAcSh L - NAcC L	sex	1	16	0.149	0.705		0.003
NAcSh L - NAcC L	treatmt	1	16	0.002	0.964		5.13E-05
NAcSh L - NAcC L	time	1	16	0.104	0.751		0.000939
NAcSh L - NAcC L	sex:treatmt	1	16	5.402	0.034	*	0.115
NAcSh L - NAcC L	sex:time	1	16	0.561	0.465		0.005
NAcSh L - NAcC L	treatmt:time	1	16	8.457	0.01	*	0.048
NAcSh L - NAcC L	sex:treatmt:time	1	16	0.055	0.817		0.000326
NAcSh L - PrL L	sex	1	16	0.00024	0.988		4.12E-06
NAcSh L - PrL L	treatmt	1	16	0.082	0.778		0.001
NAcSh L - PrL L	time	1	16	0.875	0.363		0.017
NAcSh L - PrL L	sex:treatmt	1	16	3.197	0.093		0.05
NAcSh L - PrL L	sex:time	1	16	0.052	0.823		0.001
NAcSh L - PrL L	treatmt:time	1	16	1.63	0.22		0.015
NAcSh L - PrL L	sex:treatmt:time	1	16	0.444	0.515		0.004
NAcSh L - S1 L	sex	1	16	1.899	0.187		0.026
NAcSh L - S1 L	treatmt	1	16	0.28	0.604		0.005
NAcSh L - S1 L	time	1	16	2.337	0.146		0.045
NAcSh L - S1 L	sex:treatmt	1	16	2.093	0.167		0.037
NAcSh L - S1 L	sex:time	1	16	2.334	0.146		0.045
NAcSh L - S1 L	treatmt:time	1	16	4.448	0.051		0.041
NAcSh L - S1 L	sex:treatmt:time	1	16	6.763	0.019	*	0.062
NAcSh R - AI L	sex	1	16	0.013	0.909		0.000222
NAcSh R - AI L	treatmt	1	16	0.099	0.757		0.002
NAcSh R - AI L	time	1	16	9.683	0.007	*	0.111
NAcSh R - AI L	sex:treatmt	1	16	0.24	0.631		0.004
NAcSh R - AI L	sex:time	1	16	0.023	0.882		0.000293
NAcSh R - AI L	treatmt:time	1	16	0.248	0.625		0.004

NAcSh R - AI L	sex:treatmt:time	1	16	0.346	0.564		0.005
NAcSh R - Cg1 L	sex	1	16	0.486	0.496		0.01
NAcSh R - Cg1 L	treatmt	1	16	0.82	0.378		0.02
NAcSh R - Cg1 L	time	1	16	2.145	0.162		0.022
NAcSh R - Cg1 L	sex:treatmt	1	16	1.188	0.292		0.028
NAcSh R - Cg1 L	sex:time	1	16	0.902	0.356		0.009
NAcSh R - Cg1 L	treatmt:time	1	16	0.519	0.482		0.003
NAcSh R - Cg1 L	sex:treatmt:time	1	16	1.82	0.196		0.012
NAcSh R - CPu L	sex	1	16	0.028	0.87		0.000378
NAcSh R - CPu L	treatmt	1	16	0.098	0.759		0.003
NAcSh R - CPu L	time	1	16	8.94	0.009	*	0.103
NAcSh R - CPu L	sex:treatmt	1	16	0.606	0.448		0.016
NAcSh R - CPu L	sex:time	1	16	0.25	0.624		0.003
NAcSh R - CPu L	treatmt:time	1	16	5.48	0.033	*	0.044
NAcSh R - CPu L	sex:treatmt:time	1	16	1.087	0.313		0.009
NAcSh R - GIDI L	sex	1	16	0.709	0.412		0.006
NAcSh R - GIDI L	treatmt	1	16	0.673	0.424		0.015
NAcSh R - GIDI L	time	1	16	2.297	0.149		0.04
NAcSh R - GIDI L	sex:treatmt	1	16	0.044	0.837		0.000986
NAcSh R - GIDI L	sex:time	1	16	0.143	0.71		0.003
NAcSh R - GIDI L	treatmt:time	1	16	1.155	0.299		0.015
NAcSh R - GIDI L	sex:treatmt:time	1	16	0.801	0.384		0.011
NAcSh R - IL L	sex	1	16	0.226	0.641		0.007
NAcSh R - IL L	treatmt	1	16	0.074	0.79		0.000748
NAcSh R - IL L	time	1	16	0.76	0.396		0.008
NAcSh R - IL L	sex:treatmt	1	16	3.763	0.07		0.037
NAcSh R - IL L	sex:time	1	16	0.908	0.355		0.009
NAcSh R - IL L	treatmt:time	1	16	2.417	0.14		0.024
NAcSh R - IL L	sex:treatmt:time	1	16	0.644	0.434		0.007
NAcSh R - M1 L	sex	1	16	0.108	0.747		0.003
NAcSh R - M1 L	treatmt	1	16	0.338	0.569		0.007
NAcSh R - M1 L	time	1	16	16.251	0.00097	*	0.1
NAcSh R - M1 L	sex:treatmt	1	16	0.069	0.796		0.001
NAcSh R - M1 L	sex:time	1	16	0.093	0.765		0.000632
NAcSh R - M1 L	treatmt:time	1	16	4.91	0.042	*	0.028
NAcSh R - M1 L	sex:treatmt:time	1	16	0.352	0.561		0.002
NAcSh R - M2 L	sex	1	16	0.00011	0.992		3.54E-06
NAcSh R - M2 L	treatmt	1	16	0.021	0.886		0.000379
NAcSh R - M2 L	time	1	16	0.86	0.368		0.008
NAcSh R - M2 L	sex:treatmt	1	16	0.278	0.605		0.005
NAcSh R - M2 L	sex:time	1	16	0.089	0.769		0.000806
NAcSh R - M2 L	treatmt:time	1	16	1.843	0.193		0.008
NAcSh R - M2 L	sex:treatmt:time	1	16	0.113	0.741		0.000514
NAcSh R - NAcC l	sex	1	16	0.342	0.567		0.012
NAcSh R - NAcC l	treatmt	1	16	1.183	0.293		0.018
NAcSh R - NAcC l	time	1	16	1.471	0.243		0.01
NAcSh R - NAcC l	sex:treatmt	1	16	1.861	0.191		0.029
NAcSh R - NAcC l	sex:time	1	16	0.959	0.342		0.007
NAcSh R - NAcC l	treatmt:time	1	16	3.137	0.096		0.016
NAcSh R - NAcC l	sex:treatmt:time	1	16	0.868	0.365		0.004
NAcSh R - NAcSh	sex	1	16	0.32	0.58		0.009
NAcSh R - NAcSh	treatmt	1	16	0.545	0.471		0.011
NAcSh R - NAcSh	time	1	16	2.643	0.124		0.022
NAcSh R - NAcSh	sex:treatmt	1	16	0.204	0.658		0.004
NAcSh R - NAcSh	sex:time	1	16	2.673	0.122		0.022
NAcSh R - NAcSh	treatmt:time	1	16	1.976	0.179		0.01
NAcSh R - NAcSh	sex:treatmt:time	1	16	0.535	0.475		0.003
NAcSh R - PrL L	sex	1	16	0.064	0.803		0.001
NAcSh R - PrL L	treatmt	1	16	0.999	0.332		0.014
NAcSh R - PrL L	time	1	16	0.738	0.403		0.015
NAcSh R - PrL L	sex:treatmt	1	16	2.595	0.127		0.035
NAcSh R - PrL L	sex:time	1	16	1.816	0.197		0.037
NAcSh R - PrL L	treatmt:time	1	16	0.411	0.53		0.004

NAcSh R - PrL L	sex:treatmt:time	1	16	0.693	0.417		0.007
NAcSh R - S1 L	sex	1	16	0.673	0.424		0.011
NAcSh R - S1 L	treatmt	1	16	0.441	0.516		0.01
NAcSh R - S1 L	time	1	16	2.943	0.106		0.045
NAcSh R - S1 L	sex:treatmt	1	16	0.014	0.908		0.000302
NAcSh R - S1 L	sex:time	1	16	0.742	0.402		0.012
NAcSh R - S1 L	treatmt:time	1	16	2.048	0.172		0.017
NAcSh R - S1 L	sex:treatmt:time	1	16	5.594	0.031	*	0.044
PrL L - AI L	sex	1	16	0.486	0.496		0.011
PrL L - AI L	treatmt	1	16	2.131	0.164		0.044
PrL L - AI L	time	1	16	1.631	0.22		0.019
PrL L - AI L	sex:treatmt	1	16	0.208	0.655		0.004
PrL L - AI L	sex:time	1	16	0.609	0.447		0.007
PrL L - AI L	treatmt:time	1	16	0.327	0.575		0.002
PrL L - AI L	sex:treatmt:time	1	16	5.166	0.037	*	0.03
PrL L - Cg1 L	sex	1	16	0.027	0.871		0.000773
PrL L - Cg1 L	treatmt	1	16	1.004	0.331		0.013
PrL L - Cg1 L	time	1	16	1.221	0.286		0.017
PrL L - Cg1 L	sex:treatmt	1	16	0.364	0.555		0.005
PrL L - Cg1 L	sex:time	1	16	1.329	0.266		0.018
PrL L - Cg1 L	treatmt:time	1	16	7.095	0.017	*	0.047
PrL L - Cg1 L	sex:treatmt:time	1	16	2.517	0.132		0.017
PrL L - GIDI L	sex	1	16	0.659	0.429		0.015
PrL L - GIDI L	treatmt	1	16	0.371	0.551		0.008
PrL L - GIDI L	time	1	16	4.134	0.059		0.045
PrL L - GIDI L	sex:treatmt	1	16	0.172	0.684		0.004
PrL L - GIDI L	sex:time	1	16	0.009	0.927		9.79E-05
PrL L - GIDI L	treatmt:time	1	16	3.077	0.099		0.016
PrL L - GIDI L	sex:treatmt:time	1	16	1.511	0.237		0.008
PrL L - M1 L	sex	1	16	0.36	0.557		0.009
PrL L - M1 L	treatmt	1	16	1.277	0.275		0.025
PrL L - M1 L	time	1	16	0.197	0.663		0.002
PrL L - M1 L	sex:treatmt	1	16	0.005	0.947		8.96E-05
PrL L - M1 L	sex:time	1	16	0.517	0.483		0.006
PrL L - M1 L	treatmt:time	1	16	0.376	0.549		0.002
PrL L - M1 L	sex:treatmt:time	1	16	0.027	0.873		0.000111
PrL L - M2 L	sex	1	16	0.068	0.797		0.001
PrL L - M2 L	treatmt	1	16	0.406	0.533		0.006
PrL L - M2 L	time	1	16	0.615	0.444		0.012
PrL L - M2 L	sex:treatmt	1	16	0.266	0.613		0.004
PrL L - M2 L	sex:time	1	16	2.167	0.16		0.041
PrL L - M2 L	treatmt:time	1	16	0.036	0.852		0.000313
PrL L - M2 L	sex:treatmt:time	1	16	0.882	0.362		0.008
PrL L - S1 L	sex	1	16	0.959	0.342		0.017
PrL L - S1 L	treatmt	1	16	0.429	0.522		0.012
PrL L - S1 L	time	1	16	0.667	0.426		0.005
PrL L - S1 L	sex:treatmt	1	16	0.191	0.668		0.005
PrL L - S1 L	sex:time	1	16	0.04	0.845		0.000318
PrL L - S1 L	treatmt:time	1	16	0.008	0.931		6.53E-05
PrL L - S1 L	sex:treatmt:time	1	16	2.847	0.111		0.024
PrL R - AI L	sex	1	16	0.355	0.56		0.008
PrL R - AI L	treatmt	1	16	0.303	0.59		0.007
PrL R - AI L	time	1	16	0.00087	0.977		7.01E-06
PrL R - AI L	sex:treatmt	1	16	0.12	0.734		0.003
PrL R - AI L	sex:time	1	16	0.359	0.558		0.003
PrL R - AI L	treatmt:time	1	16	0.049	0.827		0.000456
PrL R - AI L	sex:treatmt:time	1	16	5.243	0.036	*	0.046
PrL R - Cg1 L	sex	1	16	0.532	0.476		0.011
PrL R - Cg1 L	treatmt	1	16	0.21	0.653		0.004
PrL R - Cg1 L	time	1	16	0.016	0.902		0.000254
PrL R - Cg1 L	sex:treatmt	1	16	1.688	0.212		0.029
PrL R - Cg1 L	sex:time	1	16	0.05	0.827		0.000798
PrL R - Cg1 L	treatmt:time	1	16	1.014	0.329		0.008

PrL R - Cg1 L	sex:treatmt:time	1	16	0.129	0.724		0.001
PrL R - CPu L	sex	1	16	0.115	0.739		0.003
PrL R - CPu L	treatmt	1	16	2.112	0.165		0.042
PrL R - CPu L	time	1	16	2.981	0.104		0.024
PrL R - CPu L	sex:treatmt	1	16	1.311	0.269		0.026
PrL R - CPu L	sex:time	1	16	0.071	0.793		0.000592
PrL R - CPu L	treatmt:time	1	16	3.249	0.09		0.025
PrL R - CPu L	sex:treatmt:time	1	16	0.277	0.606		0.002
PrL R - CPu R	sex	1	16	0.595	0.452		0.014
PrL R - CPu R	treatmt	1	16	0.311	0.585		0.006
PrL R - CPu R	time	1	16	0.561	0.465		0.007
PrL R - CPu R	sex:treatmt	1	16	1.201	0.289		0.023
PrL R - CPu R	sex:time	1	16	1.077	0.315		0.013
PrL R - CPu R	treatmt:time	1	16	5.381	0.034	*	0.035
PrL R - CPu R	sex:treatmt:time	1	16	0.051	0.824		0.000348
PrL R - GIDI L	sex	1	16	0.099	0.757		0.002
PrL R - GIDI L	treatmt	1	16	0.171	0.685		0.004
PrL R - GIDI L	time	1	16	0.349	0.563		0.003
PrL R - GIDI L	sex:treatmt	1	16	0.533	0.476		0.014
PrL R - GIDI L	sex:time	1	16	4.949	0.041	*	0.042
PrL R - GIDI L	treatmt:time	1	16	1.989	0.178		0.01
PrL R - GIDI L	sex:treatmt:time	1	16	0.045	0.835		0.000227
PrL R - IL L	sex	1	16	0.836	0.374		0.013
PrL R - IL L	treatmt	1	16	0.00034	0.985		6.67E-06
PrL R - IL L	time	1	16	10.726	0.005	*	0.165
PrL R - IL L	sex:treatmt	1	16	1.673	0.214		0.031
PrL R - IL L	sex:time	1	16	0.784	0.389		0.014
PrL R - IL L	treatmt:time	1	16	0.38	0.546		0.003
PrL R - IL L	sex:treatmt:time	1	16	0.614	0.445		0.006
PrL R - IL R	sex	1	16	1.502	0.238		0.037
PrL R - IL R	treatmt	1	16	1.648	0.218		0.023
PrL R - IL R	time	1	16	0.685	0.42		0.009
PrL R - IL R	sex:treatmt	1	16	0.984	0.336		0.014
PrL R - IL R	sex:time	1	16	0.795	0.386		0.011
PrL R - IL R	treatmt:time	1	16	0.042	0.841		0.000377
PrL R - IL R	sex:treatmt:time	1	16	0.03	0.864		0.000275
PrL R - M1 L	sex	1	16	0.14	0.713		0.004
PrL R - M1 L	treatmt	1	16	0.59	0.454		0.012
PrL R - M1 L	time	1	16	5.163	0.037	*	0.045
PrL R - M1 L	sex:treatmt	1	16	0.00049	0.983		1.00E-05
PrL R - M1 L	sex:time	1	16	0.317	0.581		0.003
PrL R - M1 L	treatmt:time	1	16	1.031	0.325		0.008
PrL R - M1 L	sex:treatmt:time	1	16	0.253	0.622		0.002
PrL R - M2 L	sex	1	16	0.052	0.822		0.001
PrL R - M2 L	treatmt	1	16	0.086	0.774		0.002
PrL R - M2 L	time	1	16	5.561	0.031	*	0.068
PrL R - M2 L	sex:treatmt	1	16	0.0006	0.981		1.14E-05
PrL R - M2 L	sex:time	1	16	0.763	0.395		0.01
PrL R - M2 L	treatmt:time	1	16	0.95	0.344		0.007
PrL R - M2 L	sex:treatmt:time	1	16	1.399	0.254		0.01
PrL R - NAcC L	sex	1	16	0.059	0.812		0.000801
PrL R - NAcC L	treatmt	1	16	0.351	0.562		0.009
PrL R - NAcC L	time	1	16	5.979	0.026	*	0.076
PrL R - NAcC L	sex:treatmt	1	16	0.16	0.694		0.004
PrL R - NAcC L	sex:time	1	16	3.23E-06	0.999		4.47E-08
PrL R - NAcC L	treatmt:time	1	16	4.608	0.047	*	0.035
PrL R - NAcC L	sex:treatmt:time	1	16	0.297	0.593		0.002
PrL R - NAcC R	sex	1	16	1.04	0.323		0.022
PrL R - NAcC R	treatmt	1	16	2.221	0.156		0.043
PrL R - NAcC R	time	1	16	5.332	0.035	*	0.05
PrL R - NAcC R	sex:treatmt	1	16	0.436	0.519		0.009
PrL R - NAcC R	sex:time	1	16	1.468	0.243		0.014
PrL R - NAcC R	treatmt:time	1	16	1.638	0.219		0.018

PrL R - NAcC R	sex:treatmt:time	1	16	0.093	0.764		0.001
PrL R - NAcSh L	sex	1	16	0.006	0.938		8.79E-05
PrL R - NAcSh L	treatmt	1	16	0.233	0.636		0.004
PrL R - NAcSh L	time	1	16	1.182	0.293		0.015
PrL R - NAcSh L	sex:treatmt	1	16	3.432	0.082		0.061
PrL R - NAcSh L	sex:time	1	16	0.01	0.92		0.000133
PrL R - NAcSh L	treatmt:time	1	16	0.169	0.687		0.003
PrL R - NAcSh L	sex:treatmt:time	1	16	0.851	0.37		0.014
PrL R - NAcSh R	sex	1	16	1.701	0.211		0.029
PrL R - NAcSh R	treatmt	1	16	2.024	0.174		0.03
PrL R - NAcSh R	time	1	16	0.026	0.873		0.000367
PrL R - NAcSh R	sex:treatmt	1	16	3.007	0.102		0.044
PrL R - NAcSh R	sex:time	1	16	0.589	0.454		0.008
PrL R - NAcSh R	treatmt:time	1	16	0.02	0.889		0.000312
PrL R - NAcSh R	sex:treatmt:time	1	16	0.16	0.694		0.002
PrL R - PrL L	sex	1	16	0.332	0.573		0.006
PrL R - PrL L	treatmt	1	16	0.248	0.625		0.006
PrL R - PrL L	time	1	16	23.633	0.00017	*	0.22
PrL R - PrL L	sex:treatmt	1	16	2.825	0.112		0.061
PrL R - PrL L	sex:time	1	16	0.089	0.769		0.001
PrL R - PrL L	treatmt:time	1	16	1	0.332		0.009
PrL R - PrL L	sex:treatmt:time	1	16	3.47	0.081		0.03
PrL R - S1 L	sex	1	16	0.355	0.56		0.009
PrL R - S1 L	treatmt	1	16	0.163	0.692		0.005
PrL R - S1 L	time	1	16	11.282	0.004	*	0.035
PrL R - S1 L	sex:treatmt	1	16	0.087	0.772		0.002
PrL R - S1 L	sex:time	1	16	7.608	0.014	*	0.024
PrL R - S1 L	treatmt:time	1	16	0.15	0.704		0.000732
PrL R - S1 L	sex:treatmt:time	1	16	0.354	0.56		0.002
S1 L - AI L	sex	1	16	0.633	0.438		0.013
S1 L - AI L	treatmt	1	16	1.842	0.194		0.049
S1 L - AI L	time	1	16	6.837	0.019	*	0.048
S1 L - AI L	sex:treatmt	1	16	0.023	0.883		0.000634
S1 L - AI L	sex:time	1	16	0.035	0.855		0.000255
S1 L - AI L	treatmt:time	1	16	0.17	0.685		0.000984
S1 L - AI L	sex:treatmt:time	1	16	7.345	0.015	*	0.041
S1 L - GIDI L	sex	1	16	0.186	0.672		0.005
S1 L - GIDI L	treatmt	1	16	2.173	0.16		0.038
S1 L - GIDI L	time	1	16	3.897	0.066		0.053
S1 L - GIDI L	sex:treatmt	1	16	0.814	0.38		0.014
S1 L - GIDI L	sex:time	1	16	1.724	0.208		0.024
S1 L - GIDI L	treatmt:time	1	16	0.002	0.965		7.93E-06
S1 L - GIDI L	sex:treatmt:time	1	16	0.214	0.65		0.000833
S1 R - AI L	sex	1	16	1.775	0.201		0.043
S1 R - AI L	treatmt	1	16	0.499	0.49		0.008
S1 R - AI L	time	1	16	5.779	0.029	*	0.064
S1 R - AI L	sex:treatmt	1	16	0.459	0.508		0.007
S1 R - AI L	sex:time	1	16	0.205	0.657		0.002
S1 R - AI L	treatmt:time	1	16	1.286	0.273		0.012
S1 R - AI L	sex:treatmt:time	1	16	3.543	0.078		0.031
S1 R - Cg1 L	sex	1	16	0.137	0.716		0.003
S1 R - Cg1 L	treatmt	1	16	0.437	0.518		0.008
S1 R - Cg1 L	time	1	16	0.328	0.575		0.002
S1 R - Cg1 L	sex:treatmt	1	16	0.833	0.375		0.014
S1 R - Cg1 L	sex:time	1	16	0.497	0.491		0.004
S1 R - Cg1 L	treatmt:time	1	16	0.00075	0.979		9.18E-06
S1 R - Cg1 L	sex:treatmt:time	1	16	0.821	0.378		0.01
S1 R - Cg1 R	sex	1	16	1.429	0.249		0.04
S1 R - Cg1 R	treatmt	1	16	0.005	0.946		9.16E-05
S1 R - Cg1 R	time	1	16	0.18	0.677		0.001
S1 R - Cg1 R	sex:treatmt	1	16	0.179	0.678		0.003
S1 R - Cg1 R	sex:time	1	16	1.32	0.268		0.01
S1 R - Cg1 R	treatmt:time	1	16	0.335	0.571		0.002

S1 R - Cg1 R	sex:treatmt:time	1	16	0.458	0.508		0.003
S1 R - CPu L	sex	1	16	0.255	0.62		0.005
S1 R - CPu L	treatmt	1	16	0.348	0.563		0.006
S1 R - CPu L	time	1	16	0.182	0.675		0.004
S1 R - CPu L	sex:treatmt	1	16	0.064	0.804		0.001
S1 R - CPu L	sex:time	1	16	0.008	0.928		0.000166
S1 R - CPu L	treatmt:time	1	16	0.175	0.681		0.002
S1 R - CPu L	sex:treatmt:time	1	16	1.292	0.272		0.011
S1 R - CPu R	sex	1	16	0.089	0.769		0.002
S1 R - CPu R	treatmt	1	16	0.415	0.529		0.006
S1 R - CPu R	time	1	16	1.435	0.248		0.023
S1 R - CPu R	sex:treatmt	1	16	0.198	0.662		0.003
S1 R - CPu R	sex:time	1	16	0.569	0.462		0.009
S1 R - CPu R	treatmt:time	1	16	0.112	0.742		0.000929
S1 R - CPu R	sex:treatmt:time	1	16	0.456	0.509		0.004
S1 R - GIDI L	sex	1	16	2.958	0.105		0.064
S1 R - GIDI L	treatmt	1	16	0.087	0.771		0.001
S1 R - GIDI L	time	1	16	2.124	0.164		0.04
S1 R - GIDI L	sex:treatmt	1	16	1.703	0.21		0.026
S1 R - GIDI L	sex:time	1	16	1.432	0.249		0.027
S1 R - GIDI L	treatmt:time	1	16	1.028	0.326		0.004
S1 R - GIDI L	sex:treatmt:time	1	16	11.112	0.004	*	0.043
S1 R - IL L	sex	1	16	0.424	0.524		0.007
S1 R - IL L	treatmt	1	16	3.191	0.093		0.083
S1 R - IL L	time	1	16	0.009	0.924		9.85E-05
S1 R - IL L	sex:treatmt	1	16	1.706	0.21		0.046
S1 R - IL L	sex:time	1	16	3.501	0.08		0.035
S1 R - IL L	treatmt:time	1	16	0.04	0.844		0.000276
S1 R - IL L	sex:treatmt:time	1	16	0.247	0.626		0.002
S1 R - IL R	sex	1	16	1.602	0.224		0.04
S1 R - IL R	treatmt	1	16	3.053	0.1		0.065
S1 R - IL R	time	1	16	3.625	0.075		0.017
S1 R - IL R	sex:treatmt	1	16	1.504	0.238		0.033
S1 R - IL R	sex:time	1	16	6.029	0.026	*	0.029
S1 R - IL R	treatmt:time	1	16	3.773	0.07		0.033
S1 R - IL R	sex:treatmt:time	1	16	0.168	0.688		0.002
S1 R - M1 L	sex	1	16	3.031	0.101		0.049
S1 R - M1 L	treatmt	1	16	0.048	0.829		0.000645
S1 R - M1 L	time	1	16	0.257	0.619		0.006
S1 R - M1 L	sex:treatmt	1	16	0.043	0.838		0.000576
S1 R - M1 L	sex:time	1	16	0.018	0.895		0.000427
S1 R - M1 L	treatmt:time	1	16	0.871	0.365		0.007
S1 R - M1 L	sex:treatmt:time	1	16	3.401	0.084		0.028
S1 R - M1 R	sex	1	16	0.022	0.883		0.000349
S1 R - M1 R	treatmt	1	16	0.042	0.84		0.001
S1 R - M1 R	time	1	16	3.532	0.079		0.049
S1 R - M1 R	sex:treatmt	1	16	0.479	0.499		0.012
S1 R - M1 R	sex:time	1	16	0.155	0.699		0.002
S1 R - M1 R	treatmt:time	1	16	0.506	0.487		0.004
S1 R - M1 R	sex:treatmt:time	1	16	0.473	0.501		0.003
S1 R - M2 L	sex	1	16	0.063	0.806		0.001
S1 R - M2 L	treatmt	1	16	0.06	0.809		0.001
S1 R - M2 L	time	1	16	0.161	0.693		0.001
S1 R - M2 L	sex:treatmt	1	16	1.264	0.277		0.023
S1 R - M2 L	sex:time	1	16	0.67	0.425		0.006
S1 R - M2 L	treatmt:time	1	16	0.234	0.635		0.003
S1 R - M2 L	sex:treatmt:time	1	16	0.569	0.462		0.007
S1 R - M2 R	sex	1	16	3.193	0.093		0.061
S1 R - M2 R	treatmt	1	16	0.286	0.6		0.005
S1 R - M2 R	time	1	16	3.75	0.071		0.06
S1 R - M2 R	sex:treatmt	1	16	3.387	0.084		0.053
S1 R - M2 R	sex:time	1	16	0.758	0.397		0.013
S1 R - M2 R	treatmt:time	1	16	0.296	0.594		0.003

S1 R - M2 R	sex:treatmt:time	1	16	2.26	0.152		0.019
S1 R - NAcC L	sex	1	16	0.241	0.63		0.004
S1 R - NAcC L	treatmt	1	16	0.566	0.463		0.01
S1 R - NAcC L	time	1	16	0.204	0.657		0.003
S1 R - NAcC L	sex:treatmt	1	16	0.368	0.553		0.006
S1 R - NAcC L	sex:time	1	16	2.646	0.123		0.032
S1 R - NAcC L	treatmt:time	1	16	0.218	0.647		0.004
S1 R - NAcC L	sex:treatmt:time	1	16	8.74E-05	0.993		1.42E-06
S1 R - NAcC R	sex	1	16	0.106	0.749		0.002
S1 R - NAcC R	treatmt	1	16	2.079	0.169		0.032
S1 R - NAcC R	time	1	16	0.263	0.615		0.004
S1 R - NAcC R	sex:treatmt	1	16	0.155	0.699		0.002
S1 R - NAcC R	sex:time	1	16	0.833	0.375		0.014
S1 R - NAcC R	treatmt:time	1	16	0.355	0.56		0.004
S1 R - NAcC R	sex:treatmt:time	1	16	0.048	0.829		0.00059
S1 R - NAcSh L	sex	1	16	0.1	0.756		0.002
S1 R - NAcSh L	treatmt	1	16	0.002	0.968		3.31E-05
S1 R - NAcSh L	time	1	16	1.712	0.209		0.022
S1 R - NAcSh L	sex:treatmt	1	16	2.231	0.155		0.041
S1 R - NAcSh L	sex:time	1	16	2.576	0.128		0.033
S1 R - NAcSh L	treatmt:time	1	16	4.969	0.04	*	0.046
S1 R - NAcSh L	sex:treatmt:time	1	16	0.529	0.477		0.005
S1 R - NAcSh R	sex	1	16	0.149	0.704		0.003
S1 R - NAcSh R	treatmt	1	16	1.184	0.293		0.028
S1 R - NAcSh R	time	1	16	8.586	0.01	*	0.065
S1 R - NAcSh R	sex:treatmt	1	16	0.761	0.396		0.018
S1 R - NAcSh R	sex:time	1	16	0.12	0.734		0.000967
S1 R - NAcSh R	treatmt:time	1	16	0.836	0.374		0.008
S1 R - NAcSh R	sex:treatmt:time	1	16	0.712	0.411		0.007
S1 R - PrL L	sex	1	16	0.048	0.829		0.001
S1 R - PrL L	treatmt	1	16	0.613	0.445		0.016
S1 R - PrL L	time	1	16	0.203	0.658		0.002
S1 R - PrL L	sex:treatmt	1	16	0.62	0.442		0.017
S1 R - PrL L	sex:time	1	16	0.078	0.784		0.000793
S1 R - PrL L	treatmt:time	1	16	0.615	0.444		0.001
S1 R - PrL L	sex:treatmt:time	1	16	8.182	0.011	*	0.02
S1 R - PrL R	sex	1	16	0.454	0.51		0.01
S1 R - PrL R	treatmt	1	16	0.283	0.602		0.009
S1 R - PrL R	time	1	16	24.81	0.00014	*	0.075
S1 R - PrL R	sex:treatmt	1	16	0.513	0.484		0.016
S1 R - PrL R	sex:time	1	16	0.177	0.679		0.000579
S1 R - PrL R	treatmt:time	1	16	3.242	0.091		0.021
S1 R - PrL R	sex:treatmt:time	1	16	0.312	0.584		0.002
S1 R - S1 L	sex	1	16	6.857	0.019	*	0.12
S1 R - S1 L	treatmt	1	16	1.04	0.323		0.022
S1 R - S1 L	time	1	16	2.276	0.151		0.024
S1 R - S1 L	sex:treatmt	1	16	0.434	0.519		0.009
S1 R - S1 L	sex:time	1	16	2.178	0.159		0.023
S1 R - S1 L	treatmt:time	1	16	2.86	0.11		0.027
S1 R - S1 L	sex:treatmt:time	1	16	4.251	0.056		0.04

Figure 3B. Kendall's coefficient of concordance

Sample size: n = 18 rats, 9 females

Tukey's HSD post-hoc test

Hedge's g effect size

IL - males

group1	group2	estimate	conf.low	conf.high	p.adj	p.adj.signif
baseline	nalket	-0.22977	-0.60355	0.144003	0.293	ns
baseline	salket	-0.38127	-0.75504	-0.00749	0.045	*
nalket	salket	-0.15149	-0.52527	0.22228	0.577	ns

effsize	n1	n2	magnitude
0.387297	9	9	small
0.722569	9	9	moderate
0.454051	9	9	small

IL - females

group1	group2	estimate	conf.low	conf.high	p.adj	p.adj.signif
baseline	nalket	-0.08693	-0.44347	0.26961	0.817	ns
baseline	salket	-0.04137	-0.39791	0.315166	0.955	ns
nalket	salket	0.045556	-0.31098	0.402096	0.946	ns

effsize	n1	n2	magnitude
0.15991	9	9	negligible
0.074751	9	9	negligible
-0.08466	9	9	negligible

CPu - males

group1	group2	estimate	conf.low	conf.high	p.adj	p.adj.signif
baseline	nalket	-0.04658	-0.39431	0.301153	0.94	ns
baseline	salket	-0.38153	-0.72926	-0.0338	0.0296	*
nalket	salket	-0.33495	-0.68268	0.012783	0.0606	ns

effsize	n1	n2	magnitude
0.091261	9	9	negligible
0.818843	9	9	large
0.662483	9	9	moderate

CPu - females

group1	group2	estimate	conf.low	conf.high	p.adj	p.adj.signif
baseline	nalket	0.109053	-0.35527	0.573372	0.829	ns
baseline	salket	-0.12662	-0.59094	0.337695	0.777	ns
nalket	salket	-0.23568	-0.7	0.228642	0.427	ns

effsize	n1	n2	magnitude
-0.2041	9	9	negligible
0.484049	9	9	negligible
0.567182	9	9	small

Figure 5B. Time fraction per brain state

Sample size: n = 18 rats, 9 females

VEH+KET - M - Brain State #1							
Two-tailed paired t-test							
**group1**	**group2**	**t**	**df**	**p**	**CI_Lower**	**CI_Upper**	**Significance**
Pre-KET	Post-KET	4.612352437	8	0.001727352	0.052900772	0.158686529	**

VEH+KET - F - Brain State #1							
Two-tailed paired t-test							
**group1**	**group2**	**t**	**df**	**p**	**CI_Lower**	**CI_Upper**	**Significance**
Pre-KET	Post-KET	0.175032965	8	0.865402577	-0.144399921	0.168121261	n.s.

VEH+KET - M - Brain State #2							
Two-tailed paired t-test							
**group1**	**group2**	**t**	**df**	**p**	**CI_Lower**	**CI_Upper**	**Significance**
Pre-KET	Post-KET	-0.839156646	8	0.425744612	-0.191234035	0.089188179	n.s.

VEH+KET - F - Brain State #2							
Two-tailed paired t-test							
**group1**	**group2**	**t**	**df**	**p**	**CI_Lower**	**CI_Upper**	**Significance**
Pre-KET	Post-KET	-0.277716226	8	0.788272181	-0.120928505	0.094932032	n.s.

VEH+KET - M - Brain State #3							
Two-tailed paired t-test							
**group1**	**group2**	**t**	**df**	**p**	**CI_Lower**	**CI_Upper**	**Significance**
Pre-KET	Post-KET	1.778044607	8	0.113287903	-0.026988404	0.208769709	n.s.

VEH+KET - F - Brain State #3							
Two-tailed paired t-test							
**group1**	**group2**	**t**	**df**	**p**	**CI_Lower**	**CI_Upper**	**Significance**
Pre-KET	Post-KET	1.49730971	8	0.172686604	-0.039121406	0.183989131	n.s.

VEH+KET - M - Brain State #4							
Two-tailed paired t-test							
**group1**	**group2**	**t**	**df**	**p**	**CI_Lower**	**CI_Upper**	**Significance**
Pre-KET	Post-KET	-0.136004338	8	0.895177931	-0.068876427	0.061204469	n.s.

VEH+KET - F - Brain State #4							
Two-tailed paired t-test							
**group1**	**group2**	**t**	**df**	**p**	**CI_Lower**	**CI_Upper**	**Significance**
Pre-KET	Post-KET	-0.582422914	8	0.57632436	-0.116592346	0.069572946	n.s.

VEH+KET - M - Brain State #5							
Two-tailed paired t-test							
**group1**	**group2**	**t**	**df**	**p**	**CI_Lower**	**CI_Upper**	**Significance**
Pre-KET	Post-KET	-2.47415895	8	0.038460267	-0.270484979	-0.009515021	*

VEH+KET - F - Brain State #5
Two-tailed paired t-test

group1	group2	t	df	p	CI_Lower	CI_Upper	Significance
Pre-KET	Post-KET	-0.698737223	8	0.504518797	-0.197644425	0.105722026	n.s.

NTX+KET - M - Brain State #1

Two-tailed paired t-test

group1	group2	t	df	p	CI_Lower	CI_Upper	Significance
Pre-KET	Post-KET	1.150639078	8	0.283110201	-0.084676171	0.253335783	n.s.

NTX+KET - F - Brain State #1

Two-tailed paired t-test

group1	group2	t	df	p	CI_Lower	CI_Upper	Significance
Pre-KET	Post-KET	1.479029701	8	0.17739406	-0.056233799	0.257380183	n.s.

NTX+KET - M - Brain State #2

Two-tailed paired t-test

group1	group2	t	df	p	CI_Lower	CI_Upper	Significance
Pre-KET	Post-KET	-0.207708599	8	0.840646584	-0.158373318	0.132200479	n.s.

NTX+KET - F - Brain State #2

Two-tailed paired t-test

group1	group2	t	df	p	CI_Lower	CI_Upper	Significance
Pre-KET	Post-KET	-1.544171609	8	0.16112516	-0.182318706	0.036075319	n.s.

NTX+KET - M - Brain State #3

Two-tailed paired t-test

group1	group2	t	df	p	CI_Lower	CI_Upper	Significance
Pre-KET	Post-KET	-1.088094319	8	0.308244394	-0.156542918	0.056172547	n.s.

NTX+KET - F - Brain State #3

Two-tailed paired t-test

group1	group2	t	df	p	CI_Lower	CI_Upper	Significance
Pre-KET	Post-KET	1.320996029	8	0.223035385	-0.014682972	0.054065688	n.s.

NTX+KET - M - Brain State #4

Two-tailed paired t-test

group1	group2	t	df	p	CI_Lower	CI_Upper	Significance
Pre-KET	Post-KET	-1.78643064	8	0.111847403	-0.186459388	0.023672792	n.s.

NTX+KET - F - Brain State #4

Two-tailed paired t-test

group1	group2	t	df	p	CI_Lower	CI_Upper	Significance
Pre-KET	Post-KET	-0.717516425	8	0.493471253	-0.149492036	0.078539655	n.s.

NTX+KET - M - Brain State #5

Two-tailed paired t-test

group1	group2	t	df	p	CI_Lower	CI_Upper	Significance
Pre-KET	Post-KET	0.677437914	8	0.517236117	-0.14943433	0.273755317	n.s.

NTX+KET - F - Brain State #5

Two-tailed paired t-test

group1	group2	t	df	p	CI_Lower	CI_Upper	Significance
Pre-KET	Post-KET	-0.089039551	8	0.931239184	-0.26471687	0.24503433	n.s.

Figure 5C. Delta time fraction (post-ketamine - pre-ketamine) per brain state

Sample size: n = 18 rats, 9 females

**VEH+KET vs NXT+KET - M - Brain State #1**							
Two-tailed paired t-test							
**group1**	**group2**	**t**	**df**	**p**	**CI_Lower**	**CI_Upper**	**Significance**
VEH-KET	NXT-KET	-0.294432637	8	0.775922655	-0.18956924	0.146641551	n.s.

**M vs F - VEH+KET - Brain State #1**							
Two-tailed paired t-test							
**group1**	**group2**	**t**	**df**	**p**	**CI_Lower**	**CI_Upper**	**Significance**
Males	Females	-1.485446254	8	0.175728811	-0.239754373	0.051888412	n.s.

**VEH+KET vs NXT+KET - M - Brain State #2**							
Two-tailed paired t-test							
**group1**	**group2**	**t**	**df**	**p**	**CI_Lower**	**CI_Upper**	**Significance**
VEH-KET	NXT-KET	0.383620604	8	0.711253699	-0.190105842	0.265978858	n.s.

**M vs F - VEH+KET - Brain State #2**							
Two-tailed paired t-test							
**group1**	**group2**	**t**	**df**	**p**	**CI_Lower**	**CI_Upper**	**Significance**
Males	Females	0.516569272	8	0.619429317	-0.131720395	0.207769777	n.s.

**VEH+KET vs NXT+KET - M - Brain State #3**							
Two-tailed paired t-test							
**group1**	**group2**	**t**	**df**	**p**	**CI_Lower**	**CI_Upper**	**Significance**
VEH-KET	NXT-KET	-2.297453682	8	0.050671844	-0.282676719	0.000525043	n.s.

**M vs F - VEH+KET - Brain State #3**							
Two-tailed paired t-test							
**group1**	**group2**	**t**	**df**	**p**	**CI_Lower**	**CI_Upper**	**Significance**
Males	Females	-0.376781231	8	0.716133712	-0.131417391	0.094503811	n.s.

**VEH+KET vs NXT+KET - M - Brain State #4**							
Two-tailed paired t-test							
**group1**	**group2**	**t**	**df**	**p**	**CI_Lower**	**CI_Upper**	**Significance**
VEH-KET	NXT-KET	-1.431130481	8	0.190273643	-0.202526706	0.047412068	n.s.

**M vs F - VEH+KET - Brain State #4**							
Two-tailed paired t-test							
**group1**	**group2**	**t**	**df**	**p**	**CI_Lower**	**CI_Upper**	**Significance**
Males	Females	-0.310751017	8	0.763932225	-0.165667396	0.126319953	n.s.

**VEH+KET vs NXT+KET - M - Brain State #5**							
Two-tailed paired t-test							
**group1**	**group2**	**t**	**df**	**p**	**CI_Lower**	**CI_Upper**	**Significance**
VEH-KET	NXT-KET	2.598722539	8	0.031680647	0.022771226	0.381549762	*

**M vs F - VEH+KET - Brain State #5**
Two-tailed paired t-test

group1	group2	t	df	p	CI_Lower	CI_Upper	Significance
Males	Females	1.628171031	8	0.142138604	-0.039149826	0.227227427	n.s.

VEH+KET vs NXT+KET - F - Brain State #1

Two-tailed paired t-test

group1	group2	t	df	p	CI_Lower	CI_Upper	Significance
VEH-KET	NXT-KET	0.822904574	8	0.434404047	-0.159884276	0.337309321	n.s.

M vs F - NTX+KET - Brain State #1

Two-tailed paired t-test

group1	group2	t	df	p	CI_Lower	CI_Upper	Significance
Males	Females	0.156567199	8	0.879464987	-0.222997758	0.25548453	n.s.

VEH+KET vs NXT+KET - F - Brain State #2

Two-tailed paired t-test

group1	group2	t	df	p	CI_Lower	CI_Upper	Significance
VEH-KET	NXT-KET	-0.797585892	8	0.448135206	-0.233954189	0.113707276	n.s.

M vs F - NTX+KET - Brain State #2

Two-tailed paired t-test

group1	group2	t	df	p	CI_Lower	CI_Upper	Significance
Males	Females	-0.706887178	8	0.499705194	-0.255882069	0.135811522	n.s.

VEH+KET vs NXT+KET - F - Brain State #3

Two-tailed paired t-test

group1	group2	t	df	p	CI_Lower	CI_Upper	Significance
VEH-KET	NXT-KET	-0.948643462	8	0.370574123	-0.180951297	0.075466289	n.s.

M vs F - NTX+KET - Brain State #3

Two-tailed paired t-test

group1	group2	t	df	p	CI_Lower	CI_Upper	Significance
Males	Females	1.314648216	8	0.225063482	-0.052692822	0.192445909	n.s.

VEH+KET vs NXT+KET - F - Brain State #4

Two-tailed paired t-test

group1	group2	t	df	p	CI_Lower	CI_Upper	Significance
VEH-KET	NXT-KET	-0.20805837	8	0.84038255	-0.144596464	0.120663483	n.s.

M vs F - NTX+KET - Brain State #4

Two-tailed paired t-test

group1	group2	t	df	p	CI_Lower	CI_Upper	Significance
Males	Females	0.591721503	8	0.570376145	-0.133026939	0.224861154	n.s.

VEH+KET vs NXT+KET - F - Brain State #5

Two-tailed paired t-test

group1	group2	t	df	p	CI_Lower	CI_Upper	Significance

VEH-KET	NXT-KET	0.259357243	8	0.801908846	-0.285030564	0.357270423	n.s.

M vs F - NTX+KET - Brain State #5

Two-tailed paired t-test

group1	group2	t	df	p	CI_Lower	CI_Upper	Significance
Males	Females	-0.495362016	8	0.63366318	-0.407183629	0.263180102	n.s.

Figure 5D. Dwell time per brain state

Sample size: n = 18 rats, 9 females

VEH+KET - M - Brain State #1

Two-tailed paired t-test

group1	group2	t	df	p	CI_Lower	CI_Upper	Significance
Pre-KET	Post-KET	3.450121773	8	0.008693241	2.00061512	10.0652172	**

VEH+KET - F - Brain State #1

Two-tailed paired t-test

group1	group2	t	df	p	CI_Lower	CI_Upper	Significance
Pre-KET	Post-KET	-0.131928124	8	0.898298835	-6.341720577	5.655359177	n.s.

VEH+KET - M - Brain State #2

Two-tailed paired t-test

group1	group2	t	df	p	CI_Lower	CI_Upper	Significance
Pre-KET	Post-KET	-1.36906052	8	0.208181489	-5.682008388	1.448606209	n.s.

VEH+KET - F - Brain State #2

Two-tailed paired t-test

group1	group2	t	df	p	CI_Lower	CI_Upper	Significance
Pre-KET	Post-KET	-0.903580081	8	0.39261321	-3.392685904	1.482430122	n.s.

VEH+KET - M - Brain State #3

Two-tailed paired t-test

group1	group2	t	df	p	CI_Lower	CI_Upper	Significance
Pre-KET	Post-KET	1.791907512	8	0.110915944	-0.831298327	6.626355719	n.s.

VEH+KET - F - Brain State #3

Two-tailed paired t-test

group1	group2	t	df	p	CI_Lower	CI_Upper	Significance
Pre-KET	Post-KET	0.507962038	8	0.62518622	-3.986570566	6.239050097	n.s.

VEH+KET - M - Brain State #4

Two-tailed paired t-test

group1	group2	t	df	p	CI_Lower	CI_Upper	Significance
Pre-KET	Post-KET	-0.188012462	8	0.855548301	-4.242931252	3.603221035	n.s.

VEH+KET - F - Brain State #4

Two-tailed paired t-test

group1	group2	t	df	p	CI_Lower	CI_Upper	Significance
Pre-KET	Post-KET	-0.931257384	8	0.378966407	-5.302297599	2.251692166	n.s.

VEH+KET - M - Brain State #5

Two-tailed paired t-test

group1	group2	t	df	p	CI_Lower	CI_Upper	Significance
Pre-KET	Post-KET	-1.57301655	8	0.154360194	-15.13456702	2.859858357	n.s.

VEH+KET - F - Brain State #5

Two-tailed paired t-test

group1	group2	t	df	p	CI_Lower	CI_Upper	Significance
Pre-KET	Post-KET	-1.967348392	8	0.084690271	-13.29829809	1.053866955	n.s.

NTX+KET - M - Brain State #1

Two-tailed paired t-test

group1	group2	t	df	p	CI_Lower	CI_Upper	Significance
Pre-KET	Post-KET	0.537840234	8	0.605322204	-3.056063235	4.915250344	n.s.

NTX+KET - F - Brain State #1

Two-tailed paired t-test

group1	group2	t	df	p	CI_Lower	CI_Upper	Significance
Pre-KET	Post-KET	0.089234338	8	0.931089207	-4.319781245	4.667560091	n.s.

NTX+KET - M - Brain State #2

Two-tailed paired t-test

group1	group2	t	df	p	CI_Lower	CI_Upper	Significance
Pre-KET	Post-KET	-0.179783336	8	0.861792998	-4.311605249	3.68793586	n.s.

NTX+KET - F - Brain State #2

Two-tailed paired t-test

group1	group2	t	df	p	CI_Lower	CI_Upper	Significance
Pre-KET	Post-KET	-2.207384754	8	0.0583184	-8.719871075	0.190532725	n.s.

NTX+KET - M - Brain State #3

Two-tailed paired t-test

group1	group2	t	df	p	CI_Lower	CI_Upper	Significance
Pre-KET	Post-KET	-1.872612557	8	0.098012603	-5.803097866	0.601877111	n.s.

NTX+KET - F - Brain State #3

Two-tailed paired t-test

group1	group2	t	df	p	CI_Lower	CI_Upper	Significance
Pre-KET	Post-KET	0.252785785	8	0.806807881	-3.423667889	4.266690331	n.s.

NTX+KET - M - Brain State #4

Two-tailed paired t-test

group1	group2	t	df	p	CI_Lower	CI_Upper	Significance
Pre-KET	Post-KET	-0.45576621	8	0.66067464	-5.545568187	3.71523309	n.s.

NTX+KET - F - Brain State #4

Two-tailed paired t-test

group1	group2	t	df	p	CI_Lower	CI_Upper	Significance
Pre-KET	Post-KET	-1.105454809	8	0.301096006	-7.845369216	2.760916337	n.s.

NTX+KET - M - Brain State #5

Two-tailed paired t-test

group1	group2	t	df	p	CI_Lower	CI_Upper	Significance
Pre-KET	Post-KET	1.76751185	8	0.115121859	-2.461826006	18.62289928	n.s.

NTX+KET - F - Brain State #5

Two-tailed paired t-test

group1	group2	t	df	p	CI_Lower	CI_Upper	Significance
Pre-KET	Post-KET	-0.253028801	8	0.806626549	-14.42737212	11.57430945	n.s.

Figure 5E. Delta dwell time (post-ketamine - pre-ketamine) per brain state

Sample size: n = 18 rats, 9 females

VEH+KET vs NXT+KET - M - Brain State #1							
Two-tailed paired t-test							
**group1**	**group2**	**t**	**df**	**p**	**CI_Lower**	**CI_Upper**	**Significance**
VEH-KET	NXT-KET	-1.995505706	8	0.081078732	-11.00071643	0.794071221	n.s.

M vs F - VEH+KET - Brain State #1							
Two-tailed paired t-test							
**group1**	**group2**	**t**	**df**	**p**	**CI_Lower**	**CI_Upper**	**Significance**
Males	Females	-1.831114611	8	0.104458537	-14.40579944	1.653605725	n.s.

VEH+KET vs NXT+KET - M - Brain State #2							
Two-tailed paired t-test							
**group1**	**group2**	**t**	**df**	**p**	**CI_Lower**	**CI_Upper**	**Significance**
VEH-KET	NXT-KET	-1.995505706	8	0.081078732	-11.00071643	0.794071221	n.s.

M vs F - VEH+KET - Brain State #2							
Two-tailed paired t-test							
**group1**	**group2**	**t**	**df**	**p**	**CI_Lower**	**CI_Upper**	**Significance**
Males	Females	0.621074696	8	0.551831314	-3.151261656	5.474408052	n.s.

VEH+KET vs NXT+KET - M - Brain State #3							
Two-tailed paired t-test							
**group1**	**group2**	**t**	**df**	**p**	**CI_Lower**	**CI_Upper**	**Significance**
VEH-KET	NXT-KET	-2.272946735	8	0.05264774	-11.07624227	0.079964119	n.s.

M vs F - VEH+KET - Brain State #3							
Two-tailed paired t-test							
**group1**	**group2**	**t**	**df**	**p**	**CI_Lower**	**CI_Upper**	**Significance**
Males	Females	-0.59782547	8	0.566490601	-8.603717126	5.061139266	n.s.

VEH+KET vs NXT+KET - M - Brain State #4							
Two-tailed paired t-test							
**group1**	**group2**	**t**	**df**	**p**	**CI_Lower**	**CI_Upper**	**Significance**
VEH-KET	NXT-KET	-0.386578116	8	0.709147825	-4.146452281	2.955827401	n.s.

M vs F - VEH+KET - Brain State #4							
Two-tailed paired t-test							
**group1**	**group2**	**t**	**df**	**p**	**CI_Lower**	**CI_Upper**	**Significance**
Males	Females	-0.523005951	8	0.61514233	-6.520429522	4.109534306	n.s.

VEH+KET vs NXT+KET - M - Brain State #5							
Two-tailed paired t-test							
**group1**	**group2**	**t**	**df**	**p**	**CI_Lower**	**CI_Upper**	**Significance**
VEH-KET	NXT-KET	2.539314474	8	0.034747841	1.306329323	27.12945261	*

M vs F - VEH+KET - Brain State #5							
Two-tailed paired t-test							
**group1**	**group2**	**t**	**df**	**p**	**CI_Lower**	**CI_Upper**	**Significance**
Males	Females	0.004511545	8	0.996510793	-7.722797441	7.753074965	n.s.

VEH+KET vs NXT+KET - F - Brain State #1							
Two-tailed paired t-test							
**group1**	**group2**	**t**	**df**	**p**	**CI_Lower**	**CI_Upper**	**Significance**

VEH-KET	NXT-KET	0.12217742	8	0.905771881	-9.242227816	10.27636806	n.s.

M vs F - NTX+KET - Brain State #1

Two-tailed paired t-test

group1	group2	t	df	p	CI_Lower	CI_Upper	Significance
Males	Females	-0.289285048	8	0.779718491	-6.779717011	5.268308747	n.s.

VEH+KET vs NXT+KET - F - Brain State #2

Two-tailed paired t-test

group1	group2	t	df	p	CI_Lower	CI_Upper	Significance
VEH-KET	NXT-KET	-1.311604976	8	0.226041352	-9.128226043	2.509143476	n.s.

M vs F - NTX+KET - Brain State #2

Two-tailed paired t-test

group1	group2	t	df	p	CI_Lower	CI_Upper	Significance
Males	Females	-1.445651294	8	0.18628458	-10.25812587	2.352456909	n.s.

VEH+KET vs NXT+KET - F - Brain State #3

Two-tailed paired t-test

group1	group2	t	df	p	CI_Lower	CI_Upper	Significance
VEH-KET	NXT-KET	-0.261894699	8	0.800019646	-6.909920715	5.500463626	n.s.

M vs F - NTX+KET - Brain State #3

Two-tailed paired t-test

group1	group2	t	df	p	CI_Lower	CI_Upper	Significance
Males	Females	1.889958751	8	0.095431974	-0.665273643	6.709516839	n.s.

VEH+KET vs NXT+KET - F - Brain State #4

Two-tailed paired t-test

group1	group2	t	df	p	CI_Lower	CI_Upper	Significance
VEH-KET	NXT-KET	-0.406986393	8	0.694689558	-6.778861592	4.745014146	n.s.

M vs F - NTX+KET - Brain State #4

Two-tailed paired t-test

group1	group2	t	df	p	CI_Lower	CI_Upper	Significance
Males	Females	-0.487180098	8	0.63919885	-9.328532214	6.074414432	n.s.

VEH+KET vs NXT+KET - F - Brain State #5

Two-tailed paired t-test

group1	group2	t	df	p	CI_Lower	CI_Upper	Significance
VEH-KET	NXT-KET	0.593203037	8	0.569431663	-13.55821296	22.94958143	n.s.

M vs F - NTX+KET - Brain State #5

Two-tailed paired t-test

group1	group2	t	df	p	CI_Lower	CI_Upper	Significance
Males	Females	-1.46839683	8	0.18018445	-24.43718604	5.4230501	n.s.

Figure 5F. Markov entropy

Sample size: n = 18 rats, 9 females

**VEH+KET - M**							
Two-tailed paired t-test							
**group1**	**group2**	**t**	**df**	**p**	**CI_Lower**	**CI_Upper**	**Significance**
Pre-KET	Post-KET	-2.766895733	8	0.02441341	-0.289902908	-0.026338745	*

**NTX+KET - M**							
Two-tailed paired t-test							
**group1**	**group2**	**t**	**df**	**p**	**CI_Lower**	**CI_Upper**	**Significance**
Pre-KET	Post-KET	-0.345319292	8	0.738758464	-0.119762237	0.088565582	n.s.

**VEH+KET - F**							
Two-tailed paired t-test							
**group1**	**group2**	**t**	**df**	**p**	**CI_Lower**	**CI_Upper**	**Significance**
Pre-KET	Post-KET	-0.177336393	8	0.863651887	-0.247606463	0.212243099	n.s.

**NTX+KET - F**							
Two-tailed paired t-test							
**group1**	**group2**	**t**	**df**	**p**	**CI_Lower**	**CI_Upper**	**Significance**
Pre-KET	Post-KET	1.032161067	8	0.332188125	-0.047015502	0.123206317	n.s.

**VEH+KET - M vs F**							
Two-tailed paired t-test							
**group1**	**group2**	**t**	**df**	**p**	**CI_Lower**	**CI_Upper**	**Significance**
Males	Females	-1.03985866	8	0.328809687	-0.451878824	0.171000535	n.s.

**NTX+KET - M vs F**							
Two-tailed paired t-test							
**group1**	**group2**	**t**	**df**	**p**	**CI_Lower**	**CI_Upper**	**Significance**
Males	Females	-0.789585714	8	0.452534764	-0.210507584	0.103120114	n.s.

**NTX+KET vs VEH+KET - M**							
Two-tailed paired t-test							
**group1**	**group2**	**t**	**df**	**p**	**CI_Lower**	**CI_Upper**	**Significance**
VEH-KET	NXT-KET	1.63683836	8	0.14030055	-0.058265483	0.343310481	n.s.

**NTX+KET vs VEH+KET - F**							
Two-tailed paired t-test							
**group1**	**group2**	**t**	**df**	**p**	**CI_Lower**	**CI_Upper**	**Significance**
VEH-KET	NXT-KET	0.56745163	8	0.585974473	-0.170889277	0.282443455	n.s.

Supplementary Figure 1. Test-test reliability of functional ultrasound imaging functional connectivity

Sample size: n = 18 rats, 9 females

Two-way mixed-effects ANOVA with within-subjects factor of ROI and between-subjects factor of sex

Effect	DFn	DFd	F	p	p<.05	ges
roi	230	3910	17.628	0	*	0.258
session	2	34	1.265	0.295		0.013
roi:session	460	7820	0.755	1		0.019

Supplementary Fig. 2. Functional connectivity post ketamine administration

See Fig 1E-F sheet

Supplementary Figure 3: Functional connectivity effects of naltrexone administration.

Sample size: n = 18 rats, 9 females

Three-way mixed-effects ANOVA with within-subjects factor of ROI and time and between-subjects factor of sex						
**Effect**	**DFn**	**DFd**	**F**	**p**	**p<.05**	**ges**
**sex**	1	16	0.014	0.909		7.48E-05
**time**	1.84	29.5	1.411	0.259		0.008
**roi**	230	3680	9.308	2.65E-229	*	0.24
**sex:time**	1.84	29.5	1.365	0.27		0.008
**sex:roi**	230	3680	1.032	0.36		0.034
**time:roi**	690	11040	1.16	0.003	*	0.019
**sex:time:roi**	690	11040	1.048	0.192		0.018

Supplementary Figure 4. ROI-dependent effects of treatment and time in gonadectomized male rats

Sample size: n = 7 gonadectomized male rats

Two-way ANOVA with within-subjects factors of treatment and time, stratified by ROI

roi	Effect	DFn	DFd	F	p	p<.05	ges
AI R - AI L	treatmt	1	6	1.189	0.317		0.046
AI R - AI L	time	2	12	0.242	0.789		0.007
AI R - AI L	treatmt:time	2	12	1.084	0.369		0.032
AI R - Cg1 L	treatmt	1	6	0.688	0.439		0.015
AI R - Cg1 L	time	2	12	3.631	0.058		0.16
AI R - Cg1 L	treatmt:time	2	12	0.278	0.762		0.015
AI R - Cg1 R	treatmt	1	6	0.001	0.972		3.07E-05
AI R - Cg1 R	time	2	12	2.287	0.144		0.089
AI R - Cg1 R	treatmt:time	2	12	0.448	0.649		0.03
AI R - CPu L	treatmt	1	6	4.632	0.075		0.124
AI R - CPu L	time	2	12	9.275	0.004	*	0.15
AI R - CPu L	treatmt:time	2	12	0.382	0.69		0.015
AI R - CPu R	treatmt	1	6	0.537	0.491		0.014
AI R - CPu R	time	2	12	0.963	0.409		0.036
AI R - CPu R	treatmt:time	2	12	0.474	0.634		0.013
AI R - GIDI L	treatmt	1	6	0.494	0.509		0.011
AI R - GIDI L	time	1.18	7.07	0.507	0.529		0.028
AI R - GIDI L	treatmt:time	2	12	0.52	0.607		0.029
AI R - GIDI R	treatmt	1	6	0.046	0.837		0.001
AI R - GIDI R	time	2	12	3.127	0.081		0.102
AI R - GIDI R	treatmt:time	2	12	0.38	0.692		0.008
AI R - IL L	treatmt	1	6	1.671	0.244		0.039
AI R - IL L	time	2	12	0.037	0.964		0.001
AI R - IL L	treatmt:time	2	12	0.377	0.694		0.024
AI R - IL R	treatmt	1	6	1.333	0.292		0.009
AI R - IL R	time	2	12	0.88	0.44		0.029
AI R - IL R	treatmt:time	2	12	0.426	0.663		0.025
AI R - M1 L	treatmt	1	6	1.7	0.24		0.035
AI R - M1 L	time	1.15	6.9	1.481	0.27		0.067
AI R - M1 L	treatmt:time	2	12	0.074	0.929		0.003
AI R - M1 R	treatmt	1	6	0.613	0.463		0.025
AI R - M1 R	time	2	12	0.13	0.879		0.008
AI R - M1 R	treatmt:time	2	12	0.779	0.481		0.029
AI R - M2 L	treatmt	1	6	3.598	0.107		0.102
AI R - M2 L	time	2	12	2.109	0.164		0.061
AI R - M2 L	treatmt:time	2	12	0.015	0.985		0.00091
AI R - M2 R	treatmt	1	6	1.64	0.248		0.072
AI R - M2 R	time	2	12	2.152	0.159		0.091
AI R - M2 R	treatmt:time	2	12	1.392	0.286		0.043
AI R - NAcC L	treatmt	1	6	2.263	0.183		0.029
AI R - NAcC L	time	2	12	10.74	0.002	*	0.314
AI R - NAcC L	treatmt:time	2	12	0.311	0.738		0.018
AI R - NAcC R	treatmt	1	6	3.571	0.108		0.104
AI R - NAcC R	time	2	12	6.898	0.01	*	0.144
AI R - NAcC R	treatmt:time	2	12	0.093	0.912		0.002
AI R - NAcSh L	treatmt	1	6	0.311	0.597		0.015
AI R - NAcSh L	time	2	12	4.591	0.033	*	0.162
AI R - NAcSh L	treatmt:time	2	12	0.382	0.69		0.015
AI R - NAcSh R	treatmt	1	6	0.684	0.44		0.023
AI R - NAcSh R	time	2	12	5.795	0.017	*	0.119
AI R - NAcSh R	treatmt:time	2	12	0.423	0.665		0.007
AI R - PrL L	treatmt	1	6	4.634	0.075		0.028
AI R - PrL L	time	2	12	0.77	0.484		0.038
AI R - PrL L	treatmt:time	2	12	0.286	0.756		0.016
AI R - PrL R	treatmt	1	6	5.701	0.054		0.054
AI R - PrL R	time	1.09	6.55	0.016	0.919		0.00054
AI R - PrL R	treatmt:time	2	12	0.081	0.923		0.005
AI R - S1 L	treatmt	1	6	0.317	0.594		0.007
AI R - S1 L	time	2	12	0.288	0.754		0.019
AI R - S1 L	treatmt:time	2	12	0.345	0.715		0.021
AI R - S1 R	treatmt	1	6	2.02	0.205		0.043
AI R - S1 R	time	2	12	1.469	0.269		0.065
AI R - S1 R	treatmt:time	2	12	1.449	0.273		0.054
Cg1 L - AI L	treatmt	1	6	2.812	0.145		0.011
Cg1 L - AI L	time	2	12	0.953	0.413		0.032
Cg1 L - AI L	treatmt:time	2	12	0.598	0.566		0.02
Cg1 L - GIDI L	treatmt	1	6	0.097	0.766		0.002
Cg1 L - GIDI L	time	2	12	2.493	0.124		0.135
Cg1 L - GIDI L	treatmt:time	2	12	0.57	0.58		0.027
Cg1 L - M1 L	treatmt	1	6	0.062	0.812		0.001
Cg1 L - M1 L	time	2	12	1.919	0.189		0.066
Cg1 L - M1 L	treatmt:time	2	12	0.097	0.908		0.004
Cg1 L - M2 L	treatmt	1	6	1.865	0.221		0.037
Cg1 L - M2 L	time	1.13	6.78	0.334	0.608		0.016
Cg1 L - M2 L	treatmt:time	2	12	0.153	0.86		0.011
Cg1 L - S1 L	treatmt	1	6	0.356	0.572		0.012
Cg1 L - S1 L	time	2	12	1.287	0.312		0.057
Cg1 L - S1 L	treatmt:time	2	12	0.117	0.891		0.004
Cg1 R - AI L	treatmt	1	6	0.763	0.416		0.009

Sample size: n = 9 intact male rats

Two-way ANOVA with within-subjects factors of treatment and time, stratified by ROI

roi	Effect	DFn	DFd	F	p	p<.05	ges
AI R - AI L	treatmt	1	8	0.665	0.438		0.026
AI R - AI L	time	1	8	7.447	0.026	*	0.098
AI R - AI L	treatmt:time	1	8	2.65	0.142		0.026
AI R - Cg1 L	treatmt	1	8	0.267	0.619		0.008
AI R - Cg1 L	time	1	8	1.811	0.215		0.051
AI R - Cg1 L	treatmt:time	1	8	4.022	0.08		0.061
AI R - Cg1 R	treatmt	1	8	0.302	0.598		0.009
AI R - Cg1 R	time	1	8	0.728	0.418		0.018
AI R - Cg1 R	treatmt:time	1	8	1.273	0.292		0.039
AI R - CPu L	treatmt	1	8	0.063	0.808		0.002
AI R - CPu L	time	1	8	9.719	0.014	*	0.27
AI R - CPu L	treatmt:time	1	8	0.273	0.616		0.006
AI R - CPu R	treatmt	1	8	0.197	0.669		0.008
AI R - CPu R	time	1	8	7.73	0.024	*	0.082
AI R - CPu R	treatmt:time	1	8	0.076	0.789		0.003
AI R - GIDI L	treatmt	1	8	0.002	0.966		0.0001
AI R - GIDI L	time	1	8	5.103	0.054		0.109
AI R - GIDI L	treatmt:time	1	8	0.262	0.622		0.004
AI R - GIDI R	treatmt	1	8	0.448	0.522		0.023
AI R - GIDI R	time	1	8	3.666	0.092		0.081
AI R - GIDI R	treatmt:time	1	8	2.236	0.173		0.027
AI R - IL L	treatmt	1	8	3.721	0.09		0.103
AI R - IL L	time	1	8	4.434	0.068		0.142
AI R - IL L	treatmt:time	1	8	0.669	0.437		0.019
AI R - IL R	treatmt	1	8	1.87	0.209		0.059
AI R - IL R	time	1	8	5.58	0.046	*	0.082
AI R - IL R	treatmt:time	1	8	0.773	0.405		0.025
AI R - M1 L	treatmt	1	8	0.534	0.486		0.026
AI R - M1 L	time	1	8	14.883	0.005	*	0.191
AI R - M1 L	treatmt:time	1	8	2.004	0.195		0.008
AI R - M1 R	treatmt	1	8	1.171	0.311		0.044
AI R - M1 R	time	1	8	4.225	0.074		0.08
AI R - M1 R	treatmt:time	1	8	0.128	0.729		0.002
AI R - M2 L	treatmt	1	8	1.236	0.299		0.047
AI R - M2 L	time	1	8	3.82	0.086		0.083
AI R - M2 L	treatmt:time	1	8	3.132	0.115		0.023
AI R - M2 R	treatmt	1	8	1.395	0.271		0.029
AI R - M2 R	time	1	8	4.359	0.07		0.059
AI R - M2 R	treatmt:time	1	8	1.638	0.236		0.037
AI R - NAcC L	treatmt	1	8	0.475	0.51		0.018
AI R - NAcC L	time	1	8	11.001	0.011	*	0.248
AI R - NAcC L	treatmt:time	1	8	0.322	0.586		0.006
AI R - NAcC R	treatmt	1	8	0.177	0.685		0.006
AI R - NAcC R	time	1	8	6.956	0.03	*	0.111
AI R - NAcC R	treatmt:time	1	8	0.637	0.448		0.013
AI R - NAcSh L	treatmt	1	8	4.328	0.071		0.161
AI R - NAcSh L	time	1	8	7.66	0.024	*	0.177
AI R - NAcSh L	treatmt:time	1	8	1.585	0.244		0.019
AI R - NAcSh R	treatmt	1	8	0.979	0.351		0.064
AI R - NAcSh R	time	1	8	12.344	0.008	*	0.166
AI R - NAcSh R	treatmt:time	1	8	0.000154	0.99		2.96E-06
AI R - PrL L	treatmt	1	8	3.777	0.088		0.08
AI R - PrL L	time	1	8	3.32	0.106		0.095
AI R - PrL L	treatmt:time	1	8	0.413	0.539		0.016
AI R - PrL R	treatmt	1	8	1.007	0.345		0.026
AI R - PrL R	time	1	8	2.079	0.187		0.043
AI R - PrL R	treatmt:time	1	8	1.035	0.339		0.038
AI R - S1 L	treatmt	1	8	0.058	0.816		0.003
AI R - S1 L	time	1	8	4.773	0.06		0.058
AI R - S1 L	treatmt:time	1	8	0.005	0.944		7.03E-05
AI R - S1 R	treatmt	1	8	0.137	0.721		0.006
AI R - S1 R	time	1	8	1.504	0.255		0.035
AI R - S1 R	treatmt:time	1	8	0.028	0.871		0.00028
Cg1 L - AI L	treatmt	1	8	0.005	0.946		0.00028
Cg1 L - AI L	time	1	8	0.109	0.75		0.002
Cg1 L - AI L	treatmt:time	1	8	0.006	0.942		8.74E-05
Cg1 L - GIDI L	treatmt	1	8	0.032	0.863		0.002
Cg1 L - GIDI L	time	1	8	0.541	0.483		0.003
Cg1 L - GIDI L	treatmt:time	1	8	0.064	0.807		0.00071
Cg1 L - M1 L	treatmt	1	8	0.332	0.58		0.024
Cg1 L - M1 L	time	1	8	1.891	0.206		0.022
Cg1 L - M1 L	treatmt:time	1	8	0.725	0.419		0.005
Cg1 L - M2 L	treatmt	1	8	3.803	0.087		0.104
Cg1 L - M2 L	time	1	8	1.072	0.331		0.028
Cg1 L - M2 L	treatmt:time	1	8	0.106	0.753		0.001
Cg1 L - S1 L	treatmt	1	8	0.004	0.952		0.00025
Cg1 L - S1 L	time	1	8	0.26	0.624		0.002
Cg1 L - S1 L	treatmt:time	1	8	0.399	0.545		0.006
Cg1 R - AI L	treatmt	1	8	0.996	0.347		0.047

Cg1 R - AI L	time	2	12	2.044	0.172		0.068
Cg1 R - AI L	treatmt:time	2	12	1.046	0.381		0.024
Cg1 R - Cg1 L	treatmt	1	6	0.138	0.723		0.001
Cg1 R - Cg1 L	time	2	12	10.814	0.002	*	0.321
Cg1 R - Cg1 L	treatmt:time	2	12	0.498	0.62		0.023
Cg1 R - CPu L	treatmt	1	6	0.227	0.651		0.003
Cg1 R - CPu L	time	1.09	6.55	0.263	0.645		0.011
Cg1 R - CPu L	treatmt:time	1.06	6.38	0.188	0.693		0.012
Cg1 R - CPu R	treatmt	1	6	0.03	0.869		0.001
Cg1 R - CPu R	time	2	12	1.129	0.355		0.045
Cg1 R - CPu R	treatmt:time	2	12	1.191	0.338		0.05
Cg1 R - GIDI L	treatmt	1	6	0.044	0.842		0.002
Cg1 R - GIDI L	time	2	12	1.275	0.315		0.067
Cg1 R - GIDI L	treatmt:time	2	12	0.901	0.432		0.027
Cg1 R - IL L	treatmt	1	6	3.305	0.119		0.074
Cg1 R - IL L	time	2	12	4.658	0.032	*	0.169
Cg1 R - IL L	treatmt:time	2	12	0.139	0.871		0.005
Cg1 R - IL R	treatmt	1	6	1.255	0.305		0.034
Cg1 R - IL R	time	2	12	0.36	0.705		0.013
Cg1 R - IL R	treatmt:time	2	12	0.52	0.607		0.018
Cg1 R - M1 L	treatmt	1	6	0.034	0.859		0.002
Cg1 R - M1 L	time	2	12	1.376	0.29		0.053
Cg1 R - M1 L	treatmt:time	2	12	0.048	0.953		0.001
Cg1 R - M2 L	treatmt	1	6	0.158	0.705		0.003
Cg1 R - M2 L	time	2	12	0.409	0.673		0.014
Cg1 R - M2 L	treatmt:time	2	12	0.013	0.987		0.00069
Cg1 R - NAcC L	treatmt	1	6	0.713	0.431		0.023
Cg1 R - NAcC L	time	2	12	4.455	0.036	*	0.166
Cg1 R - NAcC L	treatmt:time	2	12	0.118	0.89		0.007
Cg1 R - NAcC R	treatmt	1	6	7.865	0.031	*	0.088
Cg1 R - NAcC R	time	1.1	6.62	0.329	0.606		0.02
Cg1 R - NAcC R	treatmt:time	1.11	6.63	0.744	0.432		0.043
Cg1 R - NAcSh L	treatmt	1	6	0.003	0.961		0.00019
Cg1 R - NAcSh L	time	2	12	8.105	0.006	*	0.261
Cg1 R - NAcSh L	treatmt:time	2	12	0.754	0.492		0.022
Cg1 R - NAcSh R	treatmt	1	6	3.172	0.125		0.101
Cg1 R - NAcSh R	time	1.18	7.07	2.342	0.17		0.116
Cg1 R - NAcSh R	treatmt:time	2	12	0.673	0.529		0.017
Cg1 R - PrL L	treatmt	1	6	4.95E-06	0.998		1.36E-07
Cg1 R - PrL L	time	1.14	6.87	4.091	0.081		0.148
Cg1 R - PrL L	treatmt:time	2	12	0.086	0.918		0.003
Cg1 R - PrL R	treatmt	1	6	4.557	0.077		0.12
Cg1 R - PrL R	time	2	12	0.75	0.493		0.03
Cg1 R - PrL R	treatmt:time	2	12	2.048	0.172		0.053
Cg1 R - S1 L	treatmt	1	6	0.002	0.963		0.00012
Cg1 R - S1 L	time	2	12	1.268	0.316		0.054
Cg1 R - S1 L	treatmt:time	2	12	0.436	0.657		0.011
CPu L - AI L	treatmt	1	6	0.244	0.639		0.01
CPu L - AI L	time	2	12	0.231	0.797		0.005
CPu L - AI L	treatmt:time	2	12	2.222	0.151		0.057
CPu L - Cg1 L	treatmt	1	6	0.776	0.412		0.009
CPu L - Cg1 L	time	2	12	0.447	0.65		0.017
CPu L - Cg1 L	treatmt:time	2	12	0.25	0.783		0.01
CPu L - GIDI L	treatmt	1	6	2.114	0.196		0.01
CPu L - GIDI L	time	2	12	1.244	0.323		0.066
CPu L - GIDI L	treatmt:time	2	12	1.788	0.209		0.081
CPu L - IL L	treatmt	1	6	0.05	0.83		0.002
CPu L - IL L	time	1.04	6.25	0.14	0.731		0.005
CPu L - IL L	treatmt:time	2	12	1.416	0.28		0.062
CPu L - M1 L	treatmt	1	6	0.003	0.957		7.65E-05
CPu L - M1 L	time	1.12	6.72	0.679	0.455		0.037
CPu L - M1 L	treatmt:time	2	12	0.227	0.8		0.014
CPu L - M2 L	treatmt	1	6	0.141	0.72		0.005
CPu L - M2 L	time	2	12	0.682	0.524		0.024
CPu L - M2 L	treatmt:time	2	12	0.244	0.787		0.017
CPu L - PrL L	treatmt	1	6	0.013	0.913		0.00038
CPu L - PrL L	time	2	12	0.148	0.864		0.008
CPu L - PrL L	treatmt:time	2	12	0.538	0.597		0.032
CPu L - S1 L	treatmt	1	6	0.011	0.921		0.00029
CPu L - S1 L	time	1.14	6.87	0.291	0.637		0.013
CPu L - S1 L	treatmt:time	2	12	0.837	0.457		0.06
CPu R - AI L	treatmt	1	6	4.713	0.073		0.153
CPu R - AI L	time	2	12	2.284	0.144		0.047
CPu R - AI L	treatmt:time	2	12	2.002	0.178		0.08
CPu R - Cg1 L	treatmt	1	6	0.293	0.608		0.009
CPu R - Cg1 L	time	2	12	0.56	0.585		0.021
CPu R - Cg1 L	treatmt:time	2	12	0.812	0.467		0.03
CPu R - CPu L	treatmt	1	6	0.12	0.741		0.004
CPu R - CPu L	time	2	12	1.046	0.381		0.02
CPu R - CPu L	treatmt:time	2	12	0.381	0.691		0.011
CPu R - GIDI L	treatmt	1	6	4.554	0.077		0.063
CPu R - GIDI L	time	2	12	0.946	0.416		0.05
CPu R - GIDI L	treatmt:time	2	12	1.473	0.268		0.056
CPu R - IL L	treatmt	1	6	0.08	0.787		0.003
CPu R - IL L	time	1.14	6.83	0.89	0.393		0.023
CPu R - IL L	treatmt:time	1.13	6.78	1.161	0.328		0.043

Cg1 R - AI L	time	1	8	0.12	0.738		0.003
Cg1 R - AI L	treatmt:time	1	8	0.008	0.932		0.00015
Cg1 R - Cg1 L	treatmt	1	8	7.949	0.023	*	0.181
Cg1 R - Cg1 L	time	1	8	2.069	0.188		0.064
Cg1 R - Cg1 L	treatmt:time	1	8	7.441	0.026	*	0.137
Cg1 R - CPu L	treatmt	1	8	0.726	0.419		0.039
Cg1 R - CPu L	time	1	8	0.348	0.571		0.008
Cg1 R - CPu L	treatmt:time	1	8	2.297	0.168		0.03
Cg1 R - CPu R	treatmt	1	8	1.007	0.345		0.047
Cg1 R - CPu R	time	1	8	0.014	0.907		0.00044
Cg1 R - CPu R	treatmt:time	1	8	0.002	0.969		2.33E-05
Cg1 R - GIDI L	treatmt	1	8	0.666	0.438		0.041
Cg1 R - GIDI L	time	1	8	0.063	0.808		0.00029
Cg1 R - GIDI L	treatmt:time	1	8	0.05	0.829		0.00028
Cg1 R - IL L	treatmt	1	8	1.421	0.267		0.052
Cg1 R - IL L	time	1	8	0.301	0.598		0.003
Cg1 R - IL L	treatmt:time	1	8	0.883	0.375		0.012
Cg1 R - IL R	treatmt	1	8	0.58	0.468		0.015
Cg1 R - IL R	time	1	8	6.102	0.039	*	0.06
Cg1 R - IL R	treatmt:time	1	8	1.624	0.238		0.024
Cg1 R - M1 L	treatmt	1	8	1.702	0.228		0.075
Cg1 R - M1 L	time	1	8	0.832	0.388		0.02
Cg1 R - M1 L	treatmt:time	1	8	0.042	0.842		0.00044
Cg1 R - M2 L	treatmt	1	8	12.008	0.009	*	0.182
Cg1 R - M2 L	time	1	8	0.977	0.352		0.045
Cg1 R - M2 L	treatmt:time	1	8	4.258	0.073		0.029
Cg1 R - NAcC L	treatmt	1	8	0.218	0.653		0.007
Cg1 R - NAcC L	time	1	8	2.788	0.134		0.055
Cg1 R - NAcC L	treatmt:time	1	8	1.856	0.21		0.021
Cg1 R - NAcC R	treatmt	1	8	0.093	0.768		0.003
Cg1 R - NAcC R	time	1	8	0.234	0.642		0.006
Cg1 R - NAcC R	treatmt:time	1	8	0.332	0.58		0.005
Cg1 R - NAcSh L	treatmt	1	8	1.3	0.287		0.038
Cg1 R - NAcSh L	time	1	8	4.452	0.068		0.167
Cg1 R - NAcSh L	treatmt:time	1	8	1.983	0.197		0.026
Cg1 R - NAcSh R	treatmt	1	8	0.155	0.704		0.005
Cg1 R - NAcSh R	time	1	8	1.246	0.297		0.035
Cg1 R - NAcSh R	treatmt:time	1	8	1.569	0.246		0.02
Cg1 R - PrL L	treatmt	1	8	0.007	0.934		0.00029
Cg1 R - PrL L	time	1	8	0.615	0.456		0.016
Cg1 R - PrL L	treatmt:time	1	8	0.881	0.375		0.016
Cg1 R - PrL R	treatmt	1	8	0.031	0.864		0.001
Cg1 R - PrL R	time	1	8	2.889	0.128		0.049
Cg1 R - PrL R	treatmt:time	1	8	10.49	0.012	*	0.089
Cg1 R - S1 L	treatmt	1	8	0.382	0.554		0.02
Cg1 R - S1 L	time	1	8	0.29	0.605		0.003
Cg1 R - S1 L	treatmt:time	1	8	0.182	0.681		0.002
CPu L - AI L	treatmt	1	8	1.935	0.202		0.079
CPu L - AI L	time	1	8	4.877	0.058		0.15
CPu L - AI L	treatmt:time	1	8	0.354	0.569		0.004
CPu L - Cg1 L	treatmt	1	8	0.31	0.593		0.02
CPu L - Cg1 L	time	1	8	1.771	0.22		0.027
CPu L - Cg1 L	treatmt:time	1	8	0.841	0.386		0.006
CPu L - GIDI L	treatmt	1	8	1.047	0.336		0.028
CPu L - GIDI L	time	1	8	3.533	0.097		0.134
CPu L - GIDI L	treatmt:time	1	8	0.414	0.538		0.007
CPu L - IL L	treatmt	1	8	5.215	0.052		0.206
CPu L - IL L	time	1	8	0.967	0.354		0.025
CPu L - IL L	treatmt:time	1	8	0.044	0.839		0.00081
CPu L - M1 L	treatmt	1	8	4.579	0.065		0.096
CPu L - M1 L	time	1	8	1.608	0.24		0.033
CPu L - M1 L	treatmt:time	1	8	1.477	0.259		0.027
CPu L - M2 L	treatmt	1	8	0.02	0.89		0.001
CPu L - M2 L	time	1	8	0.247	0.633		0.005
CPu L - M2 L	treatmt:time	1	8	0.000724	0.979		1.75E-05
CPu L - PrL L	treatmt	1	8	1.619	0.239		0.095
CPu L - PrL L	time	1	8	0.143	0.715		0.003
CPu L - PrL L	treatmt:time	1	8	0.185	0.679		0.0009
CPu L - S1 L	treatmt	1	8	6.949	0.03	*	0.07
CPu L - S1 L	time	1	8	0.91	0.368		0.032
CPu L - S1 L	treatmt:time	1	8	0.346	0.573		0.006
CPu R - AI L	treatmt	1	8	6.60E-07	0.999		2.41E-08
CPu R - AI L	time	1	8	3.79	0.087		0.089
CPu R - AI L	treatmt:time	1	8	0.786	0.401		0.019
CPu R - Cg1 L	treatmt	1	8	1.139	0.317		0.078
CPu R - Cg1 L	time	1	8	1.161	0.313		0.015
CPu R - Cg1 L	treatmt:time	1	8	0.275	0.614		0.006
CPu R - CPu L	treatmt	1	8	2.277	0.17		0.046
CPu R - CPu L	time	1	8	0.63	0.45		0.008
CPu R - CPu L	treatmt:time	1	8	0.091	0.771		0.002
CPu R - GIDI L	treatmt	1	8	0.447	0.522		0.026
CPu R - GIDI L	time	1	8	33.378	0.00042	*	0.317
CPu R - GIDI L	treatmt:time	1	8	4.131	0.077		0.024
CPu R - IL L	treatmt	1	8	4.542	0.066		0.161
CPu R - IL L	time	1	8	2.818	0.132		0.064
CPu R - IL L	treatmt:time	1	8	0.019	0.894		0.00049

CPu R - M1 L	treatmt	1	6	4.441	0.08		0.057
CPu R - M1 L	time	2	12	1.233	0.326		0.055
CPu R - M1 L	treatmt:time	2	12	0.398	0.68		0.018
CPu R - M2 L	treatmt	1	6	0.11	0.751		0.005
CPu R - M2 L	time	2	12	0.517	0.609		0.017
CPu R - M2 L	treatmt:time	2	12	0.658	0.536		0.033
CPu R - NAcC L	treatmt	1	6	16.92	0.006	*	0.082
CPu R - NAcC L	time	1.05	6.28	4.068	0.087		0.127
CPu R - NAcC L	treatmt:time	2	12	0.073	0.93		0.004
CPu R - NAcC R	treatmt	1	6	0.23	0.649		0.008
CPu R - NAcC R	time	2	12	10.085	0.003	*	0.192
CPu R - NAcC R	treatmt:time	2	12	0.052	0.95		0.00099
CPu R - NAcSh L	treatmt	1	6	4.165	0.087		0.161
CPu R - NAcSh L	time	1.17	7.02	1.445	0.276		0.065
CPu R - NAcSh L	treatmt:time	2	12	1.574	0.247		0.046
CPu R - NAcSh R	treatmt	1	6	0.026	0.878		0.00096
CPu R - NAcSh R	time	2	12	3.112	0.082		0.07
CPu R - NAcSh R	treatmt:time	2	12	0.095	0.91		0.002
CPu R - PrL L	treatmt	1	6	3.454	0.112		0.017
CPu R - PrL L	time	2	12	0.572	0.579		0.023
CPu R - PrL L	treatmt:time	2	12	0.935	0.42		0.042
CPu R - S1 L	treatmt	1	6	4.678	0.074		0.106
CPu R - S1 L	time	1.18	7.07	0.91	0.39		0.045
CPu R - S1 L	treatmt:time	2	12	2.79	0.101		0.117
GIDI L - AI L	treatmt	1	6	1.745	0.235		0.093
GIDI L - AI L	time	2	12	4.875	0.028	*	0.15
GIDI L - AI L	treatmt:time	2	12	0.427	0.662		0.014
GIDI R - AI L	treatmt	1	6	0.032	0.863		0.00074
GIDI R - AI L	time	2	12	3.852	0.051		0.1
GIDI R - AI L	treatmt:time	2	12	1.645	0.234		0.076
GIDI R - Cg1 L	treatmt	1	6	0.705	0.433		0.006
GIDI R - Cg1 L	time	1.12	6.69	2.957	0.13		0.077
GIDI R - Cg1 L	treatmt:time	2	12	0.719	0.507		0.037
GIDI R - Cg1 R	treatmt	1	6	6.047	0.049	*	0.055
GIDI R - Cg1 R	time	2	12	3.675	0.057		0.113
GIDI R - Cg1 R	treatmt:time	2	12	0.022	0.978		0.002
GIDI R - CPu L	treatmt	1	6	0.019	0.894		0.001
GIDI R - CPu L	time	2	12	2.476	0.126		0.082
GIDI R - CPu L	treatmt:time	2	12	1.379	0.289		0.032
GIDI R - CPu R	treatmt	1	6	2.672	0.153		0.076
GIDI R - CPu R	time	2	12	0.381	0.691		0.006
GIDI R - CPu R	treatmt:time	2	12	0.704	0.514		0.027
GIDI R - GIDI L	treatmt	1	6	2.014	0.206		0.067
GIDI R - GIDI L	time	2	12	1.307	0.307		0.066
GIDI R - GIDI L	treatmt:time	2	12	0.216	0.809		0.009
GIDI R - IL L	treatmt	1	6	0.157	0.706		0.01
GIDI R - IL L	time	2	12	1.044	0.382		0.043
GIDI R - IL L	treatmt:time	2	12	0.251	0.782		0.006
GIDI R - IL R	treatmt	1	6	0.163	0.7		0.004
GIDI R - IL R	time	2	12	0.655	0.537		0.03
GIDI R - IL R	treatmt:time	2	12	0.789	0.476		0.043
GIDI R - M1 L	treatmt	1	6	0.004	0.952		0.00016
GIDI R - M1 L	time	1.04	6.24	2.439	0.168		0.066
GIDI R - M1 L	treatmt:time	2	12	1.183	0.34		0.033
GIDI R - M1 R	treatmt	1	6	0.036	0.855		0.002
GIDI R - M1 R	time	2	12	0.827	0.461		0.032
GIDI R - M1 R	treatmt:time	2	12	0.222	0.804		0.007
GIDI R - M2 L	treatmt	1	6	0.034	0.86		0.00054
GIDI R - M2 L	time	2	12	0.444	0.651		0.017
GIDI R - M2 L	treatmt:time	2	12	4.958	0.027	*	0.096
GIDI R - M2 R	treatmt	1	6	0.62	0.461		0.024
GIDI R - M2 R	time	2	12	0.708	0.512		0.02
GIDI R - M2 R	treatmt:time	2	12	0.372	0.697		0.017
GIDI R - NAcC L	treatmt	1	6	1.347	0.29		0.042
GIDI R - NAcC L	time	2	12	7.302	0.008	*	0.163
GIDI R - NAcC L	treatmt:time	2	12	0.066	0.936		0.003
GIDI R - NAcC R	treatmt	1	6	0.196	0.674		0.012
GIDI R - NAcC R	time	2	12	3.184	0.078		0.071
GIDI R - NAcC R	treatmt:time	2	12	0.444	0.652		0.013
GIDI R - NAcSh L	treatmt	1	6	1.582	0.255		0.062
GIDI R - NAcSh L	time	1.07	6.44	5.39	0.055		0.239
GIDI R - NAcSh L	treatmt:time	2	12	1.597	0.243		0.064
GIDI R - NAcSh R	treatmt	1	6	0.001	0.975		4.96E-05
GIDI R - NAcSh R	time	1.15	6.89	2.833	0.136		0.094
GIDI R - NAcSh R	treatmt:time	2	12	1.191	0.337		0.031
GIDI R - PrL L	treatmt	1	6	0.03	0.868		0.00045
GIDI R - PrL L	time	2	12	1.278	0.314		0.046
GIDI R - PrL L	treatmt:time	2	12	0.246	0.786		0.006
GIDI R - PrL R	treatmt	1	6	0.29	0.61		0.009
GIDI R - PrL R	time	2	12	0.245	0.787		0.005
GIDI R - PrL R	treatmt:time	2	12	0.551	0.59		0.014
GIDI R - S1 L	treatmt	1	6	0.265	0.625		0.009
GIDI R - S1 L	time	2	12	3.05	0.085		0.101
GIDI R - S1 L	treatmt:time	2	12	1.597	0.243		0.04
GIDI R - S1 R	treatmt	1	6	2.586	0.159		0.078
GIDI R - S1 R	time	2	12	3.469	0.065		0.084

CPu R - M1 L	treatmt	1	8	0.239	0.638		0.009
CPu R - M1 L	time	1	8	3.028	0.12		0.05
CPu R - M1 L	treatmt:time	1	8	0.362	0.564		0.002
CPu R - M2 L	treatmt	1	8	0.022	0.885		0.001
CPu R - M2 L	time	1	8	0.048	0.832		0.00063
CPu R - M2 L	treatmt:time	1	8	0.061	0.811		0.002
CPu R - NAcC L	treatmt	1	8	10.902	0.011	*	0.157
CPu R - NAcC L	time	1	8	6.701	0.032	*	0.105
CPu R - NAcC L	treatmt:time	1	8	0.133	0.724		0.004
CPu R - NAcC R	treatmt	1	8	3.351	0.105		0.134
CPu R - NAcC R	time	1	8	2.655	0.142		0.044
CPu R - NAcC R	treatmt:time	1	8	1.646	0.235		0.051
CPu R - NAcSh L	treatmt	1	8	0.056	0.819		0.003
CPu R - NAcSh L	time	1	8	2.635	0.143		0.024
CPu R - NAcSh L	treatmt:time	1	8	6.706	0.032	*	0.025
CPu R - NAcSh R	treatmt	1	8	0.799	0.398		0.041
CPu R - NAcSh R	time	1	8	10.11	0.013	*	0.081
CPu R - NAcSh R	treatmt:time	1	8	0.947	0.359		0.01
CPu R - PrL L	treatmt	1	8	0.109	0.749		0.006
CPu R - PrL L	time	1	8	0.115	0.743		0.003
CPu R - PrL L	treatmt:time	1	8	0.114	0.744		0.001
CPu R - S1 L	treatmt	1	8	0.313	0.591		0.009
CPu R - S1 L	time	1	8	11.577	0.009	*	0.218
CPu R - S1 L	treatmt:time	1	8	0.136	0.722		0.002
GIDI L - AI L	treatmt	1	8	0.856	0.382		0.044
GIDI L - AI L	time	1	8	6.405	0.035	*	0.065
GIDI L - AI L	treatmt:time	1	8	1.653	0.234		0.015
GIDI R - AI L	treatmt	1	8	6.386	0.035	*	0.208
GIDI R - AI L	time	1	8	4.395	0.069		0.081
GIDI R - AI L	treatmt:time	1	8	0.086	0.776		0.00092
GIDI R - Cg1 L	treatmt	1	8	0.586	0.466		0.051
GIDI R - Cg1 L	time	1	8	0.000129	0.991		6.19E-07
GIDI R - Cg1 L	treatmt:time	1	8	9.881	0.014	*	0.061
GIDI R - Cg1 R	treatmt	1	8	0.001	0.973		8.61E-05
GIDI R - Cg1 R	time	1	8	1.101	0.325		0.015
GIDI R - Cg1 R	treatmt:time	1	8	5.507	0.047	*	0.055
GIDI R - CPu L	treatmt	1	8	4.917	0.057		0.089
GIDI R - CPu L	time	1	8	9.908	0.014	*	0.226
GIDI R - CPu L	treatmt:time	1	8	0.152	0.707		0.007
GIDI R - CPu R	treatmt	1	8	1.382	0.274		0.033
GIDI R - CPu R	time	1	8	2.959	0.124		0.054
GIDI R - CPu R	treatmt:time	1	8	0.412	0.539		0.012
GIDI R - GIDI L	treatmt	1	8	0.209	0.659		0.007
GIDI R - GIDI L	time	1	8	3.503	0.098		0.066
GIDI R - GIDI L	treatmt:time	1	8	0.359	0.566		0.003
GIDI R - IL L	treatmt	1	8	8.155	0.021	*	0.299
GIDI R - IL L	time	1	8	2.403	0.16		0.063
GIDI R - IL L	treatmt:time	1	8	0.27	0.617		0.004
GIDI R - IL R	treatmt	1	8	10.915	0.011	*	0.308
GIDI R - IL R	time	1	8	1.249	0.296		0.019
GIDI R - IL R	treatmt:time	1	8	0.342	0.575		0.012
GIDI R - M1 L	treatmt	1	8	13.146	0.007	*	0.18
GIDI R - M1 L	time	1	8	2.084	0.187		0.028
GIDI R - M1 L	treatmt:time	1	8	0.041	0.844		0.001
GIDI R - M1 R	treatmt	1	8	18.366	0.003	*	0.114
GIDI R - M1 R	time	1	8	0.002	0.964		9.41E-05
GIDI R - M1 R	treatmt:time	1	8	0.156	0.704		0.004
GIDI R - M2 L	treatmt	1	8	2.525	0.151		0.131
GIDI R - M2 L	time	1	8	1.007	0.345		0.008
GIDI R - M2 L	treatmt:time	1	8	2.427	0.158		0.031
GIDI R - M2 R	treatmt	1	8	9.339	0.016	*	0.154
GIDI R - M2 R	time	1	8	0.647	0.445		0.008
GIDI R - M2 R	treatmt:time	1	8	2.655	0.142		0.06
GIDI R - NAcC L	treatmt	1	8	3.067	0.118		0.096
GIDI R - NAcC L	time	1	8	7.554	0.025	*	0.177
GIDI R - NAcC L	treatmt:time	1	8	0.717	0.422		0.008
GIDI R - NAcC R	treatmt	1	8	0.312	0.592		0.022
GIDI R - NAcC R	time	1	8	3.433	0.101		0.058
GIDI R - NAcC R	treatmt:time	1	8	0.413	0.539		0.007
GIDI R - NAcSh L	treatmt	1	8	6.241	0.037	*	0.238
GIDI R - NAcSh L	time	1	8	7.252	0.027	*	0.085
GIDI R - NAcSh L	treatmt:time	1	8	0.332	0.58		0.011
GIDI R - NAcSh R	treatmt	1	8	2.654	0.142		0.182
GIDI R - NAcSh R	time	1	8	12.606	0.008	*	0.111
GIDI R - NAcSh R	treatmt:time	1	8	0.015	0.907		0.00034
GIDI R - PrL L	treatmt	1	8	2.387	0.161		0.125
GIDI R - PrL L	time	1	8	0.003	0.958		3.79E-05
GIDI R - PrL L	treatmt:time	1	8	0.024	0.88		0.00046
GIDI R - PrL R	treatmt	1	8	2.452	0.156		0.125
GIDI R - PrL R	time	1	8	0.664	0.439		0.011
GIDI R - PrL R	treatmt:time	1	8	4.71E-05	0.995		1.36E-06
GIDI R - S1 L	treatmt	1	8	1.244	0.297		0.046
GIDI R - S1 L	time	1	8	0.515	0.494		0.008
GIDI R - S1 L	treatmt:time	1	8	0.302	0.598		0.005
GIDI R - S1 R	treatmt	1	8	3.886	0.084		0.115
GIDI R - S1 R	time	1	8	0.907	0.369		0.023

GIDI R - S1 R	treatmt:time	2	12	0.236	0.793		0.011
IL L - AI L	treatmt	1	6	0.004	0.951		9.08E-05
IL L - AI L	time	2	12	0.709	0.512		0.03
IL L - AI L	treatmt:time	2	12	0.61	0.56		0.023
IL L - Cg1 L	treatmt	1	6	4.062	0.09		0.051
IL L - Cg1 L	time	2	12	1.481	0.266		0.071
IL L - Cg1 L	treatmt:time	2	12	0.22	0.806		0.008
IL L - GIDI L	treatmt	1	6	0.137	0.724		0.003
IL L - GIDI L	time	2	12	3.992	0.047	*	0.064
IL L - GIDI L	treatmt:time	2	12	0.186	0.833		0.007
IL L - M1 L	treatmt	1	6	1.43	0.277		0.07
IL L - M1 L	time	2	12	1.556	0.251		0.049
IL L - M1 L	treatmt:time	2	12	1.383	0.288		0.04
IL L - M2 L	treatmt	1	6	3.309	0.119		0.113
IL L - M2 L	time	2	12	2.213	0.152		0.112
IL L - M2 L	treatmt:time	2	12	0.21	0.814		0.009
IL L - PrL L	treatmt	1	6	0.007	0.938		0.00054
IL L - PrL L	time	2	12	1.658	0.231		0.047
IL L - PrL L	treatmt:time	1.11	6.65	0.438	0.55		0.012
IL L - S1 L	treatmt	1	6	3.651	0.105		0.114
IL L - S1 L	time	2	12	2.623	0.113		0.056
IL L - S1 L	treatmt:time	2	12	0.13	0.879		0.004
IL R - AI L	treatmt	1	6	0.769	0.414		0.008
IL R - AI L	time	2	12	1.983	0.18		0.099
IL R - AI L	treatmt:time	2	12	0.216	0.809		0.006
IL R - Cg1 L	treatmt	1	6	1.717	0.238		0.023
IL R - Cg1 L	time	1.15	6.89	1.984	0.205		0.096
IL R - Cg1 L	treatmt:time	2	12	0.129	0.88		0.006
IL R - CPu L	treatmt	1	6	0.000495	0.983		1.25E-05
IL R - CPu L	time	2	12	0.511	0.612		0.018
IL R - CPu L	treatmt:time	2	12	1.087	0.368		0.041
IL R - CPu R	treatmt	1	6	0.866	0.388		0.041
IL R - CPu R	time	2	12	2.726	0.106		0.073
IL R - CPu R	treatmt:time	2	12	1.075	0.372		0.036
IL R - GIDI L	treatmt	1	6	0.003	0.962		3.27E-05
IL R - GIDI L	time	2	12	0.924	0.423		0.041
IL R - GIDI L	treatmt:time	2	12	0.086	0.918		0.003
IL R - IL L	treatmt	1	6	0.543	0.489		0.017
IL R - IL L	time	2	12	1.149	0.35		0.027
IL R - IL L	treatmt:time	2	12	0.089	0.916		0.003
IL R - M1 L	treatmt	1	6	0.051	0.829		0.003
IL R - M1 L	time	1.03	6.18	2.368	0.174		0.075
IL R - M1 L	treatmt:time	2	12	0.635	0.547		0.022
IL R - M2 L	treatmt	1	6	0.345	0.579		0.011
IL R - M2 L	time	2	12	1.526	0.257		0.031
IL R - M2 L	treatmt:time	2	12	0.374	0.696		0.013
IL R - NAcC L	treatmt	1	6	0.012	0.918		0.0005
IL R - NAcC L	time	2	12	0.435	0.657		0.009
IL R - NAcC L	treatmt:time	2	12	1.501	0.262		0.09
IL R - NAcC R	treatmt	1	6	0.514	0.5		0.009
IL R - NAcC R	time	2	12	0.576	0.577		0.029
IL R - NAcC R	treatmt:time	2	12	1.152	0.349		0.085
IL R - NAcSh L	treatmt	1	6	0.204	0.668		0.017
IL R - NAcSh L	time	2	12	1.911	0.19		0.038
IL R - NAcSh L	treatmt:time	2	12	0.595	0.567		0.023
IL R - NAcSh R	treatmt	1	6	7.182	0.037	*	0.084
IL R - NAcSh R	time	2	12	3.335	0.071		0.111
IL R - NAcSh R	treatmt:time	2	12	0.17	0.845		0.015
IL R - PrL L	treatmt	1	6	0.378	0.561		0.025
IL R - PrL L	time	2	12	1.55	0.252		0.049
IL R - PrL L	treatmt:time	1.02	6.1	0.405	0.551		0.011
IL R - S1 L	treatmt	1	6	0.132	0.729		0.005
IL R - S1 L	time	2	12	1.43	0.277		0.047
IL R - S1 L	treatmt:time	2	12	0.296	0.749		0.012
M1 L - AI L	treatmt	1	6	4.888	0.069		0.119
M1 L - AI L	time	2	12	5	0.026	*	0.104
M1 L - AI L	treatmt:time	2	12	0.717	0.508		0.017
M1 L - GIDI L	treatmt	1	6	1.272	0.302		0.032
M1 L - GIDI L	time	2	12	2.834	0.098		0.155
M1 L - GIDI L	treatmt:time	2	12	0.182	0.836		0.005
M1 L - S1 L	treatmt	1	6	0.018	0.897		0.001
M1 L - S1 L	time	2	12	0.49	0.624		0.027
M1 L - S1 L	treatmt:time	2	12	0.106	0.9		0.004
M1 R - AI L	treatmt	1	6	1.383	0.284		0.062
M1 R - AI L	time	2	12	0.61	0.56		0.027
M1 R - AI L	treatmt:time	1.09	6.57	5.295	0.055		0.134
M1 R - Cg1 L	treatmt	1	6	0.636	0.456		0.01
M1 R - Cg1 L	time	2	12	0.809	0.468		0.059
M1 R - Cg1 L	treatmt:time	2	12	0.575	0.577		0.016
M1 R - Cg1 R	treatmt	1	6	0.128	0.732		0.002
M1 R - Cg1 R	time	2	12	0.145	0.866		0.014
M1 R - Cg1 R	treatmt:time	2	12	1.013	0.392		0.024
M1 R - CPu L	treatmt	1	6	0.704	0.434		0.013
M1 R - CPu L	time	2	12	1.029	0.387		0.049
M1 R - CPu L	treatmt:time	2	12	0.475	0.633		0.024
M1 R - CPu R	treatmt	1	6	0.321	0.591		0.01

GIDI R - S1 R	treatmt:time	1	8	0.331	0.581		0.006
IL L - AI L	treatmt	1	8	4.37	0.07		0.104
IL L - AI L	time	1	8	0.65	0.443		0.015
IL L - AI L	treatmt:time	1	8	0.297	0.6		0.004
IL L - Cg1 L	treatmt	1	8	3.531	0.097		0.044
IL L - Cg1 L	time	1	8	0.289	0.605		0.005
IL L - Cg1 L	treatmt:time	1	8	1.224	0.301		0.019
IL L - GIDI L	treatmt	1	8	4.507	0.067		0.193
IL L - GIDI L	time	1	8	0.775	0.404		0.032
IL L - GIDI L	treatmt:time	1	8	0.404	0.543		0.005
IL L - M1 L	treatmt	1	8	1.948	0.2		0.105
IL L - M1 L	time	1	8	0.799	0.398		0.011
IL L - M1 L	treatmt:time	1	8	0.402	0.544		0.01
IL L - M2 L	treatmt	1	8	0.045	0.837		0.001
IL L - M2 L	time	1	8	0.867	0.379		0.017
IL L - M2 L	treatmt:time	1	8	0.131	0.727		0.003
IL L - PrL L	treatmt	1	8	2.37	0.162		0.029
IL L - PrL L	time	1	8	4.309	0.072		0.145
IL L - PrL L	treatmt:time	1	8	0.011	0.92		0.0002
IL L - S1 L	treatmt	1	8	4.884	0.058		0.233
IL L - S1 L	time	1	8	2.273	0.17		0.051
IL L - S1 L	treatmt:time	1	8	0.524	0.49		0.007
IL R - AI L	treatmt	1	8	4.585	0.065		0.115
IL R - AI L	time	1	8	0.981	0.351		0.024
IL R - AI L	treatmt:time	1	8	0.121	0.737		0.004
IL R - Cg1 L	treatmt	1	8	2.689	0.14		0.031
IL R - Cg1 L	time	1	8	0.989	0.349		0.018
IL R - Cg1 L	treatmt:time	1	8	0.365	0.562		0.005
IL R - CPu L	treatmt	1	8	4.252	0.073		0.133
IL R - CPu L	time	1	8	0.036	0.854		0.00094
IL R - CPu L	treatmt:time	1	8	0.975	0.352		0.019
IL R - CPu R	treatmt	1	8	9.57	0.015	*	0.215
IL R - CPu R	time	1	8	0.08	0.784		0.002
IL R - CPu R	treatmt:time	1	8	0.696	0.428		0.012
IL R - GIDI L	treatmt	1	8	8.484	0.02	*	0.158
IL R - GIDI L	time	1	8	2.187	0.177		0.105
IL R - GIDI L	treatmt:time	1	8	13.242	0.007	*	0.147
IL R - IL L	treatmt	1	8	0.742	0.414		0.046
IL R - IL L	time	1	8	53.152	#######	*	0.407
IL R - IL L	treatmt:time	1	8	0.004	0.95		0.00011
IL R - M1 L	treatmt	1	8	2.185	0.178		0.085
IL R - M1 L	time	1	8	0.302	0.597		0.006
IL R - M1 L	treatmt:time	1	8	2.521	0.151		0.052
IL R - M2 L	treatmt	1	8	0.412	0.539		0.012
IL R - M2 L	time	1	8	1.484	0.258		0.044
IL R - M2 L	treatmt:time	1	8	0.426	0.532		0.008
IL R - NAcC L	treatmt	1	8	2.073	0.188		0.086
IL R - NAcC L	time	1	8	2.233	0.173		0.054
IL R - NAcC L	treatmt:time	1	8	2.688	0.14		0.02
IL R - NAcC R	treatmt	1	8	25.416	0.001	*	0.32
IL R - NAcC R	time	1	8	0.007	0.936		9.55E-05
IL R - NAcC R	treatmt:time	1	8	5.968	0.04	*	0.141
IL R - NAcSh L	treatmt	1	8	1.495	0.256		0.038
IL R - NAcSh L	time	1	8	1.274	0.292		0.014
IL R - NAcSh L	treatmt:time	1	8	0.907	0.369		0.016
IL R - NAcSh R	treatmt	1	8	6.997	0.029	*	0.051
IL R - NAcSh R	time	1	8	2.248	0.172		0.019
IL R - NAcSh R	treatmt:time	1	8	3.778	0.088		0.038
IL R - PrL L	treatmt	1	8	0.154	0.705		0.003
IL R - PrL L	time	1	8	3.471	0.099		0.126
IL R - PrL L	treatmt:time	1	8	1.501	0.255		0.023
IL R - S1 L	treatmt	1	8	4.804	0.06		0.158
IL R - S1 L	time	1	8	1.711	0.227		0.044
IL R - S1 L	treatmt:time	1	8	1.869	0.209		0.047
M1 L - AI L	treatmt	1	8	1.38	0.274		0.059
M1 L - AI L	time	1	8	3.966	0.082		0.071
M1 L - AI L	treatmt:time	1	8	0.151	0.708		0.002
M1 L - GIDI L	treatmt	1	8	1.257	0.295		0.056
M1 L - GIDI L	time	1	8	5.283	0.051		0.102
M1 L - GIDI L	treatmt:time	1	8	0.001	0.971		1.37E-05
M1 L - S1 L	treatmt	1	8	3.035	0.12		0.067
M1 L - S1 L	time	1	8	4.04	0.079		0.066
M1 L - S1 L	treatmt:time	1	8	1.337	0.281		0.007
M1 R - AI L	treatmt	1	8	1.703	0.228		0.048
M1 R - AI L	time	1	8	2.679	0.14		0.052
M1 R - AI L	treatmt:time	1	8	1.251	0.296		0.036
M1 R - Cg1 L	treatmt	1	8	0.911	0.368		0.057
M1 R - Cg1 L	time	1	8	0.003	0.957		3.75E-05
M1 R - Cg1 L	treatmt:time	1	8	0.682	0.433		0.013
M1 R - Cg1 R	treatmt	1	8	1.111	0.323		0.064
M1 R - Cg1 R	time	1	8	0.644	0.445		0.011
M1 R - Cg1 R	treatmt:time	1	8	1.793	0.217		0.018
M1 R - CPu L	treatmt	1	8	1.426	0.267		0.011
M1 R - CPu L	time	1	8	3.148	0.114		0.061
M1 R - CPu L	treatmt:time	1	8	1.76	0.221		0.024
M1 R - CPu R	treatmt	1	8	1.273	0.292		0.047

M1 R - CPu R	time	2	12	1.027	0.387		0.07
M1 R - CPu R	treatmt:time	2	12	1.624	0.238		0.074
M1 R - GIDI L	treatmt	1	6	1.862	0.221		0.059
M1 R - GIDI L	time	2	12	1.793	0.208		0.072
M1 R - GIDI L	treatmt:time	2	12	8.644	0.005	*	0.196
M1 R - IL L	treatmt	1	6	2.226	0.186		0.05
M1 R - IL L	time	2	12	0.426	0.663		0.016
M1 R - IL L	treatmt:time	2	12	0.196	0.825		0.01
M1 R - IL R	treatmt	1	6	1.502	0.266		0.021
M1 R - IL R	time	2	12	0.825	0.462		0.03
M1 R - IL R	treatmt:time	2	12	0.395	0.682		0.029
M1 R - M1 L	treatmt	1	6	0.041	0.846		0.002
M1 R - M1 L	time	2	12	0.094	0.911		0.006
M1 R - M1 L	treatmt:time	1.09	6.51	0.326	0.605		0.007
M1 R - M2 L	treatmt	1	6	2.042	0.203		0.028
M1 R - M2 L	time	2	12	0.042	0.959		0.003
M1 R - M2 L	treatmt:time	2	12	0.185	0.834		0.004
M1 R - M2 R	treatmt	1	6	0.002	0.968		3.91E-05
M1 R - M2 R	time	2	12	0.108	0.899		0.007
M1 R - M2 R	treatmt:time	2	12	0.031	0.969		0.00082
M1 R - NAcC L	treatmt	1	6	1.096	0.335		0.047
M1 R - NAcC L	time	2	12	1.199	0.335		0.057
M1 R - NAcC L	treatmt:time	2	12	0.189	0.83		0.003
M1 R - NAcC R	treatmt	1	6	0.271	0.621		0.008
M1 R - NAcC R	time	2	12	0.144	0.867		0.006
M1 R - NAcC R	treatmt:time	2	12	0.257	0.777		0.015
M1 R - NAcSh L	treatmt	1	6	0.685	0.44		0.042
M1 R - NAcSh L	time	2	12	4.481	0.035	*	0.145
M1 R - NAcSh L	treatmt:time	2	12	0.744	0.496		0.023
M1 R - NAcSh R	treatmt	1	6	0.021	0.891		0.001
M1 R - NAcSh R	time	2	12	1.164	0.345		0.043
M1 R - NAcSh R	treatmt:time	2	12	2.22	0.151		0.028
M1 R - PrL L	treatmt	1	6	0.685	0.44		0.009
M1 R - PrL L	time	2	12	0.635	0.547		0.037
M1 R - PrL L	treatmt:time	2	12	0.432	0.659		0.013
M1 R - PrL R	treatmt	1	6	0.701	0.435		0.011
M1 R - PrL R	time	2	12	0.1	0.906		0.005
M1 R - PrL R	treatmt:time	1.14	6.86	0.491	0.531		0.028
M1 R - S1 L	treatmt	1	6	2.692	0.152		0.053
M1 R - S1 L	time	2	12	0.687	0.522		0.029
M1 R - S1 L	treatmt:time	2	12	1.79	0.209		0.066
M2 L - AI L	treatmt	1	6	0.3	0.604		0.001
M2 L - AI L	time	2	12	0.003	0.997		0.00012
M2 L - AI L	treatmt:time	2	12	0.715	0.509		0.03
M2 L - GIDI L	treatmt	1	6	0.706	0.433		0.014
M2 L - GIDI L	time	2	12	0.257	0.778		0.011
M2 L - GIDI L	treatmt:time	2	12	1.312	0.305		0.078
M2 L - M1 L	treatmt	1	6	0.051	0.829		0.003
M2 L - M1 L	time	2	12	1.058	0.377		0.04
M2 L - M1 L	treatmt:time	2	12	0.381	0.691		0.013
M2 L - S1 L	treatmt	1	6	0.549	0.487		0.012
M2 L - S1 L	time	2	12	0.246	0.786		0.014
M2 L - S1 L	treatmt:time	2	12	0.001	0.999		6.58E-05
M2 R - AI L	treatmt	1	6	9.731	0.021	*	0.099
M2 R - AI L	time	2	12	2.001	0.178		0.043
M2 R - AI L	treatmt:time	2	12	1.178	0.341		0.045
M2 R - Cg1 L	treatmt	1	6	0.168	0.696		0.002
M2 R - Cg1 L	time	1.14	6.86	0.602	0.486		0.039
M2 R - Cg1 L	treatmt:time	2	12	0.594	0.567		0.013
M2 R - Cg1 R	treatmt	1	6	1.675	0.243		0.031
M2 R - Cg1 R	time	2	12	0.19	0.83		0.011
M2 R - Cg1 R	treatmt:time	2	12	2.299	0.143		0.042
M2 R - CPu L	treatmt	1	6	0.001	0.972		4.59E-05
M2 R - CPu L	time	1.16	6.94	0.992	0.367		0.057
M2 R - CPu L	treatmt:time	2	12	0.028	0.973		0.00077
M2 R - CPu R	treatmt	1	6	0.175	0.69		0.005
M2 R - CPu R	time	2	12	3.299	0.072		0.24
M2 R - CPu R	treatmt:time	2	12	2.204	0.153		0.055
M2 R - GIDI L	treatmt	1	6	27.378	0.002	*	0.231
M2 R - GIDI L	time	2	12	1.194	0.336		0.064
M2 R - GIDI L	treatmt:time	2	12	1.868	0.197		0.081
M2 R - IL L	treatmt	1	6	0.776	0.412		0.027
M2 R - IL L	time	2	12	0.915	0.427		0.036
M2 R - IL L	treatmt:time	1.18	7.05	0.682	0.46		0.037
M2 R - IL R	treatmt	1	6	1.267	0.303		0.042
M2 R - IL R	time	2	12	1.484	0.266		0.063
M2 R - IL R	treatmt:time	2	12	1.085	0.369		0.063
M2 R - M1 L	treatmt	1	6	4	0.092		0.073
M2 R - M1 L	time	2	12	0.779	0.481		0.046
M2 R - M1 L	treatmt:time	2	12	0.101	0.905		0.004
M2 R - M2 L	treatmt	1	6	0.064	0.809		0.003
M2 R - M2 L	time	2	12	0.046	0.956		0.001
M2 R - M2 L	treatmt:time	2	12	0.04	0.961		0.001
M2 R - NAcC L	treatmt	1	6	0.045	0.839		0.002
M2 R - NAcC L	time	1.15	6.88	1.666	0.243		0.101
M2 R - NAcC L	treatmt:time	2	12	0.68	0.525		0.022

M1 R - CPu R	time	1	8	1.829	0.213		0.038
M1 R - CPu R	treatmt:time	1	8	1.314	0.285		0.013
M1 R - GIDI L	treatmt	1	8	0.258	0.625		0.006
M1 R - GIDI L	time	1	8	14.08	0.006	*	0.177
M1 R - GIDI L	treatmt:time	1	8	0.978	0.352		0.013
M1 R - IL L	treatmt	1	8	3.711	0.09		0.127
M1 R - IL L	time	1	8	1.857	0.21		0.063
M1 R - IL L	treatmt:time	1	8	0.253	0.628		0.007
M1 R - IL R	treatmt	1	8	1.39	0.272		0.052
M1 R - IL R	time	1	8	0.444	0.524		0.016
M1 R - IL R	treatmt:time	1	8	3.203	0.111		0.084
M1 R - M1 L	treatmt	1	8	0.731	0.417		0.022
M1 R - M1 L	time	1	8	7.169	0.028	*	0.079
M1 R - M1 L	treatmt:time	1	8	0.015	0.907		0.00024
M1 R - M2 L	treatmt	1	8	0.02	0.891		0.001
M1 R - M2 L	time	1	8	0.668	0.437		0.012
M1 R - M2 L	treatmt:time	1	8	0.685	0.432		0.01
M1 R - M2 R	treatmt	1	8	0.145	0.713		0.005
M1 R - M2 R	time	1	8	0.788	0.401		0.011
M1 R - M2 R	treatmt:time	1	8	0.263	0.622		0.004
M1 R - NAcC L	treatmt	1	8	1.377	0.274		0.064
M1 R - NAcC L	time	1	8	2.748	0.136		0.063
M1 R - NAcC L	treatmt:time	1	8	2.47	0.155		0.053
M1 R - NAcC R	treatmt	1	8	0.035	0.857		0.002
M1 R - NAcC R	time	1	8	0.654	0.442		0.01
M1 R - NAcC R	treatmt:time	1	8	0.331	0.581		0.009
M1 R - NAcSh L	treatmt	1	8	0.153	0.706		0.01
M1 R - NAcSh L	time	1	8	13.155	0.007	*	0.181
M1 R - NAcSh L	treatmt:time	1	8	3.189	0.112		0.05
M1 R - NAcSh R	treatmt	1	8	0.354	0.568		0.02
M1 R - NAcSh R	time	1	8	16.875	0.003	*	0.222
M1 R - NAcSh R	treatmt:time	1	8	0.678	0.434		0.008
M1 R - PrL L	treatmt	1	8	0.598	0.462		0.027
M1 R - PrL L	time	1	8	0.262	0.623		0.003
M1 R - PrL L	treatmt:time	1	8	0.052	0.826		0.00074
M1 R - PrL R	treatmt	1	8	0.42	0.535		0.018
M1 R - PrL R	time	1	8	1.271	0.292		0.029
M1 R - PrL R	treatmt:time	1	8	0.016	0.902		0.00017
M1 R - S1 L	treatmt	1	8	0.07	0.798		0.002
M1 R - S1 L	time	1	8	6.295	0.036	*	0.114
M1 R - S1 L	treatmt:time	1	8	0.257	0.626		0.006
M2 L - AI L	treatmt	1	8	2.174	0.179		0.092
M2 L - AI L	time	1	8	3.783	0.088		0.045
M2 L - AI L	treatmt:time	1	8	0.205	0.663		0.001
M2 L - GIDI L	treatmt	1	8	0.006	0.941		0.00037
M2 L - GIDI L	time	1	8	2.337	0.165		0.048
M2 L - GIDI L	treatmt:time	1	8	0.198	0.668		0.004
M2 L - M1 L	treatmt	1	8	0.252	0.629		0.02
M2 L - M1 L	time	1	8	0.012	0.915		0.00021
M2 L - M1 L	treatmt:time	1	8	0.117	0.742		0.00084
M2 L - S1 L	treatmt	1	8	0.198	0.668		0.014
M2 L - S1 L	time	1	8	1.069	0.331		0.022
M2 L - S1 L	treatmt:time	1	8	1.584	0.244		0.018
M2 R - AI L	treatmt	1	8	2.842	0.13		0.039
M2 R - AI L	time	1	8	1.878	0.208		0.04
M2 R - AI L	treatmt:time	1	8	1.451	0.263		0.041
M2 R - Cg1 L	treatmt	1	8	2.232	0.174		0.11
M2 R - Cg1 L	time	1	8	0.000415	0.984		7.36E-06
M2 R - Cg1 L	treatmt:time	1	8	0.709	0.424		0.013
M2 R - Cg1 R	treatmt	1	8	0.512	0.495		0.026
M2 R - Cg1 R	time	1	8	0.009	0.928		0.00026
M2 R - Cg1 R	treatmt:time	1	8	0.073	0.794		0.00038
M2 R - CPu L	treatmt	1	8	0.12	0.738		0.002
M2 R - CPu L	time	1	8	0.092	0.77		0.004
M2 R - CPu L	treatmt:time	1	8	0.105	0.754		0.002
M2 R - CPu R	treatmt	1	8	7.39	0.026	*	0.167
M2 R - CPu R	time	1	8	0.009	0.928		0.00021
M2 R - CPu R	treatmt:time	1	8	1.121	0.321		0.027
M2 R - GIDI L	treatmt	1	8	0.064	0.806		0.003
M2 R - GIDI L	time	1	8	9.751	0.014	*	0.108
M2 R - GIDI L	treatmt:time	1	8	0.005	0.944		9.01E-05
M2 R - IL L	treatmt	1	8	5.36	0.049	*	0.156
M2 R - IL L	time	1	8	0.978	0.352		0.028
M2 R - IL L	treatmt:time	1	8	1.535	0.25		0.039
M2 R - IL R	treatmt	1	8	4.907	0.058		0.119
M2 R - IL R	time	1	8	0.359	0.566		0.011
M2 R - IL R	treatmt:time	1	8	2.498	0.153		0.048
M2 R - M1 L	treatmt	1	8	0.825	0.39		0.035
M2 R - M1 L	time	1	8	0.029	0.869		0.00091
M2 R - M1 L	treatmt:time	1	8	2.799	0.133		0.043
M2 R - M2 L	treatmt	1	8	0.124	0.734		0.005
M2 R - M2 L	time	1	8	0.192	0.673		0.004
M2 R - M2 L	treatmt:time	1	8	1.815	0.215		0.02
M2 R - NAcC L	treatmt	1	8	1.568	0.246		0.059
M2 R - NAcC L	time	1	8	0.313	0.591		0.015
M2 R - NAcC L	treatmt:time	1	8	2.792	0.133		0.033

M2 R - NAcC R	treatmt	1	6	1.087	0.337		0.04
M2 R - NAcC R	time	2	12	1.93	0.188		0.096
M2 R - NAcC R	treatmt:time	2	12	0.036	0.965		0.001
M2 R - NAcSh L	treatmt	1	6	0.505	0.504		0.034
M2 R - NAcSh L	time	2	12	2.15	0.159		0.071
M2 R - NAcSh L	treatmt:time	1.11	6.64	0.336	0.603		0.008
M2 R - NAcSh R	treatmt	1	6	1.318	0.295		0.03
M2 R - NAcSh R	time	2	12	2.707	0.107		0.087
M2 R - NAcSh R	treatmt:time	2	12	0.213	0.812		0.003
M2 R - PrL L	treatmt	1	6	1.114	0.332		0.005
M2 R - PrL L	time	2	12	0.927	0.422		0.061
M2 R - PrL L	treatmt:time	2	12	1.182	0.34		0.062
M2 R - PrL R	treatmt	1	6	0.656	0.449		0.019
M2 R - PrL R	time	2	12	0.168	0.847		0.009
M2 R - PrL R	treatmt:time	2	12	0.896	0.434		0.054
M2 R - S1 L	treatmt	1	6	15.114	0.008	*	0.071
M2 R - S1 L	time	1.1	6.63	0.227	0.673		0.015
M2 R - S1 L	treatmt:time	2	12	1.434	0.276		0.047
NAcC L - AI L	treatmt	1	6	5.281	0.061		0.188
NAcC L - AI L	time	2	12	3.808	0.052		0.065
NAcC L - AI L	treatmt:time	2	12	2.262	0.147		0.07
NAcC L - Cg1 L	treatmt	1	6	0.088	0.777		0.003
NAcC L - Cg1 L	time	2	12	10.423	0.002	*	0.206
NAcC L - Cg1 L	treatmt:time	2	12	1.048	0.381		0.043
NAcC L - CPu L	treatmt	1	6	0.000149	0.991		2.99E-06
NAcC L - CPu L	time	1.09	6.56	3.749	0.095		0.194
NAcC L - CPu L	treatmt:time	2	12	0.502	0.617		0.023
NAcC L - GIDI L	treatmt	1	6	7.012	0.038	*	0.098
NAcC L - GIDI L	time	2	12	2.808	0.1		0.094
NAcC L - GIDI L	treatmt:time	2	12	0.07	0.932		0.003
NAcC L - IL L	treatmt	1	6	0.979	0.361		0.057
NAcC L - IL L	time	2	12	0.056	0.945		0.00078
NAcC L - IL L	treatmt:time	2	12	2.521	0.122		0.076
NAcC L - M1 L	treatmt	1	6	0.809	0.403		0.036
NAcC L - M1 L	time	2	12	4.941	0.027	*	0.123
NAcC L - M1 L	treatmt:time	2	12	1.064	0.376		0.051
NAcC L - M2 L	treatmt	1	6	0.153	0.709		0.006
NAcC L - M2 L	time	2	12	5.251	0.023	*	0.156
NAcC L - M2 L	treatmt:time	2	12	0.297	0.749		0.017
NAcC L - PrL L	treatmt	1	6	1.64	0.248		0.058
NAcC L - PrL L	time	2	12	1.539	0.254		0.047
NAcC L - PrL L	treatmt:time	2	12	3.676	0.057		0.072
NAcC L - S1 L	treatmt	1	6	1.301	0.298		0.046
NAcC L - S1 L	time	1.18	7.05	3.483	0.101		0.065
NAcC L - S1 L	treatmt:time	2	12	0.755	0.491		0.047
NAcC R - AI L	treatmt	1	6	1.282	0.301		0.067
NAcC R - AI L	time	1.16	6.98	3.611	0.096		0.126
NAcC R - AI L	treatmt:time	1.14	6.85	0.66	0.464		0.031
NAcC R - Cg1 L	treatmt	1	6	2.098	0.198		0.034
NAcC R - Cg1 L	time	2	12	0.425	0.663		0.021
NAcC R - Cg1 L	treatmt:time	2	12	0.211	0.813		0.012
NAcC R - CPu L	treatmt	1	6	2.097	0.198		0.058
NAcC R - CPu L	time	2	12	2.282	0.145		0.056
NAcC R - CPu L	treatmt:time	2	12	0.184	0.835		0.004
NAcC R - GIDI L	treatmt	1	6	0.557	0.484		0.015
NAcC R - GIDI L	time	2	12	1.981	0.18		0.087
NAcC R - GIDI L	treatmt:time	2	12	1.63	0.236		0.084
NAcC R - IL L	treatmt	1	6	8.054	0.03	*	0.09
NAcC R - IL L	time	2	12	0.213	0.811		0.011
NAcC R - IL L	treatmt:time	2	12	1.637	0.235		0.098
NAcC R - M1 L	treatmt	1	6	0.061	0.813		0.003
NAcC R - M1 L	time	1.08	6.47	1.144	0.329		0.041
NAcC R - M1 L	treatmt:time	2	12	0.287	0.755		0.014
NAcC R - M2 L	treatmt	1	6	0.933	0.371		0.031
NAcC R - M2 L	time	2	12	0.016	0.985		0.0005
NAcC R - M2 L	treatmt:time	2	12	0.578	0.576		0.028
NAcC R - NAcC L	treatmt	1	6	0.233	0.646		0.005
NAcC R - NAcC L	time	2	12	1.061	0.376		0.071
NAcC R - NAcC L	treatmt:time	2	12	0.313	0.737		0.011
NAcC R - NAcSh	treatmt	1	6	0.571	0.478		0.027
NAcC R - NAcSh	time	2	12	0.831	0.459		0.044
NAcC R - NAcSh	treatmt:time	2	12	1.911	0.19		0.05
NAcC R - NAcSh	treatmt	1	6	1.018	0.352		0.048
NAcC R - NAcSh	time	1.11	6.68	2.121	0.192		0.11
NAcC R - NAcSh	treatmt:time	2	12	1.1	0.364		0.038
NAcC R - PrL L	treatmt	1	6	1.813	0.227		0.018
NAcC R - PrL L	time	2	12	0.128	0.881		0.01
NAcC R - PrL L	treatmt:time	2	12	1.714	0.221		0.084
NAcC R - S1 L	treatmt	1	6	0.00037	0.985		9.79E-06
NAcC R - S1 L	time	2	12	1.906	0.191		0.085
NAcC R - S1 L	treatmt:time	2	12	2.395	0.133		0.128
NAcSh L - AI L	treatmt	1	6	2.159	0.192		0.156
NAcSh L - AI L	time	2	12	2.219	0.151		0.052
NAcSh L - AI L	treatmt:time	2	12	2.138	0.161		0.036
NAcSh L - Cg1 L	treatmt	1	6	0.097	0.766		0.003
NAcSh L - Cg1 L	time	2	12	3.984	0.047	*	0.206

M2 R - NAcC R	treatmt	1	8	0.127	0.731		0.007
M2 R - NAcC R	time	1	8	4.48	0.067		0.045
M2 R - NAcC R	treatmt:time	1	8	1.424	0.267		0.045
M2 R - NAcSh L	treatmt	1	8	0.191	0.674		0.009
M2 R - NAcSh L	time	1	8	0.614	0.456		0.018
M2 R - NAcSh L	treatmt:time	1	8	0.382	0.554		0.006
M2 R - NAcSh R	treatmt	1	8	0.154	0.705		0.008
M2 R - NAcSh R	time	1	8	1.872	0.208		0.046
M2 R - NAcSh R	treatmt:time	1	8	0.103	0.757		0.001
M2 R - PrL L	treatmt	1	8	0.003	0.955		0.00012
M2 R - PrL L	time	1	8	0.421	0.535		0.008
M2 R - PrL L	treatmt:time	1	8	0.008	0.932		0.00026
M2 R - PrL R	treatmt	1	8	0.361	0.565		0.008
M2 R - PrL R	time	1	8	0.018	0.898		0.00023
M2 R - PrL R	treatmt:time	1	8	0.554	0.478		0.004
M2 R - S1 L	treatmt	1	8	0.684	0.432		0.034
M2 R - S1 L	time	1	8	2.64	0.143		0.082
M2 R - S1 L	treatmt:time	1	8	0.179	0.683		0.003
NAcC L - AI L	treatmt	1	8	0.579	0.469		0.021
NAcC L - AI L	time	1	8	10.686	0.011	*	0.243
NAcC L - AI L	treatmt:time	1	8	0.052	0.825		0.001
NAcC L - Cg1 L	treatmt	1	8	0.001	0.975		4.58E-05
NAcC L - Cg1 L	time	1	8	2.572	0.147		0.039
NAcC L - Cg1 L	treatmt:time	1	8	0.949	0.358		0.011
NAcC L - CPu L	treatmt	1	8	8.43E-05	0.993		2.73E-06
NAcC L - CPu L	time	1	8	1.05	0.335		0.023
NAcC L - CPu L	treatmt:time	1	8	1.377	0.274		0.021
NAcC L - GIDI L	treatmt	1	8	0.033	0.859		0.001
NAcC L - GIDI L	time	1	8	1.606	0.241		0.038
NAcC L - GIDI L	treatmt:time	1	8	1.432	0.266		0.024
NAcC L - IL L	treatmt	1	8	4.627	0.064		0.13
NAcC L - IL L	time	1	8	0.107	0.752		0.003
NAcC L - IL L	treatmt:time	1	8	0.834	0.388		0.005
NAcC L - M1 L	treatmt	1	8	0.025	0.879		0.002
NAcC L - M1 L	time	1	8	4.212	0.074		0.071
NAcC L - M1 L	treatmt:time	1	8	3.343	0.105		0.039
NAcC L - M2 L	treatmt	1	8	0.161	0.699		0.007
NAcC L - M2 L	time	1	8	0.777	0.404		0.009
NAcC L - M2 L	treatmt:time	1	8	1.497	0.256		0.032
NAcC L - PrL L	treatmt	1	8	1.517	0.253		0.06
NAcC L - PrL L	time	1	8	0.016	0.902		0.00075
NAcC L - PrL L	treatmt:time	1	8	0.789	0.4		0.008
NAcC L - S1 L	treatmt	1	8	0.019	0.893		0.00081
NAcC L - S1 L	time	1	8	0.462	0.516		0.019
NAcC L - S1 L	treatmt:time	1	8	0.496	0.501		0.007
NAcC R - AI L	treatmt	1	8	0.059	0.814		0.003
NAcC R - AI L	time	1	8	4.379	0.07		0.049
NAcC R - AI L	treatmt:time	1	8	0.068	0.801		0.002
NAcC R - Cg1 L	treatmt	1	8	0.783	0.402		0.027
NAcC R - Cg1 L	time	1	8	5.236	0.051		0.087
NAcC R - Cg1 L	treatmt:time	1	8	0.054	0.822		0.00051
NAcC R - CPu L	treatmt	1	8	0.325	0.584		0.019
NAcC R - CPu L	time	1	8	0.000604	0.981		1.68E-05
NAcC R - CPu L	treatmt:time	1	8	0.113	0.745		0.003
NAcC R - GIDI L	treatmt	1	8	0.298	0.6		0.015
NAcC R - GIDI L	time	1	8	4.523	0.066		0.051
NAcC R - GIDI L	treatmt:time	1	8	0.167	0.693		0.002
NAcC R - IL L	treatmt	1	8	9.527	0.015	*	0.183
NAcC R - IL L	time	1	8	0.892	0.373		0.018
NAcC R - IL L	treatmt:time	1	8	1.895	0.206		0.024
NAcC R - M1 L	treatmt	1	8	0.278	0.612		0.012
NAcC R - M1 L	time	1	8	0.232	0.643		0.007
NAcC R - M1 L	treatmt:time	1	8	0.006	0.939		9.05E-05
NAcC R - M2 L	treatmt	1	8	0.025	0.877		0.00074
NAcC R - M2 L	time	1	8	0.512	0.495		0.017
NAcC R - M2 L	treatmt:time	1	8	0.259	0.624		0.003
NAcC R - NAcC L	treatmt	1	8	0.323	0.586		0.008
NAcC R - NAcC L	time	1	8	0.335	0.579		0.009
NAcC R - NAcC L	treatmt:time	1	8	0.368	0.561		0.013
NAcC R - NAcSh	treatmt	1	8	9.93E-05	0.992		3.42E-06
NAcC R - NAcSh	time	1	8	0.17	0.691		0.007
NAcC R - NAcSh	treatmt:time	1	8	0.175	0.687		0.002
NAcC R - NAcSh	treatmt	1	8	0.464	0.515		0.008
NAcC R - NAcSh	time	1	8	0.03	0.867		0.00061
NAcC R - NAcSh	treatmt:time	1	8	0.071	0.796		0.00089
NAcC R - PrL L	treatmt	1	8	0.399	0.545		0.012
NAcC R - PrL L	time	1	8	0.185	0.678		0.008
NAcC R - PrL L	treatmt:time	1	8	0.305	0.596		0.006
NAcC R - S1 L	treatmt	1	8	0.464	0.515		0.027
NAcC R - S1 L	time	1	8	3.854	0.085		0.051
NAcC R - S1 L	treatmt:time	1	8	0.241	0.637		0.005
NAcSh L - AI L	treatmt	1	8	0.768	0.407		0.032
NAcSh L - AI L	time	1	8	11.31	0.01	*	0.204
NAcSh L - AI L	treatmt:time	1	8	3.039	0.119		0.064
NAcSh L - Cg1 L	treatmt	1	8	0.233	0.642		0.015
NAcSh L - Cg1 L	time	1	8	2.396	0.16		0.074

NAcSh L - Cg1 L	treatmt:time	2	12	0.634	0.547		0.028
NAcSh L - CPu L	treatmt	1	6	0.44	0.532		0.038
NAcSh L - CPu L	time	2	12	7.608	0.007	*	0.195
NAcSh L - CPu L	treatmt:time	1.18	7.05	0.227	0.686		0.008
NAcSh L - GIDI L	treatmt	1	6	2.758	0.148		0.09
NAcSh L - GIDI L	time	2	12	2.099	0.165		0.12
NAcSh L - GIDI L	treatmt:time	2	12	0.006	0.994		0.00016
NAcSh L - IL L	treatmt	1	6	1.283	0.301		0.076
NAcSh L - IL L	time	2	12	0.825	0.462		0.029
NAcSh L - IL L	treatmt:time	2	12	1.382	0.288		0.039
NAcSh L - M1 L	treatmt	1	6	0.183	0.684		0.006
NAcSh L - M1 L	time	2	12	3.469	0.065		0.168
NAcSh L - M1 L	treatmt:time	2	12	0.294	0.75		0.014
NAcSh L - M2 L	treatmt	1	6	0.000777	0.979		7.06E-05
NAcSh L - M2 L	time	1.17	7.01	14.106	0.006	*	0.295
NAcSh L - M2 L	treatmt:time	2	12	3.488	0.064		0.109
NAcSh L - NAcC l	treatmt	1	6	0.089	0.775		0.004
NAcSh L - NAcC l	time	2	12	1	0.396		0.056
NAcSh L - NAcC l	treatmt:time	2	12	0.461	0.641		0.016
NAcSh L - PrL L	treatmt	1	6	0.029	0.871		0.00075
NAcSh L - PrL L	time	2	12	2.302	0.143		0.113
NAcSh L - PrL L	treatmt:time	2	12	0.793	0.475		0.031
NAcSh L - S1 L	treatmt	1	6	1.823	0.226		0.103
NAcSh L - S1 L	time	2	12	4.605	0.033	*	0.213
NAcSh L - S1 L	treatmt:time	2	12	0.273	0.766		0.01
NAcSh R - AI L	treatmt	1	6	1.722	0.237		0.076
NAcSh R - AI L	time	1.13	6.8	0.256	0.658		0.007
NAcSh R - AI L	treatmt:time	2	12	2.202	0.153		0.023
NAcSh R - Cg1 L	treatmt	1	6	1.512	0.265		0.044
NAcSh R - Cg1 L	time	2	12	1.297	0.309		0.07
NAcSh R - Cg1 L	treatmt:time	2	12	0.077	0.926		0.002
NAcSh R - CPu L	treatmt	1	6	0.588	0.472		0.022
NAcSh R - CPu L	time	2	12	2.238	0.149		0.072
NAcSh R - CPu L	treatmt:time	1.08	6.49	0.043	0.859		0.00095
NAcSh R - GIDI L	treatmt	1	6	0.094	0.77		0.003
NAcSh R - GIDI L	time	2	12	1.361	0.293		0.06
NAcSh R - GIDI L	treatmt:time	2	12	2.017	0.176		0.043
NAcSh R - IL L	treatmt	1	6	7.269	0.036	*	0.116
NAcSh R - IL L	time	1.11	6.66	0.075	0.818		0.004
NAcSh R - IL L	treatmt:time	2	12	0.254	0.78		0.012
NAcSh R - M1 L	treatmt	1	6	0.000494	0.983		1.40E-05
NAcSh R - M1 L	time	1.12	6.69	0.7	0.448		0.034
NAcSh R - M1 L	treatmt:time	1.14	6.86	0.238	0.672		0.006
NAcSh R - M2 L	treatmt	1	6	3.991	0.093		0.079
NAcSh R - M2 L	time	2	12	1.078	0.371		0.038
NAcSh R - M2 L	treatmt:time	2	12	0.623	0.553		0.012
NAcSh R - NAcC	treatmt	1	6	0.503	0.505		0.01
NAcSh R - NAcC	time	2	12	1.896	0.192		0.101
NAcSh R - NAcC	treatmt:time	2	12	1.912	0.19		0.065
NAcSh R - NAcSh	treatmt	1	6	0.928	0.373		0.037
NAcSh R - NAcSh	time	2	12	0.43	0.66		0.011
NAcSh R - NAcSh	treatmt:time	2	12	1.454	0.272		0.038
NAcSh R - PrL L	treatmt	1	6	0.000255	0.988		1.72E-06
NAcSh R - PrL L	time	2	12	0.004	0.996		0.00029
NAcSh R - PrL L	treatmt:time	2	12	0.754	0.491		0.024
NAcSh R - S1 L	treatmt	1	6	0.004	0.949		8.54E-05
NAcSh R - S1 L	time	2	12	1.012	0.392		0.054
NAcSh R - S1 L	treatmt:time	2	12	2.419	0.131		0.059
PrL L - AI L	treatmt	1	6	1.588	0.254		0.015
PrL L - AI L	time	2	12	1.034	0.385		0.037
PrL L - AI L	treatmt:time	2	12	0.16	0.854		0.01
PrL L - Cg1 L	treatmt	1	6	0.134	0.727		0.004
PrL L - Cg1 L	time	2	12	0.193	0.827		0.008
PrL L - Cg1 L	treatmt:time	2	12	1.205	0.333		0.035
PrL L - GIDI L	treatmt	1	6	0.619	0.461		0.012
PrL L - GIDI L	time	2	12	3.169	0.079		0.089
PrL L - GIDI L	treatmt:time	2	12	0.468	0.637		0.022
PrL L - M1 L	treatmt	1	6	3.378	0.116		0.053
PrL L - M1 L	time	2	12	3.939	0.048	*	0.128
PrL L - M1 L	treatmt:time	2	12	0.78	0.48		0.044
PrL L - M2 L	treatmt	1	6	0.685	0.44		0.022
PrL L - M2 L	time	2	12	0.967	0.408		0.066
PrL L - M2 L	treatmt:time	2	12	0.433	0.659		0.011
PrL L - S1 L	treatmt	1	6	4.388	0.081		0.07
PrL L - S1 L	time	2	12	3.725	0.055		0.138
PrL L - S1 L	treatmt:time	2	12	0.297	0.749		0.016
PrL R - AI L	treatmt	1	6	0.372	0.564		0.003
PrL R - AI L	time	2	12	1.714	0.221		0.04
PrL R - AI L	treatmt:time	1.08	6.51	0.002	0.976		4.57E-05
PrL R - Cg1 L	treatmt	1	6	0.367	0.567		0.006
PrL R - Cg1 L	time	1.15	6.88	0.71	0.447		0.055
PrL R - Cg1 L	treatmt:time	2	12	0.529	0.602		0.018
PrL R - CPu L	treatmt	1	6	0.107	0.755		0.002
PrL R - CPu L	time	2	12	0.192	0.828		0.009
PrL R - CPu L	treatmt:time	2	12	0.294	0.75		0.02
PrL R - CPu R	treatmt	1	6	1.104	0.334		0.008

NAcSh L - Cg1 L	treatmt:time	1	8	0.12	0.738		0.001
NAcSh L - CPu L	treatmt	1	8	2.114	0.184		0.1
NAcSh L - CPu L	time	1	8	0.563	0.474		0.013
NAcSh L - CPu L	treatmt:time	1	8	1.515	0.253		0.016
NAcSh L - GIDI L	treatmt	1	8	0.026	0.875		0.001
NAcSh L - GIDI L	time	1	8	1.585	0.244		0.05
NAcSh L - GIDI L	treatmt:time	1	8	0.041	0.844		0.001
NAcSh L - IL L	treatmt	1	8	1.375	0.275		0.017
NAcSh L - IL L	time	1	8	0.075	0.791		0.001
NAcSh L - IL L	treatmt:time	1	8	0.014	0.908		0.00025
NAcSh L - M1 L	treatmt	1	8	0.294	0.603		0.019
NAcSh L - M1 L	time	1	8	6.82	0.031	*	0.136
NAcSh L - M1 L	treatmt:time	1	8	4.435	0.068		0.063
NAcSh L - M2 L	treatmt	1	8	0.467	0.514		0.028
NAcSh L - M2 L	time	1	8	0.288	0.606		0.006
NAcSh L - M2 L	treatmt:time	1	8	3.29	0.107		0.058
NAcSh L - NAcC l	treatmt	1	8	2.059	0.189		0.108
NAcSh L - NAcC l	time	1	8	0.356	0.567		0.01
NAcSh L - NAcC l	treatmt:time	1	8	2.925	0.126		0.04
NAcSh L - PrL L	treatmt	1	8	0.946	0.359		0.03
NAcSh L - PrL L	time	1	8	0.179	0.683		0.008
NAcSh L - PrL L	treatmt:time	1	8	0.225	0.648		0.003
NAcSh L - S1 L	treatmt	1	8	0.27	0.617		0.011
NAcSh L - S1 L	time	1	8	3.066	0.118		0.117
NAcSh L - S1 L	treatmt:time	1	8	0.089	0.773		0.002
NAcSh R - AI L	treatmt	1	8	0.015	0.906		0.00066
NAcSh R - AI L	time	1	8	7.805	0.023	*	0.12
NAcSh R - AI L	treatmt:time	1	8	0.853	0.383		0.021
NAcSh R - Cg1 L	treatmt	1	8	0.013	0.913		0.00068
NAcSh R - Cg1 L	time	1	8	2.944	0.125		0.048
NAcSh R - Cg1 L	treatmt:time	1	8	0.312	0.592		0.002
NAcSh R - CPu L	treatmt	1	8	0.077	0.788		0.005
NAcSh R - CPu L	time	1	8	5.19	0.052		0.068
NAcSh R - CPu L	treatmt:time	1	8	3.984	0.081		0.013
NAcSh R - GIDI L	treatmt	1	8	0.61	0.457		0.032
NAcSh R - GIDI L	time	1	8	0.821	0.391		0.032
NAcSh R - GIDI L	treatmt:time	1	8	0.031	0.866		6.00E-04
NAcSh R - IL L	treatmt	1	8	2.366	0.163		0.024
NAcSh R - IL L	time	1	8	0.003	0.957		6.04E-05
NAcSh R - IL L	treatmt:time	1	8	0.381	0.554		0.005
NAcSh R - M1 L	treatmt	1	8	0.242	0.636		0.013
NAcSh R - M1 L	time	1	8	12.576	0.008	*	0.077
NAcSh R - M1 L	treatmt:time	1	8	1.851	0.211		0.014
NAcSh R - M2 L	treatmt	1	8	0.04	0.846		0.002
NAcSh R - M2 L	time	1	8	0.268	0.618		0.003
NAcSh R - M2 L	treatmt:time	1	8	1.876	0.208		0.003
NAcSh R - NAcC	treatmt	1	8	0.034	0.859		0.001
NAcSh R - NAcC	time	1	8	1.752	0.222		0.027
NAcSh R - NAcC	treatmt:time	1	8	0.285	0.608		0.003
NAcSh R - NAcSh	treatmt	1	8	0.031	0.864		0.002
NAcSh R - NAcSh	time	1	8	4.52E-05	0.995		7.08E-07
NAcSh R - NAcSh	treatmt:time	1	8	5.02	0.055		0.024
NAcSh R - PrL L	treatmt	1	8	0.26	0.624		0.005
NAcSh R - PrL L	time	1	8	0.113	0.746		0.005
NAcSh R - PrL L	treatmt:time	1	8	0.036	0.854		0.00037
NAcSh R - S1 L	treatmt	1	8	0.097	0.764		0.006
NAcSh R - S1 L	time	1	8	2.819	0.132		0.088
NAcSh R - S1 L	treatmt:time	1	8	0.493	0.502		0.006
PrL L - AI L	treatmt	1	8	1.753	0.222		0.085
PrL L - AI L	time	1	8	0.207	0.661		0.003
PrL L - AI L	treatmt:time	1	8	5.732	0.044	*	0.054
PrL L - Cg1 L	treatmt	1	8	1.213	0.303		0.028
PrL L - Cg1 L	time	1	8	1.833	0.213		0.058
PrL L - Cg1 L	treatmt:time	1	8	10.11	0.013	*	0.096
PrL L - GIDI L	treatmt	1	8	0.359	0.565		0.03
PrL L - GIDI L	time	1	8	31.15	0.00052	*	0.054
PrL L - GIDI L	treatmt:time	1	8	0.175	0.687		0.002
PrL L - M1 L	treatmt	1	8	0.461	0.516		0.025
PrL L - M1 L	time	1	8	0.048	0.832		0.001
PrL L - M1 L	treatmt:time	1	8	0.615	0.455		0.003
PrL L - M2 L	treatmt	1	8	0.419	0.536		0.016
PrL L - M2 L	time	1	8	1.926	0.203		0.075
PrL L - M2 L	treatmt:time	1	8	0.258	0.625		0.004
PrL L - S1 L	treatmt	1	8	0.468	0.513		0.036
PrL L - S1 L	time	1	8	1.186	0.308		0.009
PrL L - S1 L	treatmt:time	1	8	5.961	0.04	*	0.023
PrL R - AI L	treatmt	1	8	0.426	0.532		0.02
PrL R - AI L	time	1	8	0.214	0.656		0.004
PrL R - AI L	treatmt:time	1	8	2.481	0.154		0.062
PrL R - Cg1 L	treatmt	1	8	0.204	0.663		0.01
PrL R - Cg1 L	time	1	8	0.052	0.825		0.002
PrL R - Cg1 L	treatmt:time	1	8	0.385	0.552		0.003
PrL R - CPu L	treatmt	1	8	3.662	0.092		0.148
PrL R - CPu L	time	1	8	1.563	0.247		0.039
PrL R - CPu L	treatmt:time	1	8	0.845	0.385		0.016
PrL R - CPu R	treatmt	1	8	1.132	0.318		0.041

PrL R - CPu R	time	2	12	1.908	0.191		0.062
PrL R - CPu R	treatmt:time	2	12	0.995	0.398		0.039
PrL R - GIDI L	treatmt	1	6	2.48	0.166		0.046
PrL R - GIDI L	time	2	12	0.362	0.704		0.011
PrL R - GIDI L	treatmt:time	2	12	0.304	0.744		0.014
PrL R - IL L	treatmt	1	6	0.104	0.758		0.006
PrL R - IL L	time	2	12	3.137	0.08		0.083
PrL R - IL L	treatmt:time	2	12	0.724	0.505		0.02
PrL R - IL R	treatmt	1	6	2.06	0.201		0.098
PrL R - IL R	time	2	12	1.238	0.324		0.036
PrL R - IL R	treatmt:time	2	12	1.74	0.217		0.063
PrL R - M1 L	treatmt	1	6	5.198	0.063		0.062
PrL R - M1 L	time	2	12	0.803	0.471		0.057
PrL R - M1 L	treatmt:time	2	12	1.246	0.322		0.066
PrL R - M2 L	treatmt	1	6	0.2	0.67		0.005
PrL R - M2 L	time	2	12	0.135	0.875		0.007
PrL R - M2 L	treatmt:time	2	12	0.74	0.498		0.026
PrL R - NAcC L	treatmt	1	6	0.563	0.482		0.023
PrL R - NAcC L	time	2	12	0.736	0.499		0.012
PrL R - NAcC L	treatmt:time	2	12	1.094	0.366		0.048
PrL R - NAcC R	treatmt	1	6	4.717	0.073		0.028
PrL R - NAcC R	time	2	12	0.123	0.885		0.008
PrL R - NAcC R	treatmt:time	2	12	0.658	0.536		0.04
PrL R - NAcSh L	treatmt	1	6	0.006	0.943		0.00049
PrL R - NAcSh L	time	2	12	6.405	0.013	*	0.114
PrL R - NAcSh L	treatmt:time	2	12	0.033	0.968		0.00057
PrL R - NAcSh R	treatmt	1	6	3.768	0.1		0.06
PrL R - NAcSh R	time	2	12	0.536	0.598		0.017
PrL R - NAcSh R	treatmt:time	2	12	1.128	0.356		0.046
PrL R - PrL L	treatmt	1	6	1.779	0.231		0.036
PrL R - PrL L	time	2	12	3.222	0.076		0.131
PrL R - PrL L	treatmt:time	2	12	1.79	0.209		0.046
PrL R - S1 L	treatmt	1	6	1.054	0.344		0.009
PrL R - S1 L	time	2	12	0.345	0.715		0.024
PrL R - S1 L	treatmt:time	2	12	1.233	0.326		0.052
S1 L - AI L	treatmt	1	6	4.874	0.069		0.157
S1 L - AI L	time	2	12	4.181	0.042	*	0.143
S1 L - AI L	treatmt:time	2	12	0.138	0.873		0.005
S1 L - GIDI L	treatmt	1	6	1.124	0.33		0.052
S1 L - GIDI L	time	2	12	2.595	0.116		0.117
S1 L - GIDI L	treatmt:time	2	12	1.172	0.343		0.026
S1 R - AI L	treatmt	1	6	9.354	0.022	*	0.029
S1 R - AI L	time	2	12	0.334	0.723		0.011
S1 R - AI L	treatmt:time	1.14	6.83	3.711	0.094		0.121
S1 R - Cg1 L	treatmt	1	6	1.259	0.305		0.027
S1 R - Cg1 L	time	1.07	6.41	1.233	0.312		0.061
S1 R - Cg1 L	treatmt:time	2	12	0.377	0.693		0.017
S1 R - Cg1 R	treatmt	1	6	0.405	0.548		0.007
S1 R - Cg1 R	time	2	12	0.011	0.989		0.00075
S1 R - Cg1 R	treatmt:time	2	12	0.068	0.935		0.004
S1 R - CPu L	treatmt	1	6	1.217	0.312		0.068
S1 R - CPu L	time	2	12	1.317	0.304		0.061
S1 R - CPu L	treatmt:time	2	12	1.154	0.348		0.052
S1 R - CPu R	treatmt	1	6	0.000178	0.99		1.13E-05
S1 R - CPu R	time	2	12	0.922	0.424		0.029
S1 R - CPu R	treatmt:time	2	12	1.356	0.294		0.047
S1 R - GIDI L	treatmt	1	6	1.931	0.214		0.025
S1 R - GIDI L	time	1.15	6.91	0.104	0.791		0.007
S1 R - GIDI L	treatmt:time	2	12	4.22	0.041	*	0.192
S1 R - IL L	treatmt	1	6	6.619	0.042	*	0.131
S1 R - IL L	time	2	12	0.058	0.944		0.003
S1 R - IL L	treatmt:time	2	12	0.26	0.775		0.014
S1 R - IL R	treatmt	1	6	6.466	0.044	*	0.053
S1 R - IL R	time	2	12	0.471	0.636		0.021
S1 R - IL R	treatmt:time	2	12	1.921	0.189		0.101
S1 R - M1 L	treatmt	1	6	0.043	0.842		0.001
S1 R - M1 L	time	2	12	0.3	0.746		0.02
S1 R - M1 L	treatmt:time	2	12	0.483	0.629		0.025
S1 R - M1 R	treatmt	1	6	0.056	0.821		0.002
S1 R - M1 R	time	2	12	0.409	0.673		0.013
S1 R - M1 R	treatmt:time	2	12	0.735	0.5		0.026
S1 R - M2 L	treatmt	1	6	3.653	0.105		0.021
S1 R - M2 L	time	2	12	0.523	0.606		0.038
S1 R - M2 L	treatmt:time	2	12	1.527	0.257		0.063
S1 R - M2 R	treatmt	1	6	0.229	0.649		0.006
S1 R - M2 R	time	2	12	0.249	0.784		0.011
S1 R - M2 R	treatmt:time	2	12	1.313	0.305		0.049
S1 R - NAcC L	treatmt	1	6	0.048	0.833		0.00072
S1 R - NAcC L	time	1.13	6.78	3.415	0.107		0.194
S1 R - NAcC L	treatmt:time	2	12	0.697	0.517		0.038
S1 R - NAcC R	treatmt	1	6	2.012	0.206		0.078
S1 R - NAcC R	time	2	12	0.802	0.471		0.027
S1 R - NAcC R	treatmt:time	2	12	1.01	0.393		0.04
S1 R - NAcSh L	treatmt	1	6	0.299	0.604		0.027
S1 R - NAcSh L	time	2	12	9.512	0.003	*	0.227
S1 R - NAcSh L	treatmt:time	2	12	1.207	0.333		0.024

PrL R - CPu R	time	1	8	0.038	0.85		0.0008
PrL R - CPu R	treatmt:time	1	8	3.801	0.087		0.024
PrL R - GIDI L	treatmt	1	8	0.747	0.413		0.038
PrL R - GIDI L	time	1	8	1.809	0.216		0.027
PrL R - GIDI L	treatmt:time	1	8	0.568	0.473		0.008
PrL R - IL L	treatmt	1	8	1.306	0.286		0.029
PrL R - IL L	time	1	8	6.024	0.04	*	0.223
PrL R - IL L	treatmt:time	1	8	1.593	0.243		0.016
PrL R - IL R	treatmt	1	8	0.149	0.709		0.001
PrL R - IL R	time	1	8	1.778	0.219		0.035
PrL R - IL R	treatmt:time	1	8	0.064	0.807		0.001
PrL R - M1 L	treatmt	1	8	0.275	0.614		0.011
PrL R - M1 L	time	1	8	1.102	0.324		0.023
PrL R - M1 L	treatmt:time	1	8	0.214	0.656		0.002
PrL R - M2 L	treatmt	1	8	0.024	0.882		0.00093
PrL R - M2 L	time	1	8	3.253	0.109		0.086
PrL R - M2 L	treatmt:time	1	8	8.519	0.019	*	0.023
PrL R - NAcC L	treatmt	1	8	0.356	0.567		0.019
PrL R - NAcC L	time	1	8	1.997	0.195		0.057
PrL R - NAcC L	treatmt:time	1	8	0.743	0.414		0.014
PrL R - NAcC R	treatmt	1	8	1.886	0.207		0.064
PrL R - NAcC R	time	1	8	0.381	0.554		0.009
PrL R - NAcC R	treatmt:time	1	8	0.277	0.613		0.008
PrL R - NAcSh L	treatmt	1	8	2.79	0.133		0.07
PrL R - NAcSh L	time	1	8	0.285	0.608		0.009
PrL R - NAcSh L	treatmt:time	1	8	0.105	0.754		0.003
PrL R - NAcSh R	treatmt	1	8	0.103	0.757		0.001
PrL R - NAcSh R	time	1	8	0.338	0.577		0.012
PrL R - NAcSh R	treatmt:time	1	8	0.179	0.683		0.004
PrL R - PrL L	treatmt	1	8	1.361	0.277		0.04
PrL R - PrL L	time	1	8	12.141	0.008	*	0.288
PrL R - PrL L	treatmt:time	1	8	5.492	0.047	*	0.086
PrL R - S1 L	treatmt	1	8	0.245	0.634		0.016
PrL R - S1 L	time	1	8	0.192	0.673		0.001
PrL R - S1 L	treatmt:time	1	8	0.499	0.5		0.006
S1 L - AI L	treatmt	1	8	0.67	0.437		0.036
S1 L - AI L	time	1	8	3.285	0.108		0.05
S1 L - AI L	treatmt:time	1	8	2.933	0.125		0.027
S1 L - GIDI L	treatmt	1	8	3.298	0.107		0.13
S1 L - GIDI L	time	1	8	14.537	0.005	*	0.186
S1 L - GIDI L	treatmt:time	1	8	0.26	0.624		0.001
S1 R - AI L	treatmt	1	8	0.77	0.406		0.024
S1 R - AI L	time	1	8	1.867	0.209		0.035
S1 R - AI L	treatmt:time	1	8	0.243	0.635		0.004
S1 R - Cg1 L	treatmt	1	8	0.022	0.885		0.001
S1 R - Cg1 L	time	1	8	0.011	0.918		0.00015
S1 R - Cg1 L	treatmt:time	1	8	0.567	0.473		0.012
S1 R - Cg1 R	treatmt	1	8	0.086	0.777		0.004
S1 R - Cg1 R	time	1	8	1.536	0.25		0.017
S1 R - Cg1 R	treatmt:time	1	8	0.717	0.422		0.009
S1 R - CPu L	treatmt	1	8	0.045	0.837		0.002
S1 R - CPu L	time	1	8	0.14	0.718		0.005
S1 R - CPu L	treatmt:time	1	8	1.385	0.273		0.022
S1 R - CPu R	treatmt	1	8	0.014	0.91		0.0006
S1 R - CPu R	time	1	8	3.109	0.116		0.06
S1 R - CPu R	treatmt:time	1	8	0.069	0.799		0.001
S1 R - GIDI L	treatmt	1	8	1.019	0.342		0.045
S1 R - GIDI L	time	1	8	7.531	0.025	*	0.137
S1 R - GIDI L	treatmt:time	1	8	2.416	0.159		0.024
S1 R - IL L	treatmt	1	8	7.707	0.024	*	0.257
S1 R - IL L	time	1	8	1.869	0.209		0.049
S1 R - IL L	treatmt:time	1	8	0.208	0.661		0.004
S1 R - IL R	treatmt	1	8	10.547	0.012	*	0.229
S1 R - IL R	time	1	8	0.101	0.759		0.002
S1 R - IL R	treatmt:time	1	8	0.913	0.367		0.03
S1 R - M1 L	treatmt	1	8	6.56E-05	0.994		1.70E-06
S1 R - M1 L	time	1	8	0.176	0.686		0.008
S1 R - M1 L	treatmt:time	1	8	4.124	0.077		0.055
S1 R - M1 R	treatmt	1	8	0.142	0.716		0.007
S1 R - M1 R	time	1	8	2.032	0.192		0.083
S1 R - M1 R	treatmt:time	1	8	0.958	0.356		0.017
S1 R - M2 L	treatmt	1	8	0.622	0.453		0.033
S1 R - M2 L	time	1	8	0.056	0.82		0.002
S1 R - M2 L	treatmt:time	1	8	1.015	0.343		0.019
S1 R - M2 R	treatmt	1	8	2.304	0.168		0.084
S1 R - M2 R	time	1	8	0.476	0.51		0.019
S1 R - M2 R	treatmt:time	1	8	2.501	0.152		0.034
S1 R - NAcC L	treatmt	1	8	0.009	0.925		0.00039
S1 R - NAcC L	time	1	8	0.754	0.41		0.018
S1 R - NAcC L	treatmt:time	1	8	0.127	0.731		0.004
S1 R - NAcC R	treatmt	1	8	0.385	0.552		0.022
S1 R - NAcC R	time	1	8	0.197	0.669		0.003
S1 R - NAcC R	treatmt:time	1	8	0.057	0.817		0.002
S1 R - NAcSh L	treatmt	1	8	0.756	0.41		0.03
S1 R - NAcSh L	time	1	8	2.349	0.164		0.078
S1 R - NAcSh L	treatmt:time	1	8	0.969	0.354		0.016

S1 R - NAcSh R	treatmt	1	6	3.121	0.128		0.129
S1 R - NAcSh R	time	2	12	4.924	0.027	*	0.156
S1 R - NAcSh R	treatmt:time	2	12	0.31	0.739		0.004
S1 R - PrL L	treatmt	1	6	0.91	0.377		0.013
S1 R - PrL L	time	1.1	6.61	0.169	0.718		0.008
S1 R - PrL L	treatmt:time	2	12	0.399	0.679		0.019
S1 R - PrL R	treatmt	1	6	0.605	0.466		0.01
S1 R - PrL R	time	2	12	0.932	0.421		0.044
S1 R - PrL R	treatmt:time	1.1	6.59	0.165	0.72		0.008
S1 R - S1 L	treatmt	1	6	3.06	0.131		0.047
S1 R - S1 L	time	1.07	6.41	0.003	0.968		0.00011
S1 R - S1 L	treatmt:time	2	12	1.519	0.258		0.102

S1 R - NAcSh R	treatmt	1	8	0.015	0.905		0.0009
S1 R - NAcSh R	time	1	8	4.109	0.077		0.063
S1 R - NAcSh R	treatmt:time	1	8	0.003	0.959		3.74E-05
S1 R - PrL L	treatmt	1	8	0.922	0.365		0.06
S1 R - PrL L	time	1	8	0.015	0.904		0.00029
S1 R - PrL L	treatmt:time	1	8	4.706	0.062		0.03
S1 R - PrL R	treatmt	1	8	0.628	0.451		0.042
S1 R - PrL R	time	1	8	9.401	0.015	*	0.078
S1 R - PrL R	treatmt:time	1	8	1.69	0.23		0.032
S1 R - S1 L	treatmt	1	8	0.053	0.823		0.002
S1 R - S1 L	time	1	8	3.823	0.086		0.068
S1 R - S1 L	treatmt:time	1	8	0.036	0.855		0.00098
